# Evolutionary History of the *Smyd* Gene Family in Metazoans: A Framework to Identify the Orthologs of Human *Smyd* Genes in *Drosophila* and Other Animal Species

**DOI:** 10.1371/journal.pone.0134106

**Published:** 2015-07-31

**Authors:** Eduardo Calpena, Francesc Palau, Carmen Espinós, Máximo Ibo Galindo

**Affiliations:** 1 Program in Rare and Genetic Diseases, Centro de Investigación Príncipe Felipe (CIPF), Valencia, Spain; 2 Centro de Investigación Biomédica en Red de Enfermedades Raras (CIBERER), Instituto de Salud Carlos III, Valencia, Spain; University of Maryland, UNITED STATES

## Abstract

The *Smyd* gene family code for proteins containing a conserved core consisting of a SET domain interrupted by a MYND zinc finger. Smyd proteins are important in epigenetic control of development and carcinogenesis, through posttranslational modifications in histones and other proteins. Previous reports indicated that the *Smyd* family is quite variable in metazoans, so a rigorous phylogenetic reconstruction of this complex gene family is of central importance to understand its evolutionary history and functional diversification or conservation. We have performed a phylogenetic analysis of Smyd protein sequences, and our results show that the extant metazoan *Smyd* genes can be classified in three main classes, *Smyd3* (which includes chordate-specific *Smyd1* and *Smyd2* genes), *Smyd4* and *Smyd5*. In addition, there is an arthropod-specific class, *SmydA*. While the evolutionary history of the *Smyd3* and *Smyd5* classes is relatively simple, the *Smyd4* class has suffered several events of gene loss, gene duplication and lineage-specific expansions in the animal phyla included in our analysis. A more specific study of the four *Smyd4* genes in *Drosophila melanogaster* shows that they are not redundant, since their patterns of expression are different and knock-down of individual genes can have dramatic phenotypes despite the presence of the other family members.

## Introduction

The *Smyd* gene family is widespread in eukaryotes, and in mammals it has been related to epigenetic transcriptional control, development and cell proliferation [1, 2]. This family is defined on the basis of the presence of SET and MYND domains, and a cystein-rich post-SET domain. The SET domain is named after Su(var)3–9, Enhancer of Zeste and Trithorax; and it encodes a histone lysine methyltranferase activity [3]. In Smyd proteins, the SET domain is interrupted by the MYND domain (Myeloid translocation protein, Nervy, Deaf), a protein-protein interaction domain involved in recruiting of co-factors. Mammals have five *Smyd* genes; family members 1 to 3 are most similar to each other, with N-terminal SET-MYND domains and a C-terminal domain that contains TPR repeats. *SMYD4* has an additional TPR domain N-terminal to SET-MYND, and *SMYD5* does not contain TPR domains, has a C-terminal region without known domain homology in other proteins, and ends in a glutamic acid-rich tract.

The best characterized family members are human *SMYD1-3*. SMYD1 has histone 3 methyltransferase activity [4], is expressed in mesoderm and involved in muscle and heart development [5–7]. SMYD2 is also a histone 3 methyltranferase [8], but in addition it can methylate other proteins such as p53 [9], Rb [10] and HSP90 [11–13]. SMYD3 can methylate histones 3 4 and 5 [14, 15] and its expression has been found to correlate with proliferation of cancer cells [16]. Although the three proteins can have different functions, there are also overlaps between them. For example *SMYD3* is also necessary for the development of skeletal and cardiac muscle [17]; and *SMYD2* and *SMYD3* cooperate in estrogen receptor-mediated gene expression [18, 19].

In comparison with SMYD1-3, little is known of SMYD4 and SMYD5. SMYD4 has been related to breast cancer [20], and work from our group has revealed that mutations in SMYD4 could be related to an inherited rare neuropathy (unpublished data). In order to investigate the molecular function of SMYD4 and SMYD5, researchers have used Drosophila melanogaster as a further model organism. A putative SMYD4 homolog, CG14122, was identified in D. melanogaster [21]. Its gene product is expressed in muscle and it has a role in transcriptional repression through the recruitment of histone deacetylases. As for SMYD5, it has been shown that it works in the genetic program of the immune response in Drosophila (ortholog gene CG3353) and vertebrates [22].

But these two examples in which *Drosophila* has been used to understand the role of a *Smyd* gene illustrate the need to establish a rigorous phylogeny of gene families before we determine orthology relationships. *CG14122* was singled out among all the possible the *SMYD4* homologs and renamed *Smyd4*, despite the fact that there were several other candidates [21]. Subsequent research has confirmed that the *Smyd* family has suffered extensive lineage-specific expansion (LSE) in insects [23]. Still, no major effort has been done in defining the *Smyd* family beyond the examples we have mentioned in vertebrates and insects, so we do not know the family members present in the different animal phyla, and their evolutionary history.

The number of known rare diseases is in constant increase, but the resources that can be allocated to each one of them do not grow at the same rate. Therefore, one of the strategies that can be used in research on rare diseases is to use non-murine model organisms that are inexpensive and technically approachable, such as *Drosophila melanogaster*. But the use of model organisms has to be based in the careful establishment of molecular and functional homologies. The determination of the whole collection of homologous sequences and the establishment of their phylogenetic relationships is fundamental to understand their molecular evolution, to identify true orthologs and to classify them in sub-groups that probably reflect functional differences [24]. In many cases the identity in sequence and function of single genes, and even whole pathways, is evident and it has been very useful in elucidating their function in humans. Using *Drosophila* as an example, this species has single orthologs for Parkinson disease-related genes *PINK1* and *PARK2*, and has been used to study the mitochondria-related pathomechanisms in Parkinsonism [25–27].

But the presence of clear one-to-one orthologs in the different species is not always the case. During evolution there are dramatic changes in gene number and structure, such as changes in ploidy, domain rearrangements and variations in gene copy number, both expansions and losses [28–30]. Copy number expansion can give rise to so-called gene families, groups of genes with more than one copy within the same genome that are related by a common ancestry, and similar among them by sequence homology and general domain organization. Once the genes have duplicated, the new copies can undergo different fates [31–34]. One of the copies can accumulate mutations, becoming a non-functional pseudogene, or disappear altogether. The new copies can conserve the gene function, therefore increasing the level of expression. An interesting consequence for genes that are expressed in different tissues is the sub-functionalization of each copy in a subset of the domain of original gene, which could result in reproductive isolation of populations. Finally, one of the copies could acquire a new function.

Quite often, large scale LSE are related with the acquisition of new functions required for adaptation of the species to their environment [35]. LSE often involve structural proteins, response to stress or pathogens, and signaling pathways. One particularly striking case that has been recently published is the genome of the *Mesobuthus martensii* scorpion, containing around 30,000 genes, more than any arthropod sequenced to date despite the fact that scorpions are considered living fossils among chelicerates [36]. Among the expanded families are those involved in basic metabolic pathways, signaling and stress response pathways, neurotoxins and cytochrome P450.

In this scenario of gene gain, loss and shuffling, it is evident that identifying the two orthologs of a particular gene in different species can be sometimes difficult, if they exist at all. In order to see if *Drosophila* could also be a good model for *SMYD4* gene function, we have reconstructed the phylogeny of *Smyd* genes in representative animal phyla. Our phylogeny will also be useful to understand the evolution of the *Smyd* family, to identify animal model organisms for other human *SMYD* genes and, in a wider scenario, to contribute to the understanding of gene expansion in evolution.

## Results

### A phylogeny of Smyd proteins in metazoans

To construct a phylogeny of the Smyd family in metazoans, we chose species which represent a wide set of animal phyla, and for which there is a genome project with acceptable genome coverage and sequence quality (metazoan species in Table 1). Among the most basal animals, we included sequences for a placozoan species (*Trichoplax adhaerens*) and two cnidarians (*Hydra magnipapillata* and *Nematostella vectensis*). Among protostomes, we included sequences of the two main branches, Lophotrochozoa (the mollusk *Lottia gigantea*), and Ecdysozoa (the crustacean *Daphnia pulex* and the insects *Drosophila melanogaster*, *Apis mellifera* and *Anopheles gambiae*). As representatives of the deuterostomes we included a hemichordate (*Saccoglossus kowalevskii*), a tunicate (*Ciona intestinalis*), a cepalochordate (*Branchiostoma floridae*) and four vertebrate species (*Homo sapiens*, *Gallus gallus*, *Xenopus tropicalis* and *Danio rerio*).

**Table 1:** Species included in the phylogenetic study of Smyd family proteins.

**Metazoan species**	
*Trichoplax adhaerens*	Metazoa; Placozoa
*Hydra magnipapillata*	Metazoa; Cnidaria; Hydrozoa
*Nematostella vectensis*	Metazoa; Cnidaria; Anthozoa
*Lottia gigantea*	Metazoa; Mollusca;Gastropoda
*Daphnia pulex*	Metazoa; Arthropoda; Crustacea
*Drosophila melanogaster*, *Apis mellifera*, *Anopheles gambiae*	Metazoa; Arthropoda; Hexapoda
*Saccoglossus kowalevskii*	Metazoa; Hemichordata; Enteropneusta
*Ciona intestinalis*	Metazoa; Chordata; Tunicata
*Branchiostoma floridae*	Metazoa; Chordata; Cephalochordata
*Homo sapiens*, *Gallus gallus*, *Xenopus tropicalis*, *Danio rerio*	Metazoa; Chordata; Vertebrata
**Non-metazoan species**	
*Saccharomyces cerevisiae*	Fungi; Ascomycota; Saccharomycotina
*Arabidopsis thaliana*	Viridiplantae; Magnoliophyta; Brassicales
*Capsaspora owczarzaki*	Opisthokonta; Filasterea
*Monosiga brevicollis*	Opisthokonta; Choanoflagellida;

With the aim to build a comprehensive set of sequences of Smyd proteins, we used BLAST searches, as described in the Materials and Methods section, and kept only those sequences that had high similarity and co-linearity through the SET-MYND core, which includes the interrupted SET domain, the MYND-type zinc finger and the post-SET domain. We did not use domain recognition software since our exploratory work showed that the most used platforms such as PFAM and PROSITE are not consistent, especially in the recognition of the MYND Zn finger domain. Surprisingly, these homology searches yielded a number of potential Smyd proteins much higher than expected. Insect species, for example, contained more than 10 Smyd genes each, which was an early indication that some *Smyd* genes had suffered LSE. In addition, some of these putative Smyd proteins had very low similarity to vertebrate Smyds.

**Fig 1:**
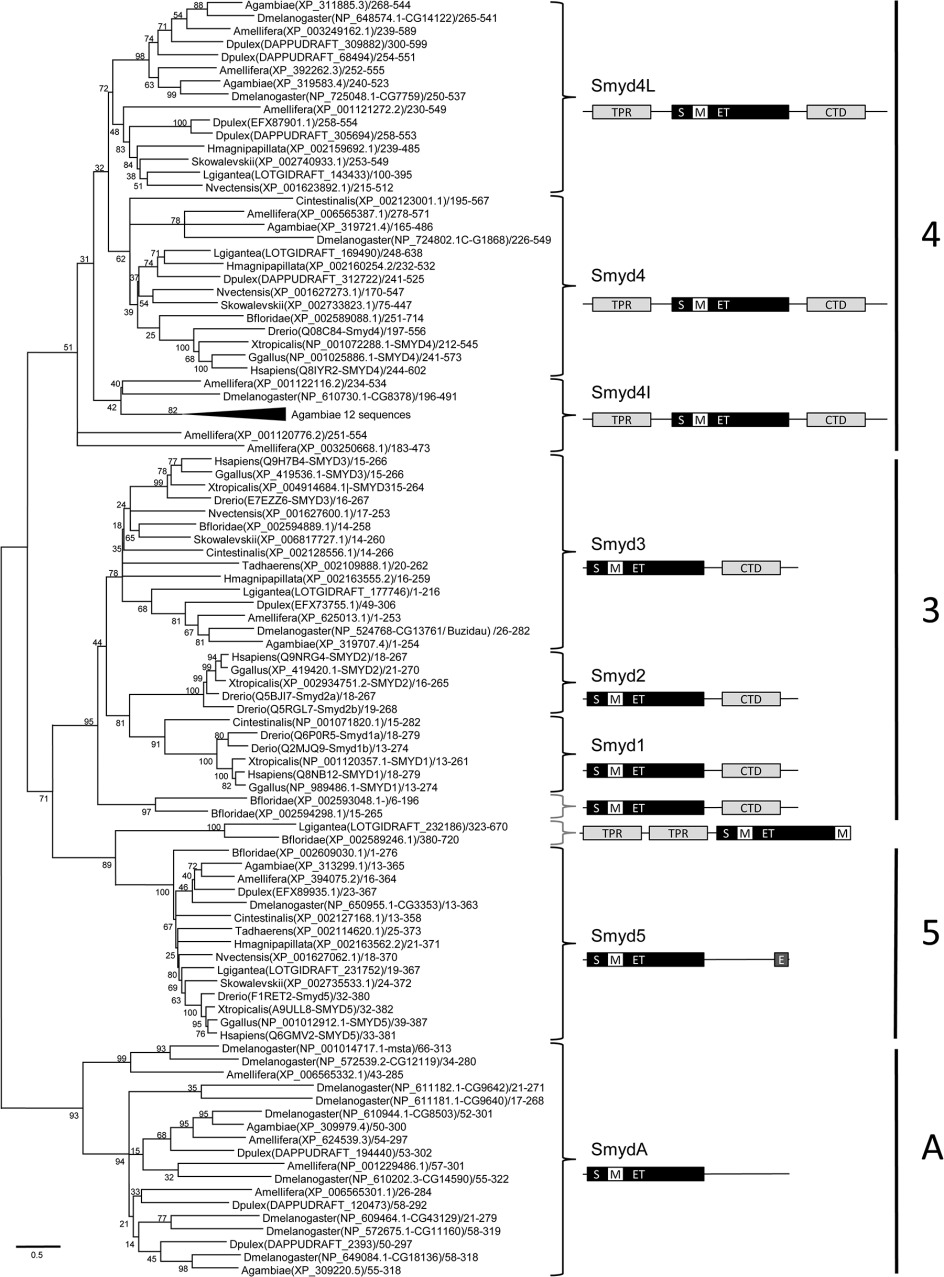
Phylogeny of metazoan Smyd proteins. The aligned sequences of the metazoan Smyd proteins were used to construct a phylogenetic tree by the maximum likelihood method. Branch lengths are proportional to the amount of genetic change measured as substitutions per site, the scale bar is shown below the tree. For each branch the bootstrap statistical support is indicated as percentage. For each sequence we indicate the species (i.e. *Homo sapiens* abbreviated as *Hsapiens*), accession number, gene name if available and residues in the alignment. The branch containing 12 sequences of *Anopheles gambiae* was compressed. The brackets indicate the major branches within the tree for which we illustrate the characteristic domain structure. The split SET domain is indicated by a black box labeled S/ET. The MYND zinc fingers are indicated with a white box labeled M. The C-terminal domains with a TPR fold and the N-terminal TPR domains are indicated by light grey boxes, and named CTD and TPR respectively. The Glu-rich, Glu- Asp-rich or Glu- Asp- Ser-rich domains are indicated by a dark grey box labeled E. On the right we indicate the classes defined on the basis of the phylogeny and domain structure.

**Fig 2:**
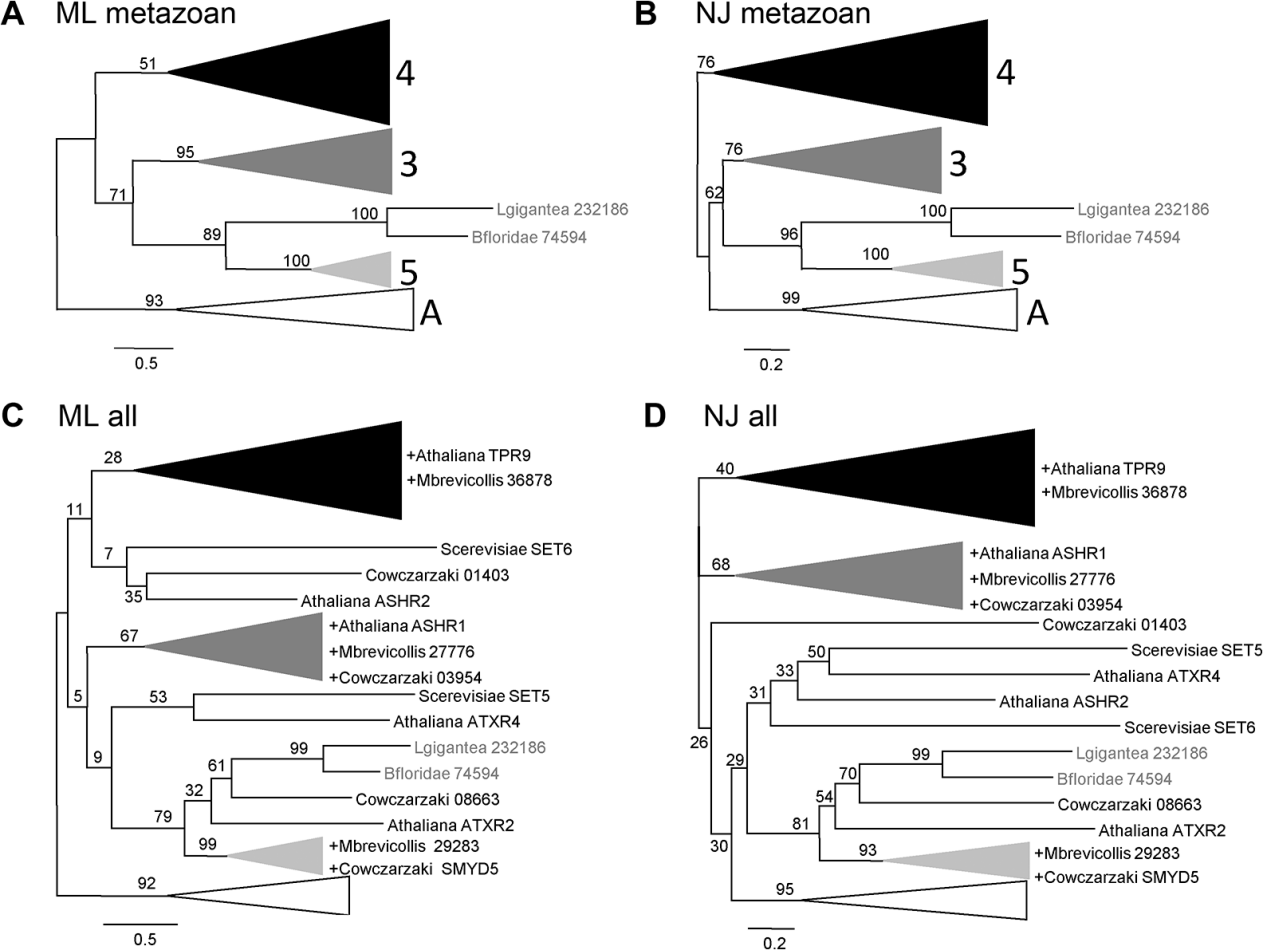
Schematic phylogenetic trees of the Smyd proteins. In all the panels sequences are indicated with abbreviated species name and annotated protein name. Branch lengths and bootstrap values are given as in Fig 1. (A, B) Phylogenetic trees obtained with the alignment of the metazoan Smyd proteins by the maximum likelihood (A) and neighbor joining (B) methods; the branches corresponding to the four main classes are compressed and the two sequences not included in one of these classes are indicated in grey. (C) Maximum likelihood and neighbor joining (D) phylogenetic trees obtained with the alignment of the extended dataset, which includes the sequences from the metazoans plus unicellular metazoan-related species *Capsaspora owczarzaki* and *Monosiga brevicollis*, the plant *Arabidopsis thaliana* and the yeast *Saccharomyces cerevisiae*. The non-metazoan species are indicated in black type, and those that are grouped within one of the classes are indicated in the compressed branch with the name preceded by +.

The sequences were aligned using Clustal Omega, and the aligned sequences were trimmed, leaving only the SET-MYND core (S1 File). To ensure that the topology we obtained was consistent, we used two different methods for phylogenetic reconstruction, maximum likelihood (ML) and neighbor joining (NJ). The ML tree, with a mosquito-specific branch compressed, is shown in Fig 1; and ML and NJ schematic trees with the main groups compressed are shown in Fig 2A and 2B. The uncompressed trees are shown in S1 Fig. Both trees yielded the same sequence groups, and it is clear that the vertebrate complement of *Smyd* genes, *Smyd* classes 1 to 5, is not the typical one for all the species. *Smyd1* is present only in chordates and *Smyd2* only in vertebrates. These two genes are most similar to *Smyd3*, for which there is a representative in all the species included in this study. Also, vertebrates have a single *Smyd4* gene but other species have at least one additional *Smyd4*-related gene, and quite often several of them. Finally, there is an arthropod-specific branch that contains members from the insect and the crustacean species, which we have called *SmydA*. There is a small branch containing one sequence from amphioxus and one sequence from the mollusk. The bootstrap values for the branches *Smyd3*, *Smyd5* and *SmydA* are very robust in both trees (Fig 2A and 2B). The bootstrap value supporting the *Smyd4* branch is weaker, but there is a further independent confirmation that these branches reflect common ancestry: although the alignment was performed only with the MYND-SET core, the domains not included in this alignment reflect similar protein architectures within each one of the four main groups (Fig 1). Based on the tree topology and on the domain organization we propose the subdivision of the *Smyd* family in three classes present in all or most of the species, and an arthropod-specific class.

### The *Smyd3* class

Our results confirm what was observed for vertebrates, that Smyd1 and Smyd2 proteins are most similar to each other, and both are most similar to Smyd3. *Smyd1* and *Smyd2* are exclusive of chordates and vertebrates respectively and probably arose from *Smyd3* by successive duplications. *Smyd3* would be the ancestral gene as it is present in all the species in this study. In addition, fish would have suffered further duplications of *Smyd1* and *Smyd2*. This class also includes the two *B*. *floridae*-specific genes, probably originated by two duplications independent from the ones giving rise to *Smyd1* and *Smyd2*. The class is defined by a domain organization as already described for Smyd1-3, with N-terminal SET and MYND domains and a C-terminal domain with a TPR fold [37, 38].

### The *Smyd4 class*

This is the most extended and complex class, and it comprises three major groups. Vertebrate *Smyd4* genes are contained in a branch that also has a representative from all species, with the exception of *T*. *adhaerens*, which we have called the *Smyd4* group. In addition, all species except *T*. *adhaerens* and the chordates have at least a second gene similar to *Smyd4*. Therefore, it is likely that all animals had a second *Smyd4*–like copy that was lost in chordates, and expanded to 2–4 copies in arthropods, and we have called this group *Smyd4L*. In the group we have called *Smyd4I* there are further insect-specific genes, with the most dramatic example of LSE in the mosquito *A*. *gambiae*, containing 12 additional copies. Finally, there are two more *Smyd4*-related genes in the honeybee *A*. *mellifera*.

All these sequences share a common domain organization, which is the same as in vertebrate Smyd4, with an N-terminal TPR domain in addition to the SET, MYND and C-terminal ones. The topologies of our trees suggest that *Smyd4* and *Smyd4L* are ancient sister groups, but the origin the insect-specific genes is not clear. To reduce the noise introduced by the other *Smyd* genes, we aligned only the Smyd4 class proteins to see if the same three groups were reproduced (S2 File). The alignment contains fewer gaps than the one obtain with the previous dataset, and ML and NJ phylogenetic reconstructions confirmed the same groups. The two putative *A*. *mellifera* Smyd4I proteins fall within the Smyd4I branch (S2 Fig).

### The *Smyd5* class

*Smyd5* is the most evolutionarily parsimonious class, with one representative in all metazoan species included in the study. It also has a typical domain organization, with the absence of the C-terminal domain present in classes *Smyd3* and *Smyd4*, and the presence in vertebrates of a C-terminal stretch of acidic aminoacids, typically a poly-glutamic acid tract. In most species these tracts are composed of glutamic acid and aspartic acid, and in the most basal ones, placozoa and cnidaria, glutamic acid, aspartic acid and serine.

### The *SmydA* class

In both trees there is an arthropod-specific group. This branch contains crustacean and insect sequences with multiple copies in each species, three in *D*. *pulex*, four in *A*. *mellifera*, two in *A*. *gambiae* and nine in *D*. *melanogaster;* which have substantial differences with sequences from the other classes. The C-terminal region does not contain any features in common with the rest of *Smyd* classes, and the putative MYND-type Zn fingers have differences with the canonical sequence (see below). The tree topology regarding this branch with respect to the others is variable (Fig 2A and 2B).

### Other *Smyd* sequences

Two Smyd sequences from amphioxus and mollusk are so diverged that it is difficult to ascertain their origin. If we add Smyd protein sequences from the mollusks *Crassostrea gigas* and *Aplysia californica*, which have not been included in the present dataset, there are further sequences from these species that also group in this branch (data not shown). In addition to the divergence in the SET/MYND core, these proteins have one or two TPR domains in their N terminus, and an additional MYND zinc finger in their C terminus.

### Evolutionary relationship with other SET and MYND genes from non-metazoan species

Our trees suggest that classes *Smyd3* and *Smyd5* are closest to each other and more distantly related to *Smyd4*, but the origin of *SmydA* and the mollusk/amphioxus group is not clear. In order to shed light on these questions, and to find out more about how the different classes originated, we extended the phylogeny by introducing Smyd sequences from other species. First, using the same similarity and co-linearity criteria employed in the metazoan dataset, we searched for sequences in two species basal to the metazoans, the choanoflagellate *Monosiga brevicollis* and the filose ameboid *Capsaspora owczarzaki*, where we found 3 and 4 sequences respectively. *Smyd* genes have also been found in plants [39]. In *Arabidopsis thaliana* five candidate Smyd proteins were defined: ATXR1, ATXR2, ATXR4, ASHR1 and ASHR2. We searched the *A*. *thaliana* protein sequence collection using the same criteria used to construct the original data set and identified only the last four, which is consistent with the fact that ATXR1 does not contain a Zn finger [39]. In addition, we had a fifth hit, the protein TPR9, which also has recognizable SET and MYND domains (see below). Finally, it has been proposed that animal and plant *Smyd* genes originated from ancestral SET genes that also gave rise to yeast *SET5* and *SET6*, in which the only recognizable domain was the SET domain [40]. Therefore, we decided to include *Saccharomyces cerevisiae* SET5 and SET6 as candidate outgroups to root our trees.

Surprisingly, ML and NJ trees obtained with the alignment of the extended set of sequences (alignment in S3 File, compressed trees in Fig 2C and 2D; complete trees in S3 Fig) revealed that classes *Smyd3* and *Smyd4* pre-date the appearance of metazoans. One protein sequence from each *A*. *thaliana* and both unicellular relatives of the metazoans now groups within the Smyd3 sequences; and sequences from *A*. *thaliana* and *M*. *brevicollis* with the Smyd4 class. These groupings are also supported by the presence and position of other domains not included in the alignment: C-terminal domain in both types of sequences, and an additional N-terminal TPR domain in Smyd4. There are no plant representatives of Smyd5, but this class contains one sequence from each *M*. *brevicollis* and *C*. *owczarzaki*, indicating that this class originated after the split from plants and fungi, but before the appearance of metazoans. These two sequences also have Smyd5-characteristic C-terminal domains rich in glutamic acid, aspartic acid and serine. One sequence from *C*. *owczarzaki* also groups with the atypical sequences from *L*. *gigantea* and *B*. *floridae*. Similar to them, it also has additional TPR repeats N-terminal to the SET-MYND core, but not an extra C-terminal MYND finger. The rest of sequences from *A*. *thaliana* and *S*. *cerevisiae*, and one sequence from *C*. *owczarzaki*, do not have further domains or noticeable features outside the SET-MYND core. It is surprising that none of these, especially the *S*. *cerevisiae* SET protein sequences, appear as an outgroup. A closer inspection of yeast SET5 and SET6 revealed that these proteins also contain putative Zn fingers similar to the MYND type ones (see below). It is thus possible that these are Smyd proteins rather than SET-only. In fact, it had been proposed that at least SET5 was actually a Smyd protein [41].

### A variety of MYND-like Zn fingers

**Fig 3:**
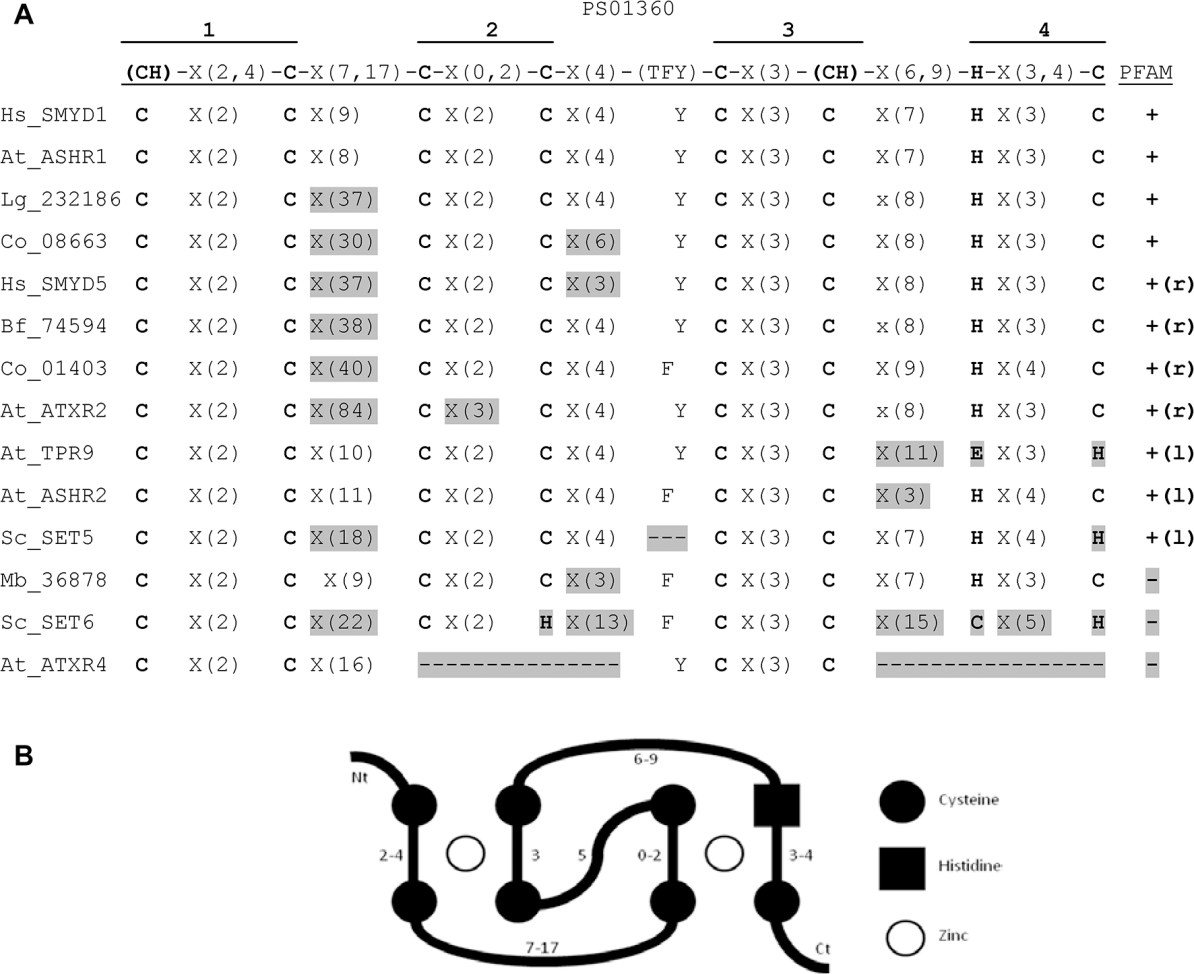
Sequence analysis of the confirmed and putative MYND domains. (A) For the protein sequences indicated on the left, the MYND Zn finger within the interrupted SET domain is compared to the PROSITE signature PS01360 and to the MYND hidden Markov model PFAM signature. For the PROSITE signature, the formula is indicated above with Zn-ligand residues in bold and highlighting the four Zn-ligand pairs. We have highlighted in grey all departures from the signature, including residues that do not match the consensus and stretches of the wrong length. For the PFAM model we indicate whether the sequence is a perfect match (+), a partial match of the right or left portions (+(r) and +(l) respectively), or not a match at all (-, highlighted grey). (B) Scheme of the cross-brace disposition of a MYND-type Zn finger, with the most common Zn-ligand residues depicted in black and Zn ions as empty circles; the numbers indicate the number of aminoacids between Zn-ligand residues according to the PS01360 formula.

The possible presence of a MYND or MYND-related Zn finger in the yeast proteins highlights the problem of what should be the criterion to classify a protein within the Smyd family. All the sequences analyzed in this work contained a SET domain according to the standard domain prediction algorithms PROSITE and PFAM. The MYND domain is more problematic, since only some of the proteins have a canonical Zn finger of the MYND type, others have partial domains representing only the left or the right portions of the domain, and a few of them are PROSITE- and PFAM-negative for the MYND domain. For this reason we decided to perform a more detailed search for the presence and identity of Zn fingers in these sequences. In Fig 3 we present the study of selected confirmed or candidate Zn fingers, the full sequences of these Zn fingers are shown in S4 Fig. As accepted Smyd proteins we included human SMYD1 and SMYD5. In addition, we searched for candidate Zn fingers in all the sequences from *A*. *thaliana* and *S*. *cerevisiae*, and in all the sequences that do not belong to one of the four main groups (non-compressed branches in Fig 2C and 2D). Finally, we included *M*. *brevicollis* 36878 as an example of a protein that clearly belongs in one of the groups, but for which none of the domain prediction suites detect a MYND domain.

We studied the candidate MYND type Zn fingers as defined by the PROSITE signature pattern PS01360 and by PFAM Hidden Markov Model PF01753 (Fig 3A). In those sequences were the MYND domain was not identified by either algorithm, or there was only a partial match, we searched manually for the full candidate Zn fingers within the interrupted SET domain, based on the conserved residues in the alignment (S3 File); and the corresponding sequences were compared with the PROSITE signature. Zn fingers of the MYND type are formed by four pairs of Zn-ligand residues, usually cysteine and more rarely histidine [42, 43]. These four pairs adopt a cross-brace disposition, similar to the RING type Zn fingers, so that the first Zn ion is bound by pairs 1 and 3 and the second Zn ion by pairs 2 and 4 (Fig 3B). The spacing between the Zn-ligand residues within each pair is relatively short and invariable, while the spacing between Zn-ligand pairs is longer and more variable.

Human SMYD1 contains a canonical MYND Zn finger that has been experimentally demonstrated in structural studies [37] and, as expected, it is a perfect match in both models. Human SMYD5 has two mismatches, a very long spacer between pairs 1 and 2, and a shorter spacer between pairs 2 and 3. Apart from SMYD1, the only perfect match in this set of sequences is *A*. *thaliana* ASHR1. Most of the other Zn fingers are not completely identified by at least one of the models, and show only a partial match. Most of the mismatches with the PROSITE signature are in the spacing regions between Zn-ligand pairs, especially in the region between Zn-ligand pairs 1 and 2. Among these, ATXR2 would have an unusually long spacer comprising 84 residues. In a few of them it would be required a change in the identity of the Zn-ligand residue, four cases of histidine instead of cysteine, one of cysteine instead of histidine, and also in one case we can only form a full Zn finger by using as a Zn-ligand glutamic acid, which is a less typical component of Zn fingers. Finally, there are three proteins that are not recognized at all by PFAM, including *M*. *brevicollis* 36878 although it has only a minor mismatch with the PROSITE signature. *S*. *cervisiae* SET6 contains a putative Zn finger with a C3H-C3H structure that would differ significantly from both models; and A. thaliana ATXR4 contains a putative Zn finger with only two coordinating pairs that could be equivalent to pairs 1 and 3 in a MYND-type Zn finger, and thus it would be able to bind only one Zn ion.

Although PFAM and PROSITE do not always indentify full MYND domains, it is most likely that all the proteins in our dataset contain a MYND or MYND-related Zn finger, with the exception of ATXR4, which would contain a half-MYND Zn finger. All the sequences analyzed would be theoretically able to form cross-brace Zn fingers. As a further proof of this, we analyzed the secondary structure predictions of these sequences (S4 Fig). Canonical MYND-type Zn fingers contain two anti-parallel β-sheets, comprising the residues just upstream of the coordinating pairs 2 and 3, and one α-helix spanning coordinating pairs 3 and 4 and the intervening region [43]. These structural features are depicted over SMYD1 in S4 Fig. When the SMYD1 sequence was subject to four different modes of secondary structure prediction, none of them detected the first β-sheet, but most of them identified the second one and the C-terminal α-helix. When we run the same prediction algorithms over the rest of the sequences, the second β-sheet was predicted in all of them by at least one algorithm, most often three or all four of them; and consistent α-helix tracts were also predicted in the right C-terminal position. The exception is A. thaliana ASHR2, with no α-helix prediction, and the smaller Zn finger in ATXR4. In summary, the presence of putative Zn-ligand pairs of residues, the spacing within and between them, and the secondary structure predictions are consistent with the presence of a cross-brace structure Zn finger of the MYND type or similar within the interrupted SET domain.

### The origin of the atypical TPR-SMYD group

The branch comprised by three Smyd sequences from the mollusk, amphioxus and filose ameboid species seems to be closer to the *Smyd5* class but it poses a problem when trying to reconstruct the evolutionary history of the *Smyd* genes in metazoans since it is present only in these three species. For this reason we decided to look further into their origin. The two alternatives are that all three sequences have a common ancestor or that they have somehow converged. Convergence by mutation would be extremely unlikely, but bearing in mind that this is a multi-copy gene family they could have originated by three independent events of recombination of the same two *Smyd* genes in similar positions. Since the MYND Zn finger in all three is more similar to the Smyd5 class (long spacer between Zn-coordinating pairs 1 and 2, Fig 3A) and they possess N-terminal TPRs like the Smyd4 class, a likely scenario would be a recombination between these two genes. If these recombinations had happened within the SET-MYND core used for the alignment, this would explain why they group together in the same branch and away from the two parental classes. To investigate this possibility, we performed BLASTP searches with all three proteins against the non-redundant collection of protein sequences from all metazoans. We used as queries the full protein, the MYND-SET core, the N-terminal SET plus MYND portion and the C-terminal SET portion.

With *C*. *owczarzaki* 086663 we consistently got hits to Smyd5 proteins from all phyla, so this protein doesn´t seem to be the result of a recombination within the core. This gene could have originated from a *Smyd5*-related gene that had acquired the TPR domain through recombination with other genomic region. The situation is much more intriguing with the *B*. *floridae* and *L*. *gigantea* proteins. Even if they originated as two independent recombinants within the core region of two *Smyd* genes, they still would have to acquire the N-terminal TPR domains and the C-terminal MYND domains. Moreover, when we perform the BLASTP searches with the *B*. *floridae* sequences, the first hits are always the similar genes from the mollusks *L*. *gigantea*, *C*. *gigas* and *Aplysia californica*, all of them with the extra TPR and MYND domains. After these mollusk sequences, the following hits are metazoan Smyd5 proteins with the whole core or the N-terminal queries and the Smyd3 class proteins with the C-terminal query. The reverse is also true: BLASTP searches with the *L*. *gigantea* sequences always yields the *B*. *floridae* sequences first and then the Smyd5 or Smyd3 sequences from other metazoans. In conclusion, the sequences from amphioxus and mollusks seem to be genuinely more related to each other than to *Smyd* genes from closer species.

If we discard independent origins, there are two alternatives. First, there could have been a horizontal gene transfer from an ancestor of mollusks to cephalochordates or *vice versa*. Against this possibility are the facts that horizontal transfer events are extremely rare in eukaryotes, especially in animals [44, 45], and that the genomic structures of the corresponding genes are very different: three exons in mollusks and nine exons in *B*. *floridae* (S5 Fig). A second possibility is that these genes represent a class that was present in the bilaterian ancestor and has suffered successive losses in most animal lineages, but kept in cephalochordates and mollusks.

Before these or other explanations can be further explored, we need to have access to more genome sequences and better annotations. Many of the protein sequences studied are predictions, and the evidence suggests that the available annotations are incomplete. As an illustration, we performed TBLASTN searches using the protein sequences of these atypical proteins against the genomic contig sequences (S5 Fig). In *L gigantea* we obtain hits corresponding to the *232186* locus, the predicted *160845* locus, which is a duplication of the last exon of *232186*, and a genomic region without annotated genes that would contain a second copy with identical gene structure. Similarly, in *B*. *floridae* there would be a second gene with the same structure as the annotated gene.

### Full catalog and classification of *D*. *melanogaster* Smyd proteins

In *D*. *melanogaster* the full *Smyd* catalog comprises 15 genes, listed in Table 2 with the corresponding group assignation. *Smyd* genes in the classes *Smyd3* (1 member), *Smyd4* (4 members) and *Smyd5* (1 member) match the typical domain structure of their counterparts in other animal species. Over half of the *Smyd* genes (9 members) belong to the *SmydA* class. As already explained above, SmydA proteins do not share domains with the other three classes, outside the SET/MYND core. A closer examination of the putative MYND domains revealed that the Zn finger in the interrupted SET domain is less similar to the MYND signature than the members of the other three groups (S6 Fig). For example they all lack the conserved tyrosine or phenylalanine residues before the third Zn-ligand pair (Fig 3 and S6 Fig). In addition, two of them (*CG8503* and *CG14590*) have a second, perfectly canonical MYND finger just N-terminal to the SET domain (S6 Fig).

**Table 2:** *Smyd* genes in *Drosophila melanogaster*. The name in bold type is the one we propose as main name of the gene, the other names would be synonyms.

CLASS	ANNOTATION	CURRENT NAME	PROPOSED NAME/SYNONYM
*Smyd3*	*CG13761*	***buzidau***	*Smyd3*
*Smyd4*	*CG1868*		***Smyd4-1***
	*CG14122*	*Smyd4*	***Smyd4-2***
	*CG7759*		***Smyd4-3***
	*CG8378*		***Smyd4-4***
*Smyd5*	*CG3353*		***Smyd5***
*SmydA*	*CG8503*		***SmydA-1***
	*CG18136*		***SmydA-2***
	*CG43129/CG17086*		***SmydA-3***
	*CG11160*		***SmydA-4***
	*CG14590*		***SmydA-5***
	*CG9642*		***SmydA-6***
	*CG9640*		***SmydA-7***
	*CG33548*	*msta*	***SmydA-8***
	*CG12119*		***SmydA-9***

Some of the *Drosophila Smyd* genes have already been described and named in the scientific literature. It is not our intention to change the names that are already in use, but we propose to establish a rational nomenclature that reflects their molecular nature and homology relationships. When genes already have a common name (*buzidau*) the serial name (*Smyd3*) could be used as a synonym. An exception would be *CG14122*, currently known as *Smyd4*, since this name could drive to confusion. In this case we would ask from the *Drosophila* community and the genomic annotation consortia that the current name is left as a synonym and the *Smyd4* genes are renamed *Smyd4-1* to *-4*, with *Smyd4-1* assigned to *CG1868* since it is closest to human *SMYD4*.

### Functional characterization of the *D*. *melanogaster Smyd4* genes

*D*. *melanogaster* has four *Smyd4* genes, one in the *Smyd4* group, two in the *Smyd4L* group and one in the *Smyd4I* group. We ignore whether the multiple copies compared to other species are redundant or functionally relevant, for example to achieve high levels of expression, anatomical subdivision of their function, or to sub-specialization of their roles. To explore this, we studied their patterns of expression and the effects of down-regulation. The objective is not a detailed study of their function, but to define the biological relevance of gene expansion within the *Smyd4* group.

**Fig 4:**
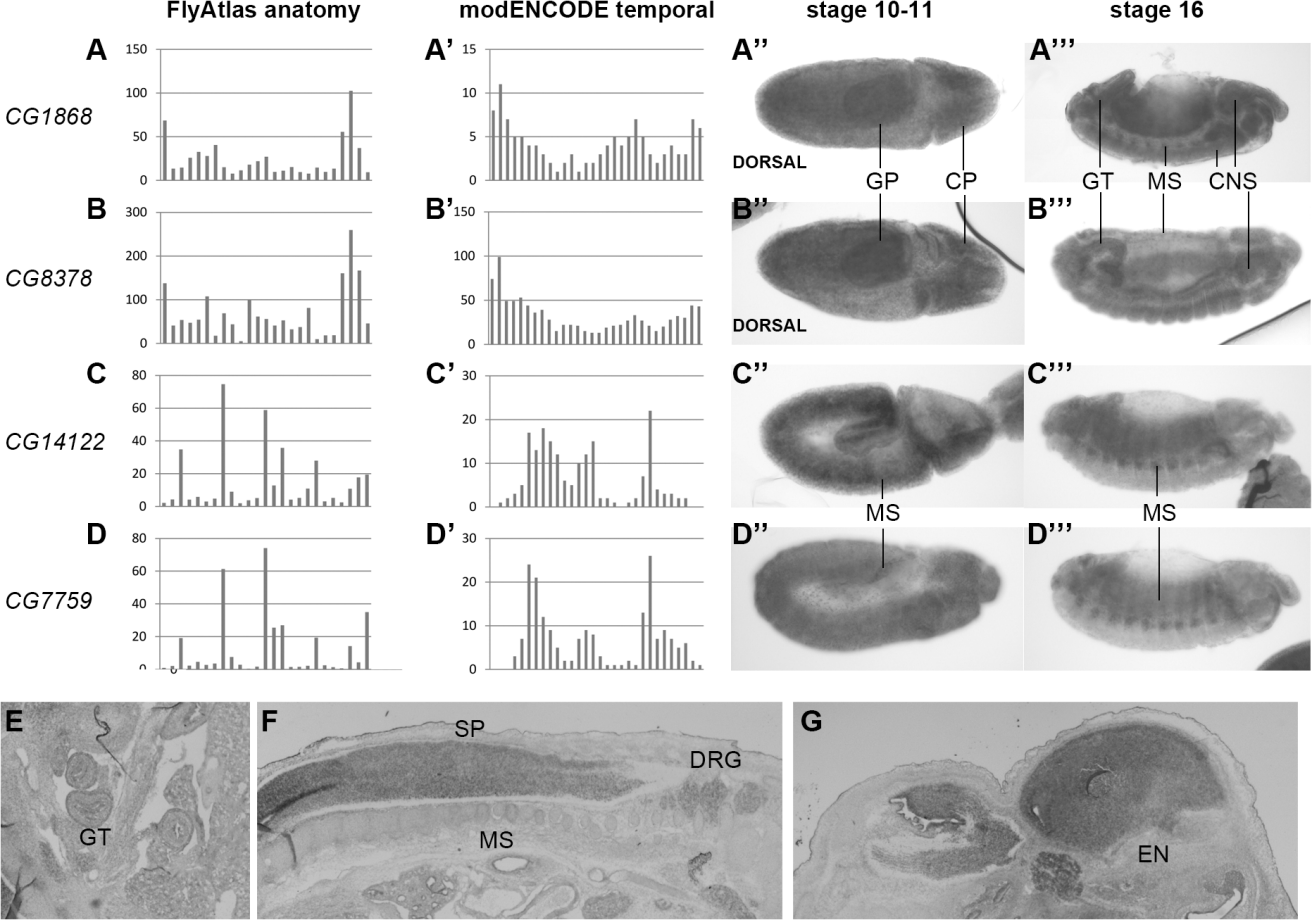
Transcriptional expression profile of *Drosophila* and mouse *Smyd4* class genes. We show a transcriptional study for *Drosophila melanogaster* genes *CG1868* (A-A”‘), *CG8378* (B-B”‘), *CG14122* (C-C”‘) and *CG7759* (D-D”‘) and *Mus musculus Smyd4* (E-G). For the *Drosophila* genes we show high throughput data from two consortia, the anatomical expression profile of FlyAtlas (A-D) and the temporal expression profile of modENCODE (A’-D’). For these genes we also determined the expression pattern by i*n situ* hybridization in embryos (anterior to the right, lateral view except where indicated). We show expression in extended germ band (A”-D”) and in stage 16 embryos (A”‘-D”‘). The ventral nerve cord in B”‘ is not visible as the embryo is slightly tilted. We also determined the expression of *Smyd4* in a 14.5E mouse embryo section. We show sections of the abdomen (E), dorsal trunk (F) and head (G). Abbreviations are used for the tissues where expression is detected as follows: GP, gut primordium; CP, cephalic primordium of the central nervous system; GT, gut; MS, mesoderm; CNS, central nervous system; SP, spinal cord; DRG, dorsal root ganglia; EN, encephalon.

To establish a transcriptional profile of the *Smyd4* genes we used two complementary approaches. First, there are high-throughput analyses of gene expression by public consortia. We downloaded the anatomical gene expression data by FlyAtlas (http://flyatlas.org/), comprising the expression levels of each gene in a collection of 25 larval and adult tissues. In addition, we also used the data generated by modENCODE (http://www.modencode.org/) corresponding to expression levels at 30 time points along development, from fertilization to adult. These data provide a regulated expression fingerprint that can be used to compare expression profiles between genes. Second, we performed *in situ* hybridization with specific probes corresponding to all four genes in developing embryos. The results of these studies are shown in Fig 4. High-throughput expression analyses (Fig 4A–4D, 4A’–4D’) clearly show that the transcriptional profiles divide these genes into two groups: *CG1868* with *CG8378*, and *CG14122* with *CG7759*. Although within each group the absolute levels of expression for each data point are different, the relative levels between data points are remarkably similar. Therefore, each group has a characteristic temporal and spatial modulation. *In situ* hybridization reveals the same groups. In extended germ band embryos *CG1868* and *CG8378* are expressed more intensely in the presumptive primordia of the cephalic central nervous system and of the posterior gut (Fig 4A” and 4B”), and in stage 16 embryos expression is evident in central nervous system and gut, and more weakly in the mesoderm-derived somatic muscles (Fig 4A”‘ and 4B”‘). In contrast, *CG14122* and *CG7759* are expressed in mesoderm both in extended germ band (Fig 4C” and 4D”) and stage 16 embryos (Fig 4C”‘ and 4D”‘), as already described for *CG14122* [21].

Very little is known about the vertebrate Smyd4 protein. To find out how its expression compares to the *D*. *melanogaster* genes we performed in situ hybridization in mouse embryos. *Smyd4* transcripts are expressed at relative low levels in mesoderm derivatives and digestive system (Fig 4E and 4F), and at high levels mainly in the nervous system (spinal cord, dorsal root and encephalon; Fig 4F and 4G). Therefore, the overall pattern of *Smyd4* expression is more reminiscent of *CG1868* and *CG8378* among the *Smyd4* genes in *D*. *melanogaster*. The four *Drosophila* proteins of the Smyd4 class have a predominantly cytosolic localization in transfected S2 cells and in the case of CG14122 also in developing muscle cells [21]. In order to find out whether this is also true of the vertebrate Smyd4 proteins, we made a fusion of the human SMYD4 protein with GFP and made use of the *Gal4/UAS* technique [46] to drive expression in different *Drosophila* tissues. When the fusion protein was expressed in neurons under the control of *elav-Gal4*, it seemed to be mostly cytosolic, as in S2 cells (S7A and S7A’ Fig). In contrast, when we expressed SMYD4-GFP in the muscle using the *Mhc-Gal4* driver the fusion protein was localized strongly to the myofibrils and the nuclei (S7B and S7B’ Fig). Within the sarcomeres, it was strongly localized in the M line, and more weakly in the Z line. There is an apparent contradiction in the fact that *Drosophila* Smyd4 proteins are predominantly cytoplasmic in S2 cells and embryonic muscle cells [21], while the SMYD4-GFP fusion is both cytoplasmic and nuclear. This nuclear localization is not an effect of the presence of GFP, since the same fusion is cytoplasmic in neurons (S7B and S7B’ Fig). In fact, nuclear localization is to be expected due to the function of Smyd proteins as histone modifiers and/or transcriptional repressors. Murine Smyd1 is excluded from the nucleus during myogenesis, but it is localized in the sarcomeric M bands and nucleus in adult muscle [7, 47], so the most likely reason for this difference is that CG14122 localization was determined in embryonic immature muscle cells, while we have studied mature muscle.

As an indicator of the functional relevance of each one of the *Smyd4* genes of *D*. *melanogaster*, and to detect any functional redundancies, we tested knock-down of each one of them by RNAi. The rationale is that if these genes are redundant, RNAi of each individual gene should not result in an abnormal phenotype. In this case we used two different *Gal4* drivers to express the RNAi constructs: *Act5C-Gal4* uses a ubiquitous *Actin* promoter, and therefore knocks down expression in all cells from early development, and *GMR-Gal4* is a retina-specific promoter expressed in post-mitotic cells in this tissue in late development.

**Fig 5:**
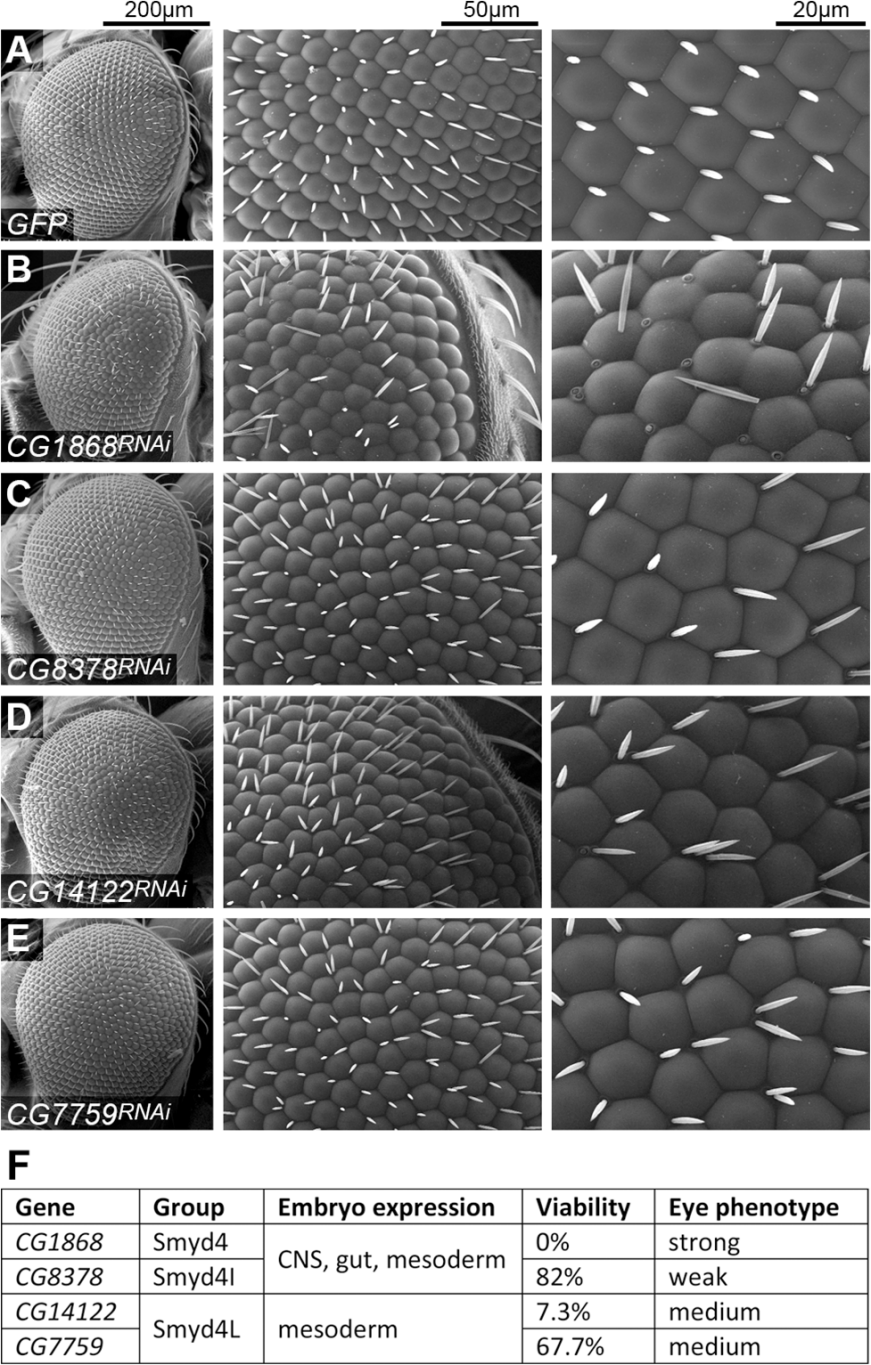
Knock down by RNA interference of the *Drosophila Smyd4* class genes. Scanning electron micrographs of fly eyes in which the eye-specific driver *GMR-Gal4* is used to express different constructs. (A) *GFP* is used as a negative control, and shows the stereotypical hexagonal arrangements of the ommatidia. Knock down was performed by expression of RNAi constructs against (B) *CG1868*, (C) *CG8378*, (D) *CG14122* and (E) *CG7759*. Three different magnifications of each eye are shown, with the corresponding scale bars shown at the top.(F) Summary of expression patterns and RNAi phenotypes of the four *Smyd4* class genes.

Early and ubiquitous RNAi expression under *Act5C-Gal4* produced severe reduction in viability (adults eclosed / expected) for two of the genes, *CG1868* (lethal) and *CG14122*, (7.3% survival). For the other two genes there was only reduced viability 67.7% and 82% for *CG7759* and *CG8378* respectively. The retina is part of the peripheral nervous system, and it is a widely used model to study neurodegeneration. Loss of retinal cells affects the external appearance of the eye, producing the so-called rough eye phenotype, in which the units of the compound eye lose their stereotypical shape and organization. In the control eyes (Fig 5A) we can observe the wild type external morphology, with dome-shaped lenses in a hexagonal tiling arrangement, and with inter-ommatidial bristles. The strongest phenotype is obtained by *GMR-Gal4* driven RNAi of *CG1868*, where we can observe several events of ommatidial fusion and the bristles are very often missing or dislodged from their sockets (Fig 5B). The hexagonal tiling is severely disrupted as a result of these fusions and losses of ommatidia. RNAi of *CG8378* results in the weakest phenotype, with a mildly disrupted arrangement compared to the previous one (Fig 5C). RNAi of either *CG14122* or *CG7759* produces a phenotype which is intermediate between the previous two, with disruption of the arrangement, ommatidia missing or partially fused, and also missing or supernumerary bristles (Fig 5D and 5E). It is worth noting that when the external morphology of the eye is affected, even mildly, that is a sign of serious neural defects. In a model of the Charcot-Marie-Tooth neuropathy we have recently developed [48] there is gradual neurodegeneration and still the external aspect of the eye is not affected.

In Fig 5F we present a summary of the patterns of expression and phenotypes of the four *Smyd4* genes. Briefly, they fall into two patterns of expression, and at least one gene within each class is required for full viability. Down-regulation of all four genes in the retina shows that they are required for photoreceptor development or survival to some extent. In summary, none of these genes seems to be functionally redundant.

## Discussion

### Evolution of the *Smyd* gene family

**Fig 6:**
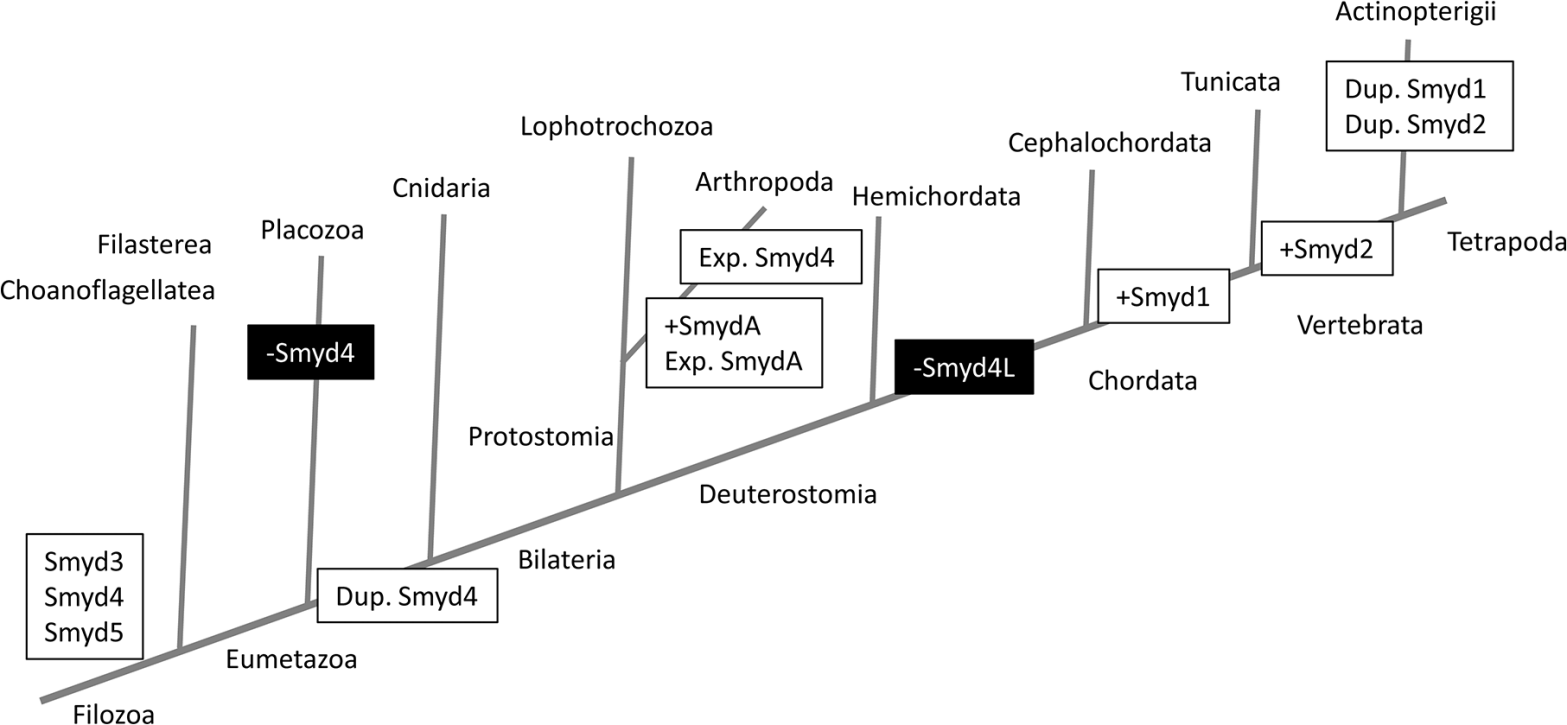
Proposed evolutionary history of *Smyd* genes in metazoans. The schematic tree represents the accepted phylogeny for the phyla/subphyla represented in this work. From an original complement of *Smyd* genes, comprising Smyd3, Smyd4 and Smyd5, we indicate with white boxes the most likely events of gene gain (+) duplication (Dup.) and expansion (Exp.); and in black boxes the events of gene loss (-).

Our results suggest an evolutionary landscape that we illustrate in Fig 6. The grouping in the phylogenetic trees and the domain organizations support the definition of three classes of *Smyd* genes that were already present in the ancestor of metazoans: *Smyd3*, *Smyd4* and *Smyd5*, and at least two of them, *Smyd3* and *Smyd4* were present from earlier eukaryotic evolution since they also have representatives in plants. Re-examination of yeast SET5 and SET6 sequences suggests that *Smyd* genes are present in all eukaryotes.

Within the metazoans, the *Smyd3* and *Smyd5* classes have a relatively simple lineage, since they have a single representative in each species, with the exception of the expansion of *Smyd3* in chordates to give rise to *Smyd1* and *Smyd2*, and the two *Smyd3*-related genes in *B*. *floridae*. Although the evolutionary relationships among chordates are controversial, recent evidence suggests that cephalochordates diverged first [49]. Our results would be consistent with this scenario, with the origin of *Smyd1* in a common ancestor of tunicates and vertebrates, and of *Smyd2* in vertebrates. Both genes would have suffered a second duplication in fish. The *Smyd4* class has a more complex evolutionary history. This class has been lost in placozoa, but has been duplicated in eumetazoa, giving rise to the *Smyd4* and *Smyd4L* groups. In the arthropods there has been an expansion of the *Smyd4L* group and a further duplication and expansion in insects (*Smyd4I*). According to our results, vertebrate *Smyd4* and insect *Smyd4* and *Smyd4I* genes are expressed in nervous system, mesodermal derivatives and digestive tract, while *Smyd4L* is expressed only in mesoderm. This points out to a duplication of *Smyd4* as the origin of *Smyd4I*. Finally, *Smyd4L* was lost in the chordate ancestor, leaving the *Smyd4* group as the only representative of this class.

In addition to the expansion of the *Smyd4* class in insects, arthropod genomes contain a further expanded class of *Smyd* genes, which we have called *SmydA*. These genes do not share domains with other *Smyd* genes outside the SET-MYND core, and also show a high divergence within this core. More research is required to understand why arthropods have suffered such an expansion in the number of *Smyd* genes and what their origin is.

*B*. *floridae* is a species that shows several differences regarding *Smyd* gene complement compared to the related phyla of hemichordata and vertebrata. In addition to the expected *Smyd* genes, it has two more genes within the *Smyd3* class, probably coming from a duplication which is different from the one that gave rise to vertebrate *Smyd1* and *Smyd2* genes. These could be further examples of amphioxus-specific gene duplications already described [50], which are quite common despite their apparently conservative anatomy close to that of the chordate ancestor. In addition, this species also has a more atypical *Smyd* gene, with N-terminal TPR domain and an additional MYND domain in the C-terminal position, which has putative orthologs in mollusk species but not in the rest of metazoan species. Our results present three types of evidence in favor of shared ancestry rather than convergence or independent origin: phylogeny of the SET-MYND core, BLASTP hits in searches against all metazoan sequences, and domain organization. Still, at this point it is impossible to be sure whether these genes represent a case of horizontal gene transfer or differential loss. Horizontal gene transfer is extremely rare in animals, probably due to the fact that the germ line is isolated from the somatic cells, leaving differential gene loss as the most likely explanation. In this respect, we also show that some Smyd-related sequences in *B*. *floridae* and *L*. *gigantea* are not correctly annotated. Obtaining genome sequences of more animal species, and a more rigorous annotation, will facilitate the reconstruction of the evolution of complex gene families.

Some of our findings are supported by an independent study [23]. In a comparison of the genomics of two ant species, *Camponotus floridanus* and *Harpegnathos saltator*, the authors constructed two phylogenies of selected insect and vertebrate species corresponding to what they call *SMYD1-3* and *SMYD4* families. Although this study is not as exhaustive as ours, since their species represent only two phyla, and do not analyze any *Smyd5* sequences it confirms some of our conclusions. For example, their tree shows the same three groups in their *SMYD4* family (our *Smyd4* class), with *CG1868* as the closest homolog of human *SMYD4*. Their *SMYD1-3* family tree includes two groups, one that would correspond to our *Smyd3* class and another one corresponding to the *SmydA* group, which contains 3 genes from each ant species, and several of the insect sequences we have identified.

### Classification of the *Smyd* genes in metazoan species

As a result of our phylogeny and classification of *Smyd* genes, it becomes evident that previous assignments of gene names cannot be supported. The work in ant genomics we mention in the previous section is a first example [23]. The authors describe differences in expression in a gene they call *SMYD3* (*Hsal_14941*, *Cflo_06803*), due to its presence in what they incorrectly identify as a *SMYD1-3* family. In fact, those genes belong to the *SmydA* group, and the true *SMYD3* orthologs would be *Hsal_08938* and *Cflo_10149*, the genes that group with human *SMYD3* and fly *buzidau*. We have also mentioned that a *D*. *melanogaster* gene has been identified as the *SMYD4* ortholog [21], but our work shows that this gene, *CG14122* belongs in the *Smyd4L* group. The real ortholog candidate is *CG1868* on the basis of phylogeny and pattern of expression.

These two examples highlight the pitfalls of attempting to classify genes in the absence of a rigorous phylogeny. Quite often, gene families and subfamilies are defined in function of the representatives found in vertebrate species. It is widely assumed that vertebrates represent the most complex genomes among metazoans, in other words, biomedicine quite often ignores evolution. If we want to take full advantage of the wide range of experimental models that can contribute to research in life and health sciences, we cannot afford to make such mistakes.

### The *Smyd4* class in arthropods

In contrast to classes *Smyd3* and *Smyd5*, the *Smyd4* class has suffered extensive expansion in the arthropod species. Our results seem to reflect that there has been expansion and a faster pace of divergence of arthropod genes within the *Smyd4L* group, so that they form separate subgroup with more than one representative; and a more dramatic event of the same type in the origin of the *Smyd4I* group. If we take into account the expression profiles of these genes in *D*. *melanogaster*, it is more likely that *Smyd4I* originates from *Smyd4*, since they share anatomical, developmental and temporal expression profiles.

But this multiplicity of *Smyd4* genes begs the question of why so many of them. The patterns of expression of the four genes we investigated are highly regulated and not completely overlapping, which in principle rules out that the high copy number is just to ensure a high level of expression. Our knock-down experiments indicate that there is not functional redundancy among all of them, as down-regulation of individual genes always results in some kind of phenotype. Therefore, the most likely explanation is that LSE of the class has adaptive value in the arthropod lineage, and the different copies of *Smyd4* have anatomical or molecular specialization. In a genome-wide analysis in *Caenorhabditis elegans* [51], phenotype masking by duplicate genes was found to be less common than expected, indicating that this duplication quite often had functional significance. All seems to indicate that expansion of the *Smyd4* genes has helped the different arthropod species to adapt their physiology to their environmental, ecological and/or ethological features. This makes sense if, as evidence from other *Smyd* family members seems to indicate, the major role of this type of proteins is epigenetic control of gene expression. Bonasio et al. (2010) found that among the *Smyd* genes in their ant species there were differences in gene expression depending on the caste and developmental stage, pointing to an important role of *Smyd* genes in transcriptional control. All insect species have a similar body plan, so it is to be expected that the genes controlling basic development are conserved in sequence and number. In contrast, they have a wide diversity regarding other biological features: body size, social structure, behavior, life cycle, feeding, ecology, etc. It is possible that *Smyd4* genes are part of the genetic and epigenetic toolkit responsible for this variability.

### *D*. *melanogaster* as a model organism to study the *Smyd* genes

From our results it is evident that in the case of *SMYD4*, the use of *Drosophila* to study gene function is of relatively limited interest. The main advantage of this model organism is the range of techniques in advanced genetics, including transgenics, RNAi, somatic clones and gene editing. The genes of the *Smyd4* class are not redundant on the basis of their patterns of expression and mutant phenotypes. We show that some experimental approaches would be still possible and informative; such as *in vivo* localization of proteins in transgenics. Human SMYD4 protein fused to GFP has a sub-cellular localization pattern in *Drosophila* adult muscle, in sarcomeres and nucleus, that is almost identical to murine Smyd1 [7]. By directed mutagenesis or other genetic alterations, this type of transgenics can be used to investigate the molecular biology of Smyd proteins. In contrast, it would be very difficult to be sure that knock-out of one of the *Smyd4* genes in *Drosophila* would be representative of a clinical phenotype without a previous validation work, dissecting the molecular functions of each one of them. Similarly, it is difficult to know *a priori* the interest of *Drosophila* to investigate *Smyd3* class. It is possible that *SMYD1* and *SMYD2* carry out roles that are specific of vertebrates and cannot be modeled outside this group, and we don´t know if a *Drosophila Smyd3* knock-out would be equivalent to a triple knock out in vertebrates of the whole class.

The situation is more straightforward with *Smyd5*, which seems to be more conserved in copy number and phylogenetic distribution. With some validation work, for example determination of the pattern of expression and mutant phenotype, and rescue of this phenotype with expression of human *SMYD5*, flies could be a good model if this gene proves to be of clinical interest. The biomedical interest of the *SmydA* group is probably null, but from a biological and evolutionary point of view, it would be extremely interesting to know what their relevance is in insect physiology, and whether they had an adaptive role in the diversification of this phylum.

## Conclusions

The *Smyd* gene family is present in all eukaryotes, since the *S*. *cerevisiae* SET5 and SET6 proteins also contain a putative Zn finger similar to the MYND type. Metazoan *Smyd* genes can be grouped in three classes: *Smyd3*, *Smyd4* and *Smyd5*. All three classes were present in metazoan ancestors, and at least *Smyd3* and *Smyd 4* are also found in plants. *Smyd4* is the most variable class in animals, with large variations in copy number. *Drosophila* contains 4 *Smyd4* genes, which are not redundant according to their expression patterns and effects of down-regulation. The phylogeny of *Smyd* genes should be taken into account to obtain information of biomedical interest using model organisms.

## Materials and Methods

The experimental research performed in animals was approved by the Foundation Prince Felipe Research Center Ethics Committee for Animal Experimentation.

### Retrieval of Smyd protein sequences, multiple sequence alignments and phylogenetic-tree construction

To build a database of Smyd proteins, we performed systematic BLASTP searches using as queries the sequences of the five human SMYD proteins. In organisms without a comprehensive protein database, additional TBLASTN searches against the genomic sequence were performed. The sequences were validated by reciprocal BLAST to check if they were most similar to the query sequence. We included in our database only those sequences with high similarity (E value less than 10^−7^) and continuous alignment within the core region containing the SET, MYND and post-SET domains. This search revealed proteins that were clearly homologous to one of the five query sequences, with homology beyond the SET-MYND core, and some other sequences that were only similar in this core, such as an arthropod-specific group. We performed further searches in all species with these novel sequences using the same criteria. Accession numbers of all the sequences are detailed in S1 Table.

The resulting set of sequences was aligned using Clustal Omega (http://www.ebi.ac.uk/Tools/msa/clustalo/). Then, the alignment was edited with Jalview 2 [52] to select the continuous region that comprises SET, MYND and post-SET domains (aminoacids 18 to 279 of the human SMYD1, Uniprot ID Q8NB12 as reference), removing from the alignment the N and C terminus of the sequences. This selected core was used to obtain the phylogenetic trees.

Phylogenetic trees were obtained both by the Neighbor-Joining (NJ) and the Maximum Likelihood (ML) methods, using the routines available in MEGA version 6.0 [53]. Gaps were treated using the partial deletion option (cut-off: 65%). In the case of the specific study of the Smyd4 sequences, the alignment has fewer gaps, so the partial deletion option is not necessary. To build the ML tree all sites were considered, but for the NJ tree this option is not available so we used the pairwise deletion option. For ML analyses, the NJ/BioNJ tree was taken as starting point for the iterative searches using the LG model of amino acidic substitutions (Le and Gascuel, 2008). A discrete Gamma (G) distribution was used to model evolutionary rate differences among sites (5 categories). The rate variation model allowed for some sites to be evolutionarily invariable (I). This LG + G+I model was chosen because it was the best for all datasets, according to the ML model comparison analyses available in MEGA 6. For NJ the evolutionary distances were computed using the substitution model of Jones–Taylor–Thornton (JTT) (Jones et al. 1992). The rate variation among sites was modeled with a gamma distribution (shape parameter = 5). The reliability of phylogenetic reconstructions was estimated by the bootstrap analysis with 500 replicates for all the analysis.

### Sequence searches and domain prediction software

BLASTP and TBLASTN sequence searches were performed at the NCBI server (http://www.ncbi.nlm.nih.gov). For the prediction of PROSITE signatures we used the EXPASY server (http://prosite.expasy.org/); and for identification of PFAM signatures using hidden Markov models we used the HMMER platform (http://hmmer.janelia.org/search/hmmscan). Secondary structure predictions were done by using four different algorithms in the Max Plank bioinformatics server (http://toolkit.tuebingen.mpg.de/quick2_d).

### High-throughput expression data

To compare the expression among the *Drosophila* Smyd4 homologous genes, the FlyAtlas Anatomical Expression Data [54] and the modENCODE Temporal Expression Data [55] were downloaded from Flybase (http://flybase.org/).

The anatomical expression data indicated in the graphics in Fig 3 correspond to the following tissues in this order: larval central nervous system, larval midgut, larval hindgut, larval malpighian tubules, larval fat body, larval salivary gland, larval trachea, larval carcass, adult head, adult eye, adult brain, adult thoracic-abdominal ganglion, adult crop, adult midgut, adult hindgut, adult malpighian tubules, adult fat body, adult salivary gland, adult heart, adult virgin female spermatheca, adult inseminated female spermatheca, adult ovary, adult testis, adult male accessory gland, adult carcass.

The temporal expression data correspond to the following time points: embryo 0-2h, embryo 2-4h, embryo 4-6h, embryo 6-8h, embryo 8-10h, embryo 10-12h, embryo 12-14h, embryo 14-16h, embryo 16-18h, embryo 18-20h, embryo 20-22h, embryo 22-24h, larva L1, larva L2, larva L3 12 h old, larva L3 puffstage 1–2, larva L3 puffstage 3–6, larva L3 puffstage 7–9, white prepupae new, white prepupae 12hr, white prepupae (WPP) 24hr, pupae 2 day post-WPP, pupae 3 day post-WPP, pupae 4 day post-WPP, adult male 1 day, adult male 5 day, adult male 30 day, adult female 1 day, adult female 5 day, adult female 30 day.

### Fly stocks and RNAi

Fly stocks were maintained at 25°C on standard corn flour medium. We obtained transgenic lines with inducible RNAi constructs from the Vienna *Drosophila* Resource Center (http://stockcenter.vdrc.at/; [56]) for the following genes: *CG14122* (stocks 51782 and 51783), *CG7759* (stocks 100412 and 21052), *CG8378* (stocks 40705 and 40706) and *CG1868* (stocks 106709 and 25378); from this repository we also obtained *UAS-Dcr2* to co-express the Dicer2 nuclease in order to increase interference. The *GMR-Gal4*, *Mhc-Gal4*, *elav-Gal4* and *Act5-Gal4* drivers were obtained from the Bloomington *Drosophila* Stock Center (http://flystocks.bio.indiana.edu/).

For viability studies, we scored the offspring from two independent crosses of *Act5-Gal4 / Cyo; UAS-Dcr2* females and males of the homozygous RNAi stocks for *CG14122* (stock 51783), *CG7759* (stock 100412), *CG8378* (stock 40706) and *CG1868* (stock 106709). All the progeny carries the UAS-RNAi construct, and the numbers of flies carrying the *Act5-Gal4* driver (knock-down) were compared to the number of siblings carrying the *CyO* chromosome (control), expecting a 1:1 segregation.

### Transgenics

The human SMYD4 ORF was amplified by PCR from cDNA obtained from the human schwannoma cell line HEI-193 (ATCC no. PTA-4544). The primers containing restriction site adapters were:

SMYD4_pEGFP_XhoI_F ggaaCTCGAGctATGGATCTGCCTGTGGATGAATG

SMYD4_pEGFP_BamHI_R gcgcGGATCCAATGCAGGCCCTACAGGG)

The PCR product was digested and cloned between the *XhoI* and *BamHI* sites of the pEGFP-N1 plasmid. The pEGFP-SMYD4 plasmid was transformed into a *dam* ^*–*^ */dcm* ^*−*^ *E*. *coli* strain to avoid methylation of the *XbaI* site in the pEGFP-N1 plasmid. A SMYD4-GFP fragment was excised by digestion with *XhoI* and *XbaI* and cloned into the pUASt plasmid. The sequence of the construct was confirmed by sequencing on an ABI Prism 3130xl Genetic Analyser (Applied Biosystems). Transgenics were made by random integration method. Injections were performed by BestGene Inc. (Chino Hills, CA), and the selection of the transformants was done by us.

### *In situ* hybridization

To construct the plasmids used to synthesize the antisense RNA probes for in situ hybridization to *Drosophila* and mouse embryos, exon-containing gene regions were amplified using specific primers in polymerase chain reaction. As a template for amplification, we used genomic DNA of the *y w* strain for the *Drosophila* probes, and adult mouse brain cDNA for the mouse probe. The primer sequences were the following:

CG14122-F TTGCGTGGACGTGCGTGATGC

CG14122-R TGTCCTTGTGAGGCAGTGCAGC

CG1868-F AACCTTCAAGTCATTGGCATCC

CG1868-R TGCTCGCTAGCCAGATAATCC

CG7759-F TGCAAGAGCGATGAGGAGCG

CG7759-R AATCCTTGAGATGCTGTTGGC

CG8378-F TGCAGGACTGGAAACTAATCG

CG8378-R ATAGTCGTCACCGTATTGCG

Smyd4-F AGTGCCTGAAGCTCTGAGTGCC

Smyd4-R TGCCTGCAGATCAGTGACAACC

The PCR products were cloned into the pCR2.1-TOPO following the manufacturer’s protocol (Invitrogen). The vectors were linearized and used to generate a digoxigenin-labelled RNA anti-sense probe using the DIG RNA labeling kit (Roche) according to the manufacturer's directions. In situ hybridization was performed in whole mount *Drosophila* embryos of the *Oregon-R* strain, and in 15 micron cryostat sections of E14.5 mouse embryos of the *C57BL/6J* genotype.

### Immunofluorescence

Heads and thoraxes from adult flies were dissected and fixed in 4% paraformaldehyde in phosphate buffer saline (pH7.2) overnight at 4°C, followed by cryoprotection with 30% sucrose for 48 h at 4°C (heads were first pre-incubated with 10% sucrose for 2 h). Then they were embedded in OCT compound and cut in the cryostat (16 μm for thoraxes and 14 μm for heads). Cryosections were washed in PBT, blocked (PBS containing 5% BSA and 0.3% Triton X-100) for 45 minutes at room temperature and incubated with anti-GFP (Rockland) overnight at 4°C. After washes with PBT, the tissue was incubated with secondary antibodies. After washes with PBT, cryosections, the tissues were incubated with phalloidin-rhodamine diluted in PBT and then with DAPI. Sections were mounted with Aqua Poly/mount (Polysciences, Inc., Warrington, PA). Images were obtained with a Leica SP8 microscope.

### Scanning electron microscopy

Scanning electron microscopy analysis of adult eyes was performed following the critical point drying method [57]. Briefly, adult flies were fixed (4% paraformaldehyde, 8% glutaraldehyde, 0.2% Triton X-100, PBS) for 3h. The fixative solution was removed and the flies were rinsed with water and dehydrated in ethanol (once in 25%, 50%, 75%, and twice in 100%) 12 h each. Flies were dried by using critical point drying, coated with palladium/gold and then analyzed by using a Scanning Electron Microscope (Philips XL-30 ESEM).

## Supporting Information

S1 Fig Phylogenetic trees of metazoan *Smyd* genes. Maximum likelihood and neighbor joining trees were constructed from the Clustal Omega alignment. The main branches are colored according to the classes shown in Fig 1.
(TIF)

S2 Fig Phylogenetic trees of Smyd4 genes from metazoans. These trees support the three main groups in the *Smyd4* class: *Smyd4*, *Smyd4L* and *Smyd4I*.
(TIF)

S3 Fig Phylogenetic tress of Smyd genes from the extended data set. In addition to the sequences in S1 Fig, these trees contain sequences from unicellular basal species, *A*. *thaliana* and *S*. *cerevisiae* (highlighted in bold type).
(TIF)

S4 Fig Secondary structure prediction of the MYND Zn fingers. Secondary structures are coded blue E for β-sheet and red H for α-helix. The secondary structure for SMYD1 determined by X-ray crystallography is indicated at the top. For the putative Zn finger regions from the sequences in Fig 3, four different prediction algorithms were used.
(TIF)

S5 Fig Annotation of atypical *Smyd* genes in *L*. *gigantea* and *B*. *floridae*. TBLASTN searches were performed against the genomic contigs using as queries the protein sequences of *L*. *gigantea* 232186 and *B*. *floridae* 74594. The first one has high homology hits to its corresponding locus, to a downstream locus that seems to be a duplication comprising the last exon, and to a second locus in a different genomic scaffold which has not been annotated. The second one also has hits corresponding to its own locus and an additional one in a different scaffold. In both cases the second copy has the same putative intron/exon structure as the annotated gene. The images are screen captures of the results obtained in the NCBI server.
(TIF)

S6 Fig Analysis of the MYND Zn fingers from the *Drosophila* Smyd proteins. At the top we show the sequence of the Zn fingers of all the proteins within each of the four classes. Two of the SmydA proteins have a second MYND Zn finger outside the Smyd core. At the bottom, we compare these sequences with the PROSITE consensus for the MYND domain, and shade those regions that depart from this consensus.
(TIF)

S7 Fig Sub-cellular localization of human SMYD4 fused to GFP in *Drosophila* tissues. The fusion protein was expressed in neurons under the control of *elav-Gal4* (A, A’) and in muscle under the control of *Mhc-Gal4* (B-B”). In neurons, SMYD4-GFP is predominantly cytoplasmic (A), as revealed by lack of co-localisation with the nuclear stain DAPI (B). In muscle, SMYD4 co-localizes with the myofibrils (B, B’) and is also present in the nuceus (B, B”). Within the sarcomere, it is more abundant in the M lines (arrow) and weaker in the Z lines (arrowhead).
(TIF)

S1 File Clustal Omega alignment of the metazoan SMYD proteins. The FASTA alignment was formatted in the mview server (http://www.ebi.ac.uk/Tools/msa/mview/) and downloaded as an html file.
(HTM)

S2 File Clustal Omega alignment of metazoan SMYD4 proteins. Formatted in mview.
(HTM)

S3 File Clustal Omega alignment of all SMYD proteins. Formatted in mview.
(HTM)

S1 Table Accession numbers of the protein sequences. (DOCX)
