# Supplementary figures and images for: Evolutionary History of the Smyd Gene Family in Metazoans: A Framework to Identify the Orthologs of Human Smyd Genes in Drosophila and Other Animal Species
Source: PLoS One. 2015 Jul 31;10(7):e0134106. doi: 10.1371/journal.pone.0134106 (PMC4521844; doi:10.1371/journal.pone.0134106)

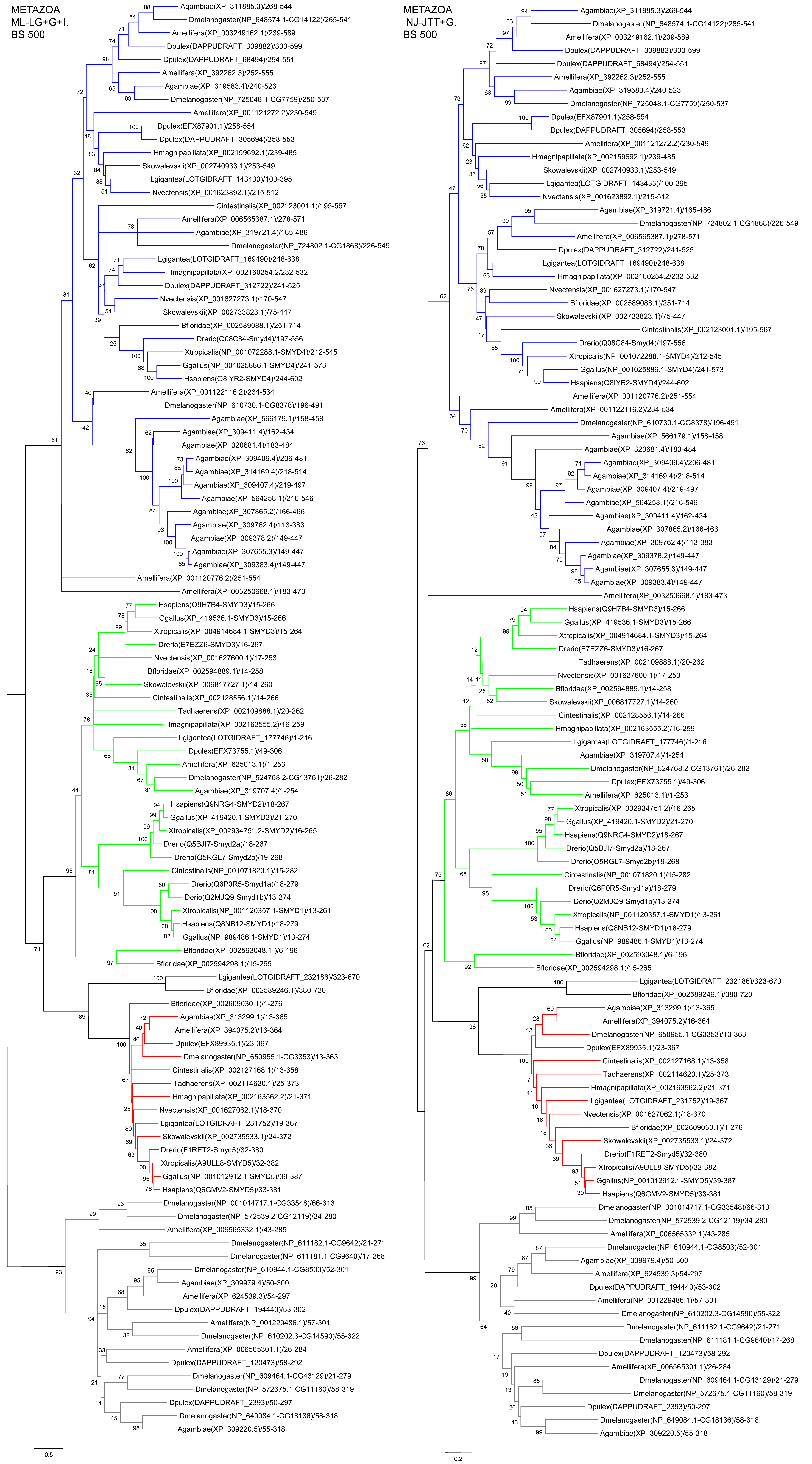

Supplement: S1 Fig — Maximum likelihood and neighbor joining trees were constructed from the Clustal Omega alignment. The main branches are colored according to the classes shown in Fig 1. (TIF) [file pone.0134106.s001.tif]

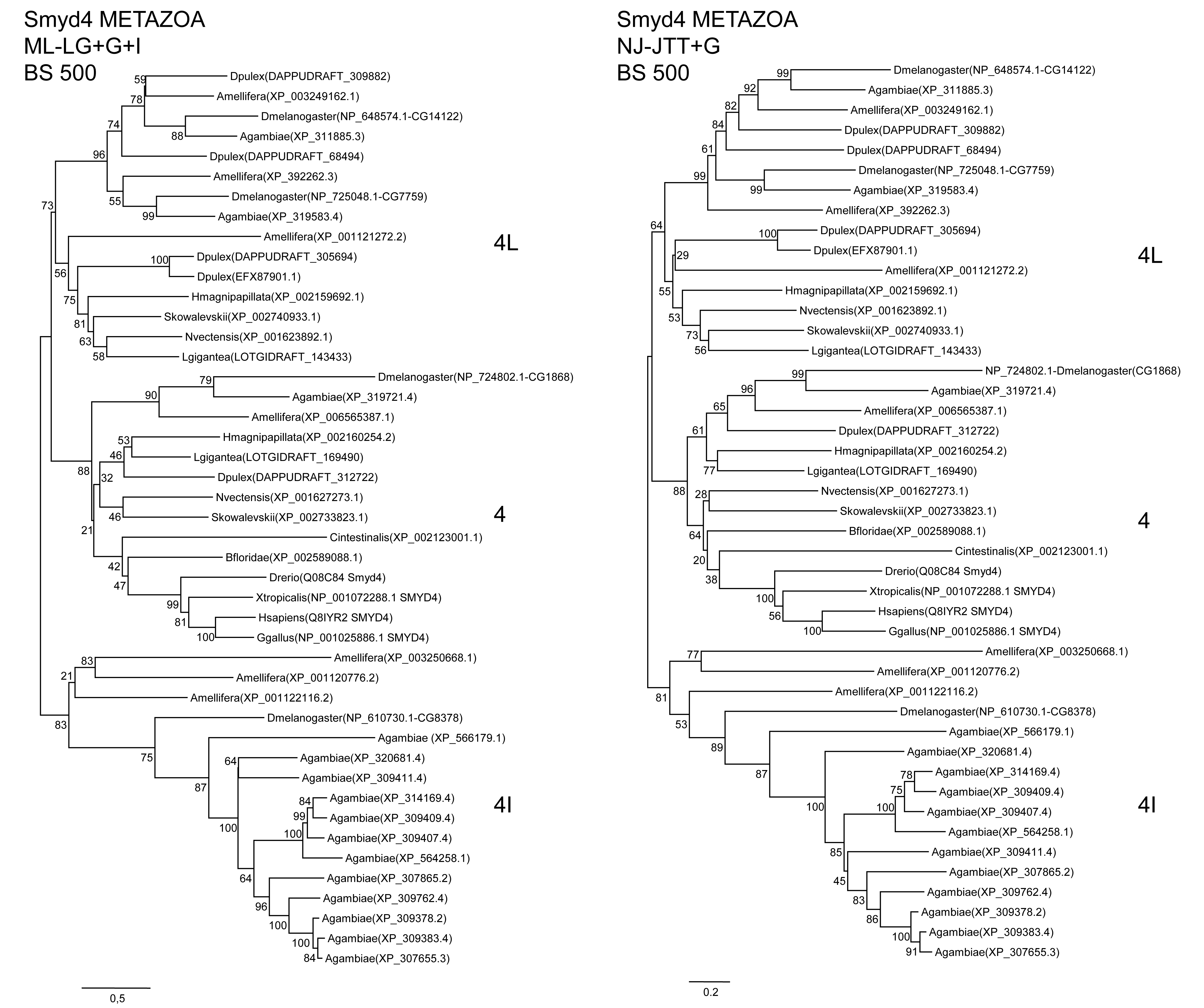

Supplement: S2 Fig — These trees support the three main groups in the Smyd4 class: Smyd4, Smyd4L and Smyd4I. (TIF) [file pone.0134106.s002.tif]

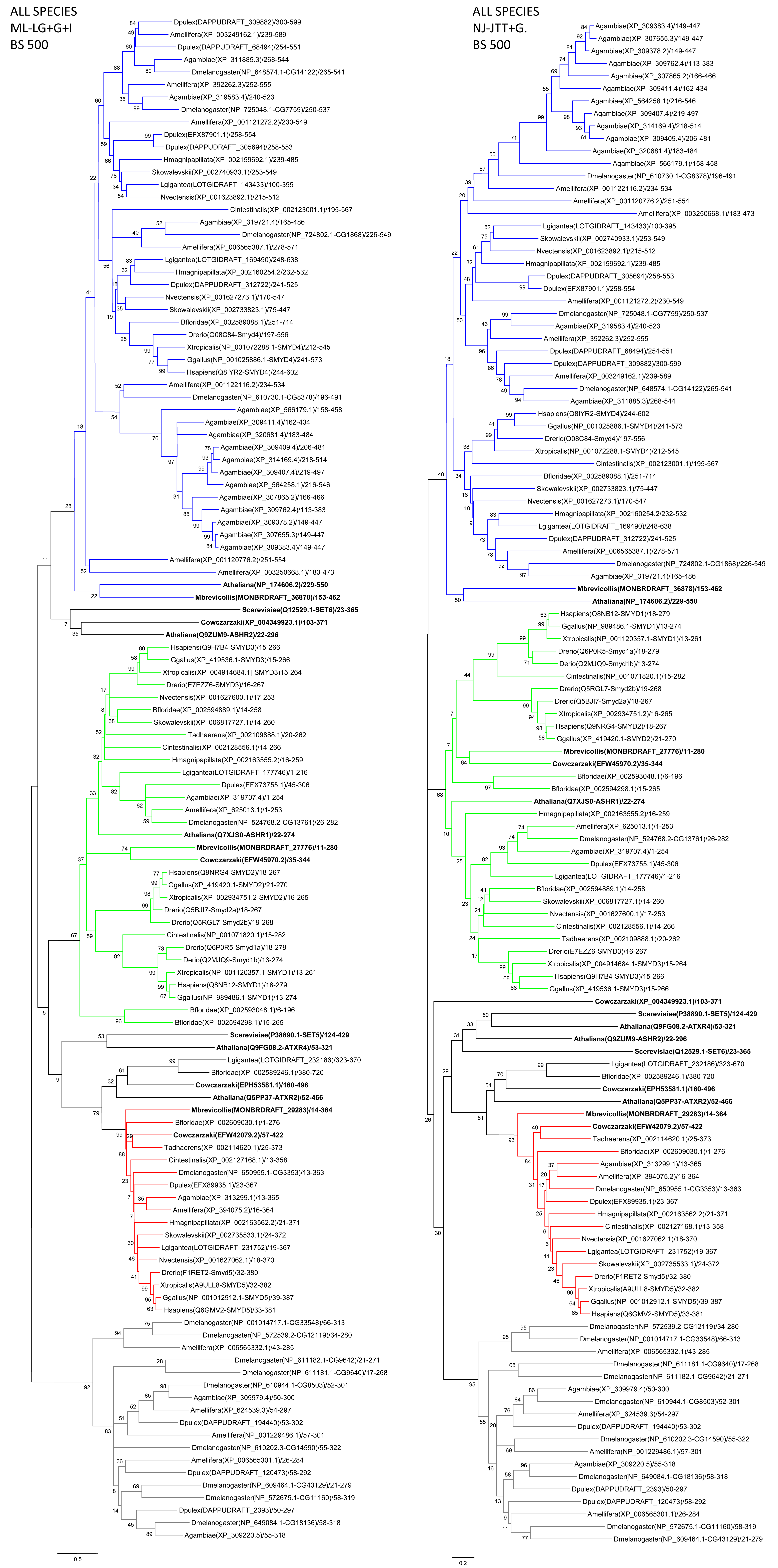

Supplement: S3 Fig — In addition to the sequences in S1 Fig, these trees contain sequences from unicellular basal species, A. thaliana and S. cerevisiae (highlighted in bold type). (TIF) [file pone.0134106.s003.tif]

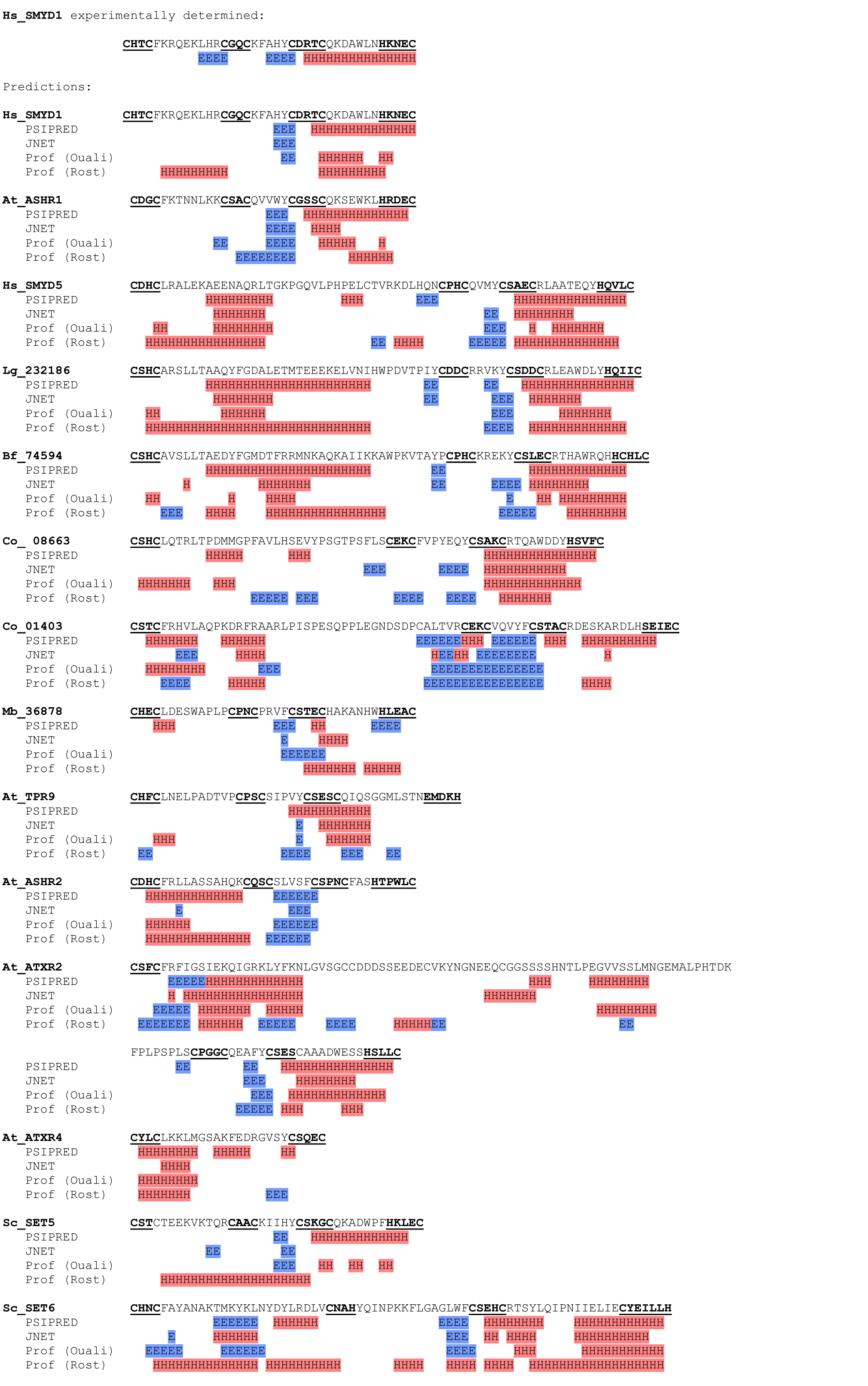

Supplement: S4 Fig — Secondary structures are coded blue E for β-sheet and red H for α-helix. The secondary structure for SMYD1 determined by X-ray crystallography is indicated at the top. For the putative Zn finger regions from the sequences in Fig 3, four different prediction algorithms were used. (TIF) [file pone.0134106.s004.tif]

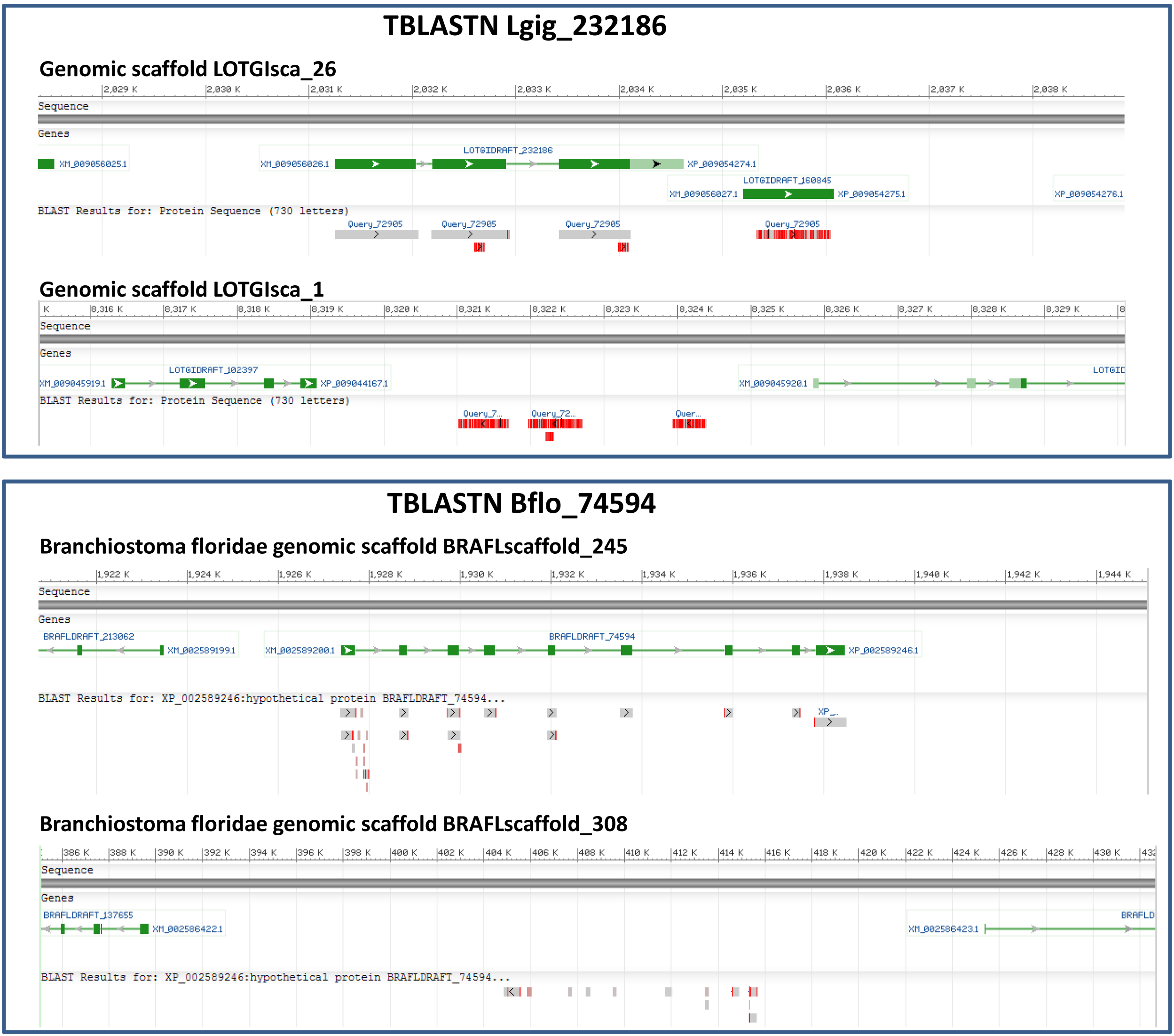

Supplement: S5 Fig — TBLASTN searches were performed against the genomic contigs using as queries the protein sequences of L. gigantea 232186 and B. floridae 74594. The first one has high homology hits to its corresponding locus, to a downstream locus that seems to be a duplication comprising the last exon, and to a second locus in a different genomic scaffold which has not been annotated. The second one also has hits corresponding to its own locus and an additional one in a different scaffold. In both cases the second copy has the same putative intron/exon structure as the annotated gene. The images are screen captures of the results obtained in the NCBI server. (TIF) [file pone.0134106.s005.tif]

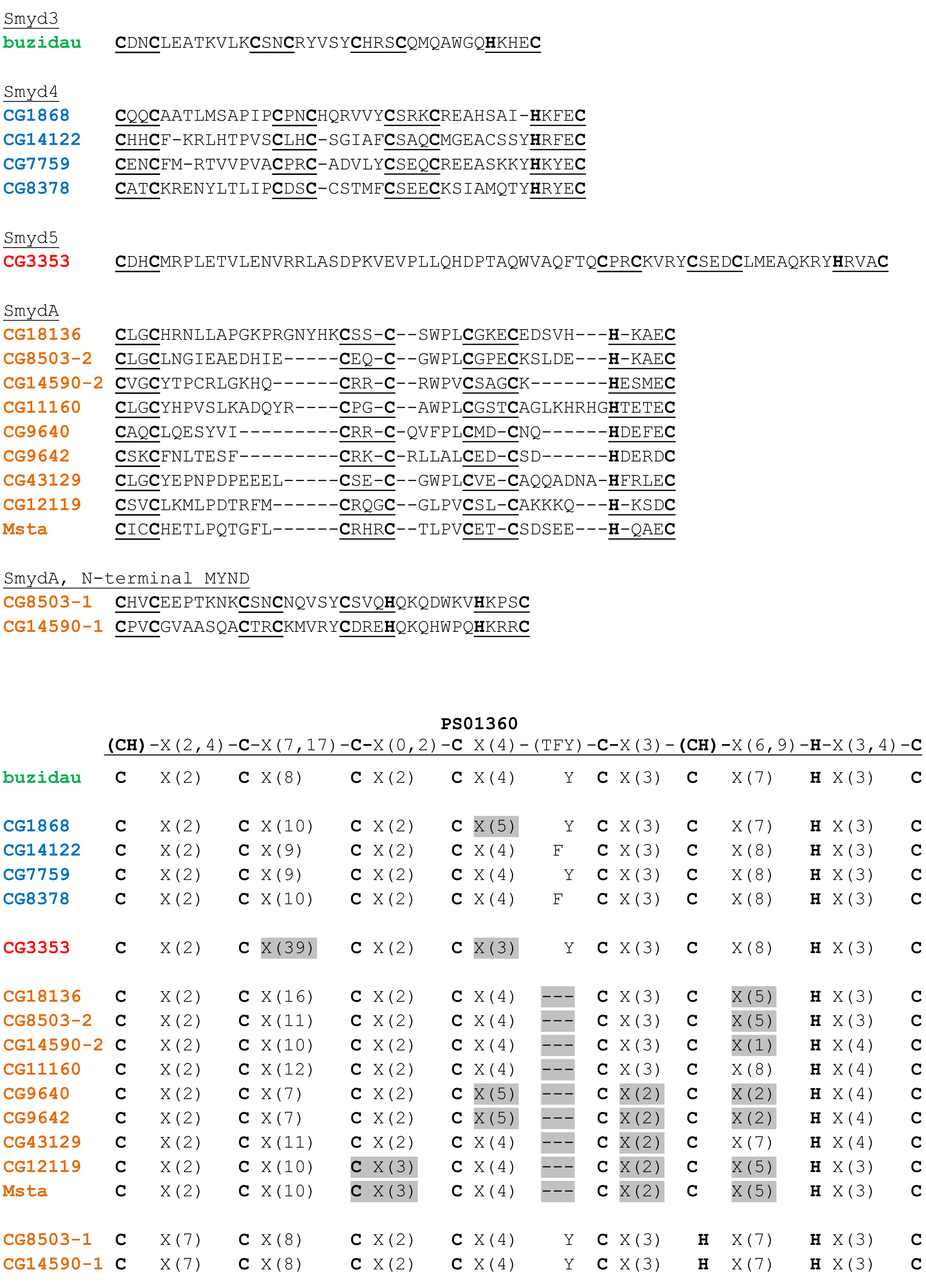

Supplement: S6 Fig — At the top we show the sequence of the Zn fingers of all the proteins within each of the four classes. Two of the SmydA proteins have a second MYND Zn finger outside the Smyd core. At the bottom, we compare these sequences with the PROSITE consensus for the MYND domain, and shade those regions that depart from this consensus. (TIF) [file pone.0134106.s006.tif]

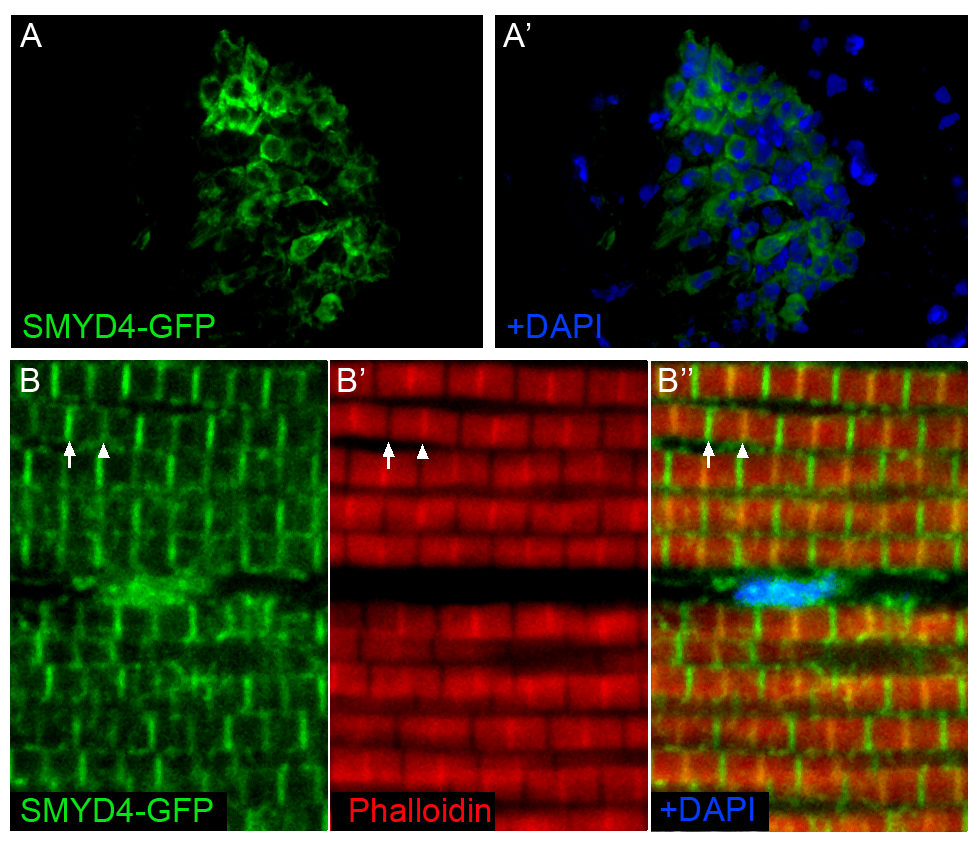

Supplement: S7 Fig — The fusion protein was expressed in neurons under the control of elav-Gal4 (A, A’) and in muscle under the control of Mhc-Gal4 (B-B”). In neurons, SMYD4-GFP is predominantly cytoplasmic (A), as revealed by lack of co-localisation with the nuclear stain DAPI (B). In muscle, SMYD4 co-localizes with the myofibrils (B, B’) and is also present in the nuceus (B, B”). Within the sarcomere, it is more abundant in the M lines (arrow) and weaker in the Z lines (arrowhead). (TIF) [file pone.0134106.s007.tif]
