# Supplementary material for: Evolutionary History of the Smyd Gene Family in Metazoans: A Framework to Identify the Orthologs of Human Smyd Genes in Drosophila and Other Animal Species
Source: PLoS One. 2015 Jul 31;10(7):e0134106. doi: 10.1371/journal.pone.0134106 (PMC4521844; doi:10.1371/journal.pone.0134106)
Supplement: S1 File — The FASTA alignment was formatted in the mview server (http://www.ebi.ac.uk/Tools/msa/mview/) and downloaded as an html file. (HTM) [file pone.0134106.s008.htm]

```
Reference sequence (1): Tadhaerens(XP_002114620.1)/25-373
Identities normalised by aligned length.
Colored by: identity + property
```

|  |
| --- |
| ```                                                                      1 [        .         .         .         .         :         .         .         . 80    1 Tadhaerens(XP_002114620.1)/25-373                       100.0%     GKGLF----ATNCFNEGDEIFKE-NPLVCAQFL---WNEFYK--------------------YEACEYCLRSLEDAETMA       2 Bfloridae(XP_002609030.1-BRAFLDRAFT_84846)/1-276         31.0%     ------------------------------MFR---SPYVPK--------------------SPSCDHCMRSMEPAEAMS       3 cintestinalis(XP_002127168.1)/13-358                     39.9%     GFGLF----STEDISSDSVILEE-DPIISCQFS---WNKLYK--------------------YRACDYCMKSLETTEEMC       4 Dpulex(EFX89935.1)/23-367                                41.0%     GRGLF----TTRSFKNGETIIEE-QPLFSCQFS---WNYAYG--------------------YSACDFCMRPLETAEENA       5 Dmelanogaster(CG3353-NP_650955.1)/13-363                 39.1%     GRAMI----ATKNFAKDEVIFEE-EPFVSRQFS---WNVAYG--------------------YAACDHCMRPLETVLENV       6 Hmagnipapillata(XP_002163562.2)/21-371                   44.2%     GKGLF----AASAIKKGDTILTE-KPLVLCQFS---WNRQYN--------------------YVACDYCMRSLETAQNMA       7 Agambiae(XP_313299.1-AGAP003552-PA)/13-365               41.4%     GRGLY----AAELIPEGGTIFEE-QPLVSCQYS---WNAAYG--------------------YLACEYCLRPLETAERNA       8 Amellifera(XP_394075.2-SMYD5-like-Prediction)/16-364     45.0%     GKGLF----AIRSFKDGDTILEE-KPIICSQFA---WNLDYG--------------------YLACDNCLTPLETAEENV       9 Nvectensis(XP_001627062.1)/18-370                        48.0%     GRALF----ASRDFKEGDTIFEE-DPLVCSQFL---WNAAYS--------------------YTACDHCMRSLETAQDMA      10 Skowalevskii(XP_002735533.1)/24-372                      50.1%     GKGVF----AKQRFRKNDVIFRE-KPIVCAQFL---WNEYYK--------------------YSACDHCMKSLETAEEMA      11 Lgigantea(LOTGIDRAFT_231752)/19-367                      51.0%     GRGLF----ARQEIKEGEAILDE-KPLVSTQFL---WNELYK--------------------YTACEYCLRSLETAEAMA      12 Drerio(F1RET2-Smyd5)/32-380                              49.9%     GKGLF----AKKPFKKGDTIFIE-RPLVSSQFL---WNALYK--------------------YRACEYCLRALETAEENA      13 Ggallus(NP_001012912.1-SMYD5)/39-387                     50.1%     GKGLF----ATRSIRKGEAVFVE-KPVVSSQFL---WNALYN--------------------YRACDHCLRALETAEENA      14 Hsapiens(Q6GMV2-SMYD5)/33-381                            49.9%     GKGLF----ATQLIRKGETIFVE-RPLVAAQFL---WNALYR--------------------YRACDHCLRALEKAEENA      15 Xtropicalis(A9ULL8-SMyd5)/32-382                         51.0%     GKGLF----ATRAIRKGETIFQE-KPLVSSQFQ---WNALYR--------------------YRACDHCLRSLETAEENA      16 Lgigantea(LOTGIDRAFT_232186)/323-670                     20.5%     GRGVY----ATEDIKEGDIAFVD-SPVVRAMIS----NPEHK--------------------IEACSHCARSLLTAAQYF      17 Bfloridae(XP_002589246.1-BRAFLDRAFT_74594)/380-720       22.0%     GRAVF----CTEDVAEGQELFRD-TPLVSSQTD----DSAKA--------------------HPACSHCAVSLLTAEDYF      18 Amellifera(XP_006565332.1)/43-285                        14.2%     GRGMF----ATRDIKQNELIFID-APLIVGPKCL-------------------------NKQTKMCICCYKNE-C-----      19 Dmelanogaster(msta-CG33548)/66-313                       14.8%     GRGVF----ATRDIAAGELIFQE-RALVTGPTAR-------------------------KGQLSSCICCHETLPQ-----      20 Dmelanogaster(CG12119)/34-280                            15.8%     GRGVV----ATRSLKRGEIIFRD-SPLLIGLAAH------------------------EEDSLNACSVCLKMLPD-----      21 Amellifera(XP_006565301.1)/26-284                        15.8%     GRYLQ----ASKDLRAGEVILRE-DPVAVGPMS--------------------------CVKDPICFECLSILPN-----      22 Dmelanogaster(CG9642)/21-271                             13.6%     GRFAV----ALCNVRAGETLLLE-NPIVVLPLM--------------------------G--ERRCSKCFNLT-------      23 Dmelanogaster(CG9640)/17-268                             12.9%     GRHLV----ASIAIEPGDTILEE-RPLLVAPHW--------------------------ECHQLKCAQCLQES-------      24 Amellifera(NP_001229486.1-LOC724300)/57-301              12.7%     GRHLL----ASRDLNPGDVILSE-SPLVWGPSI--------------------------HSDQRLCVGCGKQCKS-----      25 Dmelanogaster(CG14590-NP_610202.3)/55-322                14.9%     GRYLK----VTQNIAAGQIVFIE-EPLVVGPKWY-LSDA------------------DKEASNVPCVGCYTPCRL-----      26 Dmelanogaster(CG43129)/21-279                            11.6%     GRYLV----AKGAIRGHGLLIEE-LPFAVGPKC---------------------------NGPVVCLGCYEPNPD-----      27 Dmelanogaster(G11160)/58-319                             13.9%     GRYLV----ANRQLEAGETLIRE-EPLAIGPCV---------------------------SGDPVCLGCYHPVSL-----      28 Amellifera(XP_624539.3-msta-like-Predicted)/54-297       12.8%     GRHYI----ATRNIKVGEIILRDDQPLITGLMY---------------------------NTVPVCLQCYTVLNQ-----      29 Dmelanogaster(CG8503-NP_610944.1)/52-301                 13.4%     GRHLV----ATRTIKPYEIVLKE-APLVRGPAQ---------------------------ISAPVCLGCLNGIEA-----      30 Agambiae(XP_309979.4-AGAP011530-PA)/50-300               14.8%     GRFLV----ATRDIKAGEIVLKE-SPLVHGPAQ---------------------------ITGPVCVGCLQGLEE-----      31 Dpulex(DAPPUDRAFT_120473)/58-292                         14.4%     GRCIF----ASKNLKPGEIIFGE-TAVITGPKQ---------------------------GCTPCCLKCYASLDR-----      32 Dpulex(DAPPUDRAFT_194440-Predicted)/53-302               11.7%     GRHLI----ACRDLKAGDVILQE-KPIVMGPKH---------------------------TAGQICLGCYSGVDG-----      33 Dpulex(DAPPUDRAFT_2393)/50-297                           16.0%     GRYLV----ASRLIKAGEVILQE-LPLVVGPKL---------------------------NTLPLCLGCYKSITD-----      34 Dmelanogaster(CG18136-NP_649084.1)/58-318                15.9%     GRHLR----ATRDIKIGEQILKE-APLVLGPKV---------------------------ASAPLCLGCHRNLLA-----      35 Agambiae(XP_309220.5-AGAP001025-PA)/55-318               16.2%     GRHLV----ATRHIKQGEIIYRD-EPYAVGPKI---------------------------ANVPLCLGCNRNLMA-----      36 Dmelanogaster(CG1868-NP_724802.1)/226-549                13.1%     GRYMV----AKEAISKGNVIFSE-RASCFVPLEQ----------------------------LLICQQCAATLMS-----      37 Agambiae(XP_319721.4-AGAP008973-PA)/165-486              13.3%     GRYVV----AAEAIKANDTVARE-TAVSFVPVYD-P-----ES--------------SSTLPSFDCQKCAK-VNV-----      38 Bfloridae(XP_002593048.1-BRAFLDRAFT_74375)/6-196         11.1%     ---------GVAAGPDGQLVVVD-R-------------------------------------NERTVTIFPRPE------      39 Bfloridae(XP_002594298.1-BRAFLDRAFT_117670)/15-265       13.8%     GRGIRCNKKGSSGIEPGTLIVKE-EPYSYTLTDG-E-----LL-------------------RTRCHYCLKRLE------      40 Cintestinalis(XP_002123001.1)/195-567                    11.0%     GRHYF----TTFNTETNECLLEE-VAYLGVLNPE-F-----F--------------------STHCSYCLTPCKS-----      41 Drerio(Q08C84-Smyd4)/197-556                             11.1%     GRHML----VMENKPAGEVVLED-EAYCSVLIPA-N-----IFNT----G-TNKAVETFGTEDRHCHHCLSQSL------      42 Xtropicalis(NP_001072288.1-SMYD4)/212-545                12.2%     GRHLL----ASQNIEQGEVLIWE-EAFASVIIPE-R-----K-----QWRKEIKWDTRITACDHYCHYCLNRVI------      43 Hsapiens(Q8IYR2-SMYD4)/244-602                           11.9%     GRCLV----ATKDILPGELLVQE-DAFVSVLNPG-E-----LPP--PHHGLDSKWDTRVTNGDLYCHRCLKHTL------      44 Ggallus(NP_001025886.1-SMYD4)/241-573                    11.1%     GRHLV----ASQDILPGQNLLKE-KAFVSVLCPG-E-----GDSLLLQDSSETVWDTRVTNADLYCHHCLKQLL------      45 Hmagnipapillata(XP_002160254.2/232-532                   13.1%     GRYIF----AKEDIPNGSIIISE-KPYAAVLLPH-W-----Y--------------------KTHCQLCFDKVV------      46 Dpulex(DAPPUDRAFT_312722-Pedicted)/241-525               14.2%     GRYVV----ANRDIKAGETLFVE-QPNALVVLPD-F-----Q--------------------TSRCHHCTRHSSA-----      47 Amellifera(XP_006565387.1-SMYD4-like-Predicted)/278-571  16.8%     GRHVI----ANKFIKEGDILFLE-EPISFVLLNH-D-----T--------------------YSYCQYCNNLNTD-----      48 Bfloridae(XP_002589088.1-BRAFLDRAFT_75068)/251-714       10.4%     GRMLV----AQKAFEPGSVLIVE-QPYAAVLLQK-H-----H--------------------STHCHTCVTPVL------      49 Lgigantea(LOTGIDRAFT_169490)/248-638                     11.5%     GRYLT----TNREIEVGDTLIVE-KPFSSVLLPD-H-----Y--------------------KTHCHHCYHKLPL-----      50 Skowalevskii(XP_002733823.1)/75-447                      10.9%     GRYIL----ATETICRGEIIIKE-KPYGCVLLPS-H-----Y--------------------NTRCYHCVRKTV------      51 Nvectensis(XP_001627273.1)/170-547                       12.5%     GRFLQ----ASSEIRAGDTLIAE-EPYSAVLLPE-N-----A--------------------KTHCECCYKSLV------      52 Amellifera(XP_003250668.1-SMYD4-like-Predicted)/183-473  12.1%     GRYFV----AVKPIKMKDVILID-KSQITHLHKD-DWDDDPT--------------------SNMCHYCFK-YCR-----      53 Amellifera(XP_001121272.2-SMYD4-like-Predicted)/230-549  13.9%     GRHLI----ATKNIKAGSVLIVE-TPFAFSTNKE-A-----L--------------------GRNCLHCHITLMSSN---      54 Amellifera(XP_003249162.1-SMYD4-like-Predicted)/239-589  11.5%     GKRVI----AAKNIEPGNRLIIE-SPHAAILLPE-F-----F--------------------GTHCQHCFS-RFK-----      55 Dmelanogaster(CG14122-NP_648574.1)/265-541               14.2%     GRFVV----ANEGLRTGDVLLFE-EPVAACLEPS-Y-----F--------------------GTHCHHCFK-RLH-----      56 Agambiae(XP_311885.3-AGAP002999-PA)/268-544              13.9%     GRYVV----AAADLGPGEVILTE-PAYAACLHAK-Y-----Y--------------------GTHCSACFS-RLI-----      57 Amellifera(XP_392262.3-SMYD4-like-Predicted)/252-555     15.0%     GRHAI----ATKDIEPGEILAIE-KPYSAFLLAE-Y-----R--------------------LINCFYCFT-KIF-V---      58 Dmelanogaster(CG7759-NP_725048.1)/250-537                15.6%     GRFAR----ASADVKPGEELLVE-RPFVSVLLEK-F-----A--------------------KTHCENCFM-RTV-V---      59 Agambiae(XP_319583.4-AGAP008839-PA)/240-523              12.3%     GRFAR----TNTDLKPNTILLLE-RPHVSVLLED-Y-----S--------------------LDHCTHCFK-RVS-V---      60 Dpulex(DAPPUDRAFT_68494-Predicted)/254-551               14.7%     GRYGV----AASPIRVGDVIAVD-APYASVMNPE-K-----F--------------------STHCHHCYQ-ILE-L---      61 Dpulex(DAPPUDRAFT_309882)/300-599                        12.5%     GRYYV----AADDIKPGQTLVCE-KPYAACLLPG-K-----F--------------------TSHCHHCFV-RL------      62 Dmelanogaster(CG8378-NP_610730.1)/196-491                13.8%     GRFVV----TNRDLAVGDLVSVE-EPFCSTLLTP-M-----R--------------------YIRCATCKRENYL-----      63 Agambiae(XP_566179.1-AGAP000216-PA)/158-458              13.6%     GRYLQ----TNKALKVGDVVMID-EPYVSVLEPE-F-----C--------------------YARCDHCQRPAPF-----      64 Agambiae(XP_564258.1-AGAP011234-PA)/216-546              16.4%     GRHLV----TTQHLKAGDVLMIE-KPYASLLCER-D-----Q--------------------YKRCAFCHNEDTF-----      65 Agambiae(XP_309407.4-AGAP011238-PA)/219-497              15.4%     GRHLV----TTQHLKAGDVLLIE-KPYANLLIDV-E-----R--------------------HVRCAFCQNEDRF-----      66 Agambiae(XP_314169.4-AGAP005253-PB)/218-514              16.2%     GRHLV----TTQHLKAGDVLLIE-KPYASMLNDK-E-----R--------------------YKRCAFCHNEDTF-----      67 Agambiae(XP_309409.4-AGAP011237-PA)/206-481              15.2%     GRHLV----TTQKLKVGDVLLIE-KPYASMLNDQ-E-----R--------------------YKRCDFCQNEDRF-----      68 Agambiae(XP_307865.2-AGAP009448-PA)/166-466              15.6%     GRHVV----TTRRLKVGDVVMLD-TPFVKTLHDP-L-----R--------------------HVRCDFCHAERPF-----      69 Agambiae(XP_309762.4-AGAP010931-PA)/113-383              14.4%     GRHVV----TKRKLKVGDVVMIE-KPFVTVAKET-F-----Q--------------------YIRCDFCQAKRLF-----      70 Agambiae(XP_309378.2-AGAP011267-PA)/149-447              16.9%     GRHVV----TTRKLKVGDVVMIE-RPFVTVLRDS-L-----R--------------------YVRCDFCHEERPF-----      71 Agambiae(XP_309383.4-AGAP011257-PA)/149-447              16.4%     GRHVV----TTRKLKVGDVVMIE-RPFVTVLKDS-F-----R--------------------YVRCDFCHGERPF-----      72 Agambiae(XP_307655.3-AGAP012638-PA)/149-447              16.4%     GRHVV----TTRKLKVGDVVMIE-RPFVTVLKDS-F-----R--------------------YVRCDFCHGERPF-----      73 Agambiae(XP_320681.4-AGAP011835-PA)/183-484              14.6%     GRYVA----TNRNLEAGDVVIIE-QPFSRLLRDI-Y-----R--------------------HVRCDFCHRESIF-----      74 Agambiae(XP_309411.4-AGAP011232-PA)/162-434              14.4%     GRHVV----ATRQLRVGDVVMVE-KPYATVLSDH-M-----K--------------------RVRCAFCHAEEPF-----      75 Amellifera(XP_001120776.2-SMYD4-like-Predicted)/251-554  13.4%     GRHLV----VTKEFKPGDIITIE-DPYAYVIYTQ-R-----Y--------------------YTHCHHCLS-RSY-----      76 Dpulex(DAPPUDRAFT_305694-Predicted)/258-553              16.3%     GRCLV----ATEDIQIGTTVIVE-KALASILLEE-F-----K--------------------ESHCHHCLH-WTP-----      77 Dpulex(EFX87901.1)/258-554                               14.2%     GRCLV----ATEDIKIGETVIVE-KAHASILQYE-F-----K--------------------ESHCHHCLH-WTP-----      78 Amellifera(XP_001122116.2-SMYD4-like-Predicted)/234-534  13.5%     GRHIV----ATRKINPGEVIAIE-KPYSLILTPD-N-----I--------------------YTHCSNCLE-VSW-----      79 Hmagnipapillata(XP_002159692.1)/239-485                  13.7%     GRHAI----ASRDIKAGEVIIIE-KPFASLCLPE-C-----Y--------------------NTHCYHCLT-RFK-----      80 Nvectensis(XP_001623892.1)/215-512                       13.9%     GRHTI----AARDINIGDVLLVE-KPFASVLLQE-Q-----S--------------------KSHCHQCFV-HIL-----      81 Lgigantea(LOTGIDRAFT_143433)/100-395                     15.6%     --GIY----TTKDVEAGELLFCE-KPFASKNMHN-S-----D--------------------LTHCQNCLN-RVL-----      82 Skowalevskii(XP_002740933.1)/253-549                     16.3%     GRYAV----ATRDVKVGDVLIVE-NPYSSVGLQP-C-----N--------------------VSHCHHCYI-RVL-----      83 Dpulex(EFX73755.1)/49-306                                15.8%     MRNTG----KHEPIPKGTTILES-VPFVYCLKSS-F-----R--------------------RELCDFCLKA--------      84 Amellifera(XP_625013.1-SMYD3-Predicted)/1-253            15.0%     --MSE----SENFIKKGTTLFTA-KPFAYVLYSK-Y-----R--------------------NERCDYCFKS--------      85 Dmelanogaster(Buzidau-CG13761)/26-282                    14.4%     KNLKN----PAPQIKRGQRILTE-KPFAFVLKSQ-Y-----R--------------------LERCDNCLEA--------      86 Agambiae(XP_319707.4-AGAP008954-PA)/1-254                16.7%     ----M----RKTIHRRGDVILQE-KPFACVLDPR-Y-----R--------------------DSRCDRCFKE--------      87 cintestinalis(NP_001071820.1)/15-282                     18.6%     GRGLK----ATRKFETGQAVLKQ-EPYAYAVMSS-H-----I--------------------DVVCHYCLCAPGQP----      88 Drerio(Q6P0R5-Smyd1a)/18-279                             15.6%     GRGLR----GTRDLSAGEVVFAE-ASFAAVVLDS-L-----S--------------------LQVCHSCFRR--------      89 Derio(Q2MJQ9-Smyd1b)/13-274                              15.9%     GRGLR----ATKEAWAGDVLFAE-PPFASVVFDS-Q-----A--------------------SSICHSCFRR--------      90 Xtropicalis(NP_001120357.1-SMYD1)/13-261                 16.3%     GRGLR----AIRESWAGDIIFAE-PAYSAVVFDN-L-----S--------------------HSVCHSCFKR--------      91 Hsapiens(Q8NB12-SMYD1)/18-279                            16.1%     GRGLK----ATKEFWAADIIFAE-RAYSAVVFDS-L-----V--------------------NFVCHTCFKR--------      92 Ggallus(NP_989486.1-SMYD1)/13-274                        16.4%     GRGLK----AQKEFLPGDVIFAE-PAYAAVVFDS-L-----T--------------------HVICHTCFKR--------      93 Drerio(Q5RGL7-Smyd2b)/19-268                             15.0%     GRGLR----VSRAYGVGELLFSC-PAYSYVLSVG-E-----R--------------------GLICEQCFTR--------      94 Drerio(Q5BJI7-Smyd2a)/18-267                             15.2%     GRGLK----AIKHFKVGDLVFAC-PAYAYVLTVN-E-----R--------------------GGRCECCFTR--------      95 Xtropicalis(XP_002934751.2-SMYD2-like-Predicted)/16-265  15.2%     GRGLK----ATRPFALGELLFSC-PAYTYVLTVN-E-----R--------------------GNHCEFCFAR--------      96 Hsapiens(Q9NRG4-SMYD2)/18-267                            16.1%     GRGLR----ALQPFQVGDLLFSC-PAYAYVLTVN-E-----R--------------------GNHCEYCFTR--------      97 Ggallus(XP_419420.1-SMYD2-Predicted)/21-270              14.7%     GRGLR----ALRRYAVGELLFSC-PAYTAVLTVS-E-----R--------------------GSHCDGCFAR--------      98 cintestinalis(XP_002128556.1)/14-266                     18.0%     GRGLK----AKRNLNPGSTVLSS-EPYAYLLSKK-Q-----K--------------------GVYCDFCFKK--------      99 Lgigantea(LOTGIDRAFT_177746)/1-216                       15.4%     ------------MSKKGELIAKA-EPYVHVLAYK-E-----I--------------------DKLCSFCFLP--------     100 Drerio(E7EZZ6-SMYD3)/16-267                              18.8%     GNGLR----ALREIKPGEVIYSC-KPFAFCVARD-F-----L--------------------KTACQSCLKR--------     101 Xtropicalis(XP_004914684.1|-SMYD3-Predicted)/15-264      18.0%     GNGVR----ALKDMSHGLTVMIA-EPYVYTVCRI----------------------------KTACDHCLHR--------     102 Hsapiens(Q9H7B4-SMYD3)/15-266                            17.4%     GNGLR----AVTPLRPGELLFRS-DPLAYTVCKG-S-----R--------------------GVVCDRCLLG--------     103 Ggallus(XP_419536.1-SMYD3-Predicted)/15-266              18.0%     GSGLR----SRRQVRPGELLYRA-EPFAYVVTKE-Q-----L--------------------GGVCEQCLQR--------     104 Tadhaerens(XP_002109888.1)/20-262                        19.2%     GRGIR----CKKQLAIGTSVGKE-NPFCHVVSQD-M-----L--------------------SSYCHSCLLM--------     105 Hmagnipapillata(XP_002163555.2)/16-259                   17.2%     GRGVR----ALQDIKRGVEILKE-EPLACILTNSKY-----R--------------------GIRCDYCYSE--------     106 Nvectensis(XP_001627600.1)/17-253                        18.2%     GRGLR----AAKPLKSGDTILSE-QPVVYMLSNM-L-----R--------------------GQRCDFCLEK--------     107 Bfloridae(XP_002594889.1-BRAFLDRAFT_124463)/14-258       18.1%     GRGLC----ATKVFKPGNLVRAA-DPYAYVLCNS-E-----R--------------------GKRCDFCFAR--------     108 Skowalevskii(XP_006817727.1)/14-260                      15.8%     GRGYR----TVTRVKVGELVLKA-QPFVHVLCNT-E-----R--------------------GNRCDFCLRS--------         consensus/100%                                                     .................................................................s..h...........         consensus/90%                                                      Gphhh    s.p.h..sphlh.p .sh..h.... .......                 .....hC..Cht.........         consensus/80%                                                      GRhhh    stptht.Gphlh.- pshs.s.h.. .     .                  ....hCthChp.... .            consensus/70%                                                      GRtlh    uscplp.G-hlhhE pPhshs.h.. .     .                    h.tCphChp....                                                                               81          .         1         .         .         .         .         :         . 160   1 Tadhaerens(XP_002114620.1)/25-373                       100.0%     -----RRLSGN--PNVVLPHKEYCSTYAM--ENYVKCP-RCQ-VTMYCSS----------------------T-CLEKAV       2 Bfloridae(XP_002609030.1-BRAFLDRAFT_84846)/1-276         31.0%     -----RRLANS--PSLVLPFPQ-CCAVKL--EQHVTCP-HCQ-K-YTLP----------------------------SRN       3 cintestinalis(XP_002127168.1)/13-358                     39.9%     -----KRLAQN--PGLKLPYHE-CCESNP--VTYVHCQ---N-E-IYCSM----------------------E-CREKAY       4 Dpulex(EFX89935.1)/23-367                                41.0%     -----RRLTAK--ADLILPHPE-CDGTDK--SSHVICS-QCA-V-TYCSV----------------------D-CKDQAW       5 Dmelanogaster(CG3353-NP_650955.1)/13-363                 39.1%     -----RRLASD--PKVEVPLLQ-HDPTAQWVAQFTQCP-RCK-V-RYCSE----------------------D-CLMEAQ       6 Hmagnipapillata(XP_002163562.2)/21-371                   44.2%     -----RRLAAD--YTLELPYHEQCSLSVQRVNSIYKCP-NCC-I-PFCSK----------------------E-CYSEAY       7 Agambiae(XP_313299.1-AGAP003552-PA)/13-365               41.4%     -----QRLAND--PCIMLPRTE-CCPVEANLANHTKCE-RCG-A-LYCSA----------------------D-CLQEAA       8 Amellifera(XP_394075.2-SMYD5-like-Prediction)/16-364     45.0%     -----HRLTGN--STIILPHAE-CCETKK--ELITECS-ECG-T-KYCSI----------------------E-CQTDAY       9 Nvectensis(XP_001627062.1)/18-370                        48.0%     -----RRLSSN--PTLELPYSAECCAVTKAGEPISYCP-QCN-V-AYCSE----------------------N-CRIKAL      10 Skowalevskii(XP_002735533.1)/24-372                      50.1%     -----RRLSAI--SSLVLPYPQ-CCEVKK--DEHVSCP-ACQ-T-QYCST----------------------K-CKEDAE      11 Lgigantea(LOTGIDRAFT_231752)/19-367                      51.0%     -----RRLTNN--PALSLPHPE-CCALDP--SEFVVCP-QCQ-V-LYCSE----------------------E-CRKASW      12 Drerio(F1RET2-Smyd5)/32-380                              49.9%     -----RRLSGL--PALILPHPE-LCKVRP--DRHQACP-QCQ-V-MYCSS----------------------E-CRQAAM      13 Ggallus(NP_001012912.1-SMYD5)/39-387                     50.1%     -----QRLLGR--SSLVLPHPE-QCSIRK--DLHQQCP-RCQ-V-TYCSA----------------------E-CRQAAL      14 Hsapiens(Q6GMV2-SMYD5)/33-381                            49.9%     -----QRLTGK--PGQVLPHPE-LCTVRK--DLHQNCP-HCQ-V-MYCSA----------------------E-CRLAAT      15 Xtropicalis(A9ULL8-SMyd5)/32-382                         51.0%     -----QRLSGN--AHVVLPYPE-LCTVRN--GLHQQCP-RCQ-V-TYCSA----------------------E-CLKAAA      16 Lgigantea(LOTGIDRAFT_232186)/323-670                     20.5%     GD-ALETMTEEEKELVNIHWPD---------VTPIYCD-DCR-RVKYCSD----------------------D-CRLEAW      17 Bfloridae(XP_002589246.1-BRAFLDRAFT_74594)/380-720       22.0%     GMDTFRRMNKAQKAIIKKAWPK---------VTAYPCP-HCK-REKYCSL----------------------E-CRTHAW      18 Amellifera(XP_006565332.1)/43-285                        14.2%     --------------PL------------------FPCDKGCG--LPVCSI----------------------Q-CENSPN      19 Dmelanogaster(msta-CG33548)/66-313                       14.8%     --------------TG------------------FLCRHRCT--LPVCET------------------------CSDSEE      20 Dmelanogaster(CG12119)/34-280                            15.8%     --------------TR------------------FMCRQGCG--LPVCSL------------------------CAKKKQ      21 Amellifera(XP_006565301.1)/26-284                        15.8%     --------------IE-----E---------DVNYVCS-GCN-VVTLCGV----------------------T-CEERG-      22 Dmelanogaster(CG9642)/21-271                             13.6%     ---------------------------------ESFCR-KCR-LLALCED----------------------C-S-----      23 Dmelanogaster(CG9640)/17-268                             12.9%     ---------------------------------YVICR-RCQ-VFPLCMD----------------------C-N-----      24 Amellifera(NP_001229486.1-LOC724300)/57-301              12.7%     --------------AN------------------IRCT-KCL--WPACAV----------------------D-CSGLTD      25 Dmelanogaster(CG14590-NP_610202.3)/55-322                14.9%     --------------GK------------------HQCR-RCR--WPVCSA----------------------G-CKHES-      26 Dmelanogaster(CG43129)/21-279                            11.6%     --------------P-----------------EEELCS-ECG--WPLCVE----------------------C--AQQAD      27 Dmelanogaster(G11160)/58-319                             13.9%     --------------KA----------------DQYRCP-GCA--WPLCGS----------------------T-CAGLKH      28 Amellifera(XP_624539.3-msta-like-Predicted)/54-297       12.8%     --------------E-----------------IAIPCE-KCG--WPLCQ---------------------------NCNE      29 Dmelanogaster(CG8503-NP_610944.1)/52-301                 13.4%     --------------E-----------------DHIECE-QCG--WPLCGP----------------------E-CKSLDE      30 Agambiae(XP_309979.4-AGAP011530-PA)/50-300               14.8%     --------------K-----------------KYLDCE-RCG--WPVCKR----------------------S-CQDSPS      31 Dpulex(DAPPUDRAFT_120473)/58-292                         14.4%     --------------VQ-----E---------ASLFRCP-NCN--FPFCQE----------------------Q-CAKSPE      32 Dpulex(DAPPUDRAFT_194440-Predicted)/53-302               11.7%     --------------R-------------------TRCS-QCG--WPMCGR----------------------DDCHAHES      33 Dpulex(DAPPUDRAFT_2393)/50-297                           16.0%     --------------T-------------------YRCS-RCN--WPLCSA----------------------A-CEESAL      34 Dmelanogaster(CG18136-NP_649084.1)/58-318                15.9%     --------------PG---KPR---------GNYHKCS-SCS--WPLCGK----------------------E-CEDSVH      35 Agambiae(XP_309220.5-AGAP001025-PA)/55-318               16.2%     --------------GWDATRGL---------DRFHECS-RCG--WPLCGP----------------------G-CEEVAQ      36 Dmelanogaster(CG1868-NP_724802.1)/226-549                13.1%     --------------AP------------------IPCP-NCHQRVVYCSR----------------------K-CRE-AH      37 Agambiae(XP_319721.4-AGAP008973-PA)/165-486              13.3%     --------------VP------------------FPCP-TCG-RACYCST----------------------R-CRV-AH      38 Bfloridae(XP_002593048.1-BRAFLDRAFT_74375)/6-196         11.1%     --------------LL------------------LTCP-KCG-IAKYCDE----------------------D-CQS-AR      39 Bfloridae(XP_002594298.1-BRAFLDRAFT_117670)/15-265       13.8%     --------------NS------------------VSCD-ACR-TAKYCNE----------------------E-CKK-AA      40 Cintestinalis(XP_002123001.1)/195-567                    11.0%     --------------SG------------------IPCL-GCS-CTIYCDE----------------------Q-CRISAW      41 Drerio(Q08C84-Smyd4)/197-556                             11.1%     --------------SF------------------VPCP-KCS-YARYCGE----------------------S-CQKDAW      42 Xtropicalis(NP_001072288.1-SMYD4)/212-545                12.2%     --------------AS------------------LPCQ-YCS-FARYCSQ----------------------E-CMDKAW      43 Hsapiens(Q8IYR2-SMYD4)/244-602                           11.9%     --------------AT------------------VPCD-GCS-YAKYCSQ----------------------E-CLQQAW      44 Ggallus(NP_001025886.1-SMYD4)/241-573                    11.1%     --------------AS------------------IPCC-GCS-YAKYCSQ----------------------N-CADVAW      45 Hmagnipapillata(XP_002160254.2/232-532                   13.1%     --------------SL------------------FPCY-ECA-EVVFCSL----------------------S-CYNDAW      46 Dpulex(DAPPUDRAFT_312722-Pedicted)/241-525               14.2%     --------------KR------------------YPCL-ACG-KIWFCSD----------------------S-CRQE-S      47 Amellifera(XP_006565387.1-SMYD4-like-Predicted)/278-571  16.8%     --------------IP------------------VPCR-TCL-NTFYCNE----------------------N-CLTKAW      48 Bfloridae(XP_002589088.1-BRAFLDRAFT_75068)/251-714       10.4%     --------------VP------------------HPCR-GCQ-YVQYCSGTCEEQAWREYHRGCQYVQYCSRT-CEDQAW      49 Lgigantea(LOTGIDRAFT_169490)/248-638                     11.5%     --------------NL------------------VGCI-QCS-VVRYCSS----------------------K-CQEESW      50 Skowalevskii(XP_002733823.1)/75-447                      10.9%     --------------AP------------------IPCC-TCT-HVRYCSV----------------------E-CQQESW      51 Nvectensis(XP_001627273.1)/170-547                       12.5%     --------------AP------------------VPCN-HCS-SVLYCSA----------------------A-CRNKAW      52 Amellifera(XP_003250668.1-SMYD4-like-Predicted)/183-473  12.1%     --------------AL------------------IPCD-YCY-HALYCSK----------------------E-CRGKAY      53 Amellifera(XP_001121272.2-SMYD4-like-Predicted)/230-549  13.9%     -------------SVK------------------IPCY-YCQ-TVSFCSE----------------------K-CRSKAW      54 Amellifera(XP_003249162.1-SMYD4-like-Predicted)/239-589  11.5%     --------------AP------------------IGCP-DCS-SVAFCGR----------------------K-CRDTAL      55 Dmelanogaster(CG14122-NP_648574.1)/265-541               14.2%     --------------TP------------------VSCL-HCS-GIAFCSA----------------------Q-CMGEAC      56 Agambiae(XP_311885.3-AGAP002999-PA)/268-544              13.9%     --------------AP------------------VACP-DCC-GVAFCSV----------------------A-CRDKAC      57 Amellifera(XP_392262.3-SMYD4-like-Predicted)/252-555     15.0%     -------------PIP------------------AVCQ-TCS-CVAYCSI----------------------S-CRDKD-      58 Dmelanogaster(CG7759-NP_725048.1)/250-537                15.6%     -------------P--------------------VACP-RCA-DVLYCSE----------------------Q-CREEAS      59 Agambiae(XP_319583.4-AGAP008839-PA)/240-523              12.3%     -------------P--------------------IACP-LCA-DVVFCSD----------------------E-CETKAN      60 Dpulex(DAPPUDRAFT_68494-Predicted)/254-551               14.7%     -------------GEV------------------LPCS-HCD-LVSFCSV----------------------N-CRSRAM      61 Dpulex(DAPPUDRAFT_309882)/300-599                        12.5%     -------------IAP------------------LGCL-TCR-GVFYCSV----------------------E-CRDEAA      62 Dmelanogaster(CG8378-NP_610730.1)/196-491                13.8%     --------------TL------------------IPCD-SCC-STMFCSE----------------------E-CKSIAM      63 Agambiae(XP_566179.1-AGAP000216-PA)/158-458              13.6%     --------------TL------------------IPCE-RCT-KAMYCSK----------------------N-CLRRAR      64 Agambiae(XP_564258.1-AGAP011234-PA)/216-546              16.4%     --------------TL------------------IPCE-GCT-VAMYCSE----------------------E-CRDKAH      65 Agambiae(XP_309407.4-AGAP011238-PA)/219-497              15.4%     --------------TL------------------IPCE-GCT-VTMYCSE----------------------E-CRDKAH      66 Agambiae(XP_314169.4-AGAP005253-PB)/218-514              16.2%     --------------TL------------------IPCE-GCT-LTMYCSD----------------------E-CMDKAY      67 Agambiae(XP_309409.4-AGAP011237-PA)/206-481              15.2%     --------------TL------------------IPCE-GCT-VTMYCSK----------------------E-CMDKAH      68 Agambiae(XP_307865.2-AGAP009448-PA)/166-466              15.6%     --------------TL------------------IPCE-GCT-WVMYCSA----------------------E-CLGKAY      69 Agambiae(XP_309762.4-AGAP010931-PA)/113-383              14.4%     --------------TL------------------IPCE-GCT-VAMYCSE----------------------E-CISKAY      70 Agambiae(XP_309378.2-AGAP011267-PA)/149-447              16.9%     --------------TL------------------IPCE-GCT-AAMYCSE----------------------E-CLSKAY      71 Agambiae(XP_309383.4-AGAP011257-PA)/149-447              16.4%     --------------TL------------------IPCE-GCT-AAMYCSE----------------------E-CLSKAY      72 Agambiae(XP_307655.3-AGAP012638-PA)/149-447              16.4%     --------------TL------------------IPCE-GCT-MAMYCSE----------------------E-CLSKAY      73 Agambiae(XP_320681.4-AGAP011835-PA)/183-484              14.6%     --------------TL------------------LPCE-NCT-VAMYCSG----------------------S-CASQAA      74 Agambiae(XP_309411.4-AGAP011232-PA)/162-434              14.4%     --------------LL------------------IPCE-ECT-IAMYCSQ----------------------K-CLRAAW      75 Amellifera(XP_001120776.2-SMYD4-like-Predicted)/251-554  13.4%     --------------NL------------------IPCL-HCP-VAQYCSE----------------------K-CRILAW      76 Dpulex(DAPPUDRAFT_305694-Predicted)/258-553              16.3%     --------------GP------------------VPCH-QCS-QVGFCST----------------------L-CRDEAW      77 Dpulex(EFX87901.1)/258-554                               14.2%     --------------GP------------------VPCH-KCS-QVGFCST----------------------Q-CRDEAW      78 Amellifera(XP_001122116.2-SMYD4-like-Predicted)/234-534  13.5%     --------------AN------------------IPCE-YCT-YAMYCSE----------------------E-CKAMEW      79 Hmagnipapillata(XP_002159692.1)/239-485                  13.7%     --------------IN------------------YPCR-LCS-TVNYCSI----------------------S-CEKESW      80 Nvectensis(XP_001623892.1)/215-512                       13.9%     --------------AP------------------LPCS-YCT-TVRYCSE----------------------K-CAKESW      81 Lgigantea(LOTGIDRAFT_143433)/100-395                     15.6%     --------------SP------------------LPCD-QCS-GVVFCSE----------------------E-CKAEAM      82 Skowalevskii(XP_002740933.1)/253-549                     16.3%     --------------AS------------------IPCL-QCA-GIVYCSK----------------------E-CRNASW      83 Dpulex(EFX73755.1)/49-306                                15.8%     ------------NSNL------------------RKCL-GCM-VVSYCGR----------------------V-CQREGW      84 Amellifera(XP_625013.1-SMYD3-Predicted)/1-253            15.0%     ------------G-KL------------------FRCS-VCK-CIYYCNQ----------------------S-CQQMSW      85 Dmelanogaster(Buzidau-CG13761)/26-282                    14.4%     ------------T-KV------------------LKCS-NCR-YVSYCHR----------------------S-CQMQAW      86 Agambiae(XP_319707.4-AGAP008954-PA)/1-254                16.7%     ------------T-KV------------------MKCS-NCL-YVRYCGR----------------------S-CQKEAW      87 cintestinalis(NP_001071820.1)/15-282                     18.6%     ---------GAPVEDL------------------HRCT-GCK-FAQYCTK----------------------E-CQKKAW      88 Drerio(Q6P0R5-Smyd1a)/18-279                             15.6%     ------------QVNP------------------HRCA-QCK-FAHYCDR----------------------T-CQRAAW      89 Derio(Q2MJQ9-Smyd1b)/13-274                              15.9%     ------------QEKL------------------QRCG-QCR-FAQYCDK----------------------T-CQRAGW      90 Xtropicalis(NP_001120357.1-SMYD1)/13-261                 16.3%     ------------QEKL------------------LRCG-QCK-FAHYCDR----------------------T-CQKESW      91 Hsapiens(Q8NB12-SMYD1)/18-279                            16.1%     ------------QEKL------------------HRCG-QCK-FAHYCDR----------------------T-CQKDAW      92 Ggallus(NP_989486.1-SMYD1)/13-274                        16.4%     ------------QERL------------------HRCG-QCK-FAYYCDR----------------------T-CQRDAW      93 Drerio(Q5RGL7-Smyd2b)/19-268                             15.0%     ------------KKGL------------------AKCG-KCK-KAFYCNA----------------------N-CQKKNW      94 Drerio(Q5BJI7-Smyd2a)/18-267                             15.2%     ------------KEGL------------------SKCG-KCK-QAYYCNV----------------------E-CQRGDW      95 Xtropicalis(XP_002934751.2-SMYD2-like-Predicted)/16-265  15.2%     ------------KEGL------------------SKCG-KCK-QAFYCNV----------------------D-CQKGDW      96 Hsapiens(Q9NRG4-SMYD2)/18-267                            16.1%     ------------KEGL------------------SKCG-RCK-QAFYCNV----------------------E-CQKEDW      97 Ggallus(XP_419420.1-SMYD2-Predicted)/21-270              14.7%     ------------KEGL------------------SKCG-RCK-QAFYCNV----------------------E-CQKEDW      98 cintestinalis(XP_002128556.1)/14-266                     18.0%     ------------QDGL------------------LQCS-GCK-YMKYCNR----------------------N-CQKMAW      99 Lgigantea(LOTGIDRAFT_177746)/1-216                       15.4%     ------------CEKL------------------KKCA-ACG-LVKYCGV----------------------V-CQKADW     100 Drerio(E7EZZ6-SMYD3)/16-267                              18.8%     ------------GESL------------------SRCS-QCK-TARYCSV----------------------Q-CQKQAW     101 Xtropicalis(XP_004914684.1|-SMYD3-Predicted)/15-264      18.0%     ------------KEKL------------------LRCS-QCK-VTRYCNS----------------------H-CQRKAW     102 Hsapiens(Q9H7B4-SMYD3)/15-266                            17.4%     ------------KEKL------------------MRCS-QCR-VAKYCSA----------------------K-CQKKAW     103 Ggallus(XP_419536.1-SMYD3-Predicted)/15-266              18.0%     ------------NEHL------------------HRCS-QCK-VAKYCGK----------------------S-CQKEAW     104 Tadhaerens(XP_002109888.1)/20-262                        19.2%     ------------QSEL------------------YKCS-RCK-IIMYCCK----------------------S-CQKEDW     105 Hmagnipapillata(XP_002163555.2)/16-259                   17.2%     ------------PEKL------------------LKCS-KCK-FIAYCGK----------------------V-CQASDW     106 Nvectensis(XP_001627600.1)/17-253                        18.2%     ------------LSDL------------------QRCS-RCK-FARYCGA----------------------S-CQRAAW     107 Bfloridae(XP_002594889.1-BRAFLDRAFT_124463)/14-258       18.1%     ------------KDDM------------------SRCS-GCK-FARYCDG----------------------K-CQKAAW     108 Skowalevskii(XP_006817727.1)/14-260                      15.8%     ------------TESL------------------LRCS-SCK-FSRYCNV----------------------K-CQRSAW         consensus/100%                                                     ....................................C.........hh................................         consensus/90%                                                           ...... .......... ......  .....C. tCt ...hCs.                      t C...s.         consensus/80%                                                                  ..t.                ..h.C. tCt .h.aCs.                      p C.pts.         consensus/70%                                                                   .t.                  h.Ct tCp hhhYCut                      p Cpppuh                                                                         161          .         .         .         2         .         .         .         . 240   1 Tadhaerens(XP_002114620.1)/25-373                       100.0%     KEYHRS-LCCGSNN--CRPD-HSL------------NRLRETWRNIHYPPETSSIMLIAKMIAMIEQ--ADD------PM       2 Bfloridae(XP_002609030.1-BRAFLDRAFT_84846)/1-276         31.0%     GQYHDD-C-QN-------DS-HGQ------------AACDHCMRSM----ETAEAM--SRR--------LAN------SH       3 cintestinalis(XP_002127168.1)/13-358                     39.9%     NEFHKI-LCPSSDL--ID-R-NAL------------EILDETWRGCHYPPETASIQMIIRILARIKQ--EEK------KE       4 Dpulex(EFX89935.1)/23-367                                41.0%     NQYHKT-ICCNMFG--GNSN-HPL------------EKLNEAWKKMHYPPETSTIMLLVRILANFIQ--RTD------RE       5 Dmelanogaster(CG3353-NP_650955.1)/13-363                 39.1%     KRYHRV-ACMGAFH--SDDT-HPI------------NVLNETWKKMHYPPETGSIMLIVRLMALYQQ--STK------KE       6 Hmagnipapillata(XP_002163562.2)/21-371                   44.2%     EKYHKS-LCLHPDE--M-SE-SPV------------YRIEEAWKQLHYPPETASVMLIVRILAMIAQ--SQC------PE       7 Agambiae(XP_313299.1-AGAP003552-PA)/13-365               41.4%     NRYHTA-VCLGSKA--HNEQ-HPV------------NALVEFWKKMHYPPETCGIMLFVKIVGMFRQ--AAD------PQ       8 Amellifera(XP_394075.2-SMYD5-like-Prediction)/16-364     45.0%     LRYHST-ICLQSRE--KDES-HPL------------VQLNETWKQMHYPPETASIMLLVKMVALVNQ--ANN------KE       9 Nvectensis(XP_001627062.1)/18-370                        48.0%     DQYHRI-LCLGTST--PDPN-HPL------------VKLQETWKNIHYPPETANIMLIARIMATILQ--ATN------SD      10 Skowalevskii(XP_002735533.1)/24-372                      50.1%     KLYHRV-LCMGQHP--ADPE-HPI------------AKLQDIWRNMHFPPETASIMLIAKMIAKIKQ--APD------KS      11 Lgigantea(LOTGIDRAFT_231752)/19-367                      51.0%     DRYHQI-LCLGSSH--HDSD-HPL------------LRLQEIWRNIHYPPETASIMLICKMIAMVKQ--AED------PG      12 Drerio(F1RET2-Smyd5)/32-380                              49.9%     DQYHKI-LCLGPSN--DDPD-HPV------------NKLQDAWRSVHFPPETSSVMILAKMVATIKQ--TQD------KE      13 Ggallus(NP_001012912.1-SMYD5)/39-387                     50.1%     EQYHQV-LCLGPSR--DDPT-HPL------------NKLQEAWRNMHYPPETSSIMLMARMVATVKQ--AKD------KD      14 Hsapiens(Q6GMV2-SMYD5)/33-381                            49.9%     EQYHQV-LCPGPSQ--DDPL-HPL------------NKLQEAWRSIHYPPETASIMLMARMVATVKQ--AKD------KD      15 Xtropicalis(A9ULL8-SMyd5)/32-382                         51.0%     DQYHRA-LCLGASR--DNPA-HPL------------NKLEEAWRNMHYPPETASIMLMARMVGTIKQVQAQD------KD      16 Lgigantea(LOTGIDRAFT_232186)/323-670                     20.5%     DLYHQI-ICPKLNP--ASSE-LYDLLDNEGWGIRDDGTKGEIWGGH------YSLMILANIWASIIM--EAK------RL      17 Bfloridae(XP_002589246.1-BRAFLDRAFT_74594)/380-720       22.0%     RQHHCH-LCPSINP--PAAK-LYDFCAK------GTTQEKGMWNSM------FSPMIMARIWANILT--RVK------EL      18 Amellifera(XP_006565332.1)/43-285                        14.2%     ---HVN-ECEYLRS--LIPT-CGT--------------DW----C---L-NLLLAMIPIRGLFMT----KMQ--------      19 Dmelanogaster(msta-CG33548)/66-313                       14.8%     ---HQA-ECEHFRR--WQPK-DVD--------------AE----QEQVNPMSLRILTAVRVFHLG----KEQ--------      20 Dmelanogaster(CG12119)/34-280                            15.8%     ---HKS-DCDLFKS--WGPN-EPD--------------VA--------NSVIIRLLCVARAINLS----KEQ--------      21 Amellifera(XP_006565301.1)/26-284                        15.8%     -IYHSAYECEIIKN--NEEL-S-----------------------IENTDVLAGVLFVLRLWLLK----QKD--------      22 Dmelanogaster(CG9642)/21-271                             13.6%     --DHDERDCKRLAE--MNFS-DDQ--------------V-----ELLQKKEHTEIQPVLKCLLLR----EHE--------      23 Dmelanogaster(CG9640)/17-268                             12.9%     --QHDEFECEFFTS--GAGK-ALC--------------K-----DILVK--NFGICGLLKLLLLL----ENP--------      24 Amellifera(NP_001229486.1-LOC724300)/57-301              12.7%     KNRHDL-ECSFLIK--AK------------------------------IIPRCDVLLVIRMLILW----CKK--------      25 Dmelanogaster(CG14590-NP_610202.3)/55-322                14.9%     ------MECSVLSL--GSGS-PTR--------------ADARSLNDY---FRGDALLVLKCLLLQ----RQS--------      26 Dmelanogaster(CG43129)/21-279                            11.6%     -NAHFRLECSQLKD--ARAR-FFR--------------L----PSGSRHCPQLDCIMPLRVLLAK----EAN--------      27 Dmelanogaster(G11160)/58-319                             13.9%     RHGHTETECQLYAE--RRAV-AGE--------------LLTERAGPAEVRDLYELVMIVRILLLR----QHD--------      28 Amellifera(XP_624539.3-msta-like-Predicted)/54-297       12.8%     ---HGL-ECKFSSS--RRDS-KIS--------------I----TEFGYPHPSYQCINVIRALSLK----DTN--------      29 Dmelanogaster(CG8503-NP_610944.1)/52-301                 13.4%     ---HKA-ECGLTKD---RGQ-KVN--------------V----QEFGGPHPLYTCLSTVRCLLIG----ETS--------      30 Agambiae(XP_309979.4-AGAP011530-PA)/50-300               14.8%     ---HQA-ECKFTIA---RGS-KIS--------------I----QHFYVPHPTYQCLMPVRCLLLA----ESD--------      31 Dpulex(DAPPUDRAFT_120473)/58-292                         14.4%     ---HEA-ECLILSR---AKS-CIV--------------IN----------------------DVH----RIH--------      32 Dpulex(DAPPUDRAFT_194440-Predicted)/53-302               11.7%     ---DHAAECGVMAS---GGR----------------------PIVGSLPVQAYQSVMVLRCLALR----DQN--------      33 Dpulex(DAPPUDRAFT_2393)/50-297                           16.0%     ---HKNGECRMIDP---TLM-TNH--------------L----SQGAINSQVFQCITPLRYLTLP----DSD--------      34 Dmelanogaster(CG18136-NP_649084.1)/58-318                15.9%     ---HKA-ECQLMSG---SNF-QSK--------------INYVPGEEERKESAYCVIMLLRCMHLK----DKD--------      35 Agambiae(XP_309220.5-AGAP001025-PA)/55-318               16.2%     ---HRP-ECSVLAG---SGY-RPN--------------IRPNPSNPEQRESAYCVIVPLRVLLLE----RIA--------      36 Dmelanogaster(CG1868-NP_724802.1)/226-549                13.1%     SAIHKF-ECAAYRKDILRLL-G-------------------------------ISHLALRLLLTY---------IPYIRP      37 Agambiae(XP_319721.4-AGAP008973-PA)/165-486              13.3%     RPVHRF-ECFGYQKHLWYQI-G-------------------------------IAHLGLRCFLDG---------FGTIAG      38 Bfloridae(XP_002593048.1-BRAFLDRAFT_74375)/6-196         11.1%     KY-----------------------------------------------------------------------------E      39 Bfloridae(XP_002594298.1-BRAFLDRAFT_117670)/15-265       13.8%     KFHHTP-ECRGYSR--LMNL-P------------------------------EHLRVMGRILYKM---------HARKTD      40 Cintestinalis(XP_002123001.1)/195-567                    11.0%     KIYHWM-ECSVIPM--LAIK-C------------------------------MELRVAVRALLTG---------AYELGE      41 Drerio(Q08C84-Smyd4)/197-556                             11.1%     DQWHQW-ECPVGAD--LLAI-G------------------------------VLGHLALRVVLKA---------GQTEVQ      42 Xtropicalis(NP_001072288.1-SMYD4)/212-545                12.2%     RSYHYI-ECSMGDL--LLAL-G------------------------------MFCHTALRAVLVA---------GCRLFS      43 Hsapiens(Q8IYR2-SMYD4)/244-602                           11.9%     ELYHRT-ECPLGGL--LLTL-G------------------------------VFCHIALRLTLLV---------GFEDVR      44 Ggallus(NP_001025886.1-SMYD4)/241-573                    11.1%     EQYHRT-ECPLGAL--LLTL-G------------------------------VFFHVALRTVLLA---------GFSEVS      45 Hmagnipapillata(XP_002160254.2/232-532                   13.1%     ATYHRF-ECKKLSL--MEKV-G-------------------------------IAHLSLRIVLVS---------DAKDLL      46 Dpulex(DAPPUDRAFT_312722-Pedicted)/241-525               14.2%     SCYHNF-ECGLEAV--LNSV-G-------------------------------IAHLGARIVLSH---------GLDSVL      47 Amellifera(XP_006565387.1-SMYD4-like-Predicted)/278-571  16.8%     SSYHCW-ECPGNQMNLWKEI-G-------------------------------IGHLALKVLLTC---------STITDK      48 Bfloridae(XP_002589088.1-BRAFLDRAFT_75068)/251-714       10.4%     KEYHSY-ECEHWHL--LQMV-E------------------------------TFAQLSLRLLLTA---------AARGEK      49 Lgigantea(LOTGIDRAFT_169490)/248-638                     11.5%     KLYHSV-ECPYLDL--LHSV-G-------------------------------IAHLSLRTVLTA---------GLQFLT      50 Skowalevskii(XP_002733823.1)/75-447                      10.9%     KSYHYI-ECPLWPF--LSQA-G------------------------------NFSQLSLRILLKA---------GWSNIQ      51 Nvectensis(XP_001627273.1)/170-547                       12.5%     SQYHHV-ECEIFPV--LEIV-D------------------------------TFTHLSLRILLTT---------SAKDII      52 Amellifera(XP_003250668.1-SMYD4-like-Predicted)/183-473  12.1%     QAYHQI-YCRYGNL--DNKS-S------------------------------FVLKLLLKITDNG--------ARLKEAL      53 Amellifera(XP_001121272.2-SMYD4-like-Predicted)/230-549  13.9%     QIYHQY-ECFIFDV--FFEN-DSE--------------QI--------QRNTSYLLLAYRMIISGFLSST---EQIKNIE      54 Amellifera(XP_003249162.1-SMYD4-like-Predicted)/239-589  11.5%     ASYHKY-ECKILVL--LIGS-G----------------------------MSVLSMLALRMATQV---------GPAGCL      55 Dmelanogaster(CG14122-NP_648574.1)/265-541               14.2%     SSYHRF-ECEYMDL--MIGS-G----------------------------MSILCFIALRIFTQA---------PSLEQG      56 Agambiae(XP_311885.3-AGAP002999-PA)/268-544              13.9%     ATYHRF-ECQYLDL--MIGS-G----------------------------MSILCHVALRMVTQA---------GTPEKV      57 Amellifera(XP_392262.3-SMYD4-like-Predicted)/252-555     15.0%     AKIHEN-ECSILPT--LWAS-K----------------------------TSINCFLALRIIVQQ---------SFEKLY      58 Dmelanogaster(CG7759-NP_725048.1)/250-537                15.6%     KKYHKY-ECGIVPI--IWRS-G----------------------------ASINNHIALRIIASK---------PLDYFL      59 Agambiae(XP_319583.4-AGAP008839-PA)/240-523              12.3%     ATYHRY-ECGFLPI--LWGS-G----------------------------ASITCHMALRMITQK---------SEEYFL      60 Dpulex(DAPPUDRAFT_68494-Predicted)/254-551               14.7%     EIYHAI-ECPILSC--LYAA-G----------------------------ISIICYLSLRMIAIH---------PPSFFM      61 Dpulex(DAPPUDRAFT_309882)/300-599                        12.5%     STYHQY-ECGIIDY--MIAS-G----------------------------SSILSWIALRILTKG---------KMEDFL      62 Dmelanogaster(CG8378-NP_610730.1)/196-491                13.8%     QTYHRY-ECPIIDF--LNRM-FNK--------------I---------------HCIALRTTLVALNIFPS-IEELIDFC      63 Agambiae(XP_566179.1-AGAP000216-PA)/158-458              13.6%     TEYHEF-ECALVHH--LTET-TRD--------------P--------------VVLLAWRAVTRAISTYRYNLRHLKQRR      64 Agambiae(XP_564258.1-AGAP011234-PA)/216-546              16.4%     KQYHRY-ECAVLRD--CWRS-VGF--------------P-------------VEMLLGLRTVATAFASFDQSLGQWIYRM      65 Agambiae(XP_309407.4-AGAP011238-PA)/219-497              15.4%     KQYHRY-ECGVLRD--CWRI-VGH--------------L-------------FGGMVGLRTVATAIASFDQDLEGWNDHL      66 Agambiae(XP_314169.4-AGAP005253-PB)/218-514              16.2%     KQYHRY-ECGVLRD--CWRI-AGR--------------L-------------VGGIVGLRMVATAIASFEQDLEGWTNHL      67 Agambiae(XP_309409.4-AGAP011237-PA)/206-481              15.2%     KQYHRY-ECGVLRD--CWRM-IGS--------------L-------------PGGIMGLRTVATAFASFEQDLEGWIDHL      68 Agambiae(XP_307865.2-AGAP009448-PA)/166-466              15.6%     SQYHRY-ECGVMRD--LWRV-AGK--------------C---------------PMTAVRTVASAFGTFDDDPDALQAHL      69 Agambiae(XP_309762.4-AGAP010931-PA)/113-383              14.4%     GKYHRY-ECGVLRD--LWTV-LGI--------------S---------------GVIALRMIAIAITTFDNDLEKLKDHL      70 Agambiae(XP_309378.2-AGAP011267-PA)/149-447              16.9%     NKYHRY-ECGLLRD--MVEV-FDE--------------L---------------PLIAIRMIAIAITTFDNNPEALKDHL      71 Agambiae(XP_309383.4-AGAP011257-PA)/149-447              16.4%     NNYHRY-DCGILRD--LYED-FEE--------------V---------------SLIDIRMIAIAITTFDNNPEALKDHL      72 Agambiae(XP_307655.3-AGAP012638-PA)/149-447              16.4%     NKYHRY-ECGLLRD--MWEV-FEE--------------V---------------SLIDIRMIAIAITTFDNNPEALKDHL      73 Agambiae(XP_320681.4-AGAP011835-PA)/183-484              14.6%     RQYHRY-ECPIIRD--MWRI-FTK--------------L---------------PVMSLRTVTTAISAFEYDLQEMWEHL      74 Agambiae(XP_309411.4-AGAP011232-PA)/162-434              14.4%     QQYHRY-ECPILND--MRTI-GTE--------------Y---------------LALAVRTVAIALASFDHDLEALRAHL      75 Amellifera(XP_001120776.2-SMYD4-like-Predicted)/251-554  13.4%     EMAHDI-ECPIMAL--IGNL-L------------------------------HVDKDKIRMLTKIIRFLIIATAKGKNIN      76 Dpulex(DAPPUDRAFT_305694-Predicted)/258-553              16.3%     ASYHQS-ECGLTDS--LH-----G--------------TN----------VGRHGLLAVRTVLKV---------GRQRIM      77 Dpulex(EFX87901.1)/258-554                               14.2%     DSYHQF-ECGLTDF--LCRT-TRD--------------VN----------TGQHGLLALRTVLKA---------DRRLII      78 Amellifera(XP_001122116.2-SMYD4-like-Predicted)/234-534  13.5%     KKYHDI-ECAIFPS--ML-----K--------------MN----------FVKLDLFSLRLAIQA-------VREATSIQ      79 Hmagnipapillata(XP_002159692.1)/239-485                  13.7%     EKFHCF-ECEYLGV--LI-----N--------------DD----------V-GLAHLAFKIITNV---------GISMLL      80 Nvectensis(XP_001623892.1)/215-512                       13.9%     DAYHYA-ECMNLEH--VY-----V--------------A-----------G-KYGHLALRVVVKA---------GFQYLK      81 Lgigantea(LOTGIDRAFT_143433)/100-395                     15.6%     KSFHFA-ECRVLET--IH-----N--------------ID----------F-GLGHLALRMVLKA---------GLNHIL      82 Skowalevskii(XP_002740933.1)/253-549                     16.3%     EMYHNL-ECHHLDL--IQ-----E--------------LG----------L-GMGHLALRTIIRT---------GLAFLL      83 Dpulex(EFX73755.1)/49-306                                15.8%     KD-HKG-ECKNFVR--VKPNV-----------------------------PTDSVRLIARLILKLQVINGYI-------L      84 Amellifera(XP_625013.1-SMYD3-Predicted)/1-253            15.0%     TI-HSK-ECASLKR--FSSKV-----------------------------IPDVARLMARIIIKLNQGGG---------E      85 Dmelanogaster(Buzidau-CG13761)/26-282                    14.4%     GQ-HKH-ECPFLKK--VHPRV-----------------------------VPDAARMLCRLILRLEHGGD---------L      86 Agambiae(XP_319707.4-AGAP008954-PA)/1-254                16.7%     SD-HKE-ECEKLKA--LPPGLV----------------------------VPSAALMIARIVRRLLKGGD---------T      87 cintestinalis(NP_001071820.1)/15-282                     18.6%     PE-HKQ-ECAAIKR--ITPG-K----------------------------PVDQTRLVGRILWRRKREENLN-------G      88 Drerio(Q6P0R5-Smyd1a)/18-279                             15.6%     DE-HRK-ECSAIRN--IG-K-A----------------------------PNENVRLVARILWRIQKHTGLV-------S      89 Derio(Q2MJQ9-Smyd1b)/13-274                              15.9%     EE-HKL-ECAAIKT--YG-K-P----------------------------PSENVRLAARILWRMDKQGSVV-------S      90 Xtropicalis(NP_001120357.1-SMYD1)/13-261                 16.3%     AN-HKN-ECVAIKK--AG-K-A----------------------------PNENIRLAARILWRIEREGSGL-------T      91 Hsapiens(Q8NB12-SMYD1)/18-279                            16.1%     LN-HKN-ECSAIKR--YG-K-V----------------------------PNENIRLAARIMWRVEREGTGL-------T      92 Ggallus(NP_989486.1-SMYD1)/13-274                        16.4%     LN-HKN-ECSAIKK--HG-K-A----------------------------PTENIRLAARILWRIEREGGGL-------S      93 Drerio(Q5RGL7-Smyd2b)/19-268                             15.0%     PM-HKL-ECQAMCA--FGENWR----------------------------PSETVRLVARIIARLKAQKERS-------P      94 Drerio(Q5BJI7-Smyd2a)/18-267                             15.2%     PM-HKL-ECSAMCA--YGENWC----------------------------PSETVRLVARIILKQKHQTERT-------P      95 Xtropicalis(XP_002934751.2-SMYD2-like-Predicted)/16-265  15.2%     PM-HKL-ECSAMCT--YGQNWC----------------------------PSETVRLTARILAKQKTQTERT-------A      96 Hsapiens(Q9NRG4-SMYD2)/18-267                            16.1%     PM-HKL-ECSPMVV--FGENWN----------------------------PSETVRLTARILAKQKIHPERT-------P      97 Ggallus(XP_419420.1-SMYD2-Predicted)/21-270              14.7%     PM-HKL-ECAAMCA--FGQNWN----------------------------PSETVRLTARILAKQKIHPERT-------Q      98 cintestinalis(XP_002128556.1)/14-266                     18.0%     NEHHKA-ECPALKN--VMPK-R----------------------------PPDFVILLGRLLWNMQQYS-SA-------K      99 Lgigantea(LOTGIDRAFT_177746)/1-216                       15.4%     PI-HKT-ECPCFKE--SQPI-I----------------------------PTDSVRLFLRIIIRHMEWQ-MI-------D     100 Drerio(E7EZZ6-SMYD3)/16-267                              18.8%     PD-HKR-ECKCLKH--LQPR-I----------------------------PTDSVRLVARIIFKLLSQS-ES-------D     101 Xtropicalis(XP_004914684.1|-SMYD3-Predicted)/15-264      18.0%     QG-HKR-ECKCLRS--TLPN-V----------------------------PPNSVRLVGKIIFKMLQKP-DT-------A     102 Hsapiens(Q9H7B4-SMYD3)/15-266                            17.4%     PD-HKR-ECKCLKS--CKPR-Y----------------------------PPDSVRLLGRVVFKLMDGA-PS-------E     103 Ggallus(XP_419536.1-SMYD3-Predicted)/15-266              18.0%     LD-HKR-ECKCLQN--VKPN-F----------------------------PPDSVRLAGRIVFKLLRQS-AC-------L     104 Tadhaerens(XP_002109888.1)/20-262                        19.2%     QW-HKY-ECKSITR--LGPK-V----------------------------PPDSIRLLGRVAYTILQGQ-D---------     105 Hmagnipapillata(XP_002163555.2)/16-259                   17.2%     KM-HKY-ECKCLTK--SAPK-Q----------------------------PPDFCRLVSQLIFNFYYNK-K---------     106 Nvectensis(XP_001627600.1)/17-253                        18.2%     RI-HKS-ECERLKR--VFPR-V----------------------------PTDLVLLMFRVWQLKSQ-------------     107 Bfloridae(XP_002594889.1-BRAFLDRAFT_124463)/14-258       18.1%     TE-HKS-ECKSIKT--VKPE-T----------------------------PTDSIRLIARIINKTKTDS-P--------G     108 Skowalevskii(XP_006817727.1)/14-260                      15.8%     TC-HKA-ECKSLKK--VSPR-I----------------------------PPGSVRLMSRILYKLKDKS-C--------E         consensus/100%                                                     ................................................................................         consensus/90%                                                      ...Ht. .C.....  .... ...            ...................hh.h+hhh.................         consensus/80%                                                      ...Hp. -C.hht.  h... ...              ..  ............hhhhhRhlhhh.......  ......         consensus/70%                                                      t..Hph EC.hhp.  h... h..              .           ....hhlhhRhlhhh.......  ......                                                                         241          :         .         .         .         .         3         .         . 320   1 Tadhaerens(XP_002114620.1)/25-373                       100.0%     DVLK-------------------------------------------------------------------------L--       2 Bfloridae(XP_002609030.1-BRAFLDRAFT_84846)/1-276         31.0%     SLVL-------------------------------------------------------------------------P--       3 cintestinalis(XP_002127168.1)/13-358                     39.9%     EFIS-------------------------------------------------------------------------D--       4 Dpulex(EFX89935.1)/23-367                                41.0%     ELKS-------------------------------------------------------------------------Q--       5 Dmelanogaster(CG3353-NP_650955.1)/13-363                 39.1%     EFLE-------------------------------------------------------------------------Q--       6 Hmagnipapillata(XP_002163562.2)/21-371                   44.2%     MVMS-------------------------------------------------------------------------E--       7 Agambiae(XP_313299.1-AGAP003552-PA)/13-365               41.4%     ALQA-------------------------------------------------------------------------E--       8 Amellifera(XP_394075.2-SMYD5-like-Prediction)/16-364     45.0%     DILS-------------------------------------------------------------------------T--       9 Nvectensis(XP_001627062.1)/18-370                        48.0%     VKKG-------------------------------------------------------------------------S--      10 Skowalevskii(XP_002735533.1)/24-372                      50.1%     EAVA-------------------------------------------------------------------------A--      11 Lgigantea(LOTGIDRAFT_231752)/19-367                      51.0%     HVIT-------------------------------------------------------------------------I--      12 Drerio(F1RET2-Smyd5)/32-380                              49.9%     RWQR-------------------------------------------------------------------------L--      13 Ggallus(NP_001012912.1-SMYD5)/39-387                     50.1%     WWIK-------------------------------------------------------------------------A--      14 Hsapiens(Q6GMV2-SMYD5)/33-381                            49.9%     RWIR-------------------------------------------------------------------------L--      15 Xtropicalis(A9ULL8-SMyd5)/32-382                         51.0%     WWMH-------------------------------------------------------------------------L--      16 Lgigantea(LOTGIDRAFT_232186)/323-670                     20.5%     MITD--------------------------------------------------------------------GATTAT--      17 Bfloridae(XP_002589246.1-BRAFLDRAFT_74594)/380-720       22.0%     GVKG-----------------------------------------------------------------------EPT--      18 Amellifera(XP_006565332.1)/43-285                        14.2%     ---------------------------------------------------------------------------R---K      19 Dmelanogaster(msta-CG33548)/66-313                       14.8%     ---------------------------------------------------------------------------R---H      20 Dmelanogaster(CG12119)/34-280                            15.8%     ---------------------------------------------------------------------------R---D      21 Amellifera(XP_006565301.1)/26-284                        15.8%     ---------------------------------------------------------------------------PELWK      22 Dmelanogaster(CG9642)/21-271                             13.6%     -------------------------------------------------------------------------ETLPLYE      23 Dmelanogaster(CG9640)/17-268                             12.9%     -------------------------------------------------------------------------RTKGDCQ      24 Amellifera(NP_001229486.1-LOC724300)/57-301              12.7%     ---------------------------------------------------------------------------SKYWN      25 Dmelanogaster(CG14590-NP_610202.3)/55-322                14.9%     ---------------------------------------------------------------------------PTKWS      26 Dmelanogaster(CG43129)/21-279                            11.6%     ---------------------------------------------------------------------------PERWD      27 Dmelanogaster(G11160)/58-319                             13.9%     ---------------------------------------------------------------------------PEQFA      28 Amellifera(XP_624539.3-msta-like-Predicted)/54-297       12.8%     ---------------------------------------------------------------------------PESYK      29 Dmelanogaster(CG8503-NP_610944.1)/52-301                 13.4%     ---------------------------------------------------------------------------TEKAS      30 Agambiae(XP_309979.4-AGAP011530-PA)/50-300               14.8%     ---------------------------------------------------------------------------PARWE      31 Dpulex(DAPPUDRAFT_120473)/58-292                         14.4%     ---------------------------------------------------------------------------P----      32 Dpulex(DAPPUDRAFT_194440-Predicted)/53-302               11.7%     ---------------------------------------------------------------------------PERWD      33 Dpulex(DAPPUDRAFT_2393)/50-297                           16.0%     ---------------------------------------------------------------------------RERL-      34 Dmelanogaster(CG18136-NP_649084.1)/58-318                15.9%     ---------------------------------------------------------------------------PDAFL      35 Agambiae(XP_309220.5-AGAP001025-PA)/55-318               16.2%     ---------------------------------------------------------------------------PERYA      36 Dmelanogaster(CG1868-NP_724802.1)/226-549                13.1%     HL-QEMTSA-----K-GMWE-----------------------------------------EIMNLSRKPEESENAPEYL      37 Agambiae(XP_319721.4-AGAP008973-PA)/165-486              13.3%     EM-AKATDA-----S-VCYQ-----------------------------------------RVLEATR--EEDNPFSHYG      38 Bfloridae(XP_002593048.1-BRAFLDRAFT_74375)/6-196         11.1%     Q---------------------------------------------------------------------------GALG      39 Bfloridae(XP_002594298.1-BRAFLDRAFT_117670)/15-265       13.8%     M---------------------------------------------------------------------------GALG      40 Cintestinalis(XP_002123001.1)/195-567                    11.0%     TPQHDTTHTCTSIAK-HIY------------------QSR---------------NS-CADCSEPVGTDPKDGVYKCDYW      41 Drerio(Q08C84-Smyd4)/197-556                             11.1%     MGIKNTKDHVTTYKN-DSPV--------------------Q-------LS-----LG-GDCGKSLD---HTDCFHGSSYM      42 Xtropicalis(NP_001072288.1-SMYD4)/212-545                12.2%     QSLEQTGSADATDKT-KVCN----------------------------------------------SKSTYHEKYCSSYQ      43 Hsapiens(Q8IYR2-SMYD4)/244-602                           11.9%     KIITKLCDKI--SNK-DICLPE----------SNNQVKTLN-------YGLGESEKN-GNIVETPIPGCDINGKYENNYN      44 Ggallus(NP_001025886.1-SMYD4)/241-573                    11.1%     RLVEWS-RDD--SNK-DLCNAE----------AGGEHPSEA--------------LD-TRAGRKVIPGCNDNGQYQSSYQ      45 Hmagnipapillata(XP_002160254.2/232-532                   13.1%     RFLGSDLNKFTDSPT---------------------------------------------LPSSKIEGCNDQGIYQGDYE      46 Dpulex(DAPPUDRAFT_312722-Pedicted)/241-525               14.2%     AFLKDTDKV------------------------------------------------------KKVPGIDGP-YDTKSYQ      47 Amellifera(XP_006565387.1-SMYD4-like-Predicted)/278-571  16.8%     IKFNEMQN------------------------------------------------------------------------      48 Bfloridae(XP_002589088.1-BRAFLDRAFT_75068)/251-714       10.4%     HPSADMESP--ATAS---------------------------------------------KPSDQAKLCTDKVSPTSDGA      49 Lgigantea(LOTGIDRAFT_169490)/248-638                     11.5%     DFIKERKDD--ESKK---------------------------------------------TANSRLPGLNERGKYERSYD      50 Skowalevskii(XP_002733823.1)/75-447                      10.9%     KYSKEVSNP---------------------------------------------------VSSSHIPGCDTRGNYKSDYN      51 Nvectensis(XP_001627273.1)/170-547                       12.5%     DVLNGLSRD--V--A---------------------------------------------TTSCSLPGCTVSGSYPGDYG      52 Amellifera(XP_003250668.1-SMYD4-like-Predicted)/183-473  12.1%     EYHKELENMSEEMEK---------------------------------------------------KVYNLKEMKENNLR      53 Amellifera(XP_001121272.2-SMYD4-like-Predicted)/230-549  13.9%     KKKISFL-----NNNFLQYY--------------------------------VTNIN-----KERSNLGTNEIYSPYDYR      54 Amellifera(XP_003249162.1-SMYD4-like-Predicted)/239-589  11.5%     RIHRALNRQDSAADGEEIAESSTITTTAEKLSKSAKRRSRRKKLRDSRRAKGEETVEEERREIKGEEGEDGEKMAENVDL      55 Dmelanogaster(CG14122-NP_648574.1)/265-541               14.2%     --------------------------------------------------------------------------LATANL      56 Agambiae(XP_311885.3-AGAP002999-PA)/268-544              13.9%     --------------------------------------------------------------------------LEEGKM      57 Amellifera(XP_392262.3-SMYD4-like-Predicted)/252-555     15.0%     KLKDVKENSKDKF-----------------------------------------------------EVSASEPYRSNDFK      58 Dmelanogaster(CG7759-NP_725048.1)/250-537                15.6%     KLKPTIDEE---L-----------------------------------------------------TPEQLISLPKDDFR      59 Agambiae(XP_319583.4-AGAP008839-PA)/240-523              12.3%     KLKPELA-G---L-----------------------------------------------------TNEQIDKLPVDDYR      60 Dpulex(DAPPUDRAFT_68494-Predicted)/254-551               14.7%     DVRPVIEQPELQ------------------------------------------------------KK-AALSEDVKKYI      61 Dpulex(DAPPUDRAFT_309882)/300-599                        12.5%     EAREELEKDGDGG-----------------------------------------------------RL-LASARNPDSYS      62 Dmelanogaster(CG8378-NP_610730.1)/196-491                13.8%     EQEQNQD-----KCAFD--------------------------------------------------LNYNELTPEEHYR      63 Agambiae(XP_566179.1-AGAP000216-PA)/158-458              13.6%     NYLSRTE-----VNPLM--------------------------------------------------LNWVDGQK-IAFS      64 Agambiae(XP_564258.1-AGAP011234-PA)/216-546              16.4%     ETLDETK-----VNAFT--------------------------------------------------VDWNKATDRDIYD      65 Agambiae(XP_309407.4-AGAP011238-PA)/219-497              15.4%     NTLDETN-----VNAFT--------------------------------------------------MDWKNATVSDIYD      66 Agambiae(XP_314169.4-AGAP005253-PB)/218-514              16.2%     NALDETK-----VNAFT--------------------------------------------------VDWNKVTDSDIYD      67 Agambiae(XP_309409.4-AGAP011237-PA)/206-481              15.2%     NTLDEAK-----VNAFT--------------------------------------------------VDWNEITDSDMYD      68 Agambiae(XP_307865.2-AGAP009448-PA)/166-466              15.6%     DALDEPQ-----VNGFT--------------------------------------------------MDWRTATPKDVYS      69 Agambiae(XP_309762.4-AGAP010931-PA)/113-383              14.4%     DALDESK-----VDGFT--------------------------------------------------MDWKKATLQDVFN      70 Agambiae(XP_309378.2-AGAP011267-PA)/149-447              16.9%     DVLDESN-----VNGFT--------------------------------------------------MDWNKATQQDIFN      71 Agambiae(XP_309383.4-AGAP011257-PA)/149-447              16.4%     DALDESN-----VNGFT--------------------------------------------------MDWNKATQQDIFN      72 Agambiae(XP_307655.3-AGAP012638-PA)/149-447              16.4%     DALDESN-----VNGFT--------------------------------------------------MDWNKATQQDIFN      73 Agambiae(XP_320681.4-AGAP011835-PA)/183-484              14.6%     QVLEKAK-----VNAFT--------------------------------------------------MDWTSASAKDIYD      74 Agambiae(XP_309411.4-AGAP011232-PA)/162-434              14.4%     SHLDVSK-----VNAFE--------------------------------------------------MDWRAASPRTVYE      75 Amellifera(XP_001120776.2-SMYD4-like-Predicted)/251-554  13.4%     ELRADMKLAESNPDNRT------------------------------------------------AGFTDEDILDSTSAR      76 Dpulex(DAPPUDRAFT_305694-Predicted)/258-553              16.3%     DVASEDD----GCN-----------------------------------------------------PAGGELYDSTDYG      77 Dpulex(EFX87901.1)/258-554                               14.2%     IANEQEK----SPE-----------------------------------------------------SFASQVFDSANYD      78 Amellifera(XP_001122116.2-SMYD4-like-Predicted)/234-534  13.5%     ELRKELEEVDSCEDPRT------------------------------------------------KGFSKNGMFLSDKYR      79 Hmagnipapillata(XP_002159692.1)/239-485                  13.7%     SFKENNS------------------------------------------------------------FDDLKPYSSTDYN      80 Nvectensis(XP_001623892.1)/215-512                       13.9%     ASVKQFESEEKKCDPAE------------------------------------------------LGCNPDGVYDPSDYR      81 Lgigantea(LOTGIDRAFT_143433)/100-395                     15.6%     QNNKKYP-ESFRSDILR------------------------------------------------IGFNKDGVYDSMDYD      82 Skowalevskii(XP_002740933.1)/253-549                     16.3%     KFREQSA-NVNIPDESF------------------------------------------------HGCTVDGEYES-NYY      83 Dpulex(EFX73755.1)/49-306                                15.8%     L----------------------------------------------------------------------LSNFQQKSL      84 Amellifera(XP_625013.1-SMYD3-Predicted)/1-253            15.0%     E----------------------------------------------------------------------IGYYSKTKY      85 Dmelanogaster(Buzidau-CG13761)/26-282                    14.4%     I----------------------------------------------------------------------RGYYTEHGS      86 Agambiae(XP_319707.4-AGAP008954-PA)/1-254                16.7%     H----------------------------------------------------------------------KGYYTSKQY      87 cintestinalis(NP_001071820.1)/15-282                     18.6%     E-----------------------------------------------------------------KKDGKENDEKKVEL      88 Drerio(Q6P0R5-Smyd1a)/18-279                             15.6%     ----------------------------------------------------------------------------DSQL      89 Derio(Q2MJQ9-Smyd1b)/13-274                              15.9%     ----------------------------------------------------------------------------DNQL      90 Xtropicalis(NP_001120357.1-SMYD1)/13-261                 16.3%     ----------------------------------------------------------------------------EGCL      91 Hsapiens(Q8NB12-SMYD1)/18-279                            16.1%     ----------------------------------------------------------------------------EGCL      92 Ggallus(NP_989486.1-SMYD1)/13-274                        16.4%     ----------------------------------------------------------------------------ENCL      93 Drerio(Q5RGL7-Smyd2b)/19-268                             15.0%     ----------------------------------------------------------------------------SEIL      94 Drerio(Q5BJI7-Smyd2a)/18-267                             15.2%     ----------------------------------------------------------------------------SERV      95 Xtropicalis(XP_002934751.2-SMYD2-like-Predicted)/16-265  15.2%     ----------------------------------------------------------------------------SERF      96 Hsapiens(Q9NRG4-SMYD2)/18-267                            16.1%     ----------------------------------------------------------------------------SEKL      97 Ggallus(XP_419420.1-SMYD2-Predicted)/21-270              14.7%     ----------------------------------------------------------------------------SEKL      98 cintestinalis(XP_002128556.1)/14-266                     18.0%     ----------------------------------------------------------------------------LPEK      99 Lgigantea(LOTGIDRAFT_177746)/1-216                       15.4%     --------------------------------------------------------------------------------     100 Drerio(E7EZZ6-SMYD3)/16-267                              18.8%     ----------------------------------------------------------------------------QEEL     101 Xtropicalis(XP_004914684.1|-SMYD3-Predicted)/15-264      18.0%     ----------------------------------------------------------------------------SEEL     102 Hsapiens(Q9H7B4-SMYD3)/15-266                            17.4%     ----------------------------------------------------------------------------SEKL     103 Ggallus(XP_419536.1-SMYD3-Predicted)/15-266              18.0%     ----------------------------------------------------------------------------SERL     104 Tadhaerens(XP_002109888.1)/20-262                        19.2%     ------------------------------------------------------------------------------RA     105 Hmagnipapillata(XP_002163555.2)/16-259                   17.2%     -------------------------------------------------------------------------------N     106 Nvectensis(XP_001627600.1)/17-253                        18.2%     -------------------------------------------------------------------------------N     107 Bfloridae(XP_002594889.1-BRAFLDRAFT_124463)/14-258       18.1%     ----------------------------------------------------------------------------VP-G     108 Skowalevskii(XP_006817727.1)/14-260                      15.8%     ----------------------------------------------------------------------------SQTI         consensus/100%                                                     ................................................................................         consensus/90%                                                      .................                                            ...................         consensus/80%                                                      .........   ... .                                                 ..............         consensus/70%                                                      .......     .                                                      .............                                                                         321          .         .         :         .         .         .         .         4 400   1 Tadhaerens(XP_002114620.1)/25-373                       100.0%     ---FSQFS-RVTANNE--AHVAH-KLFG---KQFVEQIEILRLELINTLP------------------------------       2 Bfloridae(XP_002609030.1-BRAFLDRAFT_84846)/1-276         31.0%     ---FPQCC-AVKL--------EQ-HVTC---PHCQDQLELLRGLLTEALY------------------------------       3 cintestinalis(XP_002127168.1)/13-358                     39.9%     ---IEKFC-HASTNDV--EQIAH-KLLG---EQFLVQLTTLREQLASVFF------------------------------       4 Dpulex(EFX89935.1)/23-367                                41.0%     ---LMSLC-HHTVNEE--ETIAH-KLLG---QEFESQLELLRDLCTKALG------------------------------       5 Dmelanogaster(CG3353-NP_650955.1)/13-363                 39.1%     ---LQSFQ-SLIVNRE--QKIYH-KMLG---ENFEQQMEQLYLAFCNAFT------------------------------       6 Hmagnipapillata(XP_002163562.2)/21-371                   44.2%     ---FQQFF-SKTKNEE--HKITH-KLLG---NKFKEQIILMHSLLQEIVP------------------------------       7 Agambiae(XP_313299.1-AGAP003552-PA)/13-365               41.4%     ---LQDFV-HKSVNED--LLIFH-KMLG---EKFTQQIEQLYELFCKAFQ------------------------------       8 Amellifera(XP_394075.2-SMYD5-like-Prediction)/16-364     45.0%     ---FSQFC-HRTVNDT--HEIAH-KLLG---EKFVGQIDVLRQMMQKTIN------------------------------       9 Nvectensis(XP_001627062.1)/18-370                        48.0%     ---FSHFC-SNVVNKE--QQIAH-KLLG---LHFQEQLDMIRILLSEAMY------------------------------      10 Skowalevskii(XP_002735533.1)/24-372                      50.1%     ---FSQFR-RATVNEE--ADITH-KLLG---EEFQGDIDMLLPFLNEALK------------------------------      11 Lgigantea(LOTGIDRAFT_231752)/19-367                      51.0%     ---FNKFV-NNTVNEE--EQIAH-KLLG---DQFKCQLELLRSTTAEILF------------------------------      12 Drerio(F1RET2-Smyd5)/32-380                              49.9%     ---FTNFC-SRTANEE--EEIVH-KLLG---EKFQGQLGLLRNLFTTALY------------------------------      13 Ggallus(NP_001012912.1-SMYD5)/39-387                     50.1%     ---FSQFC-SKTANEE--EEIAH-KLLG---DKFKGQLELLRLLFTEALY------------------------------      14 Hsapiens(Q6GMV2-SMYD5)/33-381                            49.9%     ---FSQFC-NKTANEE--EEIVH-KLLG---DKFKGQLELLRRLFTEALY------------------------------      15 Xtropicalis(A9ULL8-SMyd5)/32-382                         51.0%     ---FSQFC-NKTANEE--EEIVH-KLLG---DKFKGQLDQLRRLFTDALY------------------------------      16 Lgigantea(LOTGIDRAFT_232186)/323-670                     20.5%     ---VTHWA-KA-------KAPYR-RFIAYGTTSAISRMPHMLPVFRRVFK------------------------------      17 Bfloridae(XP_002589246.1-BRAFLDRAFT_74594)/380-720       22.0%     ---KDQWA-RA-------KEPYR-RFLGFGVSGFVKQIPKMLKIMQAIFQ------------------------------      18 Amellifera(XP_006565332.1)/43-285                        14.2%     -C-LATLQ-Y---NKN--------LFSKY---EIELLKKNVMNSPSD---------------------------------      19 Dmelanogaster(msta-CG33548)/66-313                       14.8%     -L-VDAMQ-A---NAE--------RAYRR---EIIQAAQCFRNFPTT---------------------------------      20 Dmelanogaster(CG12119)/34-280                            15.8%     -L-IYCLQ-A---NLD--------NNHRT---EVRNAAKCFKNFPTD---------------------------------      21 Amellifera(XP_006565301.1)/26-284                        15.8%     -R-VLSLE-S---HLN--KRRNT-IVWED---REINIVNVLKSLNF----------------------------------      22 Dmelanogaster(CG9642)/21-271                             13.6%     -E-MSQMD-S---QLM--TRRGT-EVWKN---YQEHAFTPLDYGGVLAQ-------------------------------      23 Dmelanogaster(CG9640)/17-268                             12.9%     -M-LIDVPIN---LSD--YRDGE-GMWQE---HEELVVRPLMESGLADV-------------------------------      24 Amellifera(NP_001229486.1-LOC724300)/57-301              12.7%     -S-IQKLQ-S---HED--SRGPGTSVYEE----TMNIYYHIQRLLPDN--------------------------------      25 Dmelanogaster(CG14590-NP_610202.3)/55-322                14.9%     -A-LLEMQ-S---HEE--ERKGT-DLYEE---AEKRVVTYLQKRFLCRL-------------------------------      26 Dmelanogaster(CG43129)/21-279                            11.6%     -NEVAPME-H---HKE--ERQRDADVWHA---DRVNIAQYLRGPCQLA--------------------------------      27 Dmelanogaster(G11160)/58-319                             13.9%     -L-IARME-S---HTE--ERRQNAVLWRH---YEEKVVQRLRVTWQLE--------------------------------      28 Amellifera(XP_624539.3-msta-like-Predicted)/54-297       12.8%     -K-LISLE-S---HCN--EMNNS-K-------EPLNIAHFIKRFFKAD--------------------------------      29 Dmelanogaster(CG8503-NP_610944.1)/52-301                 13.4%     -K-FQDLE-S---LES--TRRGS-NQWKA---DLVSIGQFIPKFFKTQ--------------------------------      30 Agambiae(XP_309979.4-AGAP011530-PA)/50-300               14.8%     -T-LLKLE-S---HEE--ERRGS-EQWRN---DREGVAKLIPRFFKCE--------------------------------      31 Dpulex(DAPPUDRAFT_120473)/58-292                         14.4%     -E-YKMLE-N---HND--LRRQS-DMWRI---YQVNVVQFLRKICGLA--------------------------------      32 Dpulex(DAPPUDRAFT_194440-Predicted)/53-302               11.7%     -E-LMQLE-A---HVQ--ERRQK-GMEDV---DQATAVRFIRETLGLQ--------------------------------      33 Dpulex(DAPPUDRAFT_2393)/50-297                           16.0%     ----DELV-S---HLE--QRRGM-DIYRL---VEQNISSFLRYRLLLT--------------------------------      34 Dmelanogaster(CG18136-NP_649084.1)/58-318                15.9%     -K-LYNLE-D---HLK--ERLET-PLYQV---LRANLITFIKTVLGMK--------------------------------      35 Agambiae(XP_309220.5-AGAP001025-PA)/55-318               16.2%     -T-VQGFE-S---HLD--ERLAS-PLYGV---LRSNLVPFLRQVLRLQ--------------------------------      36 Dmelanogaster(CG1868-NP_724802.1)/226-549                13.1%     -R-SLRMV-S---QLD--QAIDE-ELNYH---ILCAN--LLQLYLKEHTDFYDQF----------------HSL------      37 Agambiae(XP_319721.4-AGAP008973-PA)/165-486              13.3%     -R-VLRLV-T---NFD--KMDPD-DRMRY---TLAGL--MLTIYLQECTPFAEAV----------------KDY------      38 Bfloridae(XP_002593048.1-BRAFLDRAFT_74375)/6-196         11.1%     -P-LCDLC-P---HTKELK----------------------------------------------DSS----ELRIQ---      39 Bfloridae(XP_002594298.1-BRAFLDRAFT_117670)/15-265       13.8%     -P-LSSLV-S---NVETLK----------------------------------------------NCEEGITSLDSK---      40 Cintestinalis(XP_002123001.1)/195-567                    11.0%     -S-IFCLK-TSSCVEKNSDFKND-CSWFC---MLVST--IRSEVFGEEE---NRA------------KDDVASLKEL---      41 Drerio(Q08C84-Smyd4)/197-556                             11.1%     -G-IYSLL-P---HVA--QHSPA-SRFLM---AITMA--VIYGKLQGGP-PPNK----------------------W---      42 Xtropicalis(NP_001072288.1-SMYD4)/212-545                12.2%     -S-VVNLL-P---HTE--NHPAE-RKFLC---GLTAA--ALYKKLCLIM-AKDLV------------SST--SQTEK---      43 Hsapiens(Q8IYR2-SMYD4)/244-602                           11.9%     -A-VFNLL-P---HTE--NHSPE-HKFLC---ALCVS--ALCRQLEAAS-LQAIP------------TERIVN-------      44 Ggallus(NP_001025886.1-SMYD4)/241-573                    11.1%     -A-VFNLL-P---HVE--KHSPE-HKFLC---MLSIV--AICKKLQETG-LEAAV------------LNGESSTTGS---      45 Hmagnipapillata(XP_002160254.2/232-532                   13.1%     -S-VYFLS-T---HSD--RLPIE-DLFQY---SVAGF--LLYKLLINSS-FFKTHTVL----------------------      46 Dpulex(DAPPUDRAFT_312722-Pedicted)/241-525               14.2%     -V-MFHLV-S---HTE--RMAPE-ELYQY---ALTAA--FLTLLLEQHSSFFQSA-------------------------      47 Amellifera(XP_006565387.1-SMYD4-like-Predicted)/278-571  16.8%     ------LI-T---NFD--KLSID-DLTIY---GITAI--MLTIYLFKYTNFFKTN--------------N----------      48 Bfloridae(XP_002589088.1-BRAFLDRAFT_75068)/251-714       10.4%     -K-TVQID-SETGSTS--EQPGD-----L---SVQTD--VIEENPPSAG-MESPTTADKPSDQNKLCTDDLSSTSEEAKT      49 Lgigantea(LOTGIDRAFT_169490)/248-638                     11.5%     -T-VYYLM-T---HDN--DILTE-DMYQY---SGTAA--LLLIILVHSG-WFNTNVTQI----ATHIDSTLQADLQSV--      50 Skowalevskii(XP_002733823.1)/75-447                      10.9%     -S-IYSLI-T---HSG--KQPWK-DVFFF---TLTSI--LLSTLVT-KL-ISPSD----------DVD-DLLADTEAMKM      51 Nvectensis(XP_001627273.1)/170-547                       12.5%     -S-VFSLV-T---NSD--LQPIK-ALMSF---AMNSA--FLVEFLE-NG-TSSAC---------IHCS-QIKSDKT--KV      52 Amellifera(XP_003250668.1-SMYD4-like-Predicted)/183-473  12.1%     -S-ILNLS-IPMKKDD--NRTED-NLFYS---AKIAM--LLRNH---SNYMQGS--------------------------      53 Amellifera(XP_001121272.2-SMYD4-like-Predicted)/230-549  13.9%     -T-ILNLE-T---HCT--KMEPK-TNLIR---AIEAI--FLAKCFTFVLSKMDVVY------------------------      54 Amellifera(XP_003249162.1-SMYD4-like-Predicted)/239-589  11.5%     -R-VYDLV-T---HEK--RRTAK-DFFER---SLMAA--FLFKCLQKVGF-FDNPS------------------SN----      55 Dmelanogaster(CG14122-NP_648574.1)/265-541               14.2%     -L-FEHLC-S---HEE--DRQPD-DYLRR---ALMSG--FLLRILQKSLY-FGRRK------------------TE----      56 Agambiae(XP_311885.3-AGAP002999-PA)/268-544              13.9%     -L-RDTFC-A---HTE--HRDPE-DHFKR---TLMTA--FLLRCLQKAEF-FGRRT------------------TE----      57 Amellifera(XP_392262.3-SMYD4-like-Predicted)/252-555     15.0%     -I-MFRLV-T---HED--TRTVE-DLFHR---TYIAS--WLLRLLKKGPY-FPKHVKT--------------PDTI----      58 Dmelanogaster(CG7759-NP_725048.1)/250-537                15.6%     -R-VAQLE-R---HQG--ERQPS-NFFQH---VLMAR--FLTNCLRAGGY-FGSEPK-----------------------      59 Agambiae(XP_319583.4-AGAP008839-PA)/240-523              12.3%     -K-VYKLV-T---HES--TRSPE-DFFQR---TLMAT--LLNACLTLGGY-GA---C-----------------------      60 Dpulex(DAPPUDRAFT_68494-Predicted)/254-551               14.7%     -K-TYHLV-T---HDT--LRNKE-SFFHV---TLMAN--FLLKCLKVAGY-FGTRD------------------TT----      61 Dpulex(DAPPUDRAFT_309882)/300-599                        12.5%     -G-IYHLA-T---LSH--LRSDK-DFFDR---TFMAL--FLFQCLRASGY-LQTRFR---------------YEED----      62 Dmelanogaster(CG8378-NP_610730.1)/196-491                13.8%     -A-IHGLV-T---NQH--LRSVS-DLFQR---SVVCA--VLKHFIIEYTPV-KE-YL-----------------------      63 Agambiae(XP_566179.1-AGAP000216-PA)/158-458              13.6%     -A-VYILA-S---LAR--APNDP-VEARV---AQISR--EMHCHLVSENGQ-TANDD-----------------------      64 Agambiae(XP_564258.1-AGAP011234-PA)/216-546              16.4%     -T-VHVLA-T---NQN--RRDHK-QLASL---IFFAY--IVQGLLLDRTE-LRPLCW-----------------------      65 Agambiae(XP_309407.4-AGAP011238-PA)/219-497              15.4%     -T-VHVLA-T---NQK--RRSRK-DLAEL---IFFAS--IVHRLLLERTD-FGPLCE-----------------------      66 Agambiae(XP_314169.4-AGAP005253-PB)/218-514              16.2%     -T-VHVLA-T---NQK--RRSRE-DLAVL---MFFTS--IVHRLLLERTD-LGPFCE-----------------------      67 Agambiae(XP_309409.4-AGAP011237-PA)/206-481              15.2%     -T-VHVLA-T---NQK--RRSCK-DLAML---IFFAS--IVHRLLLERTE-LGTLCE-----------------------      68 Agambiae(XP_307865.2-AGAP009448-PA)/166-466              15.6%     -T-VHVLS-T---NQE--RRPFM-QLVFM---VYLAI--IIHKLMLERTE-LGPRSR-----------------------      69 Agambiae(XP_309762.4-AGAP010931-PA)/113-383              14.4%     -T-VHVLC-T---NQE--RRNIK-ELAGL---TFFTV--VMHNHLLEWTE-LGPACE-----------------------      70 Agambiae(XP_309378.2-AGAP011267-PA)/149-447              16.9%     -T-VHVLT-T---NQE--RRDSN-FLAFH---IFNAT--ILHTLVLERTE-LGPVCE-----------------------      71 Agambiae(XP_309383.4-AGAP011257-PA)/149-447              16.4%     -T-VHVLT-T---NQE--RRHSM-FVAMF---IFNAT--ILHTLILERTE-LGPVCE-----------------------      72 Agambiae(XP_307655.3-AGAP012638-PA)/149-447              16.4%     -T-VHVLT-T---NQE--RRDSF-FVAFY---IFNAT--ILHTLVLERTE-LGPVCE-----------------------      73 Agambiae(XP_320681.4-AGAP011835-PA)/183-484              14.6%     -T-VHVLE-T---NER--TRDRK-DRMVR---VFYTT--IIYRLLEERCPELGELCA-----------------------      74 Agambiae(XP_309411.4-AGAP011232-PA)/162-434              14.4%     -T-VYSLA-T---NQR--KRARK-DFALN---VLVAM--ITHKLLLKRTPA-AQVCG-----------------------      75 Amellifera(XP_001120776.2-SMYD4-like-Predicted)/251-554  13.4%     -S-ALSLA-T---NMT--MRPLI-GISAF---ACISA--LAAILLATQTNFFCNKYEV--------------NQLK----      76 Dpulex(DAPPUDRAFT_305694-Predicted)/258-553              16.3%     RT-IHRLV-G---NTA--RRSVA-DLFRR---AVMAV--YLTSLIQQQRD------------------------------      77 Dpulex(EFX87901.1)/258-554                               14.2%     -T-VHRLV-D---NSS--QRSTT-DIFRR---AVMAV--YLTSLIQIRD-------------------------------      78 Amellifera(XP_001122116.2-SMYD4-like-Predicted)/234-534  13.5%     -S-LLGLI-T---NTE--KRSVQ-DLFRR---SLDAS--FILYFLATCSNMFGNPLKK--------------DLSV----      79 Hmagnipapillata(XP_002159692.1)/239-485                  13.7%     -S-IFSLI-E---N------------------------------------------------------------------      80 Nvectensis(XP_001623892.1)/215-512                       13.9%     -P-IYHLV-G---HTH--ERTLN-DLFVR---TLNAI--YLLRCLEGT------EYYG--------------DSTK----      81 Lgigantea(LOTGIDRAFT_143433)/100-395                     15.6%     -T-VYSLV-K---HSE--KRSLG-DLFKR---SVVAV--FMVKCLEHT------LSSQ--------------PLST----      82 Skowalevskii(XP_002740933.1)/253-549                     16.3%     -S-VYNLV-G---HSE--DRKPG-DLFKR---VVKAV--CLLRCLQQT------NFFQ--------------SVGA----      83 Dpulex(EFX73755.1)/49-306                                15.8%     NN-YREEV-I---YRK--EIKEDTKRME----YFMTICGVLNEYLSNE--------------------------------      84 Amellifera(XP_625013.1-SMYD3-Predicted)/1-253            15.0%     RK-FKDLM-S---HYS--DIKKDEKKME----HFVCVCGVLYEFLGDM--------------------------------      85 Dmelanogaster(Buzidau-CG13761)/26-282                    14.4%     RK-FRDLM-S---HYA--EIKNDPMRLE----HLDSLHAVLTDMMAESP-------------------------------      86 Agambiae(XP_319707.4-AGAP008954-PA)/1-254                16.7%     RK-FCDLM-P---HEE--NIRADSKRME----HFGTLYVVLQRLLDEA--------------------------------      87 cintestinalis(NP_001071820.1)/15-282                     18.6%     VK-IEELE-D---HLS--KRNAEEK--E----AIDEKVYSFGDYFTYDEM------------------------------      88 Drerio(Q6P0R5-Smyd1a)/18-279                             15.6%     TT-LDMLE-D---HLS--RMTPEDL--K----ELKADVKTFYTYWPKKSK------------------------------      89 Derio(Q2MJQ9-Smyd1b)/13-274                              15.9%     TT-LEDLE-D---HIC--DISEDDL--K----DFKVDIHNFLDYWPRNSK------------------------------      90 Xtropicalis(NP_001120357.1-SMYD1)/13-261                 16.3%     VS-IDDLQ-N---HID--KFDEAEK--G----LLMEDVQKFLEYWPSQSQ------------------------------      91 Hsapiens(Q8NB12-SMYD1)/18-279                            16.1%     VS-VDDLQ-N---HVE--HFGEEEQ--K----DLRVDVDTFLQYWPPQSQ------------------------------      92 Ggallus(NP_989486.1-SMYD1)/13-274                        16.4%     VS-IDDLQ-N---HVE--SFDEEEK--K----DLRVDVESFLEFWPAQSQ------------------------------      93 Drerio(Q5RGL7-Smyd2b)/19-268                             15.0%     LL-LGEME-A---HLE--DMDNEKR--E----MTEAHIAGLHQFYSKHL-------------------------------      94 Drerio(Q5BJI7-Smyd2a)/18-267                             15.2%     LT-LRELE-A---HLD--KLDNEKN--E----MNDTDIAALHHFYSRHL-------------------------------      95 Xtropicalis(XP_002934751.2-SMYD2-like-Predicted)/16-265  15.2%     LS-VKDFE-S---HLS--KLDNEKL--E----LIQNDIAALHRFYSKNL-------------------------------      96 Hsapiens(Q9NRG4-SMYD2)/18-267                            16.1%     LA-VKEFE-S---HLD--KLDNEKK--D----LIQSDIAALHHFYSKHL-------------------------------      97 Ggallus(XP_419420.1-SMYD2-Predicted)/21-270              14.7%     LA-VKEFE-S---HLD--KLDNEKR--E----LIQNDIAALHHFYSKHM-------------------------------      98 cintestinalis(XP_002128556.1)/14-266                     18.0%     NS-ILDLE-S---NYN--KLSQNQK--E----ALMNFLVILHTFWSP---------------------------------      99 Lgigantea(LOTGIDRAFT_177746)/1-216                       15.4%     ---------------------------------------MMLSTVTELTK------------------------------     100 Drerio(E7EZZ6-SMYD3)/16-267                              18.8%     YS-IAEHQ-S---HLA--DMSEEKK--E----GLKHLCTTLQVYLAEENC------------------------------     101 Xtropicalis(XP_004914684.1|-SMYD3-Predicted)/15-264      18.0%     YT-ISDLQ-S---HIK--EASEEVK--D----GLRHLATALQHYLKEEIQ------------------------------     102 Hsapiens(Q9H7B4-SMYD3)/15-266                            17.4%     YS-FYDLE-S---NIN--KLTEDKK--E----GLRQLVMTFQHFMREEIQ------------------------------     103 Ggallus(XP_419536.1-SMYD3-Predicted)/15-266              18.0%     YS-FKDLQ-S---NAE--QLSEEMK--E----GLGHLAHTLQLYLRAEIQ------------------------------     104 Tadhaerens(XP_002109888.1)/20-262                        19.2%     DQ-FKFLL-S---NRE--LLEGSRK--N----TIVDGINLLKEYLSNKV-------------------------------     105 Hmagnipapillata(XP_002163555.2)/16-259                   17.2%     TL-INNLY-A---NKG--NISNARK--E----AFFTFAAVLVEYLQDVN-------------------------------     106 Nvectensis(XP_001627600.1)/17-253                        18.2%     GW-YDSLV-S---NVE--KIDSDAK--E----DFVSVLMVLNEYLGSEI-------------------------------     107 Bfloridae(XP_002594889.1-BRAFLDRAFT_124463)/14-258       18.1%     NS-IDELQ-S---NLR--EMPENVK--E----MFAQLAVVLRMYVGKDV-------------------------------     108 Skowalevskii(XP_006817727.1)/14-260                      15.8%     TE-FMSLQ-S---HDT--ALTPEKK--E----QFSQLLFVLNQYVDEGT-------------------------------         consensus/100%                                                     ................................................................................         consensus/90%                                                      .. h..h. t...p.t  ...........  .........h..hh............               ....             consensus/80%                                                      .. h.th. s   p.p  p...p.t....   .h.t...hh..hh..... ......                                consensus/70%                                                       . h.pl. s   p.p  ph..p p.ht.   .h.s...hlhphhttt.. ....                                                                                                  401          .         .         .         .         :         .         .         . 480   1 Tadhaerens(XP_002114620.1)/25-373                       100.0%     --------------------------------------------------------------------------------       2 Bfloridae(XP_002609030.1-BRAFLDRAFT_84846)/1-276         31.0%     --------------------------------------------------------------------------------       3 cintestinalis(XP_002127168.1)/13-358                     39.9%     --------------------------------------------------------------------------------       4 Dpulex(EFX89935.1)/23-367                                41.0%     --------------------------------------------------------------------------------       5 Dmelanogaster(CG3353-NP_650955.1)/13-363                 39.1%     --------------------------------------------------------------------------------       6 Hmagnipapillata(XP_002163562.2)/21-371                   44.2%     --------------------------------------------------------------------------------       7 Agambiae(XP_313299.1-AGAP003552-PA)/13-365               41.4%     --------------------------------------------------------------------------------       8 Amellifera(XP_394075.2-SMYD5-like-Prediction)/16-364     45.0%     --------------------------------------------------------------------------------       9 Nvectensis(XP_001627062.1)/18-370                        48.0%     --------------------------------------------------------------------------------      10 Skowalevskii(XP_002735533.1)/24-372                      50.1%     --------------------------------------------------------------------------------      11 Lgigantea(LOTGIDRAFT_231752)/19-367                      51.0%     --------------------------------------------------------------------------------      12 Drerio(F1RET2-Smyd5)/32-380                              49.9%     --------------------------------------------------------------------------------      13 Ggallus(NP_001012912.1-SMYD5)/39-387                     50.1%     --------------------------------------------------------------------------------      14 Hsapiens(Q6GMV2-SMYD5)/33-381                            49.9%     --------------------------------------------------------------------------------      15 Xtropicalis(A9ULL8-SMyd5)/32-382                         51.0%     --------------------------------------------------------------------------------      16 Lgigantea(LOTGIDRAFT_232186)/323-670                     20.5%     --------------------------------------------------------------------------------      17 Bfloridae(XP_002589246.1-BRAFLDRAFT_74594)/380-720       22.0%     --------------------------------------------------------------------------------      18 Amellifera(XP_006565332.1)/43-285                        14.2%     --------------------------------------------------------------------------------      19 Dmelanogaster(msta-CG33548)/66-313                       14.8%     --------------------------------------------------------------------------------      20 Dmelanogaster(CG12119)/34-280                            15.8%     --------------------------------------------------------------------------------      21 Amellifera(XP_006565301.1)/26-284                        15.8%     ------------------------------------------L-------------------------------------      22 Dmelanogaster(CG9642)/21-271                             13.6%     ------------------------------------------L-------------------------------------      23 Dmelanogaster(CG9640)/17-268                             12.9%     ------------------------------------------L-------------------------------------      24 Amellifera(NP_001229486.1-LOC724300)/57-301              12.7%     --------------------------------------------------------------------------------      25 Dmelanogaster(CG14590-NP_610202.3)/55-322                14.9%     ------------------------------------------K-------------------------------------      26 Dmelanogaster(CG43129)/21-279                            11.6%     --------------------------------------------------------------------------------      27 Dmelanogaster(G11160)/58-319                             13.9%     --------------------------------------------------------------------------------      28 Amellifera(XP_624539.3-msta-like-Predicted)/54-297       12.8%     --------------------------------------------------------------------------------      29 Dmelanogaster(CG8503-NP_610944.1)/52-301                 13.4%     --------------------------------------------------------------------------------      30 Agambiae(XP_309979.4-AGAP011530-PA)/50-300               14.8%     --------------------------------------------------------------------------------      31 Dpulex(DAPPUDRAFT_120473)/58-292                         14.4%     --------------------------------------------------------------------------------      32 Dpulex(DAPPUDRAFT_194440-Predicted)/53-302               11.7%     --------------------------------------------------------------------------------      33 Dpulex(DAPPUDRAFT_2393)/50-297                           16.0%     --------------------------------------------------------------------------------      34 Dmelanogaster(CG18136-NP_649084.1)/58-318                15.9%     --------------------------------------------------------------------------------      35 Agambiae(XP_309220.5-AGAP001025-PA)/55-318               16.2%     --------------------------------------------------------------------------------      36 Dmelanogaster(CG1868-NP_724802.1)/226-549                13.1%     --------------------------------------------------------------------------------      37 Agambiae(XP_319721.4-AGAP008973-PA)/165-486              13.3%     --------------------------------------------------------------------------------      38 Bfloridae(XP_002593048.1-BRAFLDRAFT_74375)/6-196         11.1%     ---LE----KLS-----------------------RYVN-----------------------------------------      39 Bfloridae(XP_002594298.1-BRAFLDRAFT_117670)/15-265       13.8%     ---ME----CLS-----------------------QHME-----------------------------------------      40 Cintestinalis(XP_002123001.1)/195-567                    11.0%     ------------------------------LQEIFNSLNLKYLCENPNFDMDF--FRNRIK-TISE----M---------      41 Drerio(Q08C84-Smyd4)/197-556                             11.1%     -------------MSFK----D----------------------------------------------------------      42 Xtropicalis(NP_001072288.1-SMYD4)/212-545                12.2%     -------------SLTK----E----------------------------------------------------------      43 Hsapiens(Q8IYR2-SMYD4)/244-602                           11.9%     -------------SSQL----K----------------------------------------------------------      44 Ggallus(NP_001025886.1-SMYD4)/241-573                    11.1%     -------------EQKT----C----------------------------------------------------------      45 Hmagnipapillata(XP_002160254.2/232-532                   13.1%     --------------------------------------------------------------------------------      46 Dpulex(DAPPUDRAFT_312722-Pedicted)/241-525               14.2%     --------------------------------------------------------------------------------      47 Amellifera(XP_006565387.1-SMYD4-like-Predicted)/278-571  16.8%     ---LE------------------------------DSLM-KKF-LNNFFNLNFNILTN----------------------      48 Bfloridae(XP_002589088.1-BRAFLDRAFT_75068)/251-714       10.4%     DSETESTLEQASNMSQAESTPEQASNLSVKTDSVQNSMQDVEL-HRGNYSSVYNLMTHTEHHSVEQLLTQMMVSCLMCKC      49 Lgigantea(LOTGIDRAFT_169490)/248-638                     11.5%     ----E--IDDKGGTIYANEGKDADIANDS----KDNSLTNQKK-QNNSISTN--------EK------------------      50 Skowalevskii(XP_002733823.1)/75-447                      10.9%     TEAAE--AENKSNRSPSE--DESQINGSAPSRHTLDATDADRD-R-NTSQDN--------T-------------------      51 Nvectensis(XP_001627273.1)/170-547                       12.5%     QTELD--SDDDS--------DCSEV---------YNACEEQRT-QNGNFEQD--------R-------------------      52 Amellifera(XP_003250668.1-SMYD4-like-Predicted)/183-473  12.1%     --------------------------------------------------------------------------------      53 Amellifera(XP_001121272.2-SMYD4-like-Predicted)/230-549  13.9%     --------------------------------------------------------------------------------      54 Amellifera(XP_003249162.1-SMYD4-like-Predicted)/239-589  11.5%     --------------------------------------------------------------------------------      55 Dmelanogaster(CG14122-NP_648574.1)/265-541               14.2%     --------------------------------------------------------------------------------      56 Agambiae(XP_311885.3-AGAP002999-PA)/268-544              13.9%     --------------------------------------------------------------------------------      57 Amellifera(XP_392262.3-SMYD4-like-Predicted)/252-555     15.0%     --------------------------------------------------------------------------------      58 Dmelanogaster(CG7759-NP_725048.1)/250-537                15.6%     --------------------------------------------------------------------------------      59 Agambiae(XP_319583.4-AGAP008839-PA)/240-523              12.3%     --------------------------------------------------------------------------------      60 Dpulex(DAPPUDRAFT_68494-Predicted)/254-551               14.7%     --------------------------------------------------------------------------------      61 Dpulex(DAPPUDRAFT_309882)/300-599                        12.5%     --------------------------------------------------------------------------------      62 Dmelanogaster(CG8378-NP_610730.1)/196-491                13.8%     --------------------------------------------------------------------------------      63 Agambiae(XP_566179.1-AGAP000216-PA)/158-458              13.6%     --------------------------------------------------------------------------------      64 Agambiae(XP_564258.1-AGAP011234-PA)/216-546              16.4%     --------------------------------------------------------------------------------      65 Agambiae(XP_309407.4-AGAP011238-PA)/219-497              15.4%     --------------------------------------------------------------------------------      66 Agambiae(XP_314169.4-AGAP005253-PB)/218-514              16.2%     --------------------------------------------------------------------------------      67 Agambiae(XP_309409.4-AGAP011237-PA)/206-481              15.2%     --------------------------------------------------------------------------------      68 Agambiae(XP_307865.2-AGAP009448-PA)/166-466              15.6%     --------------------------------------------------------------------------------      69 Agambiae(XP_309762.4-AGAP010931-PA)/113-383              14.4%     --------------------------------------------------------------------------------      70 Agambiae(XP_309378.2-AGAP011267-PA)/149-447              16.9%     --------------------------------------------------------------------------------      71 Agambiae(XP_309383.4-AGAP011257-PA)/149-447              16.4%     --------------------------------------------------------------------------------      72 Agambiae(XP_307655.3-AGAP012638-PA)/149-447              16.4%     --------------------------------------------------------------------------------      73 Agambiae(XP_320681.4-AGAP011835-PA)/183-484              14.6%     --------------------------------------------------------------------------------      74 Agambiae(XP_309411.4-AGAP011232-PA)/162-434              14.4%     --------------------------------------------------------------------------------      75 Amellifera(XP_001120776.2-SMYD4-like-Predicted)/251-554  13.4%     --------------------------------------------------------------------------------      76 Dpulex(DAPPUDRAFT_305694-Predicted)/258-553              16.3%     --------------------------------------------------------------------------------      77 Dpulex(EFX87901.1)/258-554                               14.2%     --------------------------------------------------------------------------------      78 Amellifera(XP_001122116.2-SMYD4-like-Predicted)/234-534  13.5%     --------------------------------------------------------------------------------      79 Hmagnipapillata(XP_002159692.1)/239-485                  13.7%     --------------------------------------------------------------------------------      80 Nvectensis(XP_001623892.1)/215-512                       13.9%     --------------------------------------------------------------------------------      81 Lgigantea(LOTGIDRAFT_143433)/100-395                     15.6%     --------------------------------------------------------------------------------      82 Skowalevskii(XP_002740933.1)/253-549                     16.3%     --------------------------------------------------------------------------------      83 Dpulex(EFX73755.1)/49-306                                15.8%     --------------------------------------------------------------------------------      84 Amellifera(XP_625013.1-SMYD3-Predicted)/1-253            15.0%     --------------------------------------------------------------------------------      85 Dmelanogaster(Buzidau-CG13761)/26-282                    14.4%     --------------------------------------------------------------------------------      86 Agambiae(XP_319707.4-AGAP008954-PA)/1-254                16.7%     --------------------------------------------------------------------------------      87 cintestinalis(NP_001071820.1)/15-282                     18.6%     --------------------------------------------------------------------------------      88 Drerio(Q6P0R5-Smyd1a)/18-279                             15.6%     --------------------------------------------------------------------------------      89 Derio(Q2MJQ9-Smyd1b)/13-274                              15.9%     --------------------------------------------------------------------------------      90 Xtropicalis(NP_001120357.1-SMYD1)/13-261                 16.3%     --------------------------------------------------------------------------------      91 Hsapiens(Q8NB12-SMYD1)/18-279                            16.1%     --------------------------------------------------------------------------------      92 Ggallus(NP_989486.1-SMYD1)/13-274                        16.4%     --------------------------------------------------------------------------------      93 Drerio(Q5RGL7-Smyd2b)/19-268                             15.0%     --------------------------------------------------------------------------------      94 Drerio(Q5BJI7-Smyd2a)/18-267                             15.2%     --------------------------------------------------------------------------------      95 Xtropicalis(XP_002934751.2-SMYD2-like-Predicted)/16-265  15.2%     --------------------------------------------------------------------------------      96 Hsapiens(Q9NRG4-SMYD2)/18-267                            16.1%     --------------------------------------------------------------------------------      97 Ggallus(XP_419420.1-SMYD2-Predicted)/21-270              14.7%     --------------------------------------------------------------------------------      98 cintestinalis(XP_002128556.1)/14-266                     18.0%     --------------------------------------------------------------------------------      99 Lgigantea(LOTGIDRAFT_177746)/1-216                       15.4%     --------------------------------------------------------------------------------     100 Drerio(E7EZZ6-SMYD3)/16-267                              18.8%     --------------------------------------------------------------------------------     101 Xtropicalis(XP_004914684.1|-SMYD3-Predicted)/15-264      18.0%     --------------------------------------------------------------------------------     102 Hsapiens(Q9H7B4-SMYD3)/15-266                            17.4%     --------------------------------------------------------------------------------     103 Ggallus(XP_419536.1-SMYD3-Predicted)/15-266              18.0%     --------------------------------------------------------------------------------     104 Tadhaerens(XP_002109888.1)/20-262                        19.2%     --------------------------------------------------------------------------------     105 Hmagnipapillata(XP_002163555.2)/16-259                   17.2%     --------------------------------------------------------------------------------     106 Nvectensis(XP_001627600.1)/17-253                        18.2%     --------------------------------------------------------------------------------     107 Bfloridae(XP_002594889.1-BRAFLDRAFT_124463)/14-258       18.1%     --------------------------------------------------------------------------------     108 Skowalevskii(XP_006817727.1)/14-260                      15.8%     --------------------------------------------------------------------------------         consensus/100%                                                     ................................................................................         consensus/90%                                                                                                                                               consensus/80%                                                                                                                                               consensus/70%                                                                                                                                                                                                               481          .         5         .         .         .         .         :         . 560   1 Tadhaerens(XP_002114620.1)/25-373                       100.0%     ------------------------------------------SSK---ITEWYTPGGFRSLLAMIGTNGQGIASS-SFSQ       2 Bfloridae(XP_002609030.1-BRAFLDRAFT_84846)/1-276         31.0%     ------------------------------------------EES---LDQWFTPDGFRSIFAMIGRNGQGIGTS-SLSV       3 cintestinalis(XP_002127168.1)/13-358                     39.9%     ------------------------------------------DES---VQHWFTDDGFKNLFALLGTNQQGVGTS-ALSV       4 Dpulex(EFX89935.1)/23-367                                41.0%     ------------------------------------------MPE---THEFLTPQGFRSLIALIGRNGQGIGTS-AFSV       5 Dmelanogaster(CG3353-NP_650955.1)/13-363                 39.1%     ------------------------------------------GEE---FSIFKTPDAFKTLMAILGTNSQGIATS-VLSQ       6 Hmagnipapillata(XP_002163562.2)/21-371                   44.2%     ------------------------------------------TGE---LSKLLTISGVQSLFALIGMNGQGIGTS-SLSE       7 Agambiae(XP_313299.1-AGAP003552-PA)/13-365               41.4%     ------------------------------------------VES-DERLSWLTPEGFKSLVALVGTNGQGIGTS-SFGD       8 Amellifera(XP_394075.2-SMYD5-like-Prediction)/16-364     45.0%     ------------------------------------------IEF---IEHWFTPEGFKSLLALVGTNGQGIGTS-AFSR       9 Nvectensis(XP_001627062.1)/18-370                        48.0%     ------------------------------------------DDR---LEQWFTPEGFSSLFALVGTNGQGIGTS-SLSL      10 Skowalevskii(XP_002735533.1)/24-372                      50.1%     ------------------------------------------EET---VSHWFTSEGIRSLFALIGTNGQGVGTS-SLSV      11 Lgigantea(LOTGIDRAFT_231752)/19-367                      51.0%     ------------------------------------------DES---IPQWFTPEGFQSLFALIGTNGQGIGSC-SISV      12 Drerio(F1RET2-Smyd5)/32-380                              49.9%     ------------------------------------------EDR---LSQWFTPEGFRSLFSLVGTNGQGIGTS-SLSQ      13 Ggallus(NP_001012912.1-SMYD5)/39-387                     50.1%     ------------------------------------------DEQ---LSRWFTPEGFRSLFALVGTNGQGIGTS-SLSQ      14 Hsapiens(Q6GMV2-SMYD5)/33-381                            49.9%     ------------------------------------------EEA---VSQWFTPDGFRSLFALVGTNGQGIGTS-SLSQ      15 Xtropicalis(A9ULL8-SMyd5)/32-382                         51.0%     ------------------------------------------EER---VSRWFTPEGFRSLFALVGTNGQGIGTS-SLSQ      16 Lgigantea(LOTGIDRAFT_232186)/323-670                     20.5%     ------------------------------------------NSGHEGVVFNVTEEEFNGRYYQATCNLQEFSAR-STP-      17 Bfloridae(XP_002589246.1-BRAFLDRAFT_74594)/380-720       22.0%     ------------------------------------------NTE---IKYKIDELEFERRYYQVACNVQSFGPP-CVT-      18 Amellifera(XP_006565332.1)/43-285                        14.2%     -------------------------------------------------ED--I-EMMERVCRAFNTNSFETICV-H---      19 Dmelanogaster(msta-CG33548)/66-313                       14.8%     -------------------------------------------------DRVFM-DQLFRIVGVLNTNAFEAPCR-S---      20 Dmelanogaster(CG12119)/34-280                            15.8%     -------------------------------------------------KK-LI-EIMNRTVAVLRTNGFDKTTD-R---      21 Amellifera(XP_006565301.1)/26-284                        15.8%     ------------------------------------------EN-----DPSVS-EMIQQLCGILDVNSFELRSP-G---      22 Dmelanogaster(CG9642)/21-271                             13.6%     ------------------------------------------RG-----AA-DE-DLVQGLLGILDINAYEIRAP-E---      23 Dmelanogaster(CG9640)/17-268                             12.9%     ------------------------------------------PT-----QELTS-DALHAHCIRIDSNSFEVT-A-K---      24 Amellifera(NP_001229486.1-LOC724300)/57-301              12.7%     --------------------------------------------------SSTK-DIVSKICGLIDINALETVPP-E---      25 Dmelanogaster(CG14590-NP_610202.3)/55-322                14.9%     ------------------------------------------QTNPNLLTDCGP-EMLHRLCGIIETNFMVIELP-S---      26 Dmelanogaster(CG43129)/21-279                            11.6%     -------------------------------------------------NRFSE-ELIMQVVGVLEVNAFEARSP-K---      27 Dmelanogaster(G11160)/58-319                             13.9%     --------------------------------------------------DLEA-EQVHEVCGILDVNCFEIGQN-----      28 Amellifera(XP_624539.3-msta-like-Predicted)/54-297       12.8%     --------------------------------------------------DISE-EEIATIIGILQVNGHEVPLT-----      29 Dmelanogaster(CG8503-NP_610944.1)/52-301                 13.4%     --------------------------------------------------KFTE-EEIMKAVGALQINGHEVPTT-----      30 Agambiae(XP_309979.4-AGAP011530-PA)/50-300               14.8%     -------------------------------------------------NKWDE-DEILRVVGIIQVNGHEVPMT-----      31 Dpulex(DAPPUDRAFT_120473)/58-292                         14.4%     -------------------------------------------------DEFSE-EEIHASCGVIDVNAFEIRLA-----      32 Dpulex(DAPPUDRAFT_194440-Predicted)/53-302               11.7%     ---------------------------------------------------IPE-ELILQLCGILMVNSFEQPPM-K---      33 Dpulex(DAPPUDRAFT_2393)/50-297                           16.0%     --------------------------------------------------QYDS-ESIQRVCGILETNCFEIRIQ-----      34 Dmelanogaster(CG18136-NP_649084.1)/58-318                15.9%     --------------------------------------------------DWPE-MDILRIAAILDTNTFEVRQP-R---      35 Agambiae(XP_309220.5-AGAP001025-PA)/55-318               16.2%     --------------------------------------------------QYSE-QTVLKLSAILDTNCYEIRLP-E---      36 Dmelanogaster(CG1868-NP_724802.1)/226-549                13.1%     ------------------------------------------PASIEDWQLIIS-ALILRFAGQLLANGHVGDAL-LGVG      37 Agambiae(XP_319721.4-AGAP008973-PA)/165-486              13.3%     ------------------------------------------TMSPTELLVCCG-AFITRHIGQLVCNGHAISEL-RLAL      38 Bfloridae(XP_002593048.1-BRAFLDRAFT_74375)/6-196         11.1%     -------------------------------------------EDILPDRA-----QLESLYGKTTCNCFAI--------      39 Bfloridae(XP_002594298.1-BRAFLDRAFT_117670)/15-265       13.8%     -------------------------------------------KDALPDRA-----FMEEIYGKIASNSFAI--------      40 Cintestinalis(XP_002123001.1)/195-567                    11.0%     KSLSKMSNVMTKIE----------------------------PGQLTSMGEVIE-YLLHRHYLQVPINGQSISFV-TEEL      41 Drerio(Q08C84-Smyd4)/197-556                             11.1%     -----------------------EGV----------------KASWQPEMSMLG-ATALRHMMQLRCNAQAITAV-RVKE      42 Xtropicalis(NP_001072288.1-SMYD4)/212-545                12.2%     -----------------------SGT----------------IEDWSSVRQFLG-PTVLRHMLQLYCNAQAVTAL-QENE      43 Hsapiens(Q8IYR2-SMYD4)/244-602                           11.9%     -----------------------AAV----------------TPELCPDVTIWG-VAMLRHMLQLQCNAQAMTTI-QHTG      44 Ggallus(NP_001025886.1-SMYD4)/241-573                    11.1%     -----------------------GKT----------------SDELSPELMIMA-EAMLRHVLQLQCNAQAITVM-QELE      45 Hmagnipapillata(XP_002160254.2/232-532                   13.1%     ----------------------------------------------QQHHFGVG-SLLIRHIQQLICNAHAVTCL-SAEK      46 Dpulex(DAPPUDRAFT_312722-Pedicted)/241-525               14.2%     ---------------------------------------------SLESQYLVG-GLILVHVCQMVSNAHAITEL-CLID      47 Amellifera(XP_006565387.1-SMYD4-like-Predicted)/278-571  16.8%     ----------------------------------------------NDKQLYIS-SLLLRYILQLISNGHAITKS-NIFL      48 Bfloridae(XP_002589088.1-BRAFLDRAFT_75068)/251-714       10.4%     LGVDMCVEVVKKLG--LEGGNCTGATEGGGCGENKEGGDCAKSEEGVVCVEKMA-ALLCHHMQQLRCNAQAITTL-QEQD      49 Lgigantea(LOTGIDRAFT_169490)/248-638                     11.5%     ---SMESNIDGLLTNGKTEACQSSSNKTSFCGVL------------TNEMLDIG-GLLLRHIEQLVCNAHAITEV-QCTD      50 Skowalevskii(XP_002733823.1)/75-447                      10.9%     ---TVCSNDSNKFL---AA---------EQKLSL------------GDAEKAVA-SVLLHHLLQLRCNVHAVTEV-ATKT      51 Nvectensis(XP_001627273.1)/170-547                       12.5%     ---TICSRNTPYSR---QAYTSLGITTEEFCGKD------------GLSSDVVG-ALLVHHLQQMPCNVHAITAI-VSTS      52 Amellifera(XP_003250668.1-SMYD4-like-Predicted)/183-473  12.1%     ----------------------------------------------N-DILNLT-KLLCRLCYIYDIHAR--MDF-----      53 Amellifera(XP_001121272.2-SMYD4-like-Predicted)/230-549  13.9%     ---------------------------------------------LKESFISLA-VAILHHLQAINCNAYEIVEN-IYDK      54 Amellifera(XP_003249162.1-SMYD4-like-Predicted)/239-589  11.5%     ------------------------------------------EETPNDREIAVA-SLLLKHLQLLQFNAHEVFET-RLGM      55 Dmelanogaster(CG14122-NP_648574.1)/265-541               14.2%     ------------------------------------------GVNPTAVELQVA-TALLGLLQVLQYNAHQIYQT-QVTE      56 Agambiae(XP_311885.3-AGAP002999-PA)/268-544              13.9%     ------------------------------------------APEPTEQELEVG-AVLLSALQSLQFNAHEVYET-RITG      57 Amellifera(XP_392262.3-SMYD4-like-Predicted)/252-555     15.0%     ------------------------------------------EAKLSDGELYIG-GLILHNLMTIQFNAHEISEL-VIPK      58 Dmelanogaster(CG7759-NP_725048.1)/250-537                15.6%     ----------------------------------------------PDEVSIIC-SLVLRSLQFIQFNTHEVAEL-HKFS      59 Agambiae(XP_319583.4-AGAP008839-PA)/240-523              12.3%     ----------------------------------------------PQEQNFIG-GLLVHNLQLLQFNAHEVSEM-IRET      60 Dpulex(DAPPUDRAFT_68494-Predicted)/254-551               14.7%     ------------------------------------------DLKFSDQERWIG-SLLLRHLQLLQFNAHEVSEL-RMDR      61 Dpulex(DAPPUDRAFT_309882)/300-599                        12.5%     ------------------------------------------SLNITEDEIYFA-SLLLRHLQLLQFNAHEIHEF-VQLN      62 Dmelanogaster(CG8378-NP_610730.1)/196-491                13.8%     -------------------------------------------GGEE-GVNFFT-DLLFRHLQTSPSNMHGIDLVEQV--      63 Agambiae(XP_566179.1-AGAP000216-PA)/158-458              13.6%     -------------------------------------------SGSV-PYPWVG-EMCYRFLKVMQCNARPAQLT-----      64 Agambiae(XP_564258.1-AGAP011234-PA)/216-546              16.4%     -------------------------------------------SCPS-RRKLLF-ELLLRHTQTALTNKNDVYHMERVKL      65 Agambiae(XP_309407.4-AGAP011238-PA)/219-497              15.4%     -------------------------------------------SNPI-RSKLLF-DLLLRHVQTSLINKKRLNDYFDY--      66 Agambiae(XP_314169.4-AGAP005253-PB)/218-514              16.2%     -------------------------------------------SSPT-RSKLLF-DLLLRHWQTSLINKKQV--------      67 Agambiae(XP_309409.4-AGAP011237-PA)/206-481              15.2%     -------------------------------------------SNPA-RSKLLF-DLLLRHVQTSPINKKQFNNF-GY--      68 Agambiae(XP_307865.2-AGAP009448-PA)/166-466              15.6%     -------------------------------------------AKPS-IGKLLF-DLILRHVQVMRINRQFLSFY-EH--      69 Agambiae(XP_309762.4-AGAP010931-PA)/113-383              14.4%     -------------------------------------------ANPT-ASKLLL-DLILRYLQITECNYKLLTCI-KI--      70 Agambiae(XP_309378.2-AGAP011267-PA)/149-447              16.9%     -------------------------------------------ANPA-TNKFLL-DLILRYMQIVEFNRKLLSSN-AY--      71 Agambiae(XP_309383.4-AGAP011257-PA)/149-447              16.4%     -------------------------------------------ANPA-TNKFLL-DLILRYMQIVNCNRKLLSFN-AY--      72 Agambiae(XP_307655.3-AGAP012638-PA)/149-447              16.4%     -------------------------------------------ANPA-TNKILL-DLILRYEQIVECNSKLLSFN-AY--      73 Agambiae(XP_320681.4-AGAP011835-PA)/183-484              14.6%     -------------------------------------------MNDN-VRELLN-ELILRHLQTGPVNMHSLHYM-EY--      74 Agambiae(XP_309411.4-AGAP011232-PA)/162-434              14.4%     -------------------------------------------ADPI-LRKTLL-NLLLHHLQSTIVNHQFLHYM-DY--      75 Amellifera(XP_001120776.2-SMYD4-like-Predicted)/251-554  13.4%     ------------------------------------------DINNY-SDIIFCSSIMFRACVIMSSN------------      76 Dpulex(DAPPUDRAFT_305694-Predicted)/258-553              16.3%     ------------------------------------------GKEDD-PDEILA-TAVLQLIQSYPCNAHEISHL-AFPL      77 Dpulex(EFX87901.1)/258-554                               14.2%     -------------------------------------------GKDR-PDEVLA-TAVLRLLHSYPCNAHEISHM-AIPV      78 Amellifera(XP_001122116.2-SMYD4-like-Predicted)/234-534  13.5%     ------------------------------------------LIKND-NVIFVG-GLILRHQQLIPSNIHSFSEE-C---      79 Hmagnipapillata(XP_002159692.1)/239-485                  13.7%     -------------------------------------------------LKIVC-AHLLKHIQMLPCNAHEVSEL-QLKA      80 Nvectensis(XP_001623892.1)/215-512                       13.9%     ------------------------------------------LPSRE-DQAFIG-GLLLRHLQSLPCNAHEISEL-QLSL      81 Lgigantea(LOTGIDRAFT_143433)/100-395                     15.6%     ------------------------------------------KAHLP-EKCVIG-GHILRHIQMLPCNAHEVSEF-AYRE      82 Skowalevskii(XP_002740933.1)/253-549                     16.3%     ------------------------------------------DNEED-VAIFIG-GHMLTHLQTIPCNAHEISEY-ELWR      83 Dpulex(EFX73755.1)/49-306                                15.8%     --------------------------------------------------ILPNSVELLGIYGRMCINSFNILNG-----      84 Amellifera(XP_625013.1-SMYD3-Predicted)/1-253            15.0%     --------------------------------------------------SIPNSAELMGIYGRIYINSFNISDL-----      85 Dmelanogaster(Buzidau-CG13761)/26-282                    14.4%     -------------------------------------------------STVPNKTELMSIYGRLITNGFNILDA-----      86 Agambiae(XP_319707.4-AGAP008954-PA)/1-254                16.7%     --------------------------------------------------SRPTKAELLRIYGKMCINTFNILDA-----      87 cintestinalis(NP_001071820.1)/15-282                     18.6%     ------------------------------------------------PD---SDEEMAHLFAIIDCNAIGLNDH-----      88 Drerio(Q6P0R5-Smyd1a)/18-279                             15.6%     ------------------------------------------------AV---GEDYVSHLFGVISCNGFTLSDQ-----      89 Derio(Q2MJQ9-Smyd1b)/13-274                              15.9%     ------------------------------------------------PH---TVDSVSHILGVINCNGFMVSDQ-----      90 Xtropicalis(NP_001120357.1-SMYD1)/13-261                 16.3%     ------------------------------------------------QF---GMQYISHIFSVISCNGFTLSDQ-----      91 Hsapiens(Q8NB12-SMYD1)/18-279                            16.1%     ------------------------------------------------QF---SMQYISHIFGVINCNGFTLSDQ-----      92 Ggallus(NP_989486.1-SMYD1)/13-274                        16.4%     ------------------------------------------------QF---GMQYISHIFGVINCNAFTLSDQ-----      93 Drerio(Q5RGL7-Smyd2b)/19-268                             15.0%     --------------------------------------------------DFPDHQALLTLFSQVHCNGFTVEDE-----      94 Drerio(Q5BJI7-Smyd2a)/18-267                             15.2%     --------------------------------------------------DFPDNAALTELIAQVNCNGFTIEDE-----      95 Xtropicalis(XP_002934751.2-SMYD2-like-Predicted)/16-265  15.2%     --------------------------------------------------HYSDNAAQVFLFAQVNCNGFTIEDE-----      96 Hsapiens(Q9NRG4-SMYD2)/18-267                            16.1%     --------------------------------------------------GFPDNDSLVVLFAQVNCNGFTIEDE-----      97 Ggallus(XP_419420.1-SMYD2-Predicted)/21-270              14.7%     --------------------------------------------------EYPDNAALVVLFAQVNCNGFTIEDE-----      98 cintestinalis(XP_002128556.1)/14-266                     18.0%     --------------------------------------------KPL-PPQVTDNKMLLELCARIKNNSFAICNE-----      99 Lgigantea(LOTGIDRAFT_177746)/1-216                       15.4%     ------------------------------------------DHMTL-PPA----HTLFSFFGMMVINTFSICND-----     100 Drerio(E7EZZ6-SMYD3)/16-267                              18.8%     ------------------------------------------DLSRL-PSG----LDPVSLLARVTCNCFSISDG-----     101 Xtropicalis(XP_004914684.1|-SMYD3-Predicted)/15-264      18.0%     ------------------------------------------EISQL-PPG----FQVLEYFGKVTCNSFTISDG-----     102 Hsapiens(Q9H7B4-SMYD3)/15-266                            17.4%     ------------------------------------------DASQL-PPA----FDLFEAFAKVICNSFTICNA-----     103 Ggallus(XP_419536.1-SMYD3-Predicted)/15-266              18.0%     ------------------------------------------DASHL-PPA----IDFFQIFTKVTCNCFTISNG-----     104 Tadhaerens(XP_002109888.1)/20-262                        19.2%     ------------------------------------------------AI---NENEIIEIISRVTCNTFTICNS-----     105 Hmagnipapillata(XP_002163555.2)/16-259                   17.2%     ----------------------------------------------I-NI---NDIDIYGLMCKASCNSFAITNA-----     106 Nvectensis(XP_001627600.1)/17-253                        18.2%     ----------------------------------------------S-PP------EGLELFSKISCNSFAICDG-----     107 Bfloridae(XP_002594889.1-BRAFLDRAFT_124463)/14-258       18.1%     ------------------------------------------------MD---DAREIFELFGRMTCNTFSICDP-----     108 Skowalevskii(XP_006817727.1)/14-260                      15.8%     ----------------------------------------------L-PN---DVSDLLCIFGRMTSNSFSVCDS-----         consensus/100%                                                     ...................................................................p............         consensus/90%                                                                                                ...............h.thh..h.hNt..h... ....         consensus/80%                                                                                                .............t.hhphht.h.hNs.tlt.. ....         consensus/70%                                                                                                ..... ...h.s.thlhphhthltsNuhtlsp. ....                                                                         561          .         .         .         6         .         .         .         . 640   1 Tadhaerens(XP_002114620.1)/25-373                       100.0%     YARNVDAAK-FEKQEEDYINSFLD-QLY------ADMNEESGDFLDCEGSGLYLLQSCCNHDCSPNVEINFLDN------       2 Bfloridae(XP_002609030.1-BRAFLDRAFT_84846)/1-276         31.0%     YVHNCDALE-LPSQDREKLDAFID-QLY------VDMEH-------------------GNHSCEPTAEPSFDES------       3 cintestinalis(XP_002127168.1)/13-358                     39.9%     WVHNCDELD-LNPQDKEELDNLID-GLY------EELENVAGSFLNCEGAGLYRIQSKCNHSCEPNAEVCFPNN------       4 Dpulex(EFX89935.1)/23-367                                41.0%     WVRKVSEND-----IDPTTDALID-TIY------QEMENESGDFLNNEGSALFAIQSACNHSCEPNCISTFPFS------       5 Dmelanogaster(CG3353-NP_650955.1)/13-363                 39.1%     WVAKVSDLP-LTDSEKEQLDTVID-GLY------AKVGEFAGEFLNNEGSGLYLLQSKINHSCVPNACSTFPYS------       6 Hmagnipapillata(XP_002163562.2)/21-371                   44.2%     YVHNIDAKV-MSDNEREQIDAFID-QLY------LHMEKESGSFLNCEGSGLFKMQSRCNHSCYPNAEATFPYN------       7 Agambiae(XP_313299.1-AGAP003552-PA)/13-365               41.4%     WVKNATACE-MSDQERQAVDQLID-DLY------AKMDDVVGSFLNNEGSALYARQSKINHSCAPNAETVFPKS------       8 Amellifera(XP_394075.2-SMYD5-like-Prediction)/16-364     45.0%     WVKNVSALE-LPREERIQVDKLID-RIY------DDMEEAVASFLNNEGSGLYILQSSVNHSCVPNAIVEFPYS------       9 Nvectensis(XP_001627062.1)/18-370                        48.0%     YVHNIDSYPALSDDERQAIDIFLN-QLY------EEMERVSGQFLNCEGAGLYALQSSCNHSCAPNAEVTFPKN------      10 Skowalevskii(XP_002735533.1)/24-372                      50.1%     YVHNCDALD-LNTDDRQRLDLFID-QLY------VDIEKESGSFLNCEGSALYSLQSCCNHSCVPNAEVTFPDN------      11 Lgigantea(LOTGIDRAFT_231752)/19-367                      51.0%     WVKNCEDLE-LPEDKKTELDDFID-QMY------EELEKESGSFLNCEGSGLYELTSSCNHSCDPNAGITFPHN------      12 Drerio(F1RET2-Smyd5)/32-380                              49.9%     WVHACDALE-LPRQQREQLDAFID-QLY------KDIDKETGDFLNCEGSGLFLLQSSCNHSCVPNAEASFPEN------      13 Ggallus(NP_001012912.1-SMYD5)/39-387                     50.1%     WVHACDALD-LPMLQREELDAFID-QLY------KDIEKESGEFLNCEGSGLYMLQSCCNHSCIPNAETSFPDN------      14 Hsapiens(Q6GMV2-SMYD5)/33-381                            49.9%     WVHACDTLE-LKPQDREQLDAFID-QLY------KDIEAATGEFLNCEGSGLFVLQSCCNHSCVPNAETSFPEN------      15 Xtropicalis(A9ULL8-SMyd5)/32-382                         51.0%     WVHACDALE-LPPREREQLDSLID-QLY------KDIEKVTGEFLNCEGSGLYLLQSCCNHSCVPNAEASFPDN------      16 Lgigantea(LOTGIDRAFT_232186)/323-670                     20.5%     YHAFMKKL---STDLRGF-------QMI------KYLEK---SPPYAGFCGMFPLHACLNHSCCNNVEIRDGDC------      17 Bfloridae(XP_002589246.1-BRAFLDRAFT_74594)/380-720       22.0%     WHEFVAEF---HRTARPGE--NHR-RVA------QEMRG---EPKDVTFGGLYALQSSLNHSCDKNVDVMDAVV------      18 Amellifera(XP_006565332.1)/43-285                        14.2%     ----------------------------------------D-KDHFTNLRGLYPLGSLQNHCCIPNTRHYFDEK------      19 Dmelanogaster(msta-CG33548)/66-313                       14.8%     ------------------------------------------GGHETLLRGLFPLTAIMNHECTPNASHYFENG------      20 Dmelanogaster(CG12119)/34-280                            15.8%     ----------------------------------------TNDNQEFNYRALYPLFGVVNHDCIPNAYYTFEEK------      21 Amellifera(XP_006565301.1)/26-284                        15.8%     ------------------------------------------GMDGLLLRGLYLEASMMAHDCRGNVHVTADD-------      22 Dmelanogaster(CG9642)/21-271                             13.6%     --------------------------------------------VGGAMRGLYRRAGLFAHSCTPNLVISIDD-------      23 Dmelanogaster(CG9640)/17-268                             12.9%     --------------------------------------------DGDTLKGIFVWGATLPHHCVPNTVVALDE-------      24 Amellifera(NP_001229486.1-LOC724300)/57-301              12.7%     -----------------------------------------------GCVAIYETACLLEHSCLANTRHSFTID------      25 Dmelanogaster(CG14590-NP_610202.3)/55-322                14.9%     ------------------------------------------G---VELSGLFRQACMMEHACQPNCDFQFDNK------      26 Dmelanogaster(CG43129)/21-279                            11.6%     ------------------------------------------G---YPLRCLFPYTGILAHNCVPNTSRSIYPS------      27 Dmelanogaster(G11160)/58-319                             13.9%     ---------------------------------------------GAKARTLYPSAFLLAHDCTPNTAHTDDPS------      28 Amellifera(XP_624539.3-msta-like-Predicted)/54-297       12.8%     ---------------------------------------------DSPYVAVYEMASLIEHNCRANCSKSFTD-------      29 Dmelanogaster(CG8503-NP_610944.1)/52-301                 13.4%     ---------------------------------------------DPSHVAVFYTASFTENSCLPNLAKSFNK-------      30 Agambiae(XP_309979.4-AGAP011530-PA)/50-300               14.8%     ---------------------------------------------EPSSVAIYNMASMLEHSCRPNLAKSFTN-------      31 Dpulex(DAPPUDRAFT_120473)/58-292                         14.4%     ------------------------------------------GNQYQQVLGVFPLASMMSHNCVANTQHVIDA-------      32 Dpulex(DAPPUDRAFT_194440-Predicted)/53-302               11.7%     ------------------------------------------GNSQHGLVAVYSTASLLEHDCVANAIKTFTN-------      33 Dpulex(DAPPUDRAFT_2393)/50-297                           16.0%     ------------------------------------------G--RVSVRGLYPTASLMNHDCVANTRHVFDPA------      34 Dmelanogaster(CG18136-NP_649084.1)/58-318                15.9%     ------------------------------------------E--RRKIRALYPGAAMISHDCVPNMRHRFDD-------      35 Agambiae(XP_309220.5-AGAP001025-PA)/55-318               16.2%     ------------------------------------------Q--HVKVRGLYPLGAMLSHDCRPNTKHYFDD-------      36 Dmelanogaster(CG1868-NP_724802.1)/226-549                13.1%     MEP--------------KEFVMLQPELWQKPRHLKRGQLHNLSHSDPITAINLPYLSLCNHACEPSIRTKFD-G------      37 Agambiae(XP_319721.4-AGAP008973-PA)/165-486              13.3%     PSK--------------GQFYNLN------DSLLLAGTLHLCLKSSRVFTAIFPRISMFNHSCDPNIRNHFE-R------      38 Bfloridae(XP_002593048.1-BRAFLDRAFT_74375)/6-196         11.1%     ----------------------------------------HNLDLREIGVGLYPQAAMINHSCKSNCVSTFR-G------      39 Bfloridae(XP_002594298.1-BRAFLDRAFT_117670)/15-265       13.8%     ----------------------------------------LDENMCSIGIGVYPQASMINHSCKSNCIGMFY-G------      40 Cintestinalis(XP_002123001.1)/195-567                    11.0%     C------------------------------------DNVTVTRRDIVASAFFPTMSMMNHSCDCNTDALFN-G------      41 Drerio(Q08C84-Smyd4)/197-556                             11.1%     E----------------------S----------G--MAVQSSSEIRIATAIFPVLSLLNHSCSPNTSISFT-TGFQPDP      42 Xtropicalis(NP_001072288.1-SMYD4)/212-545                12.2%     D----------------------E----------SSLSLVKSNKSIRLATAVFPVLSLLNHSCDPNTTVSFT-G------      43 Hsapiens(Q8IYR2-SMYD4)/244-602                           11.9%     P----------------------K----------G--SIVTDSRQVRLATGIFPVISLLNHSCSPNTSVSFI-S------      44 Ggallus(NP_001025886.1-SMYD4)/241-573                    11.1%     S----------------------G----------D--GAVVNKKPVRLATAFFPVLSLLNHSCSPNISVSFS-G------      45 Hmagnipapillata(XP_002160254.2/232-532                   13.1%     L----------------------------------DTTSVIDQEQVRIATAIYPTTSLLNHSCEPTILNCFH-K------      46 Dpulex(DAPPUDRAFT_312722-Pedicted)/241-525               14.2%     E---------------------------------------NNERQERIATAIYPSASLMNHNCDPTVINSFQ-G------      47 Amellifera(XP_006565387.1-SMYD4-like-Predicted)/278-571  16.8%     S---------------------------------ENDSS--MIQQDIVATGIYPSASIMNHSCDPNIINIFV-N------      48 Bfloridae(XP_002589088.1-BRAFLDRAFT_75068)/251-714       10.4%     S------------------------------------VSLLEDKQVRLATAVFPTEALLNHSCRPNVFVSFQ-G------      49 Lgigantea(LOTGIDRAFT_169490)/248-638                     11.5%     T---------------------------------INDSMILDTSQVRIATAIYPTASLMNHSCDPTIISSFH-G------      50 Skowalevskii(XP_002733823.1)/75-447                      10.9%     D---------------------------------SSTSFVATTQQIRIAVAVYGTASMLNHSCTPNVIAGYD-G------      51 Nvectensis(XP_001627273.1)/170-547                       12.5%     SSD--------------EEDEEMG----------SSHDQVVAREQRRIASAIYPTASLLNHACDPDVLVSFV-D------      52 Amellifera(XP_003250668.1-SMYD4-like-Predicted)/183-473  12.1%     --------------------------------------VPIFERYIALLQNLYFLLNLVRHSCSGNTIYTVHKN------      53 Amellifera(XP_001121272.2-SMYD4-like-Predicted)/230-549  13.9%     --------------------------------------KTHIWEPRQIGGAIYPSVSLINHSCYPNVVRHTYPS------      54 Amellifera(XP_003249162.1-SMYD4-like-Predicted)/239-589  11.5%     E------------------------------------HRFRGSKPIYIGVAIYPTVARFNHDCYPAVTRYFL-G------      55 Dmelanogaster(CG14122-NP_648574.1)/265-541               14.2%     E------------------------------------HRFDGSKTVYLAAGLYGTGSYFNHECWPSTACHFV-G------      56 Agambiae(XP_311885.3-AGAP002999-PA)/268-544              13.9%     E------------------------------------HRFDTAKVQYIGVGIYRGASMFNHECYPGVTRTFL-G------      57 Amellifera(XP_392262.3-SMYD4-like-Predicted)/252-555     15.0%     A-----------------------------------DNNLANAKSKFIGGGLYPTISLFNHSCNPGIIRYFI-G------      58 Dmelanogaster(CG7759-NP_725048.1)/250-537                15.6%     S-----------------------------------S---GREKSIFIGGAIYPTLALFNHSCDPGVVRYFR-G------      59 Agambiae(XP_319583.4-AGAP008839-PA)/240-523              12.3%     A-----------------------------------E---DIGKSTFIGGGLYPTLALFNHSCDPGVTRYYR-G------      60 Dpulex(DAPPUDRAFT_68494-Predicted)/254-551               14.7%     P------------------------------------GCMEGAKTFFLGAGVYSTVALLNHSCEPGVIRHFI-G------      61 Dpulex(DAPPUDRAFT_309882)/300-599                        12.5%     E------------------------------------KNMRSTKTVYIGVGIYPTVAFFNHSCRPDVARYFL-G------      62 Dmelanogaster(CG8378-NP_610730.1)/196-491                13.8%     ---------------------------------------NETKDDQTHSSGAYAFLSLINHSCAPNTVRIYE-G------      63 Agambiae(XP_566179.1-AGAP000216-PA)/158-458              13.6%     ---------------------------------RR-DEPEGQYRAVPFALRCHPLISLLNHSCAPNVKCFDLRD------      64 Agambiae(XP_564258.1-AGAP011234-PA)/216-546              16.4%     EDDHVEDD------E-SEEDSSVD----NNNSDDRKSPRNVHHEERTHAIAIYPLFSMVNHSCIPNVAPIHLLD------      65 Agambiae(XP_309407.4-AGAP011238-PA)/219-497              15.4%     ---------------------------------DSDEGKPTHFEERPHAMAVYPLSSMLNHSCVPNVAPINLLD------      66 Agambiae(XP_314169.4-AGAP005253-PB)/218-514              16.2%     -----------------------------------D--DEEEYSDEMHAIAVYPLFSMVNHSCIPNVAPIHLLD------      67 Agambiae(XP_309409.4-AGAP011237-PA)/206-481              15.2%     ---------------------------------DSD--DEDIFEERTHAIAVYPLFSMANHSCIPNVAPIHLLD------      68 Agambiae(XP_307865.2-AGAP009448-PA)/166-466              15.6%     ------------------------------------RPDRQRFQAKEYGTACYPLVSMFNHSCASNVRRLILRD------      69 Agambiae(XP_309762.4-AGAP010931-PA)/113-383              14.4%     ------------------------------------T--NRNPEDETFTTSCYPLISMLNHSCAPNVRRLILPD------      70 Agambiae(XP_309378.2-AGAP011267-PA)/149-447              16.9%     ------------------------------------K--VKKYVAESLATSCYPLISMLNHSCAPNVQRITLRD------      71 Agambiae(XP_309383.4-AGAP011257-PA)/149-447              16.4%     ------------------------------------K--VNEYVAESFAVGCYPLISMLNHSCAPNVKRITLPD------      72 Agambiae(XP_307655.3-AGAP012638-PA)/149-447              16.4%     ------------------------------------K--VKEYVAESFAVGCYPLISMLNHSCAPNVQRITLPD------      73 Agambiae(XP_320681.4-AGAP011835-PA)/183-484              14.6%     ------------------------------------QPEQRVYEMENHVSACFPILSMLNHSCAPNVTRITLRD------      74 Agambiae(XP_309411.4-AGAP011232-PA)/162-434              14.4%     ------------------------------------LAEQDVYEPDEYAIACFPLLSMLNHSCAPNVKRITMRD------      75 Amellifera(XP_001120776.2-SMYD4-like-Predicted)/251-554  13.4%     ------------------------------------CFSVQQEPGIKIGSGLYVTNSLYNHSCAPNTFRHFE-G------      76 Dpulex(DAPPUDRAFT_305694-Predicted)/258-553              16.3%     PGTPSG---------------------------PDLPSTLQQIRLCEIGAAAMPVLSLINHSCDPNVVRDCY-G------      77 Dpulex(EFX87901.1)/258-554                               14.2%     PSGFCA---------------------------QSKSLQLQQIQSCEIGSAAFPVVSLMNHSCNPNVVHLCY-G------      78 Amellifera(XP_001122116.2-SMYD4-like-Predicted)/234-534  13.5%     -----------------------------------------GLDAVERGIAAMPFFSLINHSCNPNILRHSR-S------      79 Hmagnipapillata(XP_002159692.1)/239-485                  13.7%     S-------------------------------------NYKDSELKEIGSAVYATLSLLNHSCDPSVVRHCY-G------      80 Nvectensis(XP_001623892.1)/215-512                       13.9%     K-------------------------------------SVATSEAAEIGAGIYGTLSLFNHSCEPNVTRFFY-G------      81 Lgigantea(LOTGIDRAFT_143433)/100-395                     15.6%     Y-------------------------------------DLPNSQTMEIGSGIYATLSLINHSCDPNVVRHSY-G------      82 Skowalevskii(XP_002740933.1)/253-549                     16.3%     S-------------------------------------DITKCHFVEVGSGLYPTMSLVNHSCDPVVTRNCY-G------      83 Dpulex(EFX73755.1)/49-306                                15.8%     ------------------------------------------EMQ-AIGTGIYLAPSILDHSCSPNAVATFD-G------      84 Amellifera(XP_625013.1-SMYD3-Predicted)/1-253            15.0%     ------------------------------------------DMN-NIGAGIYLGPSILDHSCKPNAVATFE-G------      85 Dmelanogaster(Buzidau-CG13761)/26-282                    14.4%     ------------------------------------------EMN-SIATAIYLGVSITDHSCQPNAVATFE-G------      86 Agambiae(XP_319707.4-AGAP008954-PA)/1-254                16.7%     ------------------------------------------EMS-TIGTGMYIGASIIDHSCRPNVVVSFD-G------      87 cintestinalis(NP_001071820.1)/15-282                     18.6%     ------------------------------------------RGVQTIGVGIYPGISMLNHDCSPNCVAMNN-G------      88 Drerio(Q6P0R5-Smyd1a)/18-279                             15.6%     ------------------------------------------RGLQSVGIGLFPNLCLVNHDCWPNCTVILN-HGDQSAL      89 Derio(Q2MJQ9-Smyd1b)/13-274                              15.9%     ------------------------------------------RGLQAVGVGLFPNLCLVNHDCWPNCTVILN-NGNQSAI      90 Xtropicalis(NP_001120357.1-SMYD1)/13-261                 16.3%     ------------------------------------------RGLQAVGVGIFPNLCLANHDCWPNCTVIFN-NGK----      91 Hsapiens(Q8NB12-SMYD1)/18-279                            16.1%     ------------------------------------------RGLQAVGVGIFPNLGLVNHDCWPNCTVIFN-NGNHEAV      92 Ggallus(NP_989486.1-SMYD1)/13-274                        16.4%     ------------------------------------------RGLQAVGVGIFPNLCQANHDCWPNCTVIFN-NGNHEAV      93 Drerio(Q5RGL7-Smyd2b)/19-268                             15.0%     ------------------------------------------ELS-NLGLAIFPDIALLNHSCSPNVIVTYR-G------      94 Drerio(Q5BJI7-Smyd2a)/18-267                             15.2%     ------------------------------------------ELS-HLGSALFPDVALMNHSCSPNVIVTYK-G------      95 Xtropicalis(XP_002934751.2-SMYD2-like-Predicted)/16-265  15.2%     ------------------------------------------ELS-HLGSAIFPDVALMNHSCCPNVIVTYK-G------      96 Hsapiens(Q9NRG4-SMYD2)/18-267                            16.1%     ------------------------------------------ELS-HLGSAIFPDVALMNHSCCPNVIVTYK-G------      97 Ggallus(XP_419420.1-SMYD2-Predicted)/21-270              14.7%     ------------------------------------------ELS-HLGSAIFPDVALMNHSCCPNVIVTYK-G------      98 cintestinalis(XP_002128556.1)/14-266                     18.0%     ------------------------------------------ELQSDVGTGVYLNCSFINHSCEPNCVAEFN-M------      99 Lgigantea(LOTGIDRAFT_177746)/1-216                       15.4%     ------------------------------------------DLQ-PIGSGIYTSPSMLDHSCDPNAVAIFS-G------     100 Drerio(E7EZZ6-SMYD3)/16-267                              18.8%     ------------------------------------------ELQ-DVGVGLYPSMSLLNHDCQPNCIMMFE-G------     101 Xtropicalis(XP_004914684.1|-SMYD3-Predicted)/15-264      18.0%     ------------------------------------------EMQ-DVGVGLYPSMSLLNHSCDPNCVIVFE-G------     102 Hsapiens(Q9H7B4-SMYD3)/15-266                            17.4%     ------------------------------------------EMQ-EVGVGLYPSISLLNHSCDPNCSIVFN-G------     103 Ggallus(XP_419536.1-SMYD3-Predicted)/15-266              18.0%     ------------------------------------------EMQ-DVGVGLYPSMSLLNHSCDPNCVIIFE-G------     104 Tadhaerens(XP_002109888.1)/20-262                        19.2%     ------------------------------------------EMQ-TVGIGVYPGLSLVNHSCSPNCSATFR-G------     105 Hmagnipapillata(XP_002163555.2)/16-259                   17.2%     ------------------------------------------ELN-SLGTGIFSSASLFNHSCDPNCVATFN-G------     106 Nvectensis(XP_001627600.1)/17-253                        18.2%     ------------------------------------------EMQ-AIGTGIFPNAVCLNHSCAPNSVAVFN-G------     107 Bfloridae(XP_002594889.1-BRAFLDRAFT_124463)/14-258       18.1%     ------------------------------------------EMQ-YIGIGIYPKMSLFNHSCEPNCVAVFN-G------     108 Skowalevskii(XP_006817727.1)/14-260                      15.8%     ------------------------------------------EMK-PIGVGIYPSASLLNHSCDPNCVAVFN-G------         consensus/100%                                                     ............................................................ptC..s..............         consensus/90%                                                      ......... .............. ...      ..............h.uha...uhhsHsC.ssh...h...               consensus/80%                                                      ...                    .          ..............s.ula..huhhNHsC.PNs.h.h..s               consensus/70%                                                      .                                 . ......t...thusulashhShhNHSCtPNshhha..s                                                                               641          :         .         .         .         .         7         .      ] 717   1 Tadhaerens(XP_002114620.1)/25-373                       100.0%     -----------------N--ATLTVKAIRNI--SEG--QELCISYIDSDI--KNWKK--RQAILMENYLFECTCNRC       2 Bfloridae(XP_002609030.1-BRAFLDRAFT_84846)/1-276         31.0%     -----------------N--YVLSMRALRDI--TEG--EELFICYLDECERTRSRHS--RQKLLRENYLFSCTCEKC       3 cintestinalis(XP_002127168.1)/13-358                     39.9%     -----------------N--HRLAVKACRDI--AAG--EEITISYLSQCQIARGCRS--RQQYLKENYLFHCCCSKC       4 Dpulex(EFX89935.1)/23-367                                41.0%     -----------------N--HTVALVASKDL--EEG--EEIFISYLDECAQSRSRHS--RRKILKENYLFHCNCSRC       5 Dmelanogaster(CG3353-NP_650955.1)/13-363                 39.1%     -----------------N--DIVVLKALAPI--QQG--EEICISYLDECMLERSRHS--RHKVLRENYVFICQCPKC       6 Hmagnipapillata(XP_002163562.2)/21-371                   44.2%     -----------------N--STLVLVATEDI--TKD--EEICVCYLDECQRSRSRHS--RRKLLRENYLFECTCSLC       7 Agambiae(XP_313299.1-AGAP003552-PA)/13-365               41.4%     -----------------N--HMLALRATRDI--QPG--EEICISYLDECNLQRSRHS--RQKTLKDYYLFICQCEKC       8 Amellifera(XP_394075.2-SMYD5-like-Prediction)/16-364     45.0%     -----------------N--NVLVLKAIRDI--HPE--EEICISYLDECCLERSRHS--RQKALNSLYLFQCYCNKC       9 Nvectensis(XP_001627062.1)/18-370                        48.0%     -----------------N--STLVLKALHPI--KNG--EEICISYLEECQRERSRHS--RLKYLRENYIFDCTCTKC      10 Skowalevskii(XP_002735533.1)/24-372                      50.1%     -----------------D--AAVSVMALQDI--QEN--EEICISYLGECDIGRSRHS--RQKILRENYLFNCNCMKC      11 Lgigantea(LOTGIDRAFT_231752)/19-367                      51.0%     -----------------N--HVLTLVALKPI--QPE--EEIYISYISECEMSRSRHS--RQKILRENYLFTCRCRKC      12 Drerio(F1RET2-Smyd5)/32-380                              49.9%     -----------------N--FLLHLTALGDI--GPG--EEICISYLDCCQRDRSRHS--RHKILRENYLFICSCQKC      13 Ggallus(NP_001012912.1-SMYD5)/39-387                     50.1%     -----------------N--FLLYLTALEDI--EAG--EEICISYLDCCQRERSRHS--RNKILRENYLFTCSCPKC      14 Hsapiens(Q6GMV2-SMYD5)/33-381                            49.9%     -----------------N--FLLHVTALEDI--KPG--EEICISYLDCCQRERSRHS--RHKILRENYLFVCSCPKC      15 Xtropicalis(A9ULL8-SMyd5)/32-382                         51.0%     -----------------N--FILHLTALEDI--QPG--EEICISYLDCCQRDRSRHS--RQKILRENYLFVCSCPKC      16 Lgigantea(LOTGIDRAFT_232186)/323-670                     20.5%     -----------------NGTPGVNVVAKRFI--KTG--EELFTSYIDNK---LSRNI--RRAWLYKSFNFWCQCPQC      17 Bfloridae(XP_002589246.1-BRAFLDRAFT_74594)/380-720       22.0%     -----------------DGKPGVVIRAKQPI--KKG--GELYTTYIDTS---MQRPQ--RRAWLYRAYHFWCECQRC      18 Amellifera(XP_006565332.1)/43-285                        14.2%     -------------------FR-LYVRAALPI--SAG--EEITMSYTSLF---WDTTL--RRQFLNVTKNFSCMCKRC      19 Dmelanogaster(msta-CG33548)/66-313                       14.8%     --------------------RLAVVRAARDI--PKG--GEITTTYTKIL---WGNLT--RNIFLKMTKHFACDCVRC      20 Dmelanogaster(CG12119)/34-280                            15.8%     -------------------TNNMIVRAAVDI--PEG--FEVTTTYTKLF---TGNIA--RHLFLKMKKSFTCKCSRC      21 Amellifera(XP_006565301.1)/26-284                        15.8%     -------------------NFHLTVYASIPI--KEG--DTIFFNYTSSL---LGTTG--RREYLRTGKYFECECDLC      22 Dmelanogaster(CG9642)/21-271                             13.6%     -------------------EQRIKVYANRFI--AAG--EILYNCYTNVL---LGTEE--RRKILKVGKCFDCSCPRC      23 Dmelanogaster(CG9640)/17-268                             12.9%     -------------------QFNMKLYAAVPL--QPG--DIIYNSYTNPL---MGTSQ--RQHQLRLSRRLECICSRC      24 Amellifera(NP_001229486.1-LOC724300)/57-301              12.7%     -----------------KGRPRITVKALCSI--QKG--DHLSTMYTHAL---WATRV--RRSHLLETKYFSCHCKRC      25 Dmelanogaster(CG14590-NP_610202.3)/55-322                14.9%     -------------------TQQVAVRAGCDL--RKG--DHLRITYTNIL---WGTQL--RQHHLRLTKHFSCRCSRC      26 Dmelanogaster(CG43129)/21-279                            11.6%     ------------------EGYKIRLRAMVDL--EEG--QPLHHSYTYTL---DGTAQ--RQKHLKQGKFFTCQCERC      27 Dmelanogaster(G11160)/58-319                             13.9%     -------------------SFEILLRTSRRV--RER--EALTLSYAYTL---QGTLK--RRAFMHEGKLFWCCCRRC      28 Amellifera(XP_624539.3-msta-like-Predicted)/54-297       12.8%     -------------------MGGLIIRAALPI--TKG--DHISICYTDPL---WGTAN--RRHHLFKTKFFECICNRC      29 Dmelanogaster(CG8503-NP_610944.1)/52-301                 13.4%     -------------------NGHCILWAPREI--KKN--AHLSICYSDAM---WGTAD--RQRHLMQTKLFKCACERC      30 Agambiae(XP_309979.4-AGAP011530-PA)/50-300               14.8%     -------------------RGEVVMWAPNPI--RRG--DRLSICYTDVL---WTTGN--RLEHLQQTKMFRCECERC      31 Dpulex(DAPPUDRAFT_120473)/58-292                         14.4%     -------------------NYTMTVRASVPI--MKG--EQIFTSYTLPL---EGTKE--RRDVLRHSKLFECDCSRC      32 Dpulex(DAPPUDRAFT_194440-Predicted)/53-302               11.7%     -------------------KGDIVIRAAVPI--PKG--EKIALCYTEPL---WGTMN--RQRHLSQTKFFQCVCERC      33 Dpulex(DAPPUDRAFT_2393)/50-297                           16.0%     -------------------DFRIRILATKDI--PAG--DKISATYTRSL---WNTLD--RRLHLKSTKHFWCQCSRC      34 Dmelanogaster(CG18136-NP_649084.1)/58-318                15.9%     -------------------DMNIVFLAKRKI--AKG--EILSISYTQPL---RSTIQ--RRVHLRQAKCFDCSCARC      35 Agambiae(XP_309220.5-AGAP001025-PA)/55-318               16.2%     -------------------RLHMVLVATVDI--PAG--GVIHASYTQPL---LGTVQ--RRLALRQAKCFDCCCERC      36 Dmelanogaster(CG1868-NP_724802.1)/226-549                13.1%     --------------------CSVVNYAAKDI--LEG--EEIFNCYTMDYR-NSLKLQ--RSHPLKAIYKFECTCAKC      37 Agambiae(XP_319721.4-AGAP008973-PA)/165-486              13.3%     --------------------ATLTVHATRPI--GAG--GEVFNCYGPHYR-LMAAAE--RKMLLRAQYCFECGCERC      38 Bfloridae(XP_002593048.1-BRAFLDRAFT_74375)/6-196         11.1%     --------------------PTLQIRALVDI--QPG--EEVCYSYTEKG---NVTHE--RR-DELRKYFFECQCPHC      39 Bfloridae(XP_002594298.1-BRAFLDRAFT_117670)/15-265       13.8%     --------------------PQIQIRANEFI--RPG--EQIFHGYIPPL---LPTAK--RQEKLLKTYHFLCQCADC      40 Cintestinalis(XP_002123001.1)/195-567                    11.0%     --------------------STVTFRSNQFI--PVG--AEITHCYGPSVF-HASFEE--RQKTLKENYSFDCDCTPC      41 Drerio(Q08C84-Smyd4)/197-556                             11.1%     HNQLGCSEGHFDHPKGSRSGVTVTVRASKDL--TAG--QEILHCYGPHRS-RMEVKE--RQRLLLEQYFFQCVCQAC      42 Xtropicalis(NP_001072288.1-SMYD4)/212-545                12.2%     --------------------RFVTVRANRPI--RRD--EEVTHCYGPHKL-RMDVAE--RQQLLKDQYFFVCQCKAC      43 Hsapiens(Q8IYR2-SMYD4)/244-602                           11.9%     --------------------TVATIRASQRI--RKG--QEILHCYGPHKS-RMGVAE--RQQKLRSQYFFDCACPAC      44 Ggallus(NP_001025886.1-SMYD4)/241-573                    11.1%     --------------------TAATVRASQPI--PSG--QEIFHCYGEEM----------------------------      45 Hmagnipapillata(XP_002160254.2/232-532                   13.1%     --------------------NQLIVKVVKDV--VKG--EQIFNCYGPHFK-RMGYED--RRAALMQQYFFLCSCEHC      46 Dpulex(DAPPUDRAFT_312722-Pedicted)/241-525               14.2%     --------------------NTLIVRAIRNV--RQG--DEVFNCYGPHYR-RMRRSE--RVEALEAQYSFTCTCDSC      47 Amellifera(XP_006565387.1-SMYD4-like-Predicted)/278-571  16.8%     --------------------QYLIVRASRDI--SQG--EEIFNCYGPHYR-HMTTEN--RQKILKNQYCFICKCKAC      48 Bfloridae(XP_002589088.1-BRAFLDRAFT_75068)/251-714       10.4%     --------------------KTLIVRAVSHI--KPG--EELLHCYGPHAG-RMVYGE--RQAALKEQYFFSCSCDAC      49 Lgigantea(LOTGIDRAFT_169490)/248-638                     11.5%     --------------------DTLIVKSVKKV--LEG--EEIYNCYGPHHK-RMVRKR--RQEVLENQYFFHCKCPPC      50 Skowalevskii(XP_002733823.1)/75-447                      10.9%     --------------------NQLTIRATEMI--KKG--GEVLHCYGPRVS-DMFRDE--RLKVLRDQYYFTCKCMFC      51 Nvectensis(XP_001627273.1)/170-547                       12.5%     --------------------GVLVARATHNI--APG--SGITHCYGPHVN-HMPREE--RQKLLYKQYFFTCQCSAC      52 Amellifera(XP_003250668.1-SMYD4-like-Predicted)/183-473  12.1%     --------------------NVLVLRAAKDI--YPG--ELITFNFMSKYV-ALESNSMPRNVMLKNFFDISCDCEAC      53 Amellifera(XP_001121272.2-SMYD4-like-Predicted)/230-549  13.9%     --------------------GIVVVRTLRFV--GKG--TEILDCYGPHWF-SENKLS--RIEYLWKKYRFLCTCDAC      54 Amellifera(XP_003249162.1-SMYD4-like-Predicted)/239-589  11.5%     --------------------RCIVIRAIRSL--RPG--DVVAENYGPIFT-KRNLEE--RRRNLAGRYWFFCECNAC      55 Dmelanogaster(CG14122-NP_648574.1)/265-541               14.2%     --------------------KKLVLTATRPH--RAN--ELVAVNYGPIFI-KNNLKE--RQRSLRGRYSFSCSCMAC      56 Agambiae(XP_311885.3-AGAP002999-PA)/268-544              13.9%     --------------------TAMILHTSRPI--PAG--AVVPENYGPHFM-RQPKAI--RQRNLRSRYWFKCDCRAC      57 Amellifera(XP_392262.3-SMYD4-like-Predicted)/252-555     15.0%     --------------------TTMVVRAIRSI--SSG--EEISENYGQIFT-TTPESE--RKRKLRLQYFFDCNCEAC      58 Dmelanogaster(CG7759-NP_725048.1)/250-537                15.6%     --------------------TTIHINSVRPI--EAG--LPINENYGPMYT-QDERSE--RQARLKDLYWFECSCDAC      59 Agambiae(XP_319583.4-AGAP008839-PA)/240-523              12.3%     --------------------NQVCVRTVKNI--PAD--SMVAENYGPLFT-QVRRDE--RRDTLLHQYRFTCQCVPC      60 Dpulex(DAPPUDRAFT_68494-Predicted)/254-551               14.7%     --------------------DVMVVRAIKSF--QPG--EMVNENYGPIFT-QKRRVD--RQRSLKDRYWFDCRCNPC      61 Dpulex(DAPPUDRAFT_309882)/300-599                        12.5%     --------------------TTMVITSTRCV--KRG--QMVAENYGPIFT-HKHLTD--RQQSLQGRYWFNCQCLAC      62 Dmelanogaster(CG8378-NP_610730.1)/196-491                13.8%     --------------------TKAYMFVLRPI--KAG--NVLYDNYGAHFA-ICSKEQ--RLKRLSLQYRFDCKCEGC      63 Agambiae(XP_566179.1-AGAP000216-PA)/158-458              13.6%     --------------------GRCSAVVIQPI--AAG--GQLFANYGYDYL-QTGRDE--RREGLQRVFGFTCNCDAC      64 Agambiae(XP_564258.1-AGAP011234-PA)/216-546              16.4%     --------------------GRLAMVATRPI--AAG--EQLYNINGFSTF-DPDDSA--RRHALQLSHFFKCRCASC      65 Agambiae(XP_309407.4-AGAP011238-PA)/219-497              15.4%     --------------------GRCAIVAIRPI--AAG--EQLFDNYG-------------------------------      66 Agambiae(XP_314169.4-AGAP005253-PB)/218-514              16.2%     --------------------GRCAFVATRPI--AAG--EQLFDVYAFASM-DFDRSF--RIFCLRKSYYFKCRCAVC      67 Agambiae(XP_309409.4-AGAP011237-PA)/206-481              15.2%     --------------------GRCAFVVSRPI--AAG--EQLFDVYG-------------------------------      68 Agambiae(XP_307865.2-AGAP009448-PA)/166-466              15.6%     --------------------GRCAMIVIRPI--GPG--EQLFDSYGLHHF-SFERSH--RQKGTFVMFNFECCCEAC      69 Agambiae(XP_309762.4-AGAP010931-PA)/113-383              14.4%     --------------------GRCAVIVIHTV--AKG--GQLFDNYE-------------------------------      70 Agambiae(XP_309378.2-AGAP011267-PA)/149-447              16.9%     --------------------GRCAVFVIRPV--LEG--SQLFDSYETDHK-SHERAM--RQLMLSFTYSFRCTCEAC      71 Agambiae(XP_309383.4-AGAP011257-PA)/149-447              16.4%     --------------------GRCAVFVIRPV--LEG--SQLFDSYEAGHT-LHEREM--RQSMLSFTYSFRCTCEAC      72 Agambiae(XP_307655.3-AGAP012638-PA)/149-447              16.4%     --------------------GRCAVFVIRPV--LEG--SQLFDSYEADHI-LNKRAM--RQSMLSFMYSFRCTCEAC      73 Agambiae(XP_320681.4-AGAP011835-PA)/183-484              14.6%     --------------------GRCAVLVTRPI--AKG--GQLYDNYGMHHC-LMSRKE--RKTELLKQYRFICECEAC      74 Agambiae(XP_309411.4-AGAP011232-PA)/162-434              14.4%     --------------------GRCALVVTRQI--ADG--GQLFDHYE-------------------------------      75 Amellifera(XP_001120776.2-SMYD4-like-Predicted)/251-554  13.4%     --------------------LTMITRALKPL--YPG--DQIFTSYGAAYA-YMTRSE--RREKIMQDYFFECDCIAC      76 Dpulex(DAPPUDRAFT_305694-Predicted)/258-553              16.3%     --------------------DVIAVKAIRRI--ARG--DEILDNYGYHYA-THDKKE--RQLKLSQQYYFRCNCLAC      77 Dpulex(EFX87901.1)/258-554                               14.2%     --------------------DVMVVKVIHRI--ARG--EEILDNYGYHYA-THEKRE--RQLKLCQQYYFRCRCQSC      78 Amellifera(XP_001122116.2-SMYD4-like-Predicted)/234-534  13.5%     --------------------NYMIIYVIYPI--KKG--EQLYDNYGQHYA-ITPKEE--RQKELLKQYYFKCNCLAC      79 Hmagnipapillata(XP_002159692.1)/239-485                  13.7%     --------------------DTCVLRAIKHI--KEG--SEIVDNYGFLYA-VESKVI--RQSHLMEQYYFACQCEAC      80 Nvectensis(XP_001623892.1)/215-512                       13.9%     --------------------DKCVVRAFSSI--PCR--GEVVDNYGILSA-LTPRKQ--RQESLQSQYYFKCNCHAC      81 Lgigantea(LOTGIDRAFT_143433)/100-395                     15.6%     --------------------DFCAVRAIRNI--PKG--TEVYDSYGALYP-LTAKKD--RQEKLLSQYFFKCSCKAC      82 Skowalevskii(XP_002740933.1)/253-549                     16.3%     --------------------ETCVVRAIRNI--YKG--EEITDNYGYLYP-VHDKSE--RQTRLKWQYFFECKCDAC      83 Dpulex(EFX73755.1)/49-306                                15.8%     --------------------FKLRIQLTQEL--PKLEWDSIRISYIDLM---NSKSH--RKKELKDRYYFDCDCPRC      84 Amellifera(XP_625013.1-SMYD3-Predicted)/1-253            15.0%     --------------------TTIIIRTTEDL--PCLDLSQIRISYIDVI---KTTKD--RREELQSSYYFWCNCKKC      85 Dmelanogaster(Buzidau-CG13761)/26-282                    14.4%     --------------------NELHVHAIEDM--ECLDWSKIFISYIDLL---NTPEQ--RRLDLKEHYYFLCVCSKC      86 Agambiae(XP_319707.4-AGAP008954-PA)/1-254                16.7%     --------------------ETLRMRLLEDYPEQELDFGKLFISYIDLI---DTAEV--RQEQLAERYYFHCACERC      87 cintestinalis(NP_001071820.1)/15-282                     18.6%     --------------------PRLEVRALRVI--QPG--EELCISYIDSL---ETTEK--RREKLKLQYYFDCECDTC      88 Drerio(Q6P0R5-Smyd1a)/18-279                             15.6%     DAS-------------FHSSRRIELRALEPI--SAG--QELTVSYVDFL---SVSTD--RQRLLQQQYYFDCKCEHC      89 Derio(Q2MJQ9-Smyd1b)/13-274                              15.9%     DTV-------------FHSQKRIELRALGKI--SAG--EEVTVAYVDYL---NVSAD--RQRLLKQQYFFDCTCKHC      90 Xtropicalis(NP_001120357.1-SMYD1)/13-261                 16.3%     ----------------------IELRALGKI--NKG--EELTVSYVDFL---NLTED--RKAQLKKQYYFDCTCEHC      91 Hsapiens(Q8NB12-SMYD1)/18-279                            16.1%     KSM-------------FHTQMRIELRALGKI--SEG--EELTVSYIDFL---NVSEE--RKRQLKKQYYFDCTCEHC      92 Ggallus(NP_989486.1-SMYD1)/13-274                        16.4%     RSM-------------FHTQMRIELRALSKI--SPG--DELTVSYVDFL---NVSEE--RRKQLKKQYYFDCTCEHC      93 Drerio(Q5RGL7-Smyd2b)/19-268                             15.0%     --------------------INAEVRAVKDI--SPG--QEIYTSYIDLL---YPTAD--RLERLRDMYYFSCDCKEC      94 Drerio(Q5BJI7-Smyd2a)/18-267                             15.2%     --------------------TVAEVRAVQEI--NPE--EEIFNSYIDLL---YPTED--RIERLKDSYFFNCDCKEC      95 Xtropicalis(XP_002934751.2-SMYD2-like-Predicted)/16-265  15.2%     --------------------TVAEVRAVQEI--HAG--DEVFTSYIDLL---YPTED--RNDRLIDSYFFNCDCREC      96 Hsapiens(Q9NRG4-SMYD2)/18-267                            16.1%     --------------------TLAEVRAVQEI--KPG--EEVFTSYIDLL---YPTED--RNDRLRDSYFFTCECQEC      97 Ggallus(XP_419420.1-SMYD2-Predicted)/21-270              14.7%     --------------------TLAEVRAVKEI--EPG--EEVFTSYIDLL---YPTED--RNDRLRDSYFFTCDCREC      98 cintestinalis(XP_002128556.1)/14-266                     18.0%     --------------------RTLKIRAVKNI--TAG--EEVLISYVDLF---ATSFE--RQRELMSIYHFQCTCHSC      99 Lgigantea(LOTGIDRAFT_177746)/1-216                       15.4%     --------------------KTVFIRALKDI--PDTTPNKMFISYIDQL---KPSVE--RLAELEEQYYFSCECSRC     100 Drerio(E7EZZ6-SMYD3)/16-267                              18.8%     --------------------KRLTLRAVRVI--RSA--EELTISYTDIL---APSKD--RRSQLQEQYHFRCECKRC     101 Xtropicalis(XP_004914684.1|-SMYD3-Predicted)/15-264      18.0%     --------------------TCLLLRTVKEI--PKG--EELTISYIDVK---MPTQG--RRDQLQRQYCFLCDCQRC     102 Hsapiens(Q9H7B4-SMYD3)/15-266                            17.4%     --------------------PHLLLRAVRDI--EVG--EELTICYLDML---MTSEE--RRKQLRDQYCFECDCFRC     103 Ggallus(XP_419536.1-SMYD3-Predicted)/15-266              18.0%     --------------------YQLLLRSIREI--QIG--EELTISYIESL---MPTSE--RQKQLKRQYCFECDCCLC     104 Tadhaerens(XP_002109888.1)/20-262                        19.2%     --------------------KQMQLRIIENT--KIG--DELLISYIDPM---QVLSS--RQNQLQSQYCFKCICERC     105 Hmagnipapillata(XP_002163555.2)/16-259                   17.2%     --------------------RDISIRAIKPI--AEG--EELMLSYISIL---ATSDV--RQLELRESYMFTCKCTVC     106 Nvectensis(XP_001627600.1)/17-253                        18.2%     --------------------TNIYIKALEEI--PVG--EELTISYIQQL---HPRET--RQEELQTQFCFYCQCHRC     107 Bfloridae(XP_002594889.1-BRAFLDRAFT_124463)/14-258       18.1%     --------------------LRMEVRAIQNI--QPG--EELLISYVEML---AMSSV--RKQQLLQQYYFTCKCPRC     108 Skowalevskii(XP_006817727.1)/14-260                      15.8%     --------------------TDLCIRAVKPI--SVG--DECVISYIEMM---STTSE--RREHLQDQYYFQCVCHAC         consensus/100%                                                     ......................h.......h.........h....................................         consensus/90%                                                                       . ...h.hhs...l  ..s  t.lh.sYh..h........  R...L...h.F.C.C..C         consensus/80%                                                                       . ..hhhlhshp.l  ..G  pplh.sYh..h. ...ptt  Rpt.L.tthhF.CpC.tC         consensus/70%                                                                          tthhlpAhpsI  ttG  pplh.sYh..h. .hsptp  Rpp.LpppYhFpCpCptC ``` |
