# Supplementary material for: Evolutionary History of the Smyd Gene Family in Metazoans: A Framework to Identify the Orthologs of Human Smyd Genes in Drosophila and Other Animal Species
Source: PLoS One. 2015 Jul 31;10(7):e0134106. doi: 10.1371/journal.pone.0134106 (PMC4521844; doi:10.1371/journal.pone.0134106)
Supplement: S2 File — Formatted in mview. (HTM) [file pone.0134106.s009.htm]

```
Reference sequence (1): Amellifera(XP_003250668.1SMYD4likePredicted)/183473/1-291
Identities normalised by aligned length.
Colored by: identity + property
```

|  |
| --- |
| ```                                                                       1 [        .         .         .         .         :         .         .         . 80   1 Amellifera(XP_003250668.1SMYD4likePredicted)/183473/1-291 100.0%     GRYFVAVKPIKMKDVILIDKSQITHLHKDD-------------WDDDP--TSNMCHYCFKYC----RALIPCDYCY-HAL      2 Dmelanogaster(CG8378NP_610730.1)/196491/1-296              18.8%     GRFVVTNRDLAVGDLVSVEEPFCSTLLTPM--------------------RYIRCATCKRENY---LTLIPCDSCC-STM      3 Agambiae(XP_566179.1AGAP000216PA)/158458/1-301             16.4%     GRYLQTNKALKVGDVVMIDEPYVSVLEPEF--------------------CYARCDHCQRPAP---FTLIPCERCT-KAM      4 Agambiae(XP_564258.1AGAP011234PA)/216546/1-331             17.2%     GRHLVTTQHLKAGDVLMIEKPYASLLCERD--------------------QYKRCAFCHNEDT---FTLIPCEGCT-VAM      5 Agambiae(XP_309407.4AGAP011238PA)/219497/1-279             17.8%     GRHLVTTQHLKAGDVLLIEKPYANLLIDVE--------------------RHVRCAFCQNEDR---FTLIPCEGCT-VTM      6 Agambiae(XP_314169.4AGAP005253PB)/218514/1-297             17.3%     GRHLVTTQHLKAGDVLLIEKPYASMLNDKE--------------------RYKRCAFCHNEDT---FTLIPCEGCT-LTM      7 Agambiae(XP_309409.4AGAP011237PA)/206481/1-276             16.6%     GRHLVTTQKLKVGDVLLIEKPYASMLNDQE--------------------RYKRCDFCQNEDR---FTLIPCEGCT-VTM      8 Agambiae(XP_307865.2AGAP009448PA)/166466/1-301             17.6%     GRHVVTTRRLKVGDVVMLDTPFVKTLHDPL--------------------RHVRCDFCHAERP---FTLIPCEGCT-WVM      9 Agambiae(XP_309762.4AGAP010931PA)/113383/1-271             16.7%     GRHVVTKRKLKVGDVVMIEKPFVTVAKETF--------------------QYIRCDFCQAKRL---FTLIPCEGCT-VAM     10 Agambiae(XP_309378.2AGAP011267PA)/149447/1-299             18.0%     GRHVVTTRKLKVGDVVMIERPFVTVLRDSL--------------------RYVRCDFCHEERP---FTLIPCEGCT-AAM     11 Agambiae(XP_309383.4AGAP011257PA)/149447/1-299             17.6%     GRHVVTTRKLKVGDVVMIERPFVTVLKDSF--------------------RYVRCDFCHGERP---FTLIPCEGCT-AAM     12 Agambiae(XP_307655.3AGAP012638PA)/149447/1-299             17.3%     GRHVVTTRKLKVGDVVMIERPFVTVLKDSF--------------------RYVRCDFCHGERP---FTLIPCEGCT-MAM     13 Agambiae(XP_320681.4AGAP011835PA)/183484/1-302             14.5%     GRYVATNRNLEAGDVVIIEQPFSRLLRDIY--------------------RHVRCDFCHRESI---FTLLPCENCT-VAM     14 Agambiae(XP_309411.4AGAP011232PA)/162434/1-273             16.4%     GRHVVATRQLRVGDVVMVEKPYATVLSDHM--------------------KRVRCAFCHAEEP---FLLIPCEECT-IAM     15 Amellifera(XP_001121272.2SMYD4likePredicted)/230549/1-320  15.9%     GRHLIATKNIKAGSVLIVETPFAFSTNKEA--------------------LGRNCLHCHITLMSSNSVKIPCYYCQ-TVS     16 Dpulex(DAPPUDRAFT_68494Predicted)/254551/1-298             19.0%     GRYGVAASPIRVGDVIAVDAPYASVMNPEK--------------------FSTHCHHCYQILEL--GEVLPCSHCD-LVS     17 Dpulex(DAPPUDRAFT_309882)/300599/1-300                     18.2%     GRYYVAADDIKPGQTLVCEKPYAACLLPGK--------------------FTSHCHHCFVRL----IAPLGCLTCR-GVF     18 Amellifera(XP_003249162.1SMYD4likePredicted)/239589/1-351  14.0%     GKRVIAAKNIEPGNRLIIESPHAAILLPEF--------------------FGTHCQHCFSRF----KAPIGCPDCS-SVA     19 Dmelanogaster(CG14122NP_648574.1)/265541/1-277             17.9%     GRFVVANEGLRTGDVLLFEEPVAACLEPSY--------------------FGTHCHHCFKRL----HTPVSCLHCS-GIA     20 Agambiae(XP_311885.3AGAP002999PA)/268544/1-277             15.7%     GRYVVAAADLGPGEVILTEPAYAACLHAKY--------------------YGTHCSACFSRL----IAPVACPDCC-GVA     21 Amellifera(XP_392262.3SMYD4likePredicted)/252555/1-304     19.7%     GRHAIATKDIEPGEILAIEKPYSAFLLAEY--------------------RLINCFYCFTKIFV--PIPAVCQTCS-CVA     22 Dmelanogaster(CG7759NP_725048.1)/250537/1-288              17.8%     GRFARASADVKPGEELLVERPFVSVLLEKF--------------------AKTHCENCFMRTVV--P--VACPRCA-DVL     23 Agambiae(XP_319583.4AGAP008839PA)/240523/1-284             16.4%     GRFARTNTDLKPNTILLLERPHVSVLLEDY--------------------SLDHCTHCFKRVSV--P--IACPLCA-DVV     24 Amellifera(XP_001120776.2SMYD4likePredicted)/251554/1-304  22.0%     GRHLVVTKEFKPGDIITIEDPYAYVIYTQR--------------------YYTHCHHCLSRS----YNLIPCLHCP-VAQ     25 Amellifera(XP_001122116.2SMYD4likePredicted)/234534/1-301  21.0%     GRHIVATRKINPGEVIAIEKPYSLILTPDN--------------------IYTHCSNCLEVS----WANIPCEYCT-YAM     26 Dpulex(DAPPUDRAFT_305694Predicted)/258553/1-296            16.8%     GRCLVATEDIQIGTTVIVEKALASILLEEF--------------------KESHCHHCLHWT----PGPVPCHQCS-QVG     27 Dpulex(EFX87901.1)/258554/1-297                            17.4%     GRCLVATEDIKIGETVIVEKAHASILQYEF--------------------KESHCHHCLHWT----PGPVPCHKCS-QVG     28 Hmagnipapillata(XP_002159692.1)/239485/1-247               17.0%     GRHAIASRDIKAGEVIIIEKPFASLCLPEC--------------------YNTHCYHCLTRF----KINYPCRLCS-TVN     29 Skowalevskii(XP_002740933.1)/253549/1-297                  20.3%     GRYAVATRDVKVGDVLIVENPYSSVGLQPC--------------------NVSHCHHCYIRV----LASIPCLQCA-GIV     30 Nvectensis(XP_001623892.1)/215512/1-298                    19.1%     GRHTIAARDINIGDVLLVEKPFASVLLQEQ--------------------SKSHCHQCFVHI----LAPLPCSYCT-TVR     31 Lgigantea(LOTGIDRAFT_143433)/100395/1-296                  14.2%     --GIYTTKDVEAGELLFCEKPFASKNMHNS--------------------DLTHCQNCLNRV----LSPLPCDQCS-GVV     32 Drerio(Q08C84Smyd4)/197556/1-360                           14.6%     GRHMLVMENKPAGEVVLEDEAYCSVLIPANIFNT----G-TNKAVETFGTEDRHCHHCLSQS----LSFVPCPKCS-YAR     33 Xtropicalis(NP_001072288.1SMYD4)/212545/1-334              18.9%     GRHLLASQNIEQGEVLIWEEAFASVIIPERK-----QWRKEIKWDTRITACDHYCHYCLNRV----IASLPCQYCS-FAR     34 Hsapiens(Q8IYR2SMYD4)/244602/1-359                         18.9%     GRCLVATKDILPGELLVQEDAFVSVLNPGELPP--PHHGLDSKWDTRVTNGDLYCHRCLKHT----LATVPCDGCS-YAK     35 Ggallus(NP_001025886.1SMYD4)/241573/1-332                  16.3%     GRHLVASQDILPGQNLLKEKAFVSVLCPGEGDSLLLQDSSETVWDTRVTNADLYCHHCLKQL----LASIPCCGCS-YAK     36 Cintestinalis(XP_002123001.1)/195567/1-373                 12.4%     GRHYFTTFNTETNECLLEEVAYLGVLNPEF--------------------FSTHCSYCLTPCK---SSGIPCLGCS-CTI     37 Dmelanogaster(CG1868NP_724802.1)/226549/1-324              17.3%     GRYMVAKEAISKGNVIFSERASCFVPLEQL----------------------LICQQCAATLM---SAPIPCPNCHQRVV     38 Agambiae(XP_319721.4AGAP008973PA)/165486/1-322             14.4%     GRYVVAAEAIKANDTVARETAVSFVPVYDPESS--------------STLPSFDCQKCAKVN----VVPFPCPTCG-RAC     39 Amellifera(XP_006565387.1SMYD4likePredicted)/278571/1-294  18.9%     GRHVIANKFIKEGDILFLEEPISFVLLNHD--------------------TYSYCQYCNNLNT---DIPVPCRTCL-NTF     40 Bfloridae(XP_002589088.1BRAFLDRAFT_75068)/251714/1-464     12.8%     GRMLVAQKAFEPGSVLIVEQPYAAVLLQKH--------------------HSTHCHTCVTPV----LVPHPCRGCQ-YVQ     41 Nvectensis(XP_001627273.1)/170547/1-378                    14.7%     GRFLQASSEIRAGDTLIAEEPYSAVLLPEN--------------------AKTHCECCYKSL----VAPVPCNHCS-SVL     42 Skowalevskii(XP_002733823.1)/75447/1-373                   13.9%     GRYILATETICRGEIIIKEKPYGCVLLPSH--------------------YNTRCYHCVRKT----VAPIPCCTCT-HVR     43 Dpulex(DAPPUDRAFT_312722Pedicted)/241525/1-285             15.8%     GRYVVANRDIKAGETLFVEQPNALVVLPDF--------------------QTSRCHHCTRHSS---AKRYPCLACG-KIW     44 Hmagnipapillata(XP_002160254.2/232532/1-301                18.7%     GRYIFAKEDIPNGSIIISEKPYAAVLLPHW--------------------YKTHCQLCFDKV----VSLFPCYECA-EVV     45 Lgigantea(LOTGIDRAFT_169490)/248638/1-391                  14.4%     GRYLTTNREIEVGDTLIVEKPFSSVLLPDH--------------------YKTHCHHCYHKLP---LNLVGCIQCS-VVR        consensus/100%                                                       ..hh.s...h..tp.l..-.s.................................C..C...........hsC..C...h.        consensus/90%                                                        GRhhhstptlt.Gphlh.-pshs.hh.....            ..........hCt.C......  ...hsC..Ct .s.        consensus/80%                                                        GRahlsspplpsG-hlhhEpsasshl..p.                    ..h+CphCht...   hs.lPC.tCs hsh        consensus/70%                                                        GRallsscplcsG-llhhEcPasslLh.p.                    h.s+CpaChpph.   hs.lPC.tCs hsh                                                                           81          .         1         .         .         .         .         :         . 160  1 Amellifera(XP_003250668.1SMYD4likePredicted)/183473/1-291 100.0%     YCS----------------------KECRGKAYQAYHQIYCRYGNLD--NK------------SSFVLKLLLKITDNGAR      2 Dmelanogaster(CG8378NP_610730.1)/196491/1-296              18.8%     FCS----------------------EECKSIAMQTYHRYECPIIDF--LNRM-----FNKI--HCIALRTTLVALNIFP-      3 Agambiae(XP_566179.1AGAP000216PA)/158458/1-301             16.4%     YCS----------------------KNCLRRARTEYHEFECALVHH--LTET-----TRDP-VVLLAWRAVTRAISTYRY      4 Agambiae(XP_564258.1AGAP011234PA)/216546/1-331             17.2%     YCS----------------------EECRDKAHKQYHRYECAVLRD--CWRS-----VGFPVEMLLGLRTVATAFASFDQ      5 Agambiae(XP_309407.4AGAP011238PA)/219497/1-279             17.8%     YCS----------------------EECRDKAHKQYHRYECGVLRD--CWRI-----VGHLFGGMVGLRTVATAIASFDQ      6 Agambiae(XP_314169.4AGAP005253PB)/218514/1-297             17.3%     YCS----------------------DECMDKAYKQYHRYECGVLRD--CWRI-----AGRLVGGIVGLRMVATAIASFEQ      7 Agambiae(XP_309409.4AGAP011237PA)/206481/1-276             16.6%     YCS----------------------KECMDKAHKQYHRYECGVLRD--CWRM-----IGSLPGGIMGLRTVATAFASFEQ      8 Agambiae(XP_307865.2AGAP009448PA)/166466/1-301             17.6%     YCS----------------------AECLGKAYSQYHRYECGVMRD--LWRV-----AGKC--PMTAVRTVASAFGTFDD      9 Agambiae(XP_309762.4AGAP010931PA)/113383/1-271             16.7%     YCS----------------------EECISKAYGKYHRYECGVLRD--LWTV-----LGIS--GVIALRMIAIAITTFDN     10 Agambiae(XP_309378.2AGAP011267PA)/149447/1-299             18.0%     YCS----------------------EECLSKAYNKYHRYECGLLRD--MVEV-----FDEL--PLIAIRMIAIAITTFDN     11 Agambiae(XP_309383.4AGAP011257PA)/149447/1-299             17.6%     YCS----------------------EECLSKAYNNYHRYDCGILRD--LYED-----FEEV--SLIDIRMIAIAITTFDN     12 Agambiae(XP_307655.3AGAP012638PA)/149447/1-299             17.3%     YCS----------------------EECLSKAYNKYHRYECGLLRD--MWEV-----FEEV--SLIDIRMIAIAITTFDN     13 Agambiae(XP_320681.4AGAP011835PA)/183484/1-302             14.5%     YCS----------------------GSCASQAARQYHRYECPIIRD--MWRI-----FTKL--PVMSLRTVTTAISAFEY     14 Agambiae(XP_309411.4AGAP011232PA)/162434/1-273             16.4%     YCS----------------------QKCLRAAWQQYHRYECPILND--MRTI-----GTEY--LALAVRTVAIALASFDH     15 Amellifera(XP_001121272.2SMYD4likePredicted)/230549/1-320  15.9%     FCS----------------------EKCRSKAWQIYHQYECFIFDV--FFENDSEQIQRNTSYLLLAYRMIISGFLSSTE     16 Dpulex(DAPPUDRAFT_68494Predicted)/254551/1-298             19.0%     FCS----------------------VNCRSRAMEIYHAIECPILSC--LYAA------GISIICYLSLRMIAIHPPSF--     17 Dpulex(DAPPUDRAFT_309882)/300599/1-300                     18.2%     YCS----------------------VECRDEAASTYHQYECGIIDY--MIAS------GSSILSWIALRILTKGKMED--     18 Amellifera(XP_003249162.1SMYD4likePredicted)/239589/1-351  14.0%     FCG----------------------RKCRDTALASYHKYECKILVL--LIGS------GMSVLSMLALRMATQVGPAG--     19 Dmelanogaster(CG14122NP_648574.1)/265541/1-277             17.9%     FCS----------------------AQCMGEACSSYHRFECEYMDL--MIGS------GMSILCFIALRIFTQAPSLE--     20 Agambiae(XP_311885.3AGAP002999PA)/268544/1-277             15.7%     FCS----------------------VACRDKACATYHRFECQYLDL--MIGS------GMSILCHVALRMVTQAGTPE--     21 Amellifera(XP_392262.3SMYD4likePredicted)/252555/1-304     19.7%     YCS----------------------ISCRDKD-AKIHENECSILPT--LWAS------KTSINCFLALRIIVQQSFEK--     22 Dmelanogaster(CG7759NP_725048.1)/250537/1-288              17.8%     YCS----------------------EQCREEASKKYHKYECGIVPI--IWRS------GASINNHIALRIIASKPLDY--     23 Agambiae(XP_319583.4AGAP008839PA)/240523/1-284             16.4%     FCS----------------------DECETKANATYHRYECGFLPI--LWGS------GASITCHMALRMITQKSEEY--     24 Amellifera(XP_001120776.2SMYD4likePredicted)/251554/1-304  22.0%     YCS----------------------EKCRILAWEMAHDIECPIMAL--IGNLLHVD-KDKIRMLTKIIRFLIIATAKGKN     25 Amellifera(XP_001122116.2SMYD4likePredicted)/234534/1-301  21.0%     YCS----------------------EECKAMEWKKYHDIECAIFPS--MLKM------NFVKLDLFSLRLAIQAVREATS     26 Dpulex(DAPPUDRAFT_305694Predicted)/258553/1-296            16.8%     FCS----------------------TLCRDEAWASYHQSECGLTDS--LH----GT--NVGRHGLLAVRTVLKVGRQR--     27 Dpulex(EFX87901.1)/258554/1-297                            17.4%     FCS----------------------TQCRDEAWDSYHQFECGLTDF--LCRTTRDV--NTGQHGLLALRTVLKADRRL--     28 Hmagnipapillata(XP_002159692.1)/239485/1-247               17.0%     YCS----------------------ISCEKESWEKFHCFECEYLGV--LIND-------DVGLAHLAFKIITNVGISM--     29 Skowalevskii(XP_002740933.1)/253549/1-297                  20.3%     YCS----------------------KECRNASWEMYHNLECHHLDL--IQEL-------GLGMGHLALRTIIRTGLAF--     30 Nvectensis(XP_001623892.1)/215512/1-298                    19.1%     YCS----------------------EKCAKESWDAYHYAECMNLEH--VY-V-------AGKYGHLALRVVVKAGFQY--     31 Lgigantea(LOTGIDRAFT_143433)/100395/1-296                  14.2%     FCS----------------------EECKAEAMKSFHFAECRVLET--IHNI-------DFGLGHLALRMVLKAGLNH--     32 Drerio(Q08C84Smyd4)/197556/1-360                           14.6%     YCG----------------------ESCQKDAWDQWHQWECPVGAD--LLA--------IGVLGHLALRVVLKAGQTEVQ     33 Xtropicalis(NP_001072288.1SMYD4)/212545/1-334              18.9%     YCS----------------------QECMDKAWRSYHYIECSMGDL--LLA--------LGMFCHTALRAVLVAGCRLFS     34 Hsapiens(Q8IYR2SMYD4)/244602/1-359                         18.9%     YCS----------------------QECLQQAWELYHRTECPLGGL--LLT--------LGVFCHIALRLTLLVGFEDVR     35 Ggallus(NP_001025886.1SMYD4)/241573/1-332                  16.3%     YCS----------------------QNCADVAWEQYHRTECPLGAL--LLT--------LGVFFHVALRTVLLAGFSEVS     36 Cintestinalis(XP_002123001.1)/195567/1-373                 12.4%     YCD----------------------EQCRISAWKIYHWMECSVIPM--LAI--------KCMELRVAVRALLTGAYELGE     37 Dmelanogaster(CG1868NP_724802.1)/226549/1-324              17.3%     YCS----------------------RKCRE-AHSAIHKFECAAYRKDILRL--------LG-ISHLALRLLLTYIPYIRP     38 Agambiae(XP_319721.4AGAP008973PA)/165486/1-322             14.4%     YCS----------------------TRCRV-AHRPVHRFECFGYQKHLWYQ--------IG-IAHLGLRCFLDGFGTI--     39 Amellifera(XP_006565387.1SMYD4likePredicted)/278571/1-294  18.9%     YCN----------------------ENCLTKAWSSYHCWECPGNQMNLWKE--------IG-IGHLALKVLLTCSTITDK     40 Bfloridae(XP_002589088.1BRAFLDRAFT_75068)/251714/1-464     12.8%     YCSGTCEEQAWREYHRGCQYVQYCSRTCEDQAWKEYHSYECEHWHL--LQM--------VETFAQLSLRLLLTAAARGEK     41 Nvectensis(XP_001627273.1)/170547/1-378                    14.7%     YCS----------------------AACRNKAWSQYHHVECEIFPV--LEI--------VDTFTHLSLRILLTTSAKDII     42 Skowalevskii(XP_002733823.1)/75447/1-373                   13.9%     YCS----------------------VECQQESWKSYHYIECPLWPF--LSQ--------AGNFSQLSLRILLKAGWSNIQ     43 Dpulex(DAPPUDRAFT_312722Pedicted)/241525/1-285             15.8%     FCS----------------------DSCRQES-SCYHNFECGLEAV--LNS--------VG-IAHLGARIVLSHGLDSVL     44 Hmagnipapillata(XP_002160254.2/232532/1-301                18.7%     FCS----------------------LSCYNDAWATYHRFECKKLSL--MEK--------VG-IAHLSLRIVLVSDAKDLL     45 Lgigantea(LOTGIDRAFT_169490)/248638/1-391                  14.4%     YCS----------------------SKCQEESWKLYHSVECPYLDL--LHS--------VG-IAHLSLRTVLTAGLQFLT        consensus/100%                                                       aCs........................C...t.t.hH...C........................h.h+hhh........        consensus/90%                                                        aCS                      .pC.t.uht.aH.hEC.hh..  h...     .......hhulRhhh.h......        consensus/80%                                                        aCS                      tpChppAhptYHphEC.hht.  hht.     ......shlulRhlh.sh.p...        consensus/70%                                                        YCS                      ppChscAappYHphECshht.  hht.     ..hs.hshluLRhlhpshhph..                                                                          161          .         .         .         2         .         .         .         . 240  1 Amellifera(XP_003250668.1SMYD4likePredicted)/183473/1-291 100.0%     LKEALEYHKEL-------ENMS--------------------------EEM-----------------------------      2 Dmelanogaster(CG8378NP_610730.1)/196491/1-296              18.8%     SIEELIDFCEQ------EQNQD----------------------------------------------------------      3 Agambiae(XP_566179.1AGAP000216PA)/158458/1-301             16.4%     NLRHLKQRRNY------LSRTE----------------------------------------------------------      4 Agambiae(XP_564258.1AGAP011234PA)/216546/1-331             17.2%     SLGQWIYRMET------LDETK----------------------------------------------------------      5 Agambiae(XP_309407.4AGAP011238PA)/219497/1-279             17.8%     DLEGWNDHLNT------LDETN----------------------------------------------------------      6 Agambiae(XP_314169.4AGAP005253PB)/218514/1-297             17.3%     DLEGWTNHLNA------LDETK----------------------------------------------------------      7 Agambiae(XP_309409.4AGAP011237PA)/206481/1-276             16.6%     DLEGWIDHLNT------LDEAK----------------------------------------------------------      8 Agambiae(XP_307865.2AGAP009448PA)/166466/1-301             17.6%     DPDALQAHLDA------LDEPQ----------------------------------------------------------      9 Agambiae(XP_309762.4AGAP010931PA)/113383/1-271             16.7%     DLEKLKDHLDA------LDESK----------------------------------------------------------     10 Agambiae(XP_309378.2AGAP011267PA)/149447/1-299             18.0%     NPEALKDHLDV------LDESN----------------------------------------------------------     11 Agambiae(XP_309383.4AGAP011257PA)/149447/1-299             17.6%     NPEALKDHLDA------LDESN----------------------------------------------------------     12 Agambiae(XP_307655.3AGAP012638PA)/149447/1-299             17.3%     NPEALKDHLDA------LDESN----------------------------------------------------------     13 Agambiae(XP_320681.4AGAP011835PA)/183484/1-302             14.5%     DLQEMWEHLQV------LEKAK----------------------------------------------------------     14 Agambiae(XP_309411.4AGAP011232PA)/162434/1-273             16.4%     DLEALRAHLSH------LDVSK----------------------------------------------------------     15 Amellifera(XP_001121272.2SMYD4likePredicted)/230549/1-320  15.9%     QI---KNIEKK--KISFLNNNF----------------------------------------------------------     16 Dpulex(DAPPUDRAFT_68494Predicted)/254551/1-298             19.0%     FMD-VRPVIEQ--PELQK--------------------------------------------------------------     17 Dpulex(DAPPUDRAFT_309882)/300599/1-300                     18.2%     FLE-AREELEK---------------------------------------------------------------------     18 Amellifera(XP_003249162.1SMYD4likePredicted)/239589/1-351  14.0%     CLR-IHRALNR--QDSAADGEEIAESSTITTT---------------AEKLS---------------------KSAKRRS     19 Dmelanogaster(CG14122NP_648574.1)/265541/1-277             17.9%     QG------------------------------------------------------------------------------     20 Agambiae(XP_311885.3AGAP002999PA)/268544/1-277             15.7%     KV------------------------------------------------------------------------------     21 Amellifera(XP_392262.3SMYD4likePredicted)/252555/1-304     19.7%     LYK-LKDVKEN--SKDKFEVSA----------------------------------------------------------     22 Dmelanogaster(CG7759NP_725048.1)/250537/1-288              17.8%     FLK-LKPTIDE--E---LTPEQ----------------------------------------------------------     23 Agambiae(XP_319583.4AGAP008839PA)/240523/1-284             16.4%     FLK-LKPELA---G---LTNEQ----------------------------------------------------------     24 Amellifera(XP_001120776.2SMYD4likePredicted)/251554/1-304  22.0%     INE-LRADMKL--AESNPDNRT----------------------------------------------------------     25 Amellifera(XP_001122116.2SMYD4likePredicted)/234534/1-301  21.0%     IQE-LRKELEE--VDSCEDPRT----------------------------------------------------------     26 Dpulex(DAPPUDRAFT_305694Predicted)/258553/1-296            16.8%     IMD-VASEDDG------CN-------------------------------------------------------------     27 Dpulex(EFX87901.1)/258554/1-297                            17.4%     III-ANEQEKS------PE-------------------------------------------------------------     28 Hmagnipapillata(XP_002159692.1)/239485/1-247               17.0%     LLS-FKENNS----------------------------------------------------------------------     29 Skowalevskii(XP_002740933.1)/253549/1-297                  20.3%     LLK-FREQSA---NVNIPDESF----------------------------------------------------------     30 Nvectensis(XP_001623892.1)/215512/1-298                    19.1%     LKA-SVKQFES--EEKKCDPAE----------------------------------------------------------     31 Lgigantea(LOTGIDRAFT_143433)/100395/1-296                  14.2%     ILQ-NNKKYP---ESFRSDILR----------------------------------------------------------     32 Drerio(Q08C84Smyd4)/197556/1-360                           14.6%     MGI--KNTKDHVT----TYKNDSPV-------------QLS---------------------------------------     33 Xtropicalis(NP_001072288.1SMYD4)/212545/1-334              18.9%     QSL--EQTGSADA----TDKTKVCN-------------------------------------------------------     34 Hsapiens(Q8IYR2SMYD4)/244602/1-359                         18.9%     KII--TKLCDKI------SNKDICLPESNNQV---KTLNYG---------LGE---------------------------     35 Ggallus(NP_001025886.1SMYD4)/241573/1-332                  16.3%     RLV--EWS-RDD------SNKDLCNAEAGGEH---PSEA-----------------------------------------     36 Cintestinalis(XP_002123001.1)/195567/1-373                 12.4%     TPQ--HDTTHTC-----TSIAKHIYQSR-NSC------------------------------------------------     37 Dmelanogaster(CG1868NP_724802.1)/226549/1-324              17.3%     HL---QEMTS----------AKGMWEEIMNLS------------------------------------------------     38 Agambiae(XP_319721.4AGAP008973PA)/165486/1-322             14.4%     --A--GEMAK-------ATDASVCYQRVLEAT------------------------------------------------     39 Amellifera(XP_006565387.1SMYD4likePredicted)/278571/1-294  18.9%     --------------------------------------------------------------------------------     40 Bfloridae(XP_002589088.1BRAFLDRAFT_75068)/251714/1-464     12.8%     HPS--ADMESPATASKPSDQAKLCTDKVSPTSDGAKTVQIDSETGSTSEQPGDLSVQTDVIEENPPSAGMESPTTADKPS     41 Nvectensis(XP_001627273.1)/170547/1-378                    14.7%     DVL--NGLSR----DVATTSCSL------PGC------------------------------------------------     42 Skowalevskii(XP_002733823.1)/75447/1-373                   13.9%     KYS--KEVSN------PVSSSHI------PGC------------------------------------------------     43 Dpulex(DAPPUDRAFT_312722Pedicted)/241525/1-285             15.8%     AFL--KDTDK---------VKKV------PGI------------------------------------------------     44 Hmagnipapillata(XP_002160254.2/232532/1-301                18.7%     RFL--GSDLNKFTDSPTLPSSKI------EGC------------------------------------------------     45 Lgigantea(LOTGIDRAFT_169490)/248638/1-391                  14.4%     DFI--KERKD--DESKKTANSRL------PGL------------------------------------------------        consensus/100%                                                       ................................................................................        consensus/90%                                                        .........t......................                                                        consensus/80%                                                        .....tt..p.  ..........      ...                                                        consensus/70%                                                        .h...ppthp.  .  ..p.tp.                                                                                                                                   241          :         .         .         .         .         3         .         . 320  1 Amellifera(XP_003250668.1SMYD4likePredicted)/183473/1-291 100.0%     --------------------------------------------------EK--KV---YNLKEMKENNLR-SILNLSIP      2 Dmelanogaster(CG8378NP_610730.1)/196491/1-296              18.8%     -----------------------------------------------------KCAFDLNYNELTPEEHYR-AIHGLVT-      3 Agambiae(XP_566179.1AGAP000216PA)/158458/1-301             16.4%     -----------------------------------------------------VNPLMLNWVDGQK-IAFS-AVYILAS-      4 Agambiae(XP_564258.1AGAP011234PA)/216546/1-331             17.2%     -----------------------------------------------------VNAFTVDWNKATDRDIYD-TVHVLAT-      5 Agambiae(XP_309407.4AGAP011238PA)/219497/1-279             17.8%     -----------------------------------------------------VNAFTMDWKNATVSDIYD-TVHVLAT-      6 Agambiae(XP_314169.4AGAP005253PB)/218514/1-297             17.3%     -----------------------------------------------------VNAFTVDWNKVTDSDIYD-TVHVLAT-      7 Agambiae(XP_309409.4AGAP011237PA)/206481/1-276             16.6%     -----------------------------------------------------VNAFTVDWNEITDSDMYD-TVHVLAT-      8 Agambiae(XP_307865.2AGAP009448PA)/166466/1-301             17.6%     -----------------------------------------------------VNGFTMDWRTATPKDVYS-TVHVLST-      9 Agambiae(XP_309762.4AGAP010931PA)/113383/1-271             16.7%     -----------------------------------------------------VDGFTMDWKKATLQDVFN-TVHVLCT-     10 Agambiae(XP_309378.2AGAP011267PA)/149447/1-299             18.0%     -----------------------------------------------------VNGFTMDWNKATQQDIFN-TVHVLTT-     11 Agambiae(XP_309383.4AGAP011257PA)/149447/1-299             17.6%     -----------------------------------------------------VNGFTMDWNKATQQDIFN-TVHVLTT-     12 Agambiae(XP_307655.3AGAP012638PA)/149447/1-299             17.3%     -----------------------------------------------------VNGFTMDWNKATQQDIFN-TVHVLTT-     13 Agambiae(XP_320681.4AGAP011835PA)/183484/1-302             14.5%     -----------------------------------------------------VNAFTMDWTSASAKDIYD-TVHVLET-     14 Agambiae(XP_309411.4AGAP011232PA)/162434/1-273             16.4%     -----------------------------------------------------VNAFEMDWRAASPRTVYE-TVYSLAT-     15 Amellifera(XP_001121272.2SMYD4likePredicted)/230549/1-320  15.9%     --------------------------LQ------------YYVT--NINKER--SNL--GTNEIYSPYDYR-TILNLET-     16 Dpulex(DAPPUDRAFT_68494Predicted)/254551/1-298             19.0%     -----------------------------------------------------------KAALSEDVKKYI-KTYHLVT-     17 Dpulex(DAPPUDRAFT_309882)/300599/1-300                     18.2%     ---------------------------------------------------DGDGGR--LLASARNPDSYS-GIYHLAT-     18 Amellifera(XP_003249162.1SMYD4likePredicted)/239589/1-351  14.0%     RRKKLRDS-R-----RA----KGEETVE------------EERR--EIKGEEGEDGE--KM----AENVDL-RVYDLVT-     19 Dmelanogaster(CG14122NP_648574.1)/265541/1-277             17.9%     -----------------------------------------------------------------LATANL-LFEHLCS-     20 Agambiae(XP_311885.3AGAP002999PA)/268544/1-277             15.7%     -----------------------------------------------------------------LEEGKM-LRDTFCA-     21 Amellifera(XP_392262.3SMYD4likePredicted)/252555/1-304     19.7%     -------------------------------------------------------------SEPYRSNDFK-IMFRLVT-     22 Dmelanogaster(CG7759NP_725048.1)/250537/1-288              17.8%     -------------------------------------------------------------LISLPKDDFR-RVAQLER-     23 Agambiae(XP_319583.4AGAP008839PA)/240523/1-284             16.4%     -------------------------------------------------------------IDKLPVDDYR-KVYKLVT-     24 Amellifera(XP_001120776.2SMYD4likePredicted)/251554/1-304  22.0%     ------------------------------------------------------AGF--TDEDILDSTSAR-SALSLAT-     25 Amellifera(XP_001122116.2SMYD4likePredicted)/234534/1-301  21.0%     ------------------------------------------------------KGF--SKNGMFLSDKYR-SLLGLIT-     26 Dpulex(DAPPUDRAFT_305694Predicted)/258553/1-296            16.8%     --------------------------------------------------------P--AGGELYDSTDYGRTIHRLVG-     27 Dpulex(EFX87901.1)/258554/1-297                            17.4%     --------------------------------------------------------S--FASQVFDSANYD-TVHRLVD-     28 Hmagnipapillata(XP_002159692.1)/239485/1-247               17.0%     --------------------------------------------------------F--DDLKPYSSTDYN-SIFSLIE-     29 Skowalevskii(XP_002740933.1)/253549/1-297                  20.3%     ------------------------------------------------------HGC--TVDGEYES-NYY-SVYNLVG-     30 Nvectensis(XP_001623892.1)/215512/1-298                    19.1%     ------------------------------------------------------LGC--NPDGVYDPSDYR-PIYHLVG-     31 Lgigantea(LOTGIDRAFT_143433)/100395/1-296                  14.2%     ------------------------------------------------------IGF--NKDGVYDSMDYD-TVYSLVK-     32 Drerio(Q08C84Smyd4)/197556/1-360                           14.6%     -----------------------------------------LG---GDCGKSLD------HTDCFHGSSYM-GIYSLLP-     33 Xtropicalis(NP_001072288.1SMYD4)/212545/1-334              18.9%     ------------------------------------------------------SK---STYHEKYCSSYQ-SVVNLLP-     34 Hsapiens(Q8IYR2SMYD4)/244602/1-359                         18.9%     -------S--------------------------------EKN---GNIVETPIPG---CDINGKYENNYN-AVFNLLP-     35 Ggallus(NP_001025886.1SMYD4)/241573/1-332                  16.3%     -----------------------------------------LD---TRAGRKVIPG---CNDNGQYQSSYQ-AVFNLLP-     36 Cintestinalis(XP_002123001.1)/195567/1-373                 12.4%     ------AD-----------------------------------------CSEPVGT---DPKDGVYKCDYW-SIFCLKT-     37 Dmelanogaster(CG1868NP_724802.1)/226549/1-324              17.3%     -----------------------------------------------RK-----------PEESENAPEYL-RSLRMVS-     38 Agambiae(XP_319721.4AGAP008973PA)/165486/1-322             14.4%     ------------------------------------------------------------REEDNPFSHYG-RVLRLVT-     39 Amellifera(XP_006565387.1SMYD4likePredicted)/278571/1-294  18.9%     -------------------------------------------------------------------IKFN-EMQNLIT-     40 Bfloridae(XP_002589088.1BRAFLDRAFT_75068)/251714/1-464     12.8%     DQNKLCTDDLSSTSEEAKTDSETESTLEQASNMSQAESTPEQASNLSVKTDSVQNS---MQDVELHRGNYS-SVYNLMT-     41 Nvectensis(XP_001627273.1)/170547/1-378                    14.7%     ------------------------------------------------------------TVSGSYPGDYG-SVFSLVT-     42 Skowalevskii(XP_002733823.1)/75447/1-373                   13.9%     ------------------------------------------------------------DTRGNYKSDYN-SIYSLIT-     43 Dpulex(DAPPUDRAFT_312722Pedicted)/241525/1-285             15.8%     ------------------------------------------------------------DG-PYDTKSYQ-VMFHLVS-     44 Hmagnipapillata(XP_002160254.2/232532/1-301                18.7%     ------------------------------------------------------------NDQGIYQGDYE-SVYFLST-     45 Lgigantea(LOTGIDRAFT_169490)/248638/1-391                  14.4%     ------------------------------------------------------------NERGKYERSYD-TVYYLMT-        consensus/100%                                                       ............................................................................h...        consensus/90%                                                                                                 ..   .......................h. .hh.L.s         consensus/80%                                                                                                             .........t...tt.at tlh.Lhs         consensus/70%                                                                                                             ....  ..tp.h.pstYp slatLho                                                                           321          .         .         :         .         .         .         .         4 400  1 Amellifera(XP_003250668.1SMYD4likePredicted)/183473/1-291 100.0%     MKKDDNRTEDNLFYSAKIAML----LRNHS---NYM--------------------------------------------      2 Dmelanogaster(CG8378NP_610730.1)/196491/1-296              18.8%     --NQHLRSVSDLFQRSVVCAVLKHFIIEYTPVKEYL----GG--------------------------------------      3 Agambiae(XP_566179.1AGAP000216PA)/158458/1-301             16.4%     --LARAPNDPVEARVAQISREMHCHLVSEN---GQTANDDSG--------------------------------------      4 Agambiae(XP_564258.1AGAP011234PA)/216546/1-331             17.2%     --NQNRRDHKQLASLIFFAYIVQGLLLDRT-ELRPLCW--SC--------------------------------------      5 Agambiae(XP_309407.4AGAP011238PA)/219497/1-279             17.8%     --NQKRRSRKDLAELIFFASIVHRLLLERT-DFGPLCE--SN--------------------------------------      6 Agambiae(XP_314169.4AGAP005253PB)/218514/1-297             17.3%     --NQKRRSREDLAVLMFFTSIVHRLLLERT-DLGPFCE--SS--------------------------------------      7 Agambiae(XP_309409.4AGAP011237PA)/206481/1-276             16.6%     --NQKRRSCKDLAMLIFFASIVHRLLLERT-ELGTLCE--SN--------------------------------------      8 Agambiae(XP_307865.2AGAP009448PA)/166466/1-301             17.6%     --NQERRPFMQLVFMVYLAIIIHKLMLERT-ELGPRSR--AK--------------------------------------      9 Agambiae(XP_309762.4AGAP010931PA)/113383/1-271             16.7%     --NQERRNIKELAGLTFFTVVMHNHLLEWT-ELGPACE--AN--------------------------------------     10 Agambiae(XP_309378.2AGAP011267PA)/149447/1-299             18.0%     --NQERRDSNFLAFHIFNATILHTLVLERT-ELGPVCE--AN--------------------------------------     11 Agambiae(XP_309383.4AGAP011257PA)/149447/1-299             17.6%     --NQERRHSMFVAMFIFNATILHTLILERT-ELGPVCE--AN--------------------------------------     12 Agambiae(XP_307655.3AGAP012638PA)/149447/1-299             17.3%     --NQERRDSFFVAFYIFNATILHTLVLERT-ELGPVCE--AN--------------------------------------     13 Agambiae(XP_320681.4AGAP011835PA)/183484/1-302             14.5%     --NERTRDRKDRMVRVFYTTIIYRLLEERCPELGELCA--MN--------------------------------------     14 Agambiae(XP_309411.4AGAP011232PA)/162434/1-273             16.4%     --NQRKRARKDFALNVLVAMITHKLLLKRTPA-AQVCG--AD--------------------------------------     15 Amellifera(XP_001121272.2SMYD4likePredicted)/230549/1-320  15.9%     --HCTKMEPKTNLIRAIEAIFLAKCFTFVL---SKMD-------------------------------------------     16 Dpulex(DAPPUDRAFT_68494Predicted)/254551/1-298             19.0%     --HDTLRNKESFFHVTLMANFLLKCLKVA----GYFGTRDTT-D------------------------------------     17 Dpulex(DAPPUDRAFT_309882)/300599/1-300                     18.2%     --LSHLRSDKDFFDRTFMALFLFQCLRAS----GYLQTRFRY-EE-----------------------------------     18 Amellifera(XP_003249162.1SMYD4likePredicted)/239589/1-351  14.0%     --HEKRRTAKDFFERSLMAAFLFKCLQKV----GFFDNPSSN-E------------------------------------     19 Dmelanogaster(CG14122NP_648574.1)/265541/1-277             17.9%     --HEEDRQPDDYLRRALMSGFLLRILQKS----LYFGRRKTE-G------------------------------------     20 Agambiae(XP_311885.3AGAP002999PA)/268544/1-277             15.7%     --HTEHRDPEDHFKRTLMTAFLLRCLQKA----EFFGRRTTE-A------------------------------------     21 Amellifera(XP_392262.3SMYD4likePredicted)/252555/1-304     19.7%     --HEDTRTVEDLFHRTYIASWLLRLLKKG----PYFPKHVKTPDT-----------------------------------     22 Dmelanogaster(CG7759NP_725048.1)/250537/1-288              17.8%     --HQGERQPSNFFQHVLMARFLTNCLRAG----GYFGSEPK---------------------------------------     23 Agambiae(XP_319583.4AGAP008839PA)/240523/1-284             16.4%     --HESTRSPEDFFQRTLMATLLNACLTLG----GYGA---C---------------------------------------     24 Amellifera(XP_001120776.2SMYD4likePredicted)/251554/1-304  22.0%     --NMTMRPLIGISAFACISALAAILLATQT---NFFCNKYEVNQL-----------------------------------     25 Amellifera(XP_001122116.2SMYD4likePredicted)/234534/1-301  21.0%     --NTEKRSVQDLFRRSLDASFILYFLATCS---NMFGNPLKKDLS-----------------------------------     26 Dpulex(DAPPUDRAFT_305694Predicted)/258553/1-296            16.8%     --NTARRSVADLFRRAVMAVYLTSLIQQQ---------RDGK--------------------------------------     27 Dpulex(EFX87901.1)/258554/1-297                            17.4%     --NSSQRSTTDIFRRAVMAVYLTSLIQIR---------D--G--------------------------------------     28 Hmagnipapillata(XP_002159692.1)/239485/1-247               17.0%     --N-----------------------------------------------------------------------------     29 Skowalevskii(XP_002740933.1)/253549/1-297                  20.3%     --HSEDRKPGDLFKRVVKAVCLLRCLQQT---------NFFQSVG-----------------------------------     30 Nvectensis(XP_001623892.1)/215512/1-298                    19.1%     --HTHERTLNDLFVRTLNAIYLLRCLEGT---------EYYGDST-----------------------------------     31 Lgigantea(LOTGIDRAFT_143433)/100395/1-296                  14.2%     --HSEKRSLGDLFKRSVVAVFMVKCLEHT---------LSSQPLS-----------------------------------     32 Drerio(Q08C84Smyd4)/197556/1-360                           14.6%     --HVAQHSPASRFLMAITMAVIYGKLQGGP---PPNK-------------------------------------------     33 Xtropicalis(NP_001072288.1SMYD4)/212545/1-334              18.9%     --HTENHPAERKFLCGLTAAALYKKLCLIM---AKDLVSST---S-----------------------------------     34 Hsapiens(Q8IYR2SMYD4)/244602/1-359                         18.9%     --HTENHSPEHKFLCALCVSALCRQLEAAS---LQAIPTERI-VN-----------------------------------     35 Ggallus(NP_001025886.1SMYD4)/241573/1-332                  16.3%     --HVEKHSPEHKFLCMLSIVAICKKLQETG---LEAAVLNGE-SS-----------------------------------     36 Cintestinalis(XP_002123001.1)/195567/1-373                 12.4%     --SSCVEKNS--------------DFKNDC---SWFCMLVSTIRSEVFGEEENRAKDDVASLKELLQEIFN----SLNLK     37 Dmelanogaster(CG1868NP_724802.1)/226549/1-324              17.3%     --QLDQAIDEELNYHILCANLLQLYLKEHT---DFYDQFHSL--------------------------------------     38 Agambiae(XP_319721.4AGAP008973PA)/165486/1-322             14.4%     --NFDKMDPDDRMRYTLAGLMLTIYLQECT---PFAEA--------VKD-------------------------------     39 Amellifera(XP_006565387.1SMYD4likePredicted)/278571/1-294  18.9%     --NFDKLSIDDLTIYGITAIMLTIYLFKYT---NFFKTNNLE--DSLMK----K--------------------------     40 Bfloridae(XP_002589088.1BRAFLDRAFT_75068)/251714/1-464     12.8%     --HTEHHSVEQLLTQMMVSCLMCKCLGVDM------CV-------EVVK----KL-G--LEGGNCTG-------------     41 Nvectensis(XP_001627273.1)/170547/1-378                    14.7%     --NSDLQPIKALMSFAMNSAFLVEFLENGT---SSACIHCSQIKSDKTK-VQTELDS--DDDSDCSE-VY----------     42 Skowalevskii(XP_002733823.1)/75447/1-373                   13.9%     --HSGKQPWKDVFFFTLTSILLSTLVTKLI---SPSD--------DVDD-LLADTEA--MKMTEAAE------AENKSNR     43 Dpulex(DAPPUDRAFT_312722Pedicted)/241525/1-285             15.8%     --HTERMAPEELYQYALTAAFLTLLLEQHS---SFFQSAS----------------------------------------     44 Hmagnipapillata(XP_002160254.2/232532/1-301                18.7%     --HSDRLPIEDLFQYSVAGFLLYKLLI-NS---SFFKTHT----------------------------------------     45 Lgigantea(LOTGIDRAFT_169490)/248638/1-391                  14.4%     --HDNDILTEDMYQYSGTAALLLIILV-HS---GWFNTNVTQIATHIDSTLQADLQS--VEIDDKGGTIYANEGKDADIA        consensus/100%                                                       ................................................................................        consensus/90%                                                          p.t......hh...h.s.hh..hl.... ..................    .. .  ........                     consensus/80%                                                          ppppps.tphh.hhh.s.hlhhhl.tt. ........... ..                                           consensus/70%                                                          ppcp+s.pphhhhshhuhhlhphL.tt.   s.ht..... ..                                                                                                             401          .         .         .         .         :         .         .         . 480  1 Amellifera(XP_003250668.1SMYD4likePredicted)/183473/1-291 100.0%     --------------------------------------------------------QGSNDILNLTKLLCRLC-------      2 Dmelanogaster(CG8378NP_610730.1)/196491/1-296              18.8%     ----------------------------------------------------------EEGVNFFTDLLFRHLQTSPSNM      3 Agambiae(XP_566179.1AGAP000216PA)/158458/1-301             16.4%     ----------------------------------------------------------SVPYPWVGEMCYRFLKVMQCNA      4 Agambiae(XP_564258.1AGAP011234PA)/216546/1-331             17.2%     ----------------------------------------------------------PSRRKLLFELLLRHTQTALTNK      5 Agambiae(XP_309407.4AGAP011238PA)/219497/1-279             17.8%     ----------------------------------------------------------PIRSKLLFDLLLRHVQTSLINK      6 Agambiae(XP_314169.4AGAP005253PB)/218514/1-297             17.3%     ----------------------------------------------------------PTRSKLLFDLLLRHWQTSLINK      7 Agambiae(XP_309409.4AGAP011237PA)/206481/1-276             16.6%     ----------------------------------------------------------PARSKLLFDLLLRHVQTSPINK      8 Agambiae(XP_307865.2AGAP009448PA)/166466/1-301             17.6%     ----------------------------------------------------------PSIGKLLFDLILRHVQVMRINR      9 Agambiae(XP_309762.4AGAP010931PA)/113383/1-271             16.7%     ----------------------------------------------------------PTASKLLLDLILRYLQITECNY     10 Agambiae(XP_309378.2AGAP011267PA)/149447/1-299             18.0%     ----------------------------------------------------------PATNKFLLDLILRYMQIVEFNR     11 Agambiae(XP_309383.4AGAP011257PA)/149447/1-299             17.6%     ----------------------------------------------------------PATNKFLLDLILRYMQIVNCNR     12 Agambiae(XP_307655.3AGAP012638PA)/149447/1-299             17.3%     ----------------------------------------------------------PATNKILLDLILRYEQIVECNS     13 Agambiae(XP_320681.4AGAP011835PA)/183484/1-302             14.5%     ----------------------------------------------------------DNVRELLNELILRHLQTGPVNM     14 Agambiae(XP_309411.4AGAP011232PA)/162434/1-273             16.4%     ----------------------------------------------------------PILRKTLLNLLLHHLQSTIVNH     15 Amellifera(XP_001121272.2SMYD4likePredicted)/230549/1-320  15.9%     ------------------------------------------------------VVYLKESFISLAVAILHHLQAINCNA     16 Dpulex(DAPPUDRAFT_68494Predicted)/254551/1-298             19.0%     -------------------------------------------------------LKFSDQERWIGSLLLRHLQLLQFNA     17 Dpulex(DAPPUDRAFT_309882)/300599/1-300                     18.2%     -----------------------------------------------------DSLNITEDEIYFASLLLRHLQLLQFNA     18 Amellifera(XP_003249162.1SMYD4likePredicted)/239589/1-351  14.0%     -------------------------------------------------------ETPNDREIAVASLLLKHLQLLQFNA     19 Dmelanogaster(CG14122NP_648574.1)/265541/1-277             17.9%     -------------------------------------------------------VNPTAVELQVATALLGLLQVLQYNA     20 Agambiae(XP_311885.3AGAP002999PA)/268544/1-277             15.7%     -------------------------------------------------------PEPTEQELEVGAVLLSALQSLQFNA     21 Amellifera(XP_392262.3SMYD4likePredicted)/252555/1-304     19.7%     -----------------------------------------------------IEAKLSDGELYIGGLILHNLMTIQFNA     22 Dmelanogaster(CG7759NP_725048.1)/250537/1-288              17.8%     ----------------------------------------------------------PDEVSIICSLVLRSLQFIQFNT     23 Agambiae(XP_319583.4AGAP008839PA)/240523/1-284             16.4%     ----------------------------------------------------------PQEQNFIGGLLVHNLQLLQFNA     24 Amellifera(XP_001120776.2SMYD4likePredicted)/251554/1-304  22.0%     -----------------------------------------------------KDINNYSDIIFCSSIMFRACVIMSSN-     25 Amellifera(XP_001122116.2SMYD4likePredicted)/234534/1-301  21.0%     -----------------------------------------------------VLIKNDNVI-FVGGLILRHQQLIPSNI     26 Dpulex(DAPPUDRAFT_305694Predicted)/258553/1-296            16.8%     --------------------------------------------------------EDDPDE-ILATAVLQLIQSYPCNA     27 Dpulex(EFX87901.1)/258554/1-297                            17.4%     --------------------------------------------------------KDRPDE-VLATAVLRLLHSYPCNA     28 Hmagnipapillata(XP_002159692.1)/239485/1-247               17.0%     ------------------------------------------------------------LK-IVCAHLLKHIQMLPCNA     29 Skowalevskii(XP_002740933.1)/253549/1-297                  20.3%     -----------------------------------------------------ADNEEDVAI-FIGGHMLTHLQTIPCNA     30 Nvectensis(XP_001623892.1)/215512/1-298                    19.1%     -----------------------------------------------------KLPSREDQA-FIGGLLLRHLQSLPCNA     31 Lgigantea(LOTGIDRAFT_143433)/100395/1-296                  14.2%     -----------------------------------------------------TKAHLPEKC-VIGGHILRHIQMLPCNA     32 Drerio(Q08C84Smyd4)/197556/1-360                           14.6%     ----------------------------------------WMSFKDEGV-----KASWQPEMSMLGATALRHMMQLRCNA     33 Xtropicalis(NP_001072288.1SMYD4)/212545/1-334              18.9%     -------------------------------------QTEKSLTKESGT-----IEDWSSVRQFLGPTVLRHMLQLYCNA     34 Hsapiens(Q8IYR2SMYD4)/244602/1-359                         18.9%     -----------------------------------------SSQLKAAV-----TPELCPDVTIWGVAMLRHMLQLQCNA     35 Ggallus(NP_001025886.1SMYD4)/241573/1-332                  16.3%     -------------------------------------TTGSEQKTCGKT-----SDELSPELMIMAEAMLRHVLQLQCNA     36 Cintestinalis(XP_002123001.1)/195567/1-373                 12.4%     YLCENPNFDMDFFRNRI----------------KTISEMKSLSKM-SNVMTKIEPGQLTSMGEVIEYLLHRHYLQVPING     37 Dmelanogaster(CG1868NP_724802.1)/226549/1-324              17.3%     ------------------------------------------------------PASIEDWQLIISALILRFAGQLLANG     38 Agambiae(XP_319721.4AGAP008973PA)/165486/1-322             14.4%     ----------------Y-------------------------------------TMSPTELLVCCGAFITRHIGQLVCNG     39 Amellifera(XP_006565387.1SMYD4likePredicted)/278571/1-294  18.9%     -------FLNNFFNLNFNIL------------------------------------TNNDKQLYISSLLLRYILQLISNG     40 Bfloridae(XP_002589088.1BRAFLDRAFT_75068)/251714/1-464     12.8%     --------------------------ATEGGGCGENKEGGDCAKSEEG----------VVCVEKMAALLCHHMQQLRCNA     41 Nvectensis(XP_001627273.1)/170547/1-378                    14.7%     NACEEQRTQNGNFEQDRTICSRNTP--YSRQ-----------AYTSLGITTEEFCGKDGLSSDVVGALLVHHLQQMPCNV     42 Skowalevskii(XP_002733823.1)/75447/1-373                   13.9%     SPSEDESQINGSAPSRHTLDA----TDADRDRNTSQDNTTVCSNDSNKFLAAEQKLSLGDAEKAVASVLLHHLLQLRCNV     43 Dpulex(DAPPUDRAFT_312722Pedicted)/241525/1-285             15.8%     ----------------------------------------------------------LESQYLVGGLILVHVCQMVSNA     44 Hmagnipapillata(XP_002160254.2/232532/1-301                18.7%     --------------------------------------------------------VLQQHHFGVGSLLIRHIQQLICNA     45 Lgigantea(LOTGIDRAFT_169490)/248638/1-391                  14.4%     NDSKDNSLTNQ-KKQNNSISTNEKSMESNIDGLLTNGKTEACQSSSNKTSF---CGVLTNEMLDIGGLLLRHIEQLVCNA        consensus/100%                                                       ................................................................h..hhh..........        consensus/90%                                                               .... .....                    ............    ...........h.thhhphh.....Nh        consensus/80%                                                                                                             ..........hlhshll+ah..h.hNt        consensus/70%                                                                                                              ....sst..hlssllL+ahp.h.hNt                                                                          481          .         5         .         .         .         .         :         . 560  1 Amellifera(XP_003250668.1SMYD4likePredicted)/183473/1-291 100.0%     -YIYDIHARMD--------------------------FVPIFERYIALLQNLYFLLNLVRHSCSGNTIYTVHK-------      2 Dmelanogaster(CG8378NP_610730.1)/196491/1-296              18.8%     HGIDLVEQ------------------------------VNETKDDQTHSSGAYAFLSLINHSCAPNTVRIYE--------      3 Agambiae(XP_566179.1AGAP000216PA)/158458/1-301             16.4%     RPAQLTR----------------------------RDEPEGQYRAVPFALRCHPLISLLNHSCAPNVKCFDLR-------      4 Agambiae(XP_564258.1AGAP011234PA)/216546/1-331             17.2%     NDVYHMERVKLEDDHVEDDESEEDSSVDNNNSDDRKSPRNVHHEERTHAIAIYPLFSMVNHSCIPNVAPIHLL-------      5 Agambiae(XP_309407.4AGAP011238PA)/219497/1-279             17.8%     KRLNDYFDY------------------------DSDEGKPTHFEERPHAMAVYPLSSMLNHSCVPNVAPINLL-------      6 Agambiae(XP_314169.4AGAP005253PB)/218514/1-297             17.3%     KQV--------------------------------D--DEEEYSDEMHAIAVYPLFSMVNHSCIPNVAPIHLL-------      7 Agambiae(XP_309409.4AGAP011237PA)/206481/1-276             16.6%     KQFNNF-GY------------------------DSD--DEDIFEERTHAIAVYPLFSMANHSCIPNVAPIHLL-------      8 Agambiae(XP_307865.2AGAP009448PA)/166466/1-301             17.6%     QFLSFY-E---------------------------HRPDRQRFQAKEYGTACYPLVSMFNHSCASNVRRLILR-------      9 Agambiae(XP_309762.4AGAP010931PA)/113383/1-271             16.7%     KLLTCI-K---------------------------IT--NRNPEDETFTTSCYPLISMLNHSCAPNVRRLILP-------     10 Agambiae(XP_309378.2AGAP011267PA)/149447/1-299             18.0%     KLLSSN-A---------------------------YK--VKKYVAESLATSCYPLISMLNHSCAPNVQRITLR-------     11 Agambiae(XP_309383.4AGAP011257PA)/149447/1-299             17.6%     KLLSFN-A---------------------------YK--VNEYVAESFAVGCYPLISMLNHSCAPNVKRITLP-------     12 Agambiae(XP_307655.3AGAP012638PA)/149447/1-299             17.3%     KLLSFN-A---------------------------YK--VKEYVAESFAVGCYPLISMLNHSCAPNVQRITLP-------     13 Agambiae(XP_320681.4AGAP011835PA)/183484/1-302             14.5%     HSLHYM-E---------------------------YQPEQRVYEMENHVSACFPILSMLNHSCAPNVTRITLR-------     14 Agambiae(XP_309411.4AGAP011232PA)/162434/1-273             16.4%     QFLHYM-D---------------------------YLAEQDVYEPDEYAIACFPLLSMLNHSCAPNVKRITMR-------     15 Amellifera(XP_001121272.2SMYD4likePredicted)/230549/1-320  15.9%     YEIVENIYDKK----------------------------THIWEPRQIGGAIYPSVSLINHSCYPNVVRHTYP-------     16 Dpulex(DAPPUDRAFT_68494Predicted)/254551/1-298             19.0%     HEVSELRMD-R-------------------------PGCMEGAKTFFLGAGVYSTVALLNHSCEPGVIRHFIG-------     17 Dpulex(DAPPUDRAFT_309882)/300599/1-300                     18.2%     HEIHEFVQL-N-------------------------EKNMRSTKTVYIGVGIYPTVAFFNHSCRPDVARYFLG-------     18 Amellifera(XP_003249162.1SMYD4likePredicted)/239589/1-351  14.0%     HEVFETRLG-M-------------------------EHRFRGSKPIYIGVAIYPTVARFNHDCYPAVTRYFLG-------     19 Dmelanogaster(CG14122NP_648574.1)/265541/1-277             17.9%     HQIYQTQVT-E-------------------------EHRFDGSKTVYLAAGLYGTGSYFNHECWPSTACHFVG-------     20 Agambiae(XP_311885.3AGAP002999PA)/268544/1-277             15.7%     HEVYETRIT-G-------------------------EHRFDTAKVQYIGVGIYRGASMFNHECYPGVTRTFLG-------     21 Amellifera(XP_392262.3SMYD4likePredicted)/252555/1-304     19.7%     HEISELVIPKA-------------------------DNNLANAKSKFIGGGLYPTISLFNHSCNPGIIRYFIG-------     22 Dmelanogaster(CG7759NP_725048.1)/250537/1-288              17.8%     HEVAELHKFSS-------------------------S---GREKSIFIGGAIYPTLALFNHSCDPGVVRYFRG-------     23 Agambiae(XP_319583.4AGAP008839PA)/240523/1-284             16.4%     HEVSEMIRETA-------------------------E---DIGKSTFIGGGLYPTLALFNHSCDPGVTRYYRG-------     24 Amellifera(XP_001120776.2SMYD4likePredicted)/251554/1-304  22.0%     ------------------------------------CFSVQQEPGIKIGSGLYVTNSLYNHSCAPNTFRHFEG-------     25 Amellifera(XP_001122116.2SMYD4likePredicted)/234534/1-301  21.0%     HSFSEEC----------------------------------GLDAVERGIAAMPFFSLINHSCNPNILRHSRS-------     26 Dpulex(DAPPUDRAFT_305694Predicted)/258553/1-296            16.8%     HEISHLAFPLP-GT----------------PSGPDLPSTLQQIRLCEIGAAAMPVLSLINHSCDPNVVRDCYG-------     27 Dpulex(EFX87901.1)/258554/1-297                            17.4%     HEISHMAIPVP-SG----------------FCAQSKSLQLQQIQSCEIGSAAFPVVSLMNHSCNPNVVHLCYG-------     28 Hmagnipapillata(XP_002159692.1)/239485/1-247               17.0%     HEVSELQLKAS---------------------------NYKDSELKEIGSAVYATLSLLNHSCDPSVVRHCYG-------     29 Skowalevskii(XP_002740933.1)/253549/1-297                  20.3%     HEISEYELWRS---------------------------DITKCHFVEVGSGLYPTMSLVNHSCDPVVTRNCYG-------     30 Nvectensis(XP_001623892.1)/215512/1-298                    19.1%     HEISELQLSLK---------------------------SVATSEAAEIGAGIYGTLSLFNHSCEPNVTRFFYG-------     31 Lgigantea(LOTGIDRAFT_143433)/100395/1-296                  14.2%     HEVSEFAYREY---------------------------DLPNSQTMEIGSGIYATLSLINHSCDPNVVRHSYG-------     32 Drerio(Q08C84Smyd4)/197556/1-360                           14.6%     QAITAVRVKEESG------------------M------AVQSSSEIRIATAIFPVLSLLNHSCSPNTSISFTTGFQPDPH     33 Xtropicalis(NP_001072288.1SMYD4)/212545/1-334              18.9%     QAVTALQENEDES----------------SLS------LVKSNKSIRLATAVFPVLSLLNHSCDPNTTVSFTG-------     34 Hsapiens(Q8IYR2SMYD4)/244602/1-359                         18.9%     QAMTTIQHTGPKG------------------S------IVTDSRQVRLATGIFPVISLLNHSCSPNTSVSFIS-------     35 Ggallus(NP_001025886.1SMYD4)/241573/1-332                  16.3%     QAITVMQELESGD------------------G------AVVNKKPVRLATAFFPVLSLLNHSCSPNISVSFSG-------     36 Cintestinalis(XP_002123001.1)/195567/1-373                 12.4%     QSISFVTEELCD--------------------------NVTVTRRDIVASAFFPTMSMMNHSCDCNTDALFNG-------     37 Dmelanogaster(CG1868NP_724802.1)/226549/1-324              17.3%     HVGDALLGVGMEPKEFVMLQP----ELWQKPRHLKRGQLHNLSHSDPITAINLPYLSLCNHACEPSIRTKFDG-------     38 Agambiae(XP_319721.4AGAP008973PA)/165486/1-322             14.4%     HAISELRLALPSKGQF--YN--------LNDSLLLAGTLHLCLKSSRVFTAIFPRISMFNHSCDPNIRNHFER-------     39 Amellifera(XP_006565387.1SMYD4likePredicted)/278571/1-294  18.9%     HAITKSNIFLSE-----------------NDS------S--MIQQDIVATGIYPSASIMNHSCDPNIINIFVN-------     40 Bfloridae(XP_002589088.1BRAFLDRAFT_75068)/251714/1-464     12.8%     QAITTLQEQDSV--------------------------SLLEDKQVRLATAVFPTEALLNHSCRPNVFVSFQG-------     41 Nvectensis(XP_001627273.1)/170547/1-378                    14.7%     HAITAIVSTSSSDEEDEEMG--------SSHD------QVVAREQRRIASAIYPTASLLNHACDPDVLVSFVD-------     42 Skowalevskii(XP_002733823.1)/75447/1-373                   13.9%     HAVTEVATKTDS-----------------STS------FVATTQQIRIAVAVYGTASMLNHSCTPNVIAGYDG-------     43 Dpulex(DAPPUDRAFT_312722Pedicted)/241525/1-285             15.8%     HAITELCLIDEN-----------------------------NERQERIATAIYPSASLMNHNCDPTVINSFQG-------     44 Hmagnipapillata(XP_002160254.2/232532/1-301                18.7%     HAVTCLSA-EKL-----------------DTT------SVIDQEQVRIATAIYPTTSLLNHSCEPTILNCFHK-------     45 Lgigantea(LOTGIDRAFT_169490)/248638/1-391                  14.4%     HAITEVQCTDTI-----------------NDS------MILDTSQVRIATAIYPTASLMNHSCDPTIISSFHG-------        consensus/100%                                                       ...............................................h....h...shhpHtC.ssh.............        consensus/90%                                                        p.h...........               ..............p...hs.uhashhuhhNHuC.Psh...h..               consensus/80%                                                        p.lp.h.......                 ..   ......t.p...husuhashhShhNHSCtPslhh.hht               consensus/70%                                                        +tlsth......                   .   .....tt.ct..lusuhaPhhShhNHSCsPslhphhhs                                                                                 561          .         .         .         6         .         .         . ] 632  1 Amellifera(XP_003250668.1SMYD4likePredicted)/183473/1-291 100.0%     -------------------NNVLVLRAAKDIYPGELITFNFMSKYVALESNSMPRNVMLKNFFDISCDCEAC      2 Dmelanogaster(CG8378NP_610730.1)/196491/1-296              18.8%     -------------------GTKAYMFVLRPIKAGNVLYDNYGAHFAICSK--EQRLKRLSLQYRFDCKCEGC      3 Agambiae(XP_566179.1AGAP000216PA)/158458/1-301             16.4%     -------------------DGRCSAVVIQPIAAGGQLFANYGYDYLQTGR--DERREGLQRVFGFTCNCDAC      4 Agambiae(XP_564258.1AGAP011234PA)/216546/1-331             17.2%     -------------------DGRLAMVATRPIAAGEQLYNINGFSTFDPDD--SARRHALQLSHFFKCRCASC      5 Agambiae(XP_309407.4AGAP011238PA)/219497/1-279             17.8%     -------------------DGRCAIVAIRPIAAGEQLFDNYG------------------------------      6 Agambiae(XP_314169.4AGAP005253PB)/218514/1-297             17.3%     -------------------DGRCAFVATRPIAAGEQLFDVYAFASMDFDR--SFRIFCLRKSYYFKCRCAVC      7 Agambiae(XP_309409.4AGAP011237PA)/206481/1-276             16.6%     -------------------DGRCAFVVSRPIAAGEQLFDVYG------------------------------      8 Agambiae(XP_307865.2AGAP009448PA)/166466/1-301             17.6%     -------------------DGRCAMIVIRPIGPGEQLFDSYGLHHFSFER--SHRQKGTFVMFNFECCCEAC      9 Agambiae(XP_309762.4AGAP010931PA)/113383/1-271             16.7%     -------------------DGRCAVIVIHTVAKGGQLFDNYE------------------------------     10 Agambiae(XP_309378.2AGAP011267PA)/149447/1-299             18.0%     -------------------DGRCAVFVIRPVLEGSQLFDSYETDHKSHER--AMRQLMLSFTYSFRCTCEAC     11 Agambiae(XP_309383.4AGAP011257PA)/149447/1-299             17.6%     -------------------DGRCAVFVIRPVLEGSQLFDSYEAGHTLHER--EMRQSMLSFTYSFRCTCEAC     12 Agambiae(XP_307655.3AGAP012638PA)/149447/1-299             17.3%     -------------------DGRCAVFVIRPVLEGSQLFDSYEADHILNKR--AMRQSMLSFMYSFRCTCEAC     13 Agambiae(XP_320681.4AGAP011835PA)/183484/1-302             14.5%     -------------------DGRCAVLVTRPIAKGGQLYDNYGMHHCLMSR--KERKTELLKQYRFICECEAC     14 Agambiae(XP_309411.4AGAP011232PA)/162434/1-273             16.4%     -------------------DGRCALVVTRQIADGGQLFDHYE------------------------------     15 Amellifera(XP_001121272.2SMYD4likePredicted)/230549/1-320  15.9%     -------------------SGIVVVRTLRFVGKGTEILDCYGPHWFSENK--LSRIEYLWKKYRFLCTCDAC     16 Dpulex(DAPPUDRAFT_68494Predicted)/254551/1-298             19.0%     -------------------D-VMVVRAIKSFQPGEMVNENYGPIFTQKRR--VDRQRSLKDRYWFDCRCNPC     17 Dpulex(DAPPUDRAFT_309882)/300599/1-300                     18.2%     -------------------T-TMVITSTRCVKRGQMVAENYGPIFTHKHL--TDRQQSLQGRYWFNCQCLAC     18 Amellifera(XP_003249162.1SMYD4likePredicted)/239589/1-351  14.0%     -------------------R-CIVIRAIRSLRPGDVVAENYGPIFTKRNL--EERRRNLAGRYWFFCECNAC     19 Dmelanogaster(CG14122NP_648574.1)/265541/1-277             17.9%     -------------------K-KLVLTATRPHRANELVAVNYGPIFIKNNL--KERQRSLRGRYSFSCSCMAC     20 Agambiae(XP_311885.3AGAP002999PA)/268544/1-277             15.7%     -------------------T-AMILHTSRPIPAGAVVPENYGPHFMRQPK--AIRQRNLRSRYWFKCDCRAC     21 Amellifera(XP_392262.3SMYD4likePredicted)/252555/1-304     19.7%     -------------------T-TMVVRAIRSISSGEEISENYGQIFTTTPE--SERKRKLRLQYFFDCNCEAC     22 Dmelanogaster(CG7759NP_725048.1)/250537/1-288              17.8%     -------------------T-TIHINSVRPIEAGLPINENYGPMYTQDER--SERQARLKDLYWFECSCDAC     23 Agambiae(XP_319583.4AGAP008839PA)/240523/1-284             16.4%     -------------------N-QVCVRTVKNIPADSMVAENYGPLFTQVRR--DERRDTLLHQYRFTCQCVPC     24 Amellifera(XP_001120776.2SMYD4likePredicted)/251554/1-304  22.0%     -------------------L-TMITRALKPLYPGDQIFTSYGAAYAYMTR--SERREKIMQDYFFECDCIAC     25 Amellifera(XP_001122116.2SMYD4likePredicted)/234534/1-301  21.0%     -------------------N-YMIIYVIYPIKKGEQLYDNYGQHYAITPK--EERQKELLKQYYFKCNCLAC     26 Dpulex(DAPPUDRAFT_305694Predicted)/258553/1-296            16.8%     -------------------D-VIAVKAIRRIARGDEILDNYGYHYATHDK--KERQLKLSQQYYFRCNCLAC     27 Dpulex(EFX87901.1)/258554/1-297                            17.4%     -------------------D-VMVVKVIHRIARGEEILDNYGYHYATHEK--RERQLKLCQQYYFRCRCQSC     28 Hmagnipapillata(XP_002159692.1)/239485/1-247               17.0%     -------------------D-TCVLRAIKHIKEGSEIVDNYGFLYAVESK--VIRQSHLMEQYYFACQCEAC     29 Skowalevskii(XP_002740933.1)/253549/1-297                  20.3%     -------------------E-TCVVRAIRNIYKGEEITDNYGYLYPVHDK--SERQTRLKWQYFFECKCDAC     30 Nvectensis(XP_001623892.1)/215512/1-298                    19.1%     -------------------D-KCVVRAFSSIPCRGEVVDNYGILSALTPR--KQRQESLQSQYYFKCNCHAC     31 Lgigantea(LOTGIDRAFT_143433)/100395/1-296                  14.2%     -------------------D-FCAVRAIRNIPKGTEVYDSYGALYPLTAK--KDRQEKLLSQYFFKCSCKAC     32 Drerio(Q08C84Smyd4)/197556/1-360                           14.6%     NQLGCSEGHFDHPKGSRSGV-TVTVRASKDLTAGQEILHCYGPHRSRMEV--KERQRLLLEQYFFQCVCQAC     33 Xtropicalis(NP_001072288.1SMYD4)/212545/1-334              18.9%     -------------------R-FVTVRANRPIRRDEEVTHCYGPHKLRMDV--AERQQLLKDQYFFVCQCKAC     34 Hsapiens(Q8IYR2SMYD4)/244602/1-359                         18.9%     -------------------T-VATIRASQRIRKGQEILHCYGPHKSRMGV--AERQQKLRSQYFFDCACPAC     35 Ggallus(NP_001025886.1SMYD4)/241573/1-332                  16.3%     -------------------T-AATVRASQPIPSGQEIFHCYGEEM---------------------------     36 Cintestinalis(XP_002123001.1)/195567/1-373                 12.4%     -------------------S-TVTFRSNQFIPVGAEITHCYGPSVFHASF--EERQKTLKENYSFDCDCTPC     37 Dmelanogaster(CG1868NP_724802.1)/226549/1-324              17.3%     -------------------C-SVVNYAAKDILEGEEIFNCYTMDYRNSLK--LQRSHPLKAIYKFECTCAKC     38 Agambiae(XP_319721.4AGAP008973PA)/165486/1-322             14.4%     -------------------A-TLTVHATRPIGAGGEVFNCYGPHYRLMAA--AERKMLLRAQYCFECGCERC     39 Amellifera(XP_006565387.1SMYD4likePredicted)/278571/1-294  18.9%     -------------------Q-YLIVRASRDISQGEEIFNCYGPHYRHMTT--ENRQKILKNQYCFICKCKAC     40 Bfloridae(XP_002589088.1BRAFLDRAFT_75068)/251714/1-464     12.8%     -------------------K-TLIVRAVSHIKPGEELLHCYGPHAGRMVY--GERQAALKEQYFFSCSCDAC     41 Nvectensis(XP_001627273.1)/170547/1-378                    14.7%     -------------------G-VLVARATHNIAPGSGITHCYGPHVNHMPR--EERQKLLYKQYFFTCQCSAC     42 Skowalevskii(XP_002733823.1)/75447/1-373                   13.9%     -------------------N-QLTIRATEMIKKGGEVLHCYGPRVSDMFR--DERLKVLRDQYYFTCKCMFC     43 Dpulex(DAPPUDRAFT_312722Pedicted)/241525/1-285             15.8%     -------------------N-TLIVRAIRNVRQGDEVFNCYGPHYRRMRR--SERVEALEAQYSFTCTCDSC     44 Hmagnipapillata(XP_002160254.2/232532/1-301                18.7%     -------------------N-QLIVKVVKDVVKGEQIFNCYGPHFKRMGY--EDRRAALMQQYFFLCSCEHC     45 Lgigantea(LOTGIDRAFT_169490)/248638/1-391                  14.4%     -------------------D-TLIVKSVKKVLEGEEIYNCYGPHHKRMVR--KRRQEVLENQYFFHCKCPPC        consensus/100%                                                       ......................h...s...h..t..l...................................        consensus/90%                                                                           t.hhhhhs.p.l..Gt.lhpsYt........  ....................        consensus/80%                                                                           s.hhhhhsh+.l.tGttlhpsYG..h....h  ..R...L...Y.F.CpC.sC        consensus/70%                                                                           s.phslhsh+slhtGpplhcsYG.hhh.h.h  tpRpt.L.tpYhFpCpC.sC ``` |
