# Supplementary material for: Evolutionary History of the Smyd Gene Family in Metazoans: A Framework to Identify the Orthologs of Human Smyd Genes in Drosophila and Other Animal Species
Source: PLoS One. 2015 Jul 31;10(7):e0134106. doi: 10.1371/journal.pone.0134106 (PMC4521844; doi:10.1371/journal.pone.0134106)
Supplement: S3 File — Formatted in mview. (HTM) [file pone.0134106.s010.htm]

```
Reference sequence (1): cintestinalis(NP_001071820.1)/15-282
Identities normalised by aligned length.
Colored by: identity + property
```

|  |
| --- |
| ```                                                                      1 [        .         .         .         .         :         .         .         . 80    1 cintestinalis(NP_001071820.1)/15-282                    100.0%     GR----GLKATRKFETGQAVLKQ-EPYAYAVMSS-H-----I--------------------DVVCHYCLCAPGQPG---       2 Drerio(Q6P0R5-Smyd1a)/18-279                             35.9%     GR----GLRGTRDLSAGEVVFAE-ASFAAVVLDS-L-----S--------------------LQVCHSCFRR--------       3 Derio(Q2MJQ9-Smyd1b)/13-274                              34.5%     GR----GLRATKEAWAGDVLFAE-PPFASVVFDS-Q-----A--------------------SSICHSCFRR--------       4 Xtropicalis(NP_001120357.1-SMYD1)/13-261                 38.9%     GR----GLRAIRESWAGDIIFAE-PAYSAVVFDN-L-----S--------------------HSVCHSCFKR--------       5 Hsapiens(Q8NB12-SMYD1)/18-279                            38.4%     GR----GLKATKEFWAADIIFAE-RAYSAVVFDS-L-----V--------------------NFVCHTCFKR--------       6 Ggallus(NP_989486.1-SMYD1)/13-274                        39.1%     GR----GLKAQKEFLPGDVIFAE-PAYAAVVFDS-L-----T--------------------HVICHTCFKR--------       7 Athaliana(Q7XJS0-ASHR1)/22-274                           25.8%     GR----SLFTARDFRPGEVILSQ-KPYICVPNNT-S-----S--------------------ESRCDGCFKT--------       8 Amellifera(XP_625013.1-SMYD3-Predicted)/1-253            27.1%     ------MSESENFIKKGTTLFTA-KPFAYVLYSK-Y-----R--------------------NERCDYCFKS--------       9 Dmelanogaster(Buzidau-CG13761)/26-282                    25.6%     KN----LKNPAPQIKRGQRILTE-KPFAFVLKSQ-Y-----R--------------------LERCDNCLEA--------      10 Agambiae(XP_319707.4-AGAP008954-PA)/1-254                25.0%     --------MRKTIHRRGDVILQE-KPFACVLDPR-Y-----R--------------------DSRCDRCFKE--------      11 cintestinalis(XP_002128556.1)/14-266                     32.4%     GR----GLKAKRNLNPGSTVLSS-EPYAYLLSKK-Q-----K--------------------GVYCDFCFKK--------      12 Lgigantea(LOTGIDRAFT_177746)/1-216                       26.2%     ------------MSKKGELIAKA-EPYVHVLAYK-E-----I--------------------DKLCSFCFLP--------      13 Drerio(E7EZZ6-SMYD3)/16-267                              34.7%     GN----GLRALREIKPGEVIYSC-KPFAFCVARD-F-----L--------------------KTACQSCLKR--------      14 Xtropicalis(XP_004914684.1|-SMYD3-Predicted)/15-264      31.0%     GN----GVRALKDMSHGLTVMIA-EPYVYTVCRI----------------------------KTACDHCLHR--------      15 Hsapiens(Q9H7B4-SMYD3)/15-266                            33.9%     GN----GLRAVTPLRPGELLFRS-DPLAYTVCKG-S-----R--------------------GVVCDRCLLG--------      16 Ggallus(XP_419536.1-SMYD3-Predicted)/15-266              33.2%     GS----GLRSRRQVRPGELLYRA-EPFAYVVTKE-Q-----L--------------------GGVCEQCLQR--------      17 Drerio(Q5RGL7-Smyd2b)/19-268                             30.9%     GR----GLRVSRAYGVGELLFSC-PAYSYVLSVG-E-----R--------------------GLICEQCFTR--------      18 Drerio(Q5BJI7-Smyd2a)/18-267                             32.0%     GR----GLKAIKHFKVGDLVFAC-PAYAYVLTVN-E-----R--------------------GGRCECCFTR--------      19 Xtropicalis(XP_002934751.2-SMYD2-like-Predicted)/16-265  30.9%     GR----GLKATRPFALGELLFSC-PAYTYVLTVN-E-----R--------------------GNHCEFCFAR--------      20 Hsapiens(Q9NRG4-SMYD2)/18-267                            32.0%     GR----GLRALQPFQVGDLLFSC-PAYAYVLTVN-E-----R--------------------GNHCEYCFTR--------      21 Ggallus(XP_419420.1-SMYD2-Predicted)/21-270              31.6%     GR----GLRALRRYAVGELLFSC-PAYTAVLTVS-E-----R--------------------GSHCDGCFAR--------      22 Tadhaerens(XP_002109888.1)/20-262                        28.4%     GR----GIRCKKQLAIGTSVGKE-NPFCHVVSQD-M-----L--------------------SSYCHSCLLM--------      23 Hmagnipapillata(XP_002163555.2)/16-259                   29.4%     GR----GVRALQDIKRGVEILKE-EPLACILTNSKY-----R--------------------GIRCDYCYSE--------      24 Nvectensis(XP_001627600.1)/17-253                        32.1%     GR----GLRAAKPLKSGDTILSE-QPVVYMLSNM-L-----R--------------------GQRCDFCLEK--------      25 Bfloridae(XP_002594889.1-BRAFLDRAFT_124463)/14-258       38.4%     GR----GLCATKVFKPGNLVRAA-DPYAYVLCNS-E-----R--------------------GKRCDFCFAR--------      26 Skowalevskii(XP_006817727.1)/14-260                      33.2%     GR----GYRTVTRVKVGELVLKA-QPFVHVLCNT-E-----R--------------------GNRCDFCLRS--------      27 Amellifera(XP_006565332.1)/43-285                        18.4%     GR----GMFATRDIKQNELIFID-APLIVGPKCL-------------------------NKQTKMCICCYKNE-CPL---      28 Dmelanogaster(msta-CG33548)/66-313                       17.7%     GR----GVFATRDIAAGELIFQE-RALVTGPTAR-------------------------KGQLSSCICCHETLPQTG---      29 Dmelanogaster(CG12119)/34-280                            19.0%     GR----GVVATRSLKRGEIIFRD-SPLLIGLAAH------------------------EEDSLNACSVCLKMLPDTR---      30 Amellifera(XP_006565301.1)/26-284                        23.2%     GR----YLQASKDLRAGEVILRE-DPVAVGPMS--------------------------CVKDPICFECLSILPNIE---      31 Dmelanogaster(CG9642)/21-271                             18.8%     GR----FAVALCNVRAGETLLLE-NPIVVLPLM--------------------------G--ERRCSKCFNLT-------      32 Dmelanogaster(CG9640)/17-268                             16.9%     GR----HLVASIAIEPGDTILEE-RPLLVAPHW--------------------------ECHQLKCAQCLQES-------      33 Amellifera(NP_001229486.1-LOC724300)/57-301              21.5%     GR----HLLASRDLNPGDVILSE-SPLVWGPSI--------------------------HSDQRLCVGCGKQCKSAN---      34 Dmelanogaster(CG14590-NP_610202.3)/55-322                18.9%     GR----YLKVTQNIAAGQIVFIE-EPLVVGPKWY-LSDA------------------DKEASNVPCVGCYTPCRLGK---      35 Dmelanogaster(CG43129)/21-279                            17.1%     GR----YLVAKGAIRGHGLLIEE-LPFAVGPKC---------------------------NGPVVCLGCYEPNPDP----      36 Dmelanogaster(G11160)/58-319                             20.8%     GR----YLVANRQLEAGETLIRE-EPLAIGPCV---------------------------SGDPVCLGCYHPVSLKA---      37 Amellifera(XP_624539.3-msta-like-Predicted)/54-297       21.5%     GR----HYIATRNIKVGEIILRDDQPLITGLMY---------------------------NTVPVCLQCYTVLNQE----      38 Dmelanogaster(CG8503-NP_610944.1)/52-301                 20.0%     GR----HLVATRTIKPYEIVLKE-APLVRGPAQ---------------------------ISAPVCLGCLNGIEAE----      39 Agambiae(XP_309979.4-AGAP011530-PA)/50-300               22.8%     GR----FLVATRDIKAGEIVLKE-SPLVHGPAQ---------------------------ITGPVCVGCLQGLEEK----      40 Dpulex(DAPPUDRAFT_120473)/58-292                         19.1%     GR----CIFASKNLKPGEIIFGE-TAVITGPKQ---------------------------GCTPCCLKCYASLDRVQ---      41 Dpulex(DAPPUDRAFT_194440-Predicted)/53-302               19.6%     GR----HLIACRDLKAGDVILQE-KPIVMGPKH---------------------------TAGQICLGCYSGVDGR----      42 Dpulex(DAPPUDRAFT_2393)/50-297                           20.2%     GR----YLVASRLIKAGEVILQE-LPLVVGPKL---------------------------NTLPLCLGCYKSITDT----      43 Dmelanogaster(CG18136-NP_649084.1)/58-318                22.6%     GR----HLRATRDIKIGEQILKE-APLVLGPKV---------------------------ASAPLCLGCHRNLLAPG---      44 Agambiae(XP_309220.5-AGAP001025-PA)/55-318               20.7%     GR----HLVATRHIKQGEIIYRD-EPYAVGPKI---------------------------ANVPLCLGCNRNLMAGWDAT      45 Scerevisiae(P38890.1-SET5)/124-429                       16.1%     GR----GLFAKRDFSKGQIILKENKPIVYIPPLD-KL--------------------FLISNGKACARCGKALYDLT-QH      46 Athaliana(Q9ZUM9-ASHR2)/22-296                           14.0%     GR----SLVAAQSLRAGQVILRESP-LLLYSAFP-FL--------------------S-SSVSPYCDHCFRLLASSA-H-      47 Athaliana(Q9FG08.2-ATXR4)/53-321                         16.2%     GR----AVFATRKIGAGDLIHTAKP-VVACPSLL-K-------------------------LDSVCYLCLKKLMGSA-K-      48 Lgigantea(LOTGIDRAFT_232186)/323-670                     15.0%     GR----GVYATEDIKEGDIAFVD-SPVVRAMISN-P--E--H-------------------KIEACSHCARSLLTAAQYF      49 Bfloridae(XP_002589246.1-BRAFLDRAFT_74594)/380-720       14.2%     GR----AVFCTEDVAEGQELFRD-TPLVSSQTDD-S--A--K-------------------AHPACSHCAVSLLTAEDYF      50 Athaliana(Q5PP37-ATXR2)/52-466                           14.1%     GK----GVYANSEFDEDELILKD-EILVGIQHSS-N--K--V-------------------DCLVCSFCFRFIGSIEKQI      51 Cowczarzaki(EPH53581.1)/160-496                          15.7%     GK----GVVTLKELQYGTEVFHE-APVVSHRFVG-A--EENS-------------------AIPACSHCLQTRLTPDMMG      52 Mbrevicollis(MONBRDRAFT_29283)/14-364                    16.5%     GR----RLVATRHIEAGDIVWQE-SPLACAQFLW-N--R-AC-------------------GYRACQHCLRSLESPQETV      53 Cowczarzaki(EFW42079.2)/57-422                           15.4%     GR----GLFATQAFKKGDIVFTE-APLVCAQFLW-N--E-AY-------------------GYKACHQCMRSLESPGEMA      54 Tadhaerens(XP_002114620.1)/25-373                        17.6%     GK----GLFATNCFNEGDEIFKE-NPLVCAQFLW-N--E-FY-------------------KYEACEYCLRSLEDAETMA      55 Bfloridae(XP_002609030.1-BRAFLDRAFT_84846)/1-276         11.7%     ------------------------------MFRS-P--Y-VP-------------------KSPSCDHCMRSMEPAEAMS      56 cintestinalis(XP_002127168.1)/13-358                     17.5%     GF----GLFSTEDISSDSVILEE-DPIISCQFSW-N--K-LY-------------------KYRACDYCMKSLETTEEMC      57 Dpulex(EFX89935.1)/23-367                                16.0%     GR----GLFTTRSFKNGETIIEE-QPLFSCQFSW-N--Y-AY-------------------GYSACDFCMRPLETAEENA      58 Dmelanogaster(CG3353-NP_650955.1)/13-363                 15.5%     GR----AMIATKNFAKDEVIFEE-EPFVSRQFSW-N--V-AY-------------------GYAACDHCMRPLETVLENV      59 Hmagnipapillata(XP_002163562.2)/21-371                   16.0%     GK----GLFAASAIKKGDTILTE-KPLVLCQFSW-N--R-QY-------------------NYVACDYCMRSLETAQNMA      60 Agambiae(XP_313299.1-AGAP003552-PA)/13-365               16.8%     GR----GLYAAELIPEGGTIFEE-QPLVSCQYSW-N--A-AY-------------------GYLACEYCLRPLETAERNA      61 Amellifera(XP_394075.2-SMYD5-like-Prediction)/16-364     16.1%     GK----GLFAIRSFKDGDTILEE-KPIICSQFAW-N--L-DY-------------------GYLACDNCLTPLETAEENV      62 Nvectensis(XP_001627062.1)/18-370                        15.2%     GR----ALFASRDFKEGDTIFEE-DPLVCSQFLW-N--A-AY-------------------SYTACDHCMRSLETAQDMA      63 Skowalevskii(XP_002735533.1)/24-372                      13.9%     GK----GVFAKQRFRKNDVIFRE-KPIVCAQFLW-N--E-YY-------------------KYSACDHCMKSLETAEEMA      64 Lgigantea(LOTGIDRAFT_231752)/19-367                      16.4%     GR----GLFARQEIKEGEAILDE-KPLVSTQFLW-N--E-LY-------------------KYTACEYCLRSLETAEAMA      65 Drerio(F1RET2-Smyd5)/32-380                              16.4%     GK----GLFAKKPFKKGDTIFIE-RPLVSSQFLW-N--A-LY-------------------KYRACEYCLRALETAEENA      66 Ggallus(NP_001012912.1-SMYD5)/39-387                     16.9%     GK----GLFATRSIRKGEAVFVE-KPVVSSQFLW-N--A-LY-------------------NYRACDHCLRALETAEENA      67 Hsapiens(Q6GMV2-SMYD5)/33-381                            15.8%     GK----GLFATQLIRKGETIFVE-RPLVAAQFLW-N--A-LY-------------------RYRACDHCLRALEKAEENA      68 Xtropicalis(A9ULL8-SMyd5)/32-382                         16.9%     GK----GLFATRAIRKGETIFQE-KPLVSSQFQW-N--A-LY-------------------RYRACDHCLRSLETAEENA      69 Mbrevicollis(MONBRDRAFT_36878)/153-462                   16.1%     GR----ALFTSTPLAAGDVLLRE-RAFASILAPT-T--TQEN--------------------YLRCHECLDESW------      70 Mbrevicollis(MONBRDRAFT_27776)/11-280                    20.3%     GR----AVRATKALARGQTVLLN-PPLAFVLRHE-----ERV--------------------ARRCEDCFVSEKPEH---      71 Cowczarzaki(EFW45970.2)/35-344                           21.5%     GR----HITAKRDFRAGELVLAS-KPYAAVADTD-----GPA--------------------AGRCSECFQAQDEDADVA      72 Dpulex(EFX73755.1)/45-306                                19.5%     GSLKMRNTGKHEPIPKGTTILES-VPFVYCLKS------SFR--------------------RELCDFCLKANS------      73 Bfloridae(XP_002593048.1-BRAFLDRAFT_74375)/6-196         17.6%     ---------GVAAGPDGQLVVVD-R-------------------------------------NERTVTIFPRPE------      74 Bfloridae(XP_002594298.1-BRAFLDRAFT_117670)/15-265       21.7%     GRGIRCNKKGSSGIEPGTLIVKE-EPYSYTLTDG-----ELL--------------------RTRCHYCLKRLE------      75 Scerevisiae(Q12529.1)/23-365                             10.9%     GR----ACFSNGNIPKGTTVLQV-SNFTGTSISY-E-----FR-------------------KEVCHNCFAYANA-----      76 Dmelanogaster(CG1868-NP_724802.1)/226-549                15.1%     GR----YMVAKEAISKGNVIFSE-RASCFVPLEQ----------------------------LLICQQCAATLMS-----      77 Agambiae(XP_319721.4-AGAP008973-PA)/165-486              14.6%     GR----YVVAAEAIKANDTVARE-TAVSFVPVYD-P-----ES--------------SSTLPSFDCQKCAK-VNV-----      78 Athaliana(NP_174606.2)/229-550                           12.6%     GR----GMVSECDIEEASVIHVE-EPFSVVISKS-C-----R--------------------ETHCHFCLNELPA-----      79 Cintestinalis(XP_002123001.1)/195-567                    14.8%     GR----HYFTTFNTETNECLLEE-VAYLGVLNPE-F-----F--------------------STHCSYCLTPCKS-----      80 Drerio(Q08C84-Smyd4)/197-556                             15.4%     GR----HMLVMENKPAGEVVLED-EAYCSVLIPA-N-----IFNT----G-TNKAVETFGTEDRHCHHCLSQSL------      81 Xtropicalis(NP_001072288.1-SMYD4)/212-545                18.0%     GR----HLLASQNIEQGEVLIWE-EAFASVIIPE-R-----K-----QWRKEIKWDTRITACDHYCHYCLNRVI------      82 Hsapiens(Q8IYR2-SMYD4)/244-602                           16.3%     GR----CLVATKDILPGELLVQE-DAFVSVLNPG-E-----LPP--PHHGLDSKWDTRVTNGDLYCHRCLKHTL------      83 Ggallus(NP_001025886.1-SMYD4)/241-573                    15.3%     GR----HLVASQDILPGQNLLKE-KAFVSVLCPG-E-----GDSLLLQDSSETVWDTRVTNADLYCHHCLKQLL------      84 Hmagnipapillata(XP_002160254.2/232-532                   15.5%     GR----YIFAKEDIPNGSIIISE-KPYAAVLLPH-W-----Y--------------------KTHCQLCFDKVV------      85 Dpulex(DAPPUDRAFT_312722-Pedicted)/241-525               17.9%     GR----YVVANRDIKAGETLFVE-QPNALVVLPD-F-----Q--------------------TSRCHHCTRHSSA-----      86 Amellifera(XP_006565387.1-SMYD4-like-Predicted)/278-571  17.3%     GR----HVIANKFIKEGDILFLE-EPISFVLLNH-D-----T--------------------YSYCQYCNNLNTD-----      87 Bfloridae(XP_002589088.1-BRAFLDRAFT_75068)/251-714       13.4%     GR----MLVAQKAFEPGSVLIVE-QPYAAVLLQK-H-----H--------------------STHCHTCVTPVL------      88 Lgigantea(LOTGIDRAFT_169490)/248-638                     12.9%     GR----YLTTNREIEVGDTLIVE-KPFSSVLLPD-H-----Y--------------------KTHCHHCYHKLPL-----      89 Skowalevskii(XP_002733823.1)/75-447                      16.7%     GR----YILATETICRGEIIIKE-KPYGCVLLPS-H-----Y--------------------NTRCYHCVRKTV------      90 Nvectensis(XP_001627273.1)/170-547                       14.3%     GR----FLQASSEIRAGDTLIAE-EPYSAVLLPE-N-----A--------------------KTHCECCYKSLV------      91 Amellifera(XP_003250668.1-SMYD4-like-Predicted)/183-473  15.8%     GR----YFVAVKPIKMKDVILID-KSQITHLHKD-DWDDDPT--------------------SNMCHYCFK-YCR-----      92 Cowczarzaki(XP_004349923.1)/103-371                      17.2%     GY----GLVATRPIRRGEVVVRE-CIFLESQ-PP-L-----E--------------------GNDSDPCA----------      93 Amellifera(XP_001121272.2-SMYD4-like-Predicted)/230-549  19.0%     GR----HLIATKNIKAGSVLIVE-TPFAFSTNKE-A-----L--------------------GRNCLHCHITLMS-----      94 Amellifera(XP_003249162.1-SMYD4-like-Predicted)/239-589  15.1%     GK----RVIAAKNIEPGNRLIIE-SPHAAILLPE-F-----F--------------------GTHCQHCFS-RFK-----      95 Dmelanogaster(CG14122-NP_648574.1)/265-541               16.9%     GR----FVVANEGLRTGDVLLFE-EPVAACLEPS-Y-----F--------------------GTHCHHCFK-RLH-----      96 Agambiae(XP_311885.3-AGAP002999-PA)/268-544              17.3%     GR----YVVAAADLGPGEVILTE-PAYAACLHAK-Y-----Y--------------------GTHCSACFS-RLI-----      97 Amellifera(XP_392262.3-SMYD4-like-Predicted)/252-555     20.2%     GR----HAIATKDIEPGEILAIE-KPYSAFLLAE-Y-----R--------------------LINCFYCFT-KIF-----      98 Dmelanogaster(CG7759-NP_725048.1)/250-537                16.4%     GR----FARASADVKPGEELLVE-RPFVSVLLEK-F-----A--------------------KTHCENCFM-RTV-----      99 Agambiae(XP_319583.4-AGAP008839-PA)/240-523              16.0%     GR----FARTNTDLKPNTILLLE-RPHVSVLLED-Y-----S--------------------LDHCTHCFK-RVS-----     100 Dpulex(DAPPUDRAFT_68494-Predicted)/254-551               17.2%     GR----YGVAASPIRVGDVIAVD-APYASVMNPE-K-----F--------------------STHCHHCYQ-ILE-----     101 Dpulex(DAPPUDRAFT_309882)/300-599                        16.7%     GR----YYVAADDIKPGQTLVCE-KPYAACLLPG-K-----F--------------------TSHCHHCFV-RL------     102 Dmelanogaster(CG8378-NP_610730.1)/196-491                17.4%     GR----FVVTNRDLAVGDLVSVE-EPFCSTLLTP-M-----R--------------------YIRCATCKRENYL-----     103 Agambiae(XP_566179.1-AGAP000216-PA)/158-458              17.1%     GR----YLQTNKALKVGDVVMID-EPYVSVLEPE-F-----C--------------------YARCDHCQRPAPF-----     104 Agambiae(XP_564258.1-AGAP011234-PA)/216-546              17.1%     GR----HLVTTQHLKAGDVLMIE-KPYASLLCER-D-----Q--------------------YKRCAFCHNEDTF-----     105 Agambiae(XP_309407.4-AGAP011238-PA)/219-497              17.1%     GR----HLVTTQHLKAGDVLLIE-KPYANLLIDV-E-----R--------------------HVRCAFCQNEDRF-----     106 Agambiae(XP_314169.4-AGAP005253-PB)/218-514              16.6%     GR----HLVTTQHLKAGDVLLIE-KPYASMLNDK-E-----R--------------------YKRCAFCHNEDTF-----     107 Agambiae(XP_309409.4-AGAP011237-PA)/206-481              16.3%     GR----HLVTTQKLKVGDVLLIE-KPYASMLNDQ-E-----R--------------------YKRCDFCQNEDRF-----     108 Agambiae(XP_307865.2-AGAP009448-PA)/166-466              16.5%     GR----HVVTTRRLKVGDVVMLD-TPFVKTLHDP-L-----R--------------------HVRCDFCHAERPF-----     109 Agambiae(XP_309762.4-AGAP010931-PA)/113-383              17.2%     GR----HVVTKRKLKVGDVVMIE-KPFVTVAKET-F-----Q--------------------YIRCDFCQAKRLF-----     110 Agambiae(XP_309378.2-AGAP011267-PA)/149-447              17.9%     GR----HVVTTRKLKVGDVVMIE-RPFVTVLRDS-L-----R--------------------YVRCDFCHEERPF-----     111 Agambiae(XP_309383.4-AGAP011257-PA)/149-447              18.5%     GR----HVVTTRKLKVGDVVMIE-RPFVTVLKDS-F-----R--------------------YVRCDFCHGERPF-----     112 Agambiae(XP_307655.3-AGAP012638-PA)/149-447              18.5%     GR----HVVTTRKLKVGDVVMIE-RPFVTVLKDS-F-----R--------------------YVRCDFCHGERPF-----     113 Agambiae(XP_320681.4-AGAP011835-PA)/183-484              16.8%     GR----YVATNRNLEAGDVVIIE-QPFSRLLRDI-Y-----R--------------------HVRCDFCHRESIF-----     114 Agambiae(XP_309411.4-AGAP011232-PA)/162-434              16.4%     GR----HVVATRQLRVGDVVMVE-KPYATVLSDH-M-----K--------------------RVRCAFCHAEEPF-----     115 Amellifera(XP_001120776.2-SMYD4-like-Predicted)/251-554  19.0%     GR----HLVVTKEFKPGDIITIE-DPYAYVIYTQ-R-----Y--------------------YTHCHHCLS-RSY-----     116 Dpulex(DAPPUDRAFT_305694-Predicted)/258-553              19.7%     GR----CLVATEDIQIGTTVIVE-KALASILLEE-F-----K--------------------ESHCHHCLH-WTP-----     117 Dpulex(EFX87901.1)/258-554                               19.0%     GR----CLVATEDIKIGETVIVE-KAHASILQYE-F-----K--------------------ESHCHHCLH-WTP-----     118 Amellifera(XP_001122116.2-SMYD4-like-Predicted)/234-534  17.2%     GR----HIVATRKINPGEVIAIE-KPYSLILTPD-N-----I--------------------YTHCSNCLE-VSW-----     119 Hmagnipapillata(XP_002159692.1)/239-485                  18.1%     GR----HAIASRDIKAGEVIIIE-KPFASLCLPE-C-----Y--------------------NTHCYHCLT-RFK-----     120 Nvectensis(XP_001623892.1)/215-512                       18.8%     GR----HTIAARDINIGDVLLVE-KPFASVLLQE-Q-----S--------------------KSHCHQCFV-HIL-----     121 Lgigantea(LOTGIDRAFT_143433)/100-395                     19.3%     ------GIYTTKDVEAGELLFCE-KPFASKNMHN-S-----D--------------------LTHCQNCLN-RVL-----     122 Skowalevskii(XP_002740933.1)/253-549                     20.1%     GR----YAVATRDVKVGDVLIVE-NPYSSVGLQP-C-----N--------------------VSHCHHCYI-RVL-----         consensus/100%                                                     .................................................................s..h...........         consensus/90%                                                      Gp    hhhs.p.h..uphlh.p .sh..h.... .  . ..                 .....hC..Cht.........         consensus/80%                                                      G+    hhhstptht.Gphlh.- pshs.h.h.. .     .                   ...hCthChp...... .          consensus/70%                                                      GR    tlhuspplp.G-hlhhE pPhshs.h.. .     .                   .h.tCphChp......                                                                             81          .         1         .         .         .         .         :         . 160   1 cintestinalis(NP_001071820.1)/15-282                    100.0%     ---AP-------------------------------------------------------------------VEDLHRC-       2 Drerio(Q6P0R5-Smyd1a)/18-279                             35.9%     ------------------------------------------------------------------------QVNPHRC-       3 Derio(Q2MJQ9-Smyd1b)/13-274                              34.5%     ------------------------------------------------------------------------QEKLQRC-       4 Xtropicalis(NP_001120357.1-SMYD1)/13-261                 38.9%     ------------------------------------------------------------------------QEKLLRC-       5 Hsapiens(Q8NB12-SMYD1)/18-279                            38.4%     ------------------------------------------------------------------------QEKLHRC-       6 Ggallus(NP_989486.1-SMYD1)/13-274                        39.1%     ------------------------------------------------------------------------QERLHRC-       7 Athaliana(Q7XJS0-ASHR1)/22-274                           25.8%     -------------------------------------------------------------------------NNLKKC-       8 Amellifera(XP_625013.1-SMYD3-Predicted)/1-253            27.1%     -------------------------------------------------------------------------GKLFRC-       9 Dmelanogaster(Buzidau-CG13761)/26-282                    25.6%     -------------------------------------------------------------------------TKVLKC-      10 Agambiae(XP_319707.4-AGAP008954-PA)/1-254                25.0%     -------------------------------------------------------------------------TKVMKC-      11 cintestinalis(XP_002128556.1)/14-266                     32.4%     ------------------------------------------------------------------------QDGLLQC-      12 Lgigantea(LOTGIDRAFT_177746)/1-216                       26.2%     ------------------------------------------------------------------------CEKLKKC-      13 Drerio(E7EZZ6-SMYD3)/16-267                              34.7%     ------------------------------------------------------------------------GESLSRC-      14 Xtropicalis(XP_004914684.1|-SMYD3-Predicted)/15-264      31.0%     ------------------------------------------------------------------------KEKLLRC-      15 Hsapiens(Q9H7B4-SMYD3)/15-266                            33.9%     ------------------------------------------------------------------------KEKLMRC-      16 Ggallus(XP_419536.1-SMYD3-Predicted)/15-266              33.2%     ------------------------------------------------------------------------NEHLHRC-      17 Drerio(Q5RGL7-Smyd2b)/19-268                             30.9%     ------------------------------------------------------------------------KKGLAKC-      18 Drerio(Q5BJI7-Smyd2a)/18-267                             32.0%     ------------------------------------------------------------------------KEGLSKC-      19 Xtropicalis(XP_002934751.2-SMYD2-like-Predicted)/16-265  30.9%     ------------------------------------------------------------------------KEGLSKC-      20 Hsapiens(Q9NRG4-SMYD2)/18-267                            32.0%     ------------------------------------------------------------------------KEGLSKC-      21 Ggallus(XP_419420.1-SMYD2-Predicted)/21-270              31.6%     ------------------------------------------------------------------------KEGLSKC-      22 Tadhaerens(XP_002109888.1)/20-262                        28.4%     ------------------------------------------------------------------------QSELYKC-      23 Hmagnipapillata(XP_002163555.2)/16-259                   29.4%     ------------------------------------------------------------------------PEKLLKC-      24 Nvectensis(XP_001627600.1)/17-253                        32.1%     ------------------------------------------------------------------------LSDLQRC-      25 Bfloridae(XP_002594889.1-BRAFLDRAFT_124463)/14-258       38.4%     ------------------------------------------------------------------------KDDMSRC-      26 Skowalevskii(XP_006817727.1)/14-260                      33.2%     ------------------------------------------------------------------------TESLLRC-      27 Amellifera(XP_006565332.1)/43-285                        18.4%     ----------------------------------------------------------------------------FPC-      28 Dmelanogaster(msta-CG33548)/66-313                       17.7%     ----------------------------------------------------------------------------FLC-      29 Dmelanogaster(CG12119)/34-280                            19.0%     ----------------------------------------------------------------------------FMC-      30 Amellifera(XP_006565301.1)/26-284                        23.2%     ------------------------------------------------------------------------EDVNYVC-      31 Dmelanogaster(CG9642)/21-271                             18.8%     ---------------------------------------------------------------------------ESFC-      32 Dmelanogaster(CG9640)/17-268                             16.9%     ---------------------------------------------------------------------------YVIC-      33 Amellifera(NP_001229486.1-LOC724300)/57-301              21.5%     ----------------------------------------------------------------------------IRC-      34 Dmelanogaster(CG14590-NP_610202.3)/55-322                18.9%     ----------------------------------------------------------------------------HQC-      35 Dmelanogaster(CG43129)/21-279                            17.1%     --------------------------------------------------------------------------EEELC-      36 Dmelanogaster(G11160)/58-319                             20.8%     --------------------------------------------------------------------------DQYRC-      37 Amellifera(XP_624539.3-msta-like-Predicted)/54-297       21.5%     --------------------------------------------------------------------------IAIPC-      38 Dmelanogaster(CG8503-NP_610944.1)/52-301                 20.0%     --------------------------------------------------------------------------DHIEC-      39 Agambiae(XP_309979.4-AGAP011530-PA)/50-300               22.8%     --------------------------------------------------------------------------KYLDC-      40 Dpulex(DAPPUDRAFT_120473)/58-292                         19.1%     ------------------------------------------------------------------------EASLFRC-      41 Dpulex(DAPPUDRAFT_194440-Predicted)/53-302               19.6%     ----------------------------------------------------------------------------TRC-      42 Dpulex(DAPPUDRAFT_2393)/50-297                           20.2%     ----------------------------------------------------------------------------YRC-      43 Dmelanogaster(CG18136-NP_649084.1)/58-318                22.6%     ----------------------------------------------------------------------KPRGNYHKC-      44 Agambiae(XP_309220.5-AGAP001025-PA)/55-318               20.7%     ----------------------------------------------------------------------RGLDRFHEC-      45 Scerevisiae(P38890.1-SET5)/124-429                       16.1%     ----------------------------------------------------------------------KIMVHYLDC-      46 Athaliana(Q9ZUM9-ASHR2)/22-296                           14.0%     ----------------------------------------------------------------------------QKC-      47 Athaliana(Q9FG08.2-ATXR4)/53-321                         16.2%     ----------------------------------------------------------------------------FE--      48 Lgigantea(LOTGIDRAFT_232186)/323-670                     15.0%     GD-ALETMTEE----------EKE--------------------------------LVNIHW---------PDVTPIYC-      49 Bfloridae(XP_002589246.1-BRAFLDRAFT_74594)/380-720       14.2%     GMDTFRRMNKA----------QKA--------------------------------IIKKAW---------PKVTAYPC-      50 Athaliana(Q5PP37-ATXR2)/52-466                           14.1%     GRKLYFKNLGVSGCCDDDSSEEDECVKYNGNEEQCGGSSSSHNTLPEGVVSSLMNGEMALPHTDKF-----PLPSPLSC-      51 Cowczarzaki(EPH53581.1)/160-496                          15.7%     PFAVLHSEV--------------------------------------------------YPS---------GTPSFLSC-      52 Mbrevicollis(MONBRDRAFT_29283)/14-364                    16.5%     NRLTQHDLP--------------------------------------------------LPNPS-HQLEPDARIPQFSC-      53 Cowczarzaki(EFW42079.2)/57-422                           15.4%     ARLATAPTTVV----------------------------------AAGSAVAKPRTPFELPFMDQCNLTIATQQDIVTC-      54 Tadhaerens(XP_002114620.1)/25-373                        17.6%     RRLSGNPNV-------------------------------------------------VLPHKEYCSTYAM--ENYVKC-      55 Bfloridae(XP_002609030.1-BRAFLDRAFT_84846)/1-276         11.7%     RRLANSPSL-------------------------------------------------VLPFPQ-CCAVKL--EQHVTC-      56 cintestinalis(XP_002127168.1)/13-358                     17.5%     KRLAQNPGL-------------------------------------------------KLPYHE-CCESNP--VTYVHC-      57 Dpulex(EFX89935.1)/23-367                                16.0%     RRLTAKADL-------------------------------------------------ILPHPE-CDGTDK--SSHVIC-      58 Dmelanogaster(CG3353-NP_650955.1)/13-363                 15.5%     RRLASDPKV-------------------------------------------------EVPLLQ-HDPTAQWVAQFTQC-      59 Hmagnipapillata(XP_002163562.2)/21-371                   16.0%     RRLAADYTL-------------------------------------------------ELPYHEQCSLSVQRVNSIYKC-      60 Agambiae(XP_313299.1-AGAP003552-PA)/13-365               16.8%     QRLANDPCI-------------------------------------------------MLPRTE-CCPVEANLANHTKC-      61 Amellifera(XP_394075.2-SMYD5-like-Prediction)/16-364     16.1%     HRLTGNSTI-------------------------------------------------ILPHAE-CCETKK--ELITEC-      62 Nvectensis(XP_001627062.1)/18-370                        15.2%     RRLSSNPTL-------------------------------------------------ELPYSAECCAVTKAGEPISYC-      63 Skowalevskii(XP_002735533.1)/24-372                      13.9%     RRLSAISSL-------------------------------------------------VLPYPQ-CCEVKK--DEHVSC-      64 Lgigantea(LOTGIDRAFT_231752)/19-367                      16.4%     RRLTNNPAL-------------------------------------------------SLPHPE-CCALDP--SEFVVC-      65 Drerio(F1RET2-Smyd5)/32-380                              16.4%     RRLSGLPAL-------------------------------------------------ILPHPE-LCKVRP--DRHQAC-      66 Ggallus(NP_001012912.1-SMYD5)/39-387                     16.9%     QRLLGRSSL-------------------------------------------------VLPHPE-QCSIRK--DLHQQC-      67 Hsapiens(Q6GMV2-SMYD5)/33-381                            15.8%     QRLTGKPGQ-------------------------------------------------VLPHPE-LCTVRK--DLHQNC-      68 Xtropicalis(A9ULL8-SMyd5)/32-382                         16.9%     QRLSGNAHV-------------------------------------------------VLPYPE-LCTVRN--GLHQQC-      69 Mbrevicollis(MONBRDRAFT_36878)/153-462                   16.1%     ---AP------------------------------------------------------LP-----------------C-      70 Mbrevicollis(MONBRDRAFT_27776)/11-280                    20.3%     ---RL------------------------------------------------------AN-----------------C-      71 Cowczarzaki(EFW45970.2)/35-344                           21.5%     AAAEM------------------------------------------------------KR-----------------C-      72 Dpulex(EFX73755.1)/45-306                                19.5%     ---NL------------------------------------------------------RK-----------------C-      73 Bfloridae(XP_002593048.1-BRAFLDRAFT_74375)/6-196         17.6%     ---LL------------------------------------------------------LT-----------------C-      74 Bfloridae(XP_002594298.1-BRAFLDRAFT_117670)/15-265       21.7%     ---NS------------------------------------------------------VS-----------------C-      75 Scerevisiae(Q12529.1)/23-365                             10.9%     ---KT------------------------------------------------------MKYKLNY-----DYLRDLVCN      76 Dmelanogaster(CG1868-NP_724802.1)/226-549                15.1%     ---AP------------------------------------------------------IP-----------------C-      77 Agambiae(XP_319721.4-AGAP008973-PA)/165-486              14.6%     ---VP------------------------------------------------------FP-----------------C-      78 Athaliana(NP_174606.2)/229-550                           12.6%     ---DT------------------------------------------------------VP-----------------C-      79 Cintestinalis(XP_002123001.1)/195-567                    14.8%     ---SG------------------------------------------------------IP-----------------C-      80 Drerio(Q08C84-Smyd4)/197-556                             15.4%     ---SF------------------------------------------------------VP-----------------C-      81 Xtropicalis(NP_001072288.1-SMYD4)/212-545                18.0%     ---AS------------------------------------------------------LP-----------------C-      82 Hsapiens(Q8IYR2-SMYD4)/244-602                           16.3%     ---AT------------------------------------------------------VP-----------------C-      83 Ggallus(NP_001025886.1-SMYD4)/241-573                    15.3%     ---AS------------------------------------------------------IP-----------------C-      84 Hmagnipapillata(XP_002160254.2/232-532                   15.5%     ---SL------------------------------------------------------FP-----------------C-      85 Dpulex(DAPPUDRAFT_312722-Pedicted)/241-525               17.9%     ---KR------------------------------------------------------YP-----------------C-      86 Amellifera(XP_006565387.1-SMYD4-like-Predicted)/278-571  17.3%     ---IP------------------------------------------------------VP-----------------C-      87 Bfloridae(XP_002589088.1-BRAFLDRAFT_75068)/251-714       13.4%     ---VP------------------------------------------------------HP-----------------C-      88 Lgigantea(LOTGIDRAFT_169490)/248-638                     12.9%     ---NL------------------------------------------------------VG-----------------C-      89 Skowalevskii(XP_002733823.1)/75-447                      16.7%     ---AP------------------------------------------------------IP-----------------C-      90 Nvectensis(XP_001627273.1)/170-547                       14.3%     ---AP------------------------------------------------------VP-----------------C-      91 Amellifera(XP_003250668.1-SMYD4-like-Predicted)/183-473  15.8%     ---AL------------------------------------------------------IP-----------------C-      92 Cowczarzaki(XP_004349923.1)/103-371                      17.2%     ---LT------------------------------------------------------VR-----------------C-      93 Amellifera(XP_001121272.2-SMYD4-like-Predicted)/230-549  19.0%     SNSVK------------------------------------------------------IP-----------------C-      94 Amellifera(XP_003249162.1-SMYD4-like-Predicted)/239-589  15.1%     ---AP------------------------------------------------------IG-----------------C-      95 Dmelanogaster(CG14122-NP_648574.1)/265-541               16.9%     ---TP------------------------------------------------------VS-----------------C-      96 Agambiae(XP_311885.3-AGAP002999-PA)/268-544              17.3%     ---AP------------------------------------------------------VA-----------------C-      97 Amellifera(XP_392262.3-SMYD4-like-Predicted)/252-555     20.2%     -VPIP------------------------------------------------------AV-----------------C-      98 Dmelanogaster(CG7759-NP_725048.1)/250-537                16.4%     -VP--------------------------------------------------------VA-----------------C-      99 Agambiae(XP_319583.4-AGAP008839-PA)/240-523              16.0%     -VP--------------------------------------------------------IA-----------------C-     100 Dpulex(DAPPUDRAFT_68494-Predicted)/254-551               17.2%     -LGEV------------------------------------------------------LP-----------------C-     101 Dpulex(DAPPUDRAFT_309882)/300-599                        16.7%     --IAP------------------------------------------------------LG-----------------C-     102 Dmelanogaster(CG8378-NP_610730.1)/196-491                17.4%     ---TL------------------------------------------------------IP-----------------C-     103 Agambiae(XP_566179.1-AGAP000216-PA)/158-458              17.1%     ---TL------------------------------------------------------IP-----------------C-     104 Agambiae(XP_564258.1-AGAP011234-PA)/216-546              17.1%     ---TL------------------------------------------------------IP-----------------C-     105 Agambiae(XP_309407.4-AGAP011238-PA)/219-497              17.1%     ---TL------------------------------------------------------IP-----------------C-     106 Agambiae(XP_314169.4-AGAP005253-PB)/218-514              16.6%     ---TL------------------------------------------------------IP-----------------C-     107 Agambiae(XP_309409.4-AGAP011237-PA)/206-481              16.3%     ---TL------------------------------------------------------IP-----------------C-     108 Agambiae(XP_307865.2-AGAP009448-PA)/166-466              16.5%     ---TL------------------------------------------------------IP-----------------C-     109 Agambiae(XP_309762.4-AGAP010931-PA)/113-383              17.2%     ---TL------------------------------------------------------IP-----------------C-     110 Agambiae(XP_309378.2-AGAP011267-PA)/149-447              17.9%     ---TL------------------------------------------------------IP-----------------C-     111 Agambiae(XP_309383.4-AGAP011257-PA)/149-447              18.5%     ---TL------------------------------------------------------IP-----------------C-     112 Agambiae(XP_307655.3-AGAP012638-PA)/149-447              18.5%     ---TL------------------------------------------------------IP-----------------C-     113 Agambiae(XP_320681.4-AGAP011835-PA)/183-484              16.8%     ---TL------------------------------------------------------LP-----------------C-     114 Agambiae(XP_309411.4-AGAP011232-PA)/162-434              16.4%     ---LL------------------------------------------------------IP-----------------C-     115 Amellifera(XP_001120776.2-SMYD4-like-Predicted)/251-554  19.0%     ---NL------------------------------------------------------IP-----------------C-     116 Dpulex(DAPPUDRAFT_305694-Predicted)/258-553              19.7%     ---GP------------------------------------------------------VP-----------------C-     117 Dpulex(EFX87901.1)/258-554                               19.0%     ---GP------------------------------------------------------VP-----------------C-     118 Amellifera(XP_001122116.2-SMYD4-like-Predicted)/234-534  17.2%     ---AN------------------------------------------------------IP-----------------C-     119 Hmagnipapillata(XP_002159692.1)/239-485                  18.1%     ---IN------------------------------------------------------YP-----------------C-     120 Nvectensis(XP_001623892.1)/215-512                       18.8%     ---AP------------------------------------------------------LP-----------------C-     121 Lgigantea(LOTGIDRAFT_143433)/100-395                     19.3%     ---SP------------------------------------------------------LP-----------------C-     122 Skowalevskii(XP_002740933.1)/253-549                     20.1%     ---AS------------------------------------------------------IP-----------------C-         consensus/100%                                                     ................................................................................         consensus/90%                                                      .........                                                 ...... .............C          consensus/80%                                                       ....                                                      ..           ......C          consensus/70%                                                         ..                                                      ..           ......C                                                                          161          .         .         .         2         .         .         .         . 240   1 cintestinalis(NP_001071820.1)/15-282                    100.0%     --------TG-CK-F--AQYCTK----------------------E-CQKKAW--PE----HKQ-ECAAIKRIT------       2 Drerio(Q6P0R5-Smyd1a)/18-279                             35.9%     --------AQ-CK-F--AHYCDR----------------------T-CQRAAW--DE----HRK-ECSAIRNIG------       3 Derio(Q2MJQ9-Smyd1b)/13-274                              34.5%     --------GQ-CR-F--AQYCDK----------------------T-CQRAGW--EE----HKL-ECAAIKTYG------       4 Xtropicalis(NP_001120357.1-SMYD1)/13-261                 38.9%     --------GQ-CK-F--AHYCDR----------------------T-CQKESW--AN----HKN-ECVAIKKAG------       5 Hsapiens(Q8NB12-SMYD1)/18-279                            38.4%     --------GQ-CK-F--AHYCDR----------------------T-CQKDAW--LN----HKN-ECSAIKRYG------       6 Ggallus(NP_989486.1-SMYD1)/13-274                        39.1%     --------GQ-CK-F--AYYCDR----------------------T-CQRDAW--LN----HKN-ECSAIKKHG------       7 Athaliana(Q7XJS0-ASHR1)/22-274                           25.8%     --------SA-CQ-V--VWYCGS----------------------S-CQKSEW--KL----HRD-ECKALTRLE------       8 Amellifera(XP_625013.1-SMYD3-Predicted)/1-253            27.1%     --------SV-CK-C--IYYCNQ----------------------S-CQQMSW--TI----HSK-ECASLKRFS------       9 Dmelanogaster(Buzidau-CG13761)/26-282                    25.6%     --------SN-CR-Y--VSYCHR----------------------S-CQMQAW--GQ----HKH-ECPFLKKVH------      10 Agambiae(XP_319707.4-AGAP008954-PA)/1-254                25.0%     --------SN-CL-Y--VRYCGR----------------------S-CQKEAW--SD----HKE-ECEKLKALP------      11 cintestinalis(XP_002128556.1)/14-266                     32.4%     --------SG-CK-Y--MKYCNR----------------------N-CQKMAW--NE---HHKA-ECPALKNVM------      12 Lgigantea(LOTGIDRAFT_177746)/1-216                       26.2%     --------AA-CG-L--VKYCGV----------------------V-CQKADW--PI----HKT-ECPCFKESQ------      13 Drerio(E7EZZ6-SMYD3)/16-267                              34.7%     --------SQ-CK-T--ARYCSV----------------------Q-CQKQAW--PD----HKR-ECKCLKHLQ------      14 Xtropicalis(XP_004914684.1|-SMYD3-Predicted)/15-264      31.0%     --------SQ-CK-V--TRYCNS----------------------H-CQRKAW--QG----HKR-ECKCLRSTL------      15 Hsapiens(Q9H7B4-SMYD3)/15-266                            33.9%     --------SQ-CR-V--AKYCSA----------------------K-CQKKAW--PD----HKR-ECKCLKSCK------      16 Ggallus(XP_419536.1-SMYD3-Predicted)/15-266              33.2%     --------SQ-CK-V--AKYCGK----------------------S-CQKEAW--LD----HKR-ECKCLQNVK------      17 Drerio(Q5RGL7-Smyd2b)/19-268                             30.9%     --------GK-CK-K--AFYCNA----------------------N-CQKKNW--PM----HKL-ECQAMCAFG------      18 Drerio(Q5BJI7-Smyd2a)/18-267                             32.0%     --------GK-CK-Q--AYYCNV----------------------E-CQRGDW--PM----HKL-ECSAMCAYG------      19 Xtropicalis(XP_002934751.2-SMYD2-like-Predicted)/16-265  30.9%     --------GK-CK-Q--AFYCNV----------------------D-CQKGDW--PM----HKL-ECSAMCTYG------      20 Hsapiens(Q9NRG4-SMYD2)/18-267                            32.0%     --------GR-CK-Q--AFYCNV----------------------E-CQKEDW--PM----HKL-ECSPMVVFG------      21 Ggallus(XP_419420.1-SMYD2-Predicted)/21-270              31.6%     --------GR-CK-Q--AFYCNV----------------------E-CQKEDW--PM----HKL-ECAAMCAFG------      22 Tadhaerens(XP_002109888.1)/20-262                        28.4%     --------SR-CK-I--IMYCCK----------------------S-CQKEDW--QW----HKY-ECKSITRLG------      23 Hmagnipapillata(XP_002163555.2)/16-259                   29.4%     --------SK-CK-F--IAYCGK----------------------V-CQASDW--KM----HKY-ECKCLTKSA------      24 Nvectensis(XP_001627600.1)/17-253                        32.1%     --------SR-CK-F--ARYCGA----------------------S-CQRAAW--RI----HKS-ECERLKRVF------      25 Bfloridae(XP_002594889.1-BRAFLDRAFT_124463)/14-258       38.4%     --------SG-CK-F--ARYCDG----------------------K-CQKAAW--TE----HKS-ECKSIKTVK------      26 Skowalevskii(XP_006817727.1)/14-260                      33.2%     --------SS-CK-F--SRYCNV----------------------K-CQRSAW--TC----HKA-ECKSLKKVS------      27 Amellifera(XP_006565332.1)/43-285                        18.4%     --------DKGCG----LPVCSI----------------------Q-CENSPN--------HVN-ECEYLRSLIPTCGTD      28 Dmelanogaster(msta-CG33548)/66-313                       17.7%     --------RHRCT----LPVCET------------------------CSDSEE--------HQA-ECEHFRRWQPKDVDA      29 Dmelanogaster(CG12119)/34-280                            19.0%     --------RQGCG----LPVCSL------------------------CAKKKQ--------HKS-DCDLFKSWGPNEPDV      30 Amellifera(XP_006565301.1)/26-284                        23.2%     --------S-GCN-V--VTLCGV----------------------T-CEERG----I---YHSAYECEIIKNNEELS---      31 Dmelanogaster(CG9642)/21-271                             18.8%     --------R-KCR-L--LALCED----------------------C-S------------DHDERDCKRLAEMNFSDDQV      32 Dmelanogaster(CG9640)/17-268                             16.9%     --------R-RCQ-V--FPLCMD----------------------C-N------------QHDEFECEFFTSGAGKALCK      33 Amellifera(NP_001229486.1-LOC724300)/57-301              21.5%     --------T-KCL----WPACAV----------------------D-CSGLTD--KN---RHDL-ECSFLIKAK------      34 Dmelanogaster(CG14590-NP_610202.3)/55-322                18.9%     --------R-RCR----WPVCSA----------------------G-CKHES------------MECSVLSLGSGSPTRA      35 Dmelanogaster(CG43129)/21-279                            17.1%     --------S-ECG----WPLCVE----------------------C--AQQAD---N---AHFRLECSQLKDARARFFRL      36 Dmelanogaster(G11160)/58-319                             20.8%     --------P-GCA----WPLCGS----------------------T-CAGLKH--RH---GHTETECQLYAERRAVAGEL      37 Amellifera(XP_624539.3-msta-like-Predicted)/54-297       21.5%     --------E-KCG----WPLCQ---------------------------NCNE--------HGL-ECKFSSSRRDSKISI      38 Dmelanogaster(CG8503-NP_610944.1)/52-301                 20.0%     --------E-QCG----WPLCGP----------------------E-CKSLDE--------HKA-ECGLTKD-RGQKVNV      39 Agambiae(XP_309979.4-AGAP011530-PA)/50-300               22.8%     --------E-RCG----WPVCKR----------------------S-CQDSPS--------HQA-ECKFTIA-RGSKISI      40 Dpulex(DAPPUDRAFT_120473)/58-292                         19.1%     --------P-NCN----FPFCQE----------------------Q-CAKSPE--------HEA-ECLILSR-AKSCIVI      41 Dpulex(DAPPUDRAFT_194440-Predicted)/53-302               19.6%     --------S-QCG----WPMCGR----------------------DDCHAHES--------DHAAECGVMAS-GGR----      42 Dpulex(DAPPUDRAFT_2393)/50-297                           20.2%     --------S-RCN----WPLCSA----------------------A-CEESAL--------HKNGECRMIDP-TLMTNHL      43 Dmelanogaster(CG18136-NP_649084.1)/58-318                22.6%     --------S-SCS----WPLCGK----------------------E-CEDSVH--------HKA-ECQLMSG-SNFQSKI      44 Agambiae(XP_309220.5-AGAP001025-PA)/55-318               20.7%     --------S-RCG----WPLCGP----------------------G-CEEVAQ--------HRP-ECSVLAG-SGYRPNI      45 Scerevisiae(P38890.1-SET5)/124-429                       16.1%     --------E-VCK----AIWCSE----------------------K-CKKAHA----------SLHELLYHSWRSNRIDI      46 Athaliana(Q9ZUM9-ASHR2)/22-296                           14.0%     --------Q-SCS-L--VSFCSP----------------------N-CFASHT----------PW--------LCESLRR      47 Athaliana(Q9FG08.2-ATXR4)/53-321                         16.2%     -----------DR-G--VSYCSQ----------------------E-CQENSK----------GF--------LDVETRA      48 Lgigantea(LOTGIDRAFT_232186)/323-670                     15.0%     --------DD-CR-R--VKYCSD----------------------D-CRLEAW--DL---YHQI-ICPK-----LNPASS      49 Bfloridae(XP_002589246.1-BRAFLDRAFT_74594)/380-720       14.2%     --------PH-CK-R--EKYCSL----------------------E-CRTHAW--RQ---HHCH-LCPS-----INPPAA      50 Athaliana(Q5PP37-ATXR2)/52-466                           14.1%     --------PGGCQ-E--AFYCSE----------------------S-CAAADW--ES---SHSL-LCTGERSESIS--RE      51 Cowczarzaki(EPH53581.1)/160-496                          15.7%     --------EK-CF-VPYEQYCSA----------------------K-CRTQAW--DD---YHSV-FCAKDRELAKIHPIT      52 Mbrevicollis(MONBRDRAFT_29283)/14-364                    16.5%     --------PG-CP-E---TYCSE----------------------A-CLEA-D--QV---FHAH-LCPR-----AHPTVE      53 Cowczarzaki(EFW42079.2)/57-422                           15.4%     --------ET-CS-L---VFCNA----------------------A-CRDAAM--ES---HHRI-LCTR---NDADHPLQ      54 Tadhaerens(XP_002114620.1)/25-373                        17.6%     --------PR-CQ-V--TMYCSS----------------------T-CLEKAV--KE---YHRS-LCCGSNNCRPDHSLN      55 Bfloridae(XP_002609030.1-BRAFLDRAFT_84846)/1-276         11.7%     --------PH-CQ-K---YTLP----------------------------SRN--GQ---YHDD-C-QN-----DSHGQA      56 cintestinalis(XP_002127168.1)/13-358                     17.5%     --------Q---N-E---IYCSM----------------------E-CREKAY--NE---FHKI-LCPSSDLID-RNALE      57 Dpulex(EFX89935.1)/23-367                                16.0%     --------SQ-CA-V---TYCSV----------------------D-CKDQAW--NQ---YHKT-ICCNMFGGNSNHPLE      58 Dmelanogaster(CG3353-NP_650955.1)/13-363                 15.5%     --------PR-CK-V---RYCSE----------------------D-CLMEAQ--KR---YHRV-ACMGAFHSDDTHPIN      59 Hmagnipapillata(XP_002163562.2)/21-371                   16.0%     --------PN-CC-I---PFCSK----------------------E-CYSEAY--EK---YHKS-LCLHPDEM-SESPVY      60 Agambiae(XP_313299.1-AGAP003552-PA)/13-365               16.8%     --------ER-CG-A---LYCSA----------------------D-CLQEAA--NR---YHTA-VCLGSKAHNEQHPVN      61 Amellifera(XP_394075.2-SMYD5-like-Prediction)/16-364     16.1%     --------SE-CG-T---KYCSI----------------------E-CQTDAY--LR---YHST-ICLQSREKDESHPLV      62 Nvectensis(XP_001627062.1)/18-370                        15.2%     --------PQ-CN-V---AYCSE----------------------N-CRIKAL--DQ---YHRI-LCLGTSTPDPNHPLV      63 Skowalevskii(XP_002735533.1)/24-372                      13.9%     --------PA-CQ-T---QYCST----------------------K-CKEDAE--KL---YHRV-LCMGQHPADPEHPIA      64 Lgigantea(LOTGIDRAFT_231752)/19-367                      16.4%     --------PQ-CQ-V---LYCSE----------------------E-CRKASW--DR---YHQI-LCLGSSHHDSDHPLL      65 Drerio(F1RET2-Smyd5)/32-380                              16.4%     --------PQ-CQ-V---MYCSS----------------------E-CRQAAM--DQ---YHKI-LCLGPSNDDPDHPVN      66 Ggallus(NP_001012912.1-SMYD5)/39-387                     16.9%     --------PR-CQ-V---TYCSA----------------------E-CRQAAL--EQ---YHQV-LCLGPSRDDPTHPLN      67 Hsapiens(Q6GMV2-SMYD5)/33-381                            15.8%     --------PH-CQ-V---MYCSA----------------------E-CRLAAT--EQ---YHQV-LCPGPSQDDPLHPLN      68 Xtropicalis(A9ULL8-SMyd5)/32-382                         16.9%     --------PR-CQ-V---TYCSA----------------------E-CLKAAA--DQ---YHRA-LCLGASRDNPAHPLN      69 Mbrevicollis(MONBRDRAFT_36878)/153-462                   16.1%     --------PN-CP-R---VFCST----------------------E-CHAKAN--H----WHLE-ACPATPP--------      70 Mbrevicollis(MONBRDRAFT_27776)/11-280                    20.3%     --------SL-CH-T--AAYCSK----------------------P-CQTRNW--KR---AHKH-VCKLLQT--------      71 Cowczarzaki(EFW45970.2)/35-344                           21.5%     --------AQ-CR-R--AQYCSV----------------------E-CQRAAW--HG---GHKA-ECAAWVR--------      72 Dpulex(EFX73755.1)/45-306                                19.5%     --------LG-CM-V--VSYCGR----------------------V-CQREGW--K----DHKG-ECKNFVR--------      73 Bfloridae(XP_002593048.1-BRAFLDRAFT_74375)/6-196         17.6%     --------PK-CG-I--AKYCDE----------------------D-CQSARK--Y------------------------      74 Bfloridae(XP_002594298.1-BRAFLDRAFT_117670)/15-265       21.7%     --------DA-CR-T--AKYCNE----------------------E-CKKAAK--F----HHTP-ECRGYSR--------      75 Scerevisiae(Q12529.1)/23-365                             10.9%     AHYQINPKKF-LGAG--LWFCSE----------------------H-CRTSYL--QIPNIIELI-ECYEILLHH------      76 Dmelanogaster(CG1868-NP_724802.1)/226-549                15.1%     --------PN-CHQR--VVYCSR----------------------K-CRE-AH--SA---IHKF-ECAAYRKDI------      77 Agambiae(XP_319721.4-AGAP008973-PA)/165-486              14.6%     --------PT-CG-R--ACYCST----------------------R-CRV-AH--RP---VHRF-ECFGYQKHL------      78 Athaliana(NP_174606.2)/229-550                           12.6%     --------PS-CS-I--PVYCSE----------------------S-CQIQSG--GM---LSTN-EMDKHHI--------      79 Cintestinalis(XP_002123001.1)/195-567                    14.8%     --------LG-CS-C--TIYCDE----------------------Q-CRISAW--KI---YHWM-ECSVIPM--------      80 Drerio(Q08C84-Smyd4)/197-556                             15.4%     --------PK-CS-Y--ARYCGE----------------------S-CQKDAW--DQ---WHQW-ECPVGAD--------      81 Xtropicalis(NP_001072288.1-SMYD4)/212-545                18.0%     --------QY-CS-F--ARYCSQ----------------------E-CMDKAW--RS---YHYI-ECSMGDL--------      82 Hsapiens(Q8IYR2-SMYD4)/244-602                           16.3%     --------DG-CS-Y--AKYCSQ----------------------E-CLQQAW--EL---YHRT-ECPLGGL--------      83 Ggallus(NP_001025886.1-SMYD4)/241-573                    15.3%     --------CG-CS-Y--AKYCSQ----------------------N-CADVAW--EQ---YHRT-ECPLGAL--------      84 Hmagnipapillata(XP_002160254.2/232-532                   15.5%     --------YE-CA-E--VVFCSL----------------------S-CYNDAW--AT---YHRF-ECKKLSL--------      85 Dpulex(DAPPUDRAFT_312722-Pedicted)/241-525               17.9%     --------LA-CG-K--IWFCSD----------------------S-CRQE-S--SC---YHNF-ECGLEAV--------      86 Amellifera(XP_006565387.1-SMYD4-like-Predicted)/278-571  17.3%     --------RT-CL-N--TFYCNE----------------------N-CLTKAW--SS---YHCW-ECPGNQMNL------      87 Bfloridae(XP_002589088.1-BRAFLDRAFT_75068)/251-714       13.4%     --------RG-CQ-Y--VQYCSGTCEEQAWREYHRGCQYVQYCSRT-CEDQAW--KE---YHSY-ECEHWHL--------      88 Lgigantea(LOTGIDRAFT_169490)/248-638                     12.9%     --------IQ-CS-V--VRYCSS----------------------K-CQEESW--KL---YHSV-ECPYLDL--------      89 Skowalevskii(XP_002733823.1)/75-447                      16.7%     --------CT-CT-H--VRYCSV----------------------E-CQQESW--KS---YHYI-ECPLWPF--------      90 Nvectensis(XP_001627273.1)/170-547                       14.3%     --------NH-CS-S--VLYCSA----------------------A-CRNKAW--SQ---YHHV-ECEIFPV--------      91 Amellifera(XP_003250668.1-SMYD4-like-Predicted)/183-473  15.8%     --------DY-CY-H--ALYCSK----------------------E-CRGKAY--QA---YHQI-YCRYGNL--------      92 Cowczarzaki(XP_004349923.1)/103-371                      17.2%     --------EK-CV-Q--VYFCST----------------------A-CRDESKARDL---HSEI-ECNALAF--------      93 Amellifera(XP_001121272.2-SMYD4-like-Predicted)/230-549  19.0%     --------YY-CQ-T--VSFCSE----------------------K-CRSKAW--QI---YHQY-ECFIFDV--------      94 Amellifera(XP_003249162.1-SMYD4-like-Predicted)/239-589  15.1%     --------PD-CS-S--VAFCGR----------------------K-CRDTAL--AS---YHKY-ECKILVL--------      95 Dmelanogaster(CG14122-NP_648574.1)/265-541               16.9%     --------LH-CS-G--IAFCSA----------------------Q-CMGEAC--SS---YHRF-ECEYMDL--------      96 Agambiae(XP_311885.3-AGAP002999-PA)/268-544              17.3%     --------PD-CC-G--VAFCSV----------------------A-CRDKAC--AT---YHRF-ECQYLDL--------      97 Amellifera(XP_392262.3-SMYD4-like-Predicted)/252-555     20.2%     --------QT-CS-C--VAYCSI----------------------S-CRDKD---AK---IHEN-ECSILPT--------      98 Dmelanogaster(CG7759-NP_725048.1)/250-537                16.4%     --------PR-CA-D--VLYCSE----------------------Q-CREEAS--KK---YHKY-ECGIVPI--------      99 Agambiae(XP_319583.4-AGAP008839-PA)/240-523              16.0%     --------PL-CA-D--VVFCSD----------------------E-CETKAN--AT---YHRY-ECGFLPI--------     100 Dpulex(DAPPUDRAFT_68494-Predicted)/254-551               17.2%     --------SH-CD-L--VSFCSV----------------------N-CRSRAM--EI---YHAI-ECPILSC--------     101 Dpulex(DAPPUDRAFT_309882)/300-599                        16.7%     --------LT-CR-G--VFYCSV----------------------E-CRDEAA--ST---YHQY-ECGIIDY--------     102 Dmelanogaster(CG8378-NP_610730.1)/196-491                17.4%     --------DS-CC-S--TMFCSE----------------------E-CKSIAM--QT---YHRY-ECPIIDF--------     103 Agambiae(XP_566179.1-AGAP000216-PA)/158-458              17.1%     --------ER-CT-K--AMYCSK----------------------N-CLRRAR--TE---YHEF-ECALVHH--------     104 Agambiae(XP_564258.1-AGAP011234-PA)/216-546              17.1%     --------EG-CT-V--AMYCSE----------------------E-CRDKAH--KQ---YHRY-ECAVLRD--------     105 Agambiae(XP_309407.4-AGAP011238-PA)/219-497              17.1%     --------EG-CT-V--TMYCSE----------------------E-CRDKAH--KQ---YHRY-ECGVLRD--------     106 Agambiae(XP_314169.4-AGAP005253-PB)/218-514              16.6%     --------EG-CT-L--TMYCSD----------------------E-CMDKAY--KQ---YHRY-ECGVLRD--------     107 Agambiae(XP_309409.4-AGAP011237-PA)/206-481              16.3%     --------EG-CT-V--TMYCSK----------------------E-CMDKAH--KQ---YHRY-ECGVLRD--------     108 Agambiae(XP_307865.2-AGAP009448-PA)/166-466              16.5%     --------EG-CT-W--VMYCSA----------------------E-CLGKAY--SQ---YHRY-ECGVMRD--------     109 Agambiae(XP_309762.4-AGAP010931-PA)/113-383              17.2%     --------EG-CT-V--AMYCSE----------------------E-CISKAY--GK---YHRY-ECGVLRD--------     110 Agambiae(XP_309378.2-AGAP011267-PA)/149-447              17.9%     --------EG-CT-A--AMYCSE----------------------E-CLSKAY--NK---YHRY-ECGLLRD--------     111 Agambiae(XP_309383.4-AGAP011257-PA)/149-447              18.5%     --------EG-CT-A--AMYCSE----------------------E-CLSKAY--NN---YHRY-DCGILRD--------     112 Agambiae(XP_307655.3-AGAP012638-PA)/149-447              18.5%     --------EG-CT-M--AMYCSE----------------------E-CLSKAY--NK---YHRY-ECGLLRD--------     113 Agambiae(XP_320681.4-AGAP011835-PA)/183-484              16.8%     --------EN-CT-V--AMYCSG----------------------S-CASQAA--RQ---YHRY-ECPIIRD--------     114 Agambiae(XP_309411.4-AGAP011232-PA)/162-434              16.4%     --------EE-CT-I--AMYCSQ----------------------K-CLRAAW--QQ---YHRY-ECPILND--------     115 Amellifera(XP_001120776.2-SMYD4-like-Predicted)/251-554  19.0%     --------LH-CP-V--AQYCSE----------------------K-CRILAW--EM---AHDI-ECPIMAL--------     116 Dpulex(DAPPUDRAFT_305694-Predicted)/258-553              19.7%     --------HQ-CS-Q--VGFCST----------------------L-CRDEAW--AS---YHQS-ECGLTDS--------     117 Dpulex(EFX87901.1)/258-554                               19.0%     --------HK-CS-Q--VGFCST----------------------Q-CRDEAW--DS---YHQF-ECGLTDF--------     118 Amellifera(XP_001122116.2-SMYD4-like-Predicted)/234-534  17.2%     --------EY-CT-Y--AMYCSE----------------------E-CKAMEW--KK---YHDI-ECAIFPS--------     119 Hmagnipapillata(XP_002159692.1)/239-485                  18.1%     --------RL-CS-T--VNYCSI----------------------S-CEKESW--EK---FHCF-ECEYLGV--------     120 Nvectensis(XP_001623892.1)/215-512                       18.8%     --------SY-CT-T--VRYCSE----------------------K-CAKESW--DA---YHYA-ECMNLEH--------     121 Lgigantea(LOTGIDRAFT_143433)/100-395                     19.3%     --------DQ-CS-G--VVFCSE----------------------E-CKAEAM--KS---FHFA-ECRVLET--------     122 Skowalevskii(XP_002740933.1)/253-549                     20.1%     --------LQ-CA-G--IVYCSK----------------------E-CRNASW--EM---YHNL-ECHHLDL--------         consensus/100%                                                     ...................hh...........................................................         consensus/90%                                                              ...Ct .  ..hCs.                      t C.t.t.  ..   .H.. .C.............         consensus/80%                                                              .. Ct .  h.aCs.                      p C.ptsh  ..   .Hp. tC.hh..........         consensus/70%                                                              .t Cp h  hhaCut                      p Cpppuh  t.   .Hph EC.hhp.........                                                                         241          :         .         .         .         .         3         .         . 320   1 cintestinalis(NP_001071820.1)/15-282                    100.0%     ------PG-------------KPVD-----------QTRLVGRILWRRKREEN----L---NGEK---------------       2 Drerio(Q6P0R5-Smyd1a)/18-279                             35.9%     -------K-------------APNE-----------NVRLVARILWRIQKHTG----L---VS-----------------       3 Derio(Q2MJQ9-Smyd1b)/13-274                              34.5%     -------K-------------PPSE-----------NVRLAARILWRMDKQGS----V---VS-----------------       4 Xtropicalis(NP_001120357.1-SMYD1)/13-261                 38.9%     -------K-------------APNE-----------NIRLAARILWRIEREGS----G---LT-----------------       5 Hsapiens(Q8NB12-SMYD1)/18-279                            38.4%     -------K-------------VPNE-----------NIRLAARIMWRVEREGT----G---LT-----------------       6 Ggallus(NP_989486.1-SMYD1)/13-274                        39.1%     -------K-------------APTE-----------NIRLAARILWRIEREGG----G---LS-----------------       7 Athaliana(Q7XJS0-ASHR1)/22-274                           25.8%     ------KEKRK----------FVTP-----------TIRLMVRLYIKRNLQNE----K---VLP----------------       8 Amellifera(XP_625013.1-SMYD3-Predicted)/1-253            27.1%     ------SK--V-----------IPD-----------VARLMARIIIKLNQGGG----E---EIG----------------       9 Dmelanogaster(Buzidau-CG13761)/26-282                    25.6%     ------PR--V-----------VPD-----------AARMLCRLILRLEHGGD----L---IRG----------------      10 Agambiae(XP_319707.4-AGAP008954-PA)/1-254                25.0%     ------PG--L----------VVPS-----------AALMIARIVRRLLKGGD----T---HKG----------------      11 cintestinalis(XP_002128556.1)/14-266                     32.4%     ------PK--R-----------PPD-----------FVILLGRLLWNMQQYSS----A---KL-----------------      12 Lgigantea(LOTGIDRAFT_177746)/1-216                       26.2%     ------PI--I-----------PTD-----------SVRLFLRIIIRHMEWQM----I---D------------------      13 Drerio(E7EZZ6-SMYD3)/16-267                              34.7%     ------PR--I-----------PTD-----------SVRLVARIIFKLLSQSE----S---DQ-----------------      14 Xtropicalis(XP_004914684.1|-SMYD3-Predicted)/15-264      31.0%     ------PN--V-----------PPN-----------SVRLVGKIIFKMLQKPD----T---AS-----------------      15 Hsapiens(Q9H7B4-SMYD3)/15-266                            33.9%     ------PR--Y-----------PPD-----------SVRLLGRVVFKLMDGAP----S---ES-----------------      16 Ggallus(XP_419536.1-SMYD3-Predicted)/15-266              33.2%     ------PN--F-----------PPD-----------SVRLAGRIVFKLLRQSA----C---LS-----------------      17 Drerio(Q5RGL7-Smyd2b)/19-268                             30.9%     ------EN--W----------RPSE-----------TVRLVARIIARLKAQKE----R---SP-----------------      18 Drerio(Q5BJI7-Smyd2a)/18-267                             32.0%     ------EN--W----------CPSE-----------TVRLVARIILKQKHQTE----R---TP-----------------      19 Xtropicalis(XP_002934751.2-SMYD2-like-Predicted)/16-265  30.9%     ------QN--W----------CPSE-----------TVRLTARILAKQKTQTE----R---TA-----------------      20 Hsapiens(Q9NRG4-SMYD2)/18-267                            32.0%     ------EN--W----------NPSE-----------TVRLTARILAKQKIHPE----R---TP-----------------      21 Ggallus(XP_419420.1-SMYD2-Predicted)/21-270              31.6%     ------QN--W----------NPSE-----------TVRLTARILAKQKIHPE----R---TQ-----------------      22 Tadhaerens(XP_002109888.1)/20-262                        28.4%     ------PK-------------VPPD-----------SIRLLGRVAYTILQGQD---------------------------      23 Hmagnipapillata(XP_002163555.2)/16-259                   29.4%     ------PK-------------QPPD-----------FCRLVSQLIFNFYYNKK---------------------------      24 Nvectensis(XP_001627600.1)/17-253                        32.1%     ------PR-------------VPTD-----------LVLLMFRVWQLKSQ------------------------------      25 Bfloridae(XP_002594889.1-BRAFLDRAFT_124463)/14-258       38.4%     ------PE-------------TPTD-----------SIRLIARIINKTKTDSP----G---VP-----------------      26 Skowalevskii(XP_006817727.1)/14-260                      33.2%     ------PR-------------IPPG-----------SVRLMSRILYKLKDKSC----E---SQ-----------------      27 Amellifera(XP_006565332.1)/43-285                        18.4%     W------C--------------L-N---------LLLAMIPIRGLFMTKMQ-----------------------------      28 Dmelanogaster(msta-CG33548)/66-313                       17.7%     E------QEQ-----------VNPM---------SLRILTAVRVFHLGKEQ-----------------------------      29 Dmelanogaster(CG12119)/34-280                            19.0%     A---------------------NSV---------IIRLLCVARAINLSKEQ-----------------------------      30 Amellifera(XP_006565301.1)/26-284                        23.2%     --------IE-----------NTDV---------LAGVLFVLRLWLLKQKD-----------------------------      31 Dmelanogaster(CG9642)/21-271                             18.8%     -------ELL-----------QKKE---------HTEIQPVLKCLLLREHEE----------------------------      32 Dmelanogaster(CG9640)/17-268                             16.9%     -------DIL-----------VK-----------NFGICGLLKLLLLLENPR----------------------------      33 Amellifera(NP_001229486.1-LOC724300)/57-301              21.5%     ----------------------IIP---------RCDVLLVIRMLILWCKK-----------------------------      34 Dmelanogaster(CG14590-NP_610202.3)/55-322                18.9%     DARS--LNDY--------------F---------RGDALLVLKCLLLQRQS-----------------------------      35 Dmelanogaster(CG43129)/21-279                            17.1%     ------PSGS-----------RHCP---------QLDCIMPLRVLLAKEAN-----------------------------      36 Dmelanogaster(G11160)/58-319                             20.8%     LTER--AGPA-----------EVRD---------LYELVMIVRILLLRQHD-----------------------------      37 Amellifera(XP_624539.3-msta-like-Predicted)/54-297       21.5%     ------TEFG-----------YPHP---------SYQCINVIRALSLKDTN-----------------------------      38 Dmelanogaster(CG8503-NP_610944.1)/52-301                 20.0%     ------QEFG-----------GPHP---------LYTCLSTVRCLLIGETS-----------------------------      39 Agambiae(XP_309979.4-AGAP011530-PA)/50-300               22.8%     ------QHFY-----------VPHP---------TYQCLMPVRCLLLAESD-----------------------------      40 Dpulex(DAPPUDRAFT_120473)/58-292                         19.1%     N--------------------------------------------DVHRIH-----------------------------      41 Dpulex(DAPPUDRAFT_194440-Predicted)/53-302               19.6%     ---P--IVGS-----------LPVQ---------AYQSVMVLRCLALRDQN-----------------------------      42 Dpulex(DAPPUDRAFT_2393)/50-297                           20.2%     ------SQGA-----------INSQ---------VFQCITPLRYLTLPDSD-----------------------------      43 Dmelanogaster(CG18136-NP_649084.1)/58-318                22.6%     NYVP--GEEE-----------RKES---------AYCVIMLLRCMHLKDKD-----------------------------      44 Agambiae(XP_309220.5-AGAP001025-PA)/55-318               20.7%     RPNP--SNPE-----------QRES---------AYCVIVPLRVLLLERIA-----------------------------      45 Scerevisiae(P38890.1-SET5)/124-429                       16.1%     LHAGNWKRFV-----------NYCEKYCFTA---AFSVGLIYGSMLLDTTGE----------------------------      46 Athaliana(Q9ZUM9-ASHR2)/22-296                           14.0%     LHQSSSSAFS-----------DQPSDRQVQA---RFLL---SAYNLA---AA----------------------------      47 Athaliana(Q9FG08.2-ATXR4)/53-321                         16.2%     DW----SSFD-----------DYCRTHNFKY---PLMVKRLCCMIIS---GA----------------------------      48 Lgigantea(LOTGIDRAFT_232186)/323-670                     15.0%     ELYDLLDNEGWGIRDDGTKGEIWGGHYSL--------MILAN-IWASIIMEAK----R--LMI-----TDGA---TTA--      49 Bfloridae(XP_002589246.1-BRAFLDRAFT_74594)/380-720       14.2%     KLYDFCAK------GTTQEKGMWNSMFSP--------MIMAR-IWANILTRVK----E--LGV-----KG------EP--      50 Athaliana(Q5PP37-ATXR2)/52-466                           14.1%     ALGEFIKHA--------------NDTNDI--------FLLAAKAIAFTILRYR----K--LKAEHVDKKAKQ---SEP--      51 Cowczarzaki(EPH53581.1)/160-496                          15.7%     ELYALCRKHN---------------RTNP--------LIIAR-AFAMSLTGVT----S--GRF-----------------      52 Mbrevicollis(MONBRDRAFT_29283)/14-364                    16.5%     TLNEAWRDMHPP-----------PESTSI--------TLLLK-LVILEHN------------------------------      53 Cowczarzaki(EFW42079.2)/57-422                           15.4%     LLQSAWKSIHYP-----------PETTSI--------MLLAR-IIAMLRQGLD----K--NS------------------      54 Tadhaerens(XP_002114620.1)/25-373                        17.6%     RLRETWRNIHYP-----------PETSSI--------MLIAK-MIAMIEQ--A----D--DP------------------      55 Bfloridae(XP_002609030.1-BRAFLDRAFT_84846)/1-276         11.7%     ACDHCMRSM---------------ETAEA--------M--SR-R--------L----A--NS------------------      56 cintestinalis(XP_002127168.1)/13-358                     17.5%     ILDETWRGCHYP-----------PETASI--------QMIIR-ILARIKQ--E----E--KK------------------      57 Dpulex(EFX89935.1)/23-367                                16.0%     KLNEAWKKMHYP-----------PETSTI--------MLLVR-ILANFIQ--R----T--DR------------------      58 Dmelanogaster(CG3353-NP_650955.1)/13-363                 15.5%     VLNETWKKMHYP-----------PETGSI--------MLIVR-LMALYQQ--S----T--KK------------------      59 Hmagnipapillata(XP_002163562.2)/21-371                   16.0%     RIEEAWKQLHYP-----------PETASV--------MLIVR-ILAMIAQ--S----Q--CP------------------      60 Agambiae(XP_313299.1-AGAP003552-PA)/13-365               16.8%     ALVEFWKKMHYP-----------PETCGI--------MLFVK-IVGMFRQ--A----A--DP------------------      61 Amellifera(XP_394075.2-SMYD5-like-Prediction)/16-364     16.1%     QLNETWKQMHYP-----------PETASI--------MLLVK-MVALVNQ--A----N--NK------------------      62 Nvectensis(XP_001627062.1)/18-370                        15.2%     KLQETWKNIHYP-----------PETANI--------MLIAR-IMATILQ--A----T--NS------------------      63 Skowalevskii(XP_002735533.1)/24-372                      13.9%     KLQDIWRNMHFP-----------PETASI--------MLIAK-MIAKIKQ--A----P--DK------------------      64 Lgigantea(LOTGIDRAFT_231752)/19-367                      16.4%     RLQEIWRNIHYP-----------PETASI--------MLICK-MIAMVKQ--A----E--DP------------------      65 Drerio(F1RET2-Smyd5)/32-380                              16.4%     KLQDAWRSVHFP-----------PETSSV--------MILAK-MVATIKQ--T----Q--DK------------------      66 Ggallus(NP_001012912.1-SMYD5)/39-387                     16.9%     KLQEAWRNMHYP-----------PETSSI--------MLMAR-MVATVKQ--A----K--DK------------------      67 Hsapiens(Q6GMV2-SMYD5)/33-381                            15.8%     KLQEAWRSIHYP-----------PETASI--------MLMAR-MVATVKQ--A----K--DK------------------      68 Xtropicalis(A9ULL8-SMyd5)/32-382                         16.9%     KLEEAWRNMHYP-----------PETASI--------MLMAR-MVGTIKQVQA----Q--DK------------------      69 Mbrevicollis(MONBRDRAFT_36878)/153-462                   16.1%     -GSEVTPLK--------------PSSLRQA---------LAARVASCLAQSDP----SCD---ATTLDTVRPR-------      70 Mbrevicollis(MONBRDRAFT_27776)/11-280                    20.3%     -LPENPQ----------------PPHIIDAAAMTVATL-----VALERRAKLE----DKE------SEQASP--------      71 Cowczarzaki(EFW45970.2)/35-344                           21.5%     -GLQPYT----------------KDGVLDDDPVAINEVNLAARIIDARMSQVA----G-S------SRTLPP--------      72 Dpulex(EFX73755.1)/45-306                                19.5%     -VKPNVP----------------TDSVRLIARLI-----LKLQVINGYILLLS----NFQ------QKSLNN--------      73 Bfloridae(XP_002593048.1-BRAFLDRAFT_74375)/6-196         17.6%     -----------------------------------------------------------E------QGALGP--------      74 Bfloridae(XP_002594298.1-BRAFLDRAFT_117670)/15-265       21.7%     -LMNL-P-----------------EHLRVMGRIL-----YKMHARKT------------D------MGALGP--------      75 Scerevisiae(Q12529.1)/23-365                             10.9%     ------------------------------------FPSMLKRYNYTS---------EQEEKLNSILISENV-----I-Q      76 Dmelanogaster(CG1868-NP_724802.1)/226-549                15.1%     -LRLLG------------------------------ISHLALRLLLTY---------I-PYIRPHLQEMTSA-----K--      77 Agambiae(XP_319721.4-AGAP008973-PA)/165-486              14.6%     -WYQIG------------------------------IAHLGLRCFLDG---------F-GTIAGEMAKATDA-----S--      78 Athaliana(NP_174606.2)/229-550                           12.6%     ------------------------------------FQKLPDDIV------------------EHIKGVTS------A-D      79 Cintestinalis(XP_002123001.1)/195-567                    14.8%     -LAIKC----------------------M-------ELRVAVRALLTG---------AYELGETPQHDTTHTCTSIAK-H      80 Drerio(Q08C84-Smyd4)/197-556                             15.4%     -LLAIG----------------------V-------LGHLALRVVLKA---------GQTEVQMGIKNTKDHVTTYKN-D      81 Xtropicalis(NP_001072288.1-SMYD4)/212-545                18.0%     -LLALG----------------------M-------FCHTALRAVLVA---------GCRLFSQSLEQTGSADATDKT-K      82 Hsapiens(Q8IYR2-SMYD4)/244-602                           16.3%     -LLTLG----------------------V-------FCHIALRLTLLV---------GFEDVRKIITKLCDKI--SNK-D      83 Ggallus(NP_001025886.1-SMYD4)/241-573                    15.3%     -LLTLG----------------------V-------FFHVALRTVLLA---------GFSEVSRLVEWS-RDD--SNK-D      84 Hmagnipapillata(XP_002160254.2/232-532                   15.5%     -MEKVG------------------------------IAHLSLRIVLVS---------DAKDLLRFLGSDLNKFTDSPT--      85 Dpulex(DAPPUDRAFT_312722-Pedicted)/241-525               17.9%     -LNSVG------------------------------IAHLGARIVLSH---------GLDSVLAFLKDTDKV--------      86 Amellifera(XP_006565387.1-SMYD4-like-Predicted)/278-571  17.3%     -WKEIG------------------------------IGHLALKVLLTC---------STITDKIKFNEMQN---------      87 Bfloridae(XP_002589088.1-BRAFLDRAFT_75068)/251-714       13.4%     -LQMVE----------------------T-------FAQLSLRLLLTA---------AARGEKHPSADMESP--ATAS--      88 Lgigantea(LOTGIDRAFT_169490)/248-638                     12.9%     -LHSVG------------------------------IAHLSLRTVLTA---------GLQFLTDFIKERKDD--ESKK--      89 Skowalevskii(XP_002733823.1)/75-447                      16.7%     -LSQAG----------------------N-------FSQLSLRILLKA---------GWSNIQKYSKEVSNP--------      90 Nvectensis(XP_001627273.1)/170-547                       14.3%     -LEIVD----------------------T-------FTHLSLRILLTT---------SAKDIIDVLNGLSRD--V--A--      91 Amellifera(XP_003250668.1-SMYD4-like-Predicted)/183-473  15.8%     -DNKSS----------------------F-------VLKLLLKITDNG--------ARLKEALEYHKELENMSEEMEK--      92 Cowczarzaki(XP_004349923.1)/103-371                      17.2%     -LGTDAIHP--------------NDVISD-------LLRQAIRILSIR---------A--KRLQLLPLP-----------      93 Amellifera(XP_001121272.2-SMYD4-like-Predicted)/230-549  19.0%     -FFENDSEQ--------------IQRNTS-------YLLLAYRMIISGFLSST---EQIKNIEKKKISFL-----NNNFL      94 Amellifera(XP_003249162.1-SMYD4-like-Predicted)/239-589  15.1%     -LIGSG--------------------MSV-------LSMLALRMATQV---------GPAGCLRIHRALNRQDSAADGEE      95 Dmelanogaster(CG14122-NP_648574.1)/265-541               16.9%     -MIGSG--------------------MSI-------LCFIALRIFTQA---------PSLEQG-----------------      96 Agambiae(XP_311885.3-AGAP002999-PA)/268-544              17.3%     -MIGSG--------------------MSI-------LCHVALRMVTQA---------GTPEKV-----------------      97 Amellifera(XP_392262.3-SMYD4-like-Predicted)/252-555     20.2%     -LWASK--------------------TSI-------NCFLALRIIVQQ---------SFEKLYKLKDVKENSKDKF----      98 Dmelanogaster(CG7759-NP_725048.1)/250-537                16.4%     -IWRSG--------------------ASI-------NNHIALRIIASK---------PLDYFLKLKPTIDEE---L----      99 Agambiae(XP_319583.4-AGAP008839-PA)/240-523              16.0%     -LWGSG--------------------ASI-------TCHMALRMITQK---------SEEYFLKLKPELA-G---L----     100 Dpulex(DAPPUDRAFT_68494-Predicted)/254-551               17.2%     -LYAAG--------------------ISI-------ICYLSLRMIAIH---------PPSFFMDVRPVIEQPELQ-----     101 Dpulex(DAPPUDRAFT_309882)/300-599                        16.7%     -MIASG--------------------SSI-------LSWIALRILTKG---------KMEDFLEAREELEKDGDGG----     102 Dmelanogaster(CG8378-NP_610730.1)/196-491                17.4%     -LNRMFNKI----------------------------HCIALRTTLVALNIFPS-IEELIDFCEQEQNQD-----KCAFD     103 Agambiae(XP_566179.1-AGAP000216-PA)/158-458              17.1%     -LTETTRDP---------------------------VVLLAWRAVTRAISTYRYNLRHLKQRRNYLSRTE-----VNPLM     104 Agambiae(XP_564258.1-AGAP011234-PA)/216-546              17.1%     -CWRSVGFP-------------------V-------EMLLGLRTVATAFASFDQSLGQWIYRMETLDETK-----VNAFT     105 Agambiae(XP_309407.4-AGAP011238-PA)/219-497              17.1%     -CWRIVGHL-------------------F-------GGMVGLRTVATAIASFDQDLEGWNDHLNTLDETN-----VNAFT     106 Agambiae(XP_314169.4-AGAP005253-PB)/218-514              16.6%     -CWRIAGRL-------------------V-------GGIVGLRMVATAIASFEQDLEGWTNHLNALDETK-----VNAFT     107 Agambiae(XP_309409.4-AGAP011237-PA)/206-481              16.3%     -CWRMIGSL-------------------P-------GGIMGLRTVATAFASFEQDLEGWIDHLNTLDEAK-----VNAFT     108 Agambiae(XP_307865.2-AGAP009448-PA)/166-466              16.5%     -LWRVAGKC----------------------------PMTAVRTVASAFGTFDDDPDALQAHLDALDEPQ-----VNGFT     109 Agambiae(XP_309762.4-AGAP010931-PA)/113-383              17.2%     -LWTVLGIS----------------------------GVIALRMIAIAITTFDNDLEKLKDHLDALDESK-----VDGFT     110 Agambiae(XP_309378.2-AGAP011267-PA)/149-447              17.9%     -MVEVFDEL----------------------------PLIAIRMIAIAITTFDNNPEALKDHLDVLDESN-----VNGFT     111 Agambiae(XP_309383.4-AGAP011257-PA)/149-447              18.5%     -LYEDFEEV----------------------------SLIDIRMIAIAITTFDNNPEALKDHLDALDESN-----VNGFT     112 Agambiae(XP_307655.3-AGAP012638-PA)/149-447              18.5%     -MWEVFEEV----------------------------SLIDIRMIAIAITTFDNNPEALKDHLDALDESN-----VNGFT     113 Agambiae(XP_320681.4-AGAP011835-PA)/183-484              16.8%     -MWRIFTKL----------------------------PVMSLRTVTTAISAFEYDLQEMWEHLQVLEKAK-----VNAFT     114 Agambiae(XP_309411.4-AGAP011232-PA)/162-434              16.4%     -MRTIGTEY----------------------------LALAVRTVAIALASFDHDLEALRAHLSHLDVSK-----VNAFE     115 Amellifera(XP_001120776.2-SMYD4-like-Predicted)/251-554  19.0%     -IGNLL----------------------H-------VDKDKIRMLTKIIRFLIIATAKGKNINELRADMKLAESNPDNRT     116 Dpulex(DAPPUDRAFT_305694-Predicted)/258-553              19.7%     -LH----GT--------------N--VGR-------HGLLAVRTVLKV---------GRQRIMDVASEDD----GCN---     117 Dpulex(EFX87901.1)/258-554                               19.0%     -LCRTTRDV--------------N--TGQ-------HGLLALRTVLKA---------DRRLIIIANEQEK----SPE---     118 Amellifera(XP_001122116.2-SMYD4-like-Predicted)/234-534  17.2%     -ML----KM--------------N--FVK-------LDLFSLRLAIQA-------VREATSIQELRKELEEVDSCEDPRT     119 Hmagnipapillata(XP_002159692.1)/239-485                  18.1%     -LI----ND--------------D--V-G-------LAHLAFKIITNV---------GISMLLSFKENNS----------     120 Nvectensis(XP_001623892.1)/215-512                       18.8%     -VY----VA-----------------G-K-------YGHLALRVVVKA---------GFQYLKASVKQFESEEKKCDPAE     121 Lgigantea(LOTGIDRAFT_143433)/100-395                     19.3%     -IH----NI--------------D--F-G-------LGHLALRMVLKA---------GLNHILQNNKKYP-ESFRSDILR     122 Skowalevskii(XP_002740933.1)/253-549                     20.1%     -IQ----EL--------------G--L-G-------MGHLALRTIIRT---------GLAFLLKFREQSA-NVNIPDESF         consensus/100%                                                     ................................................................................         consensus/90%                                                      ............         ........     .....h.h.hh...................................         consensus/80%                                                      ...........          ........       .hhhhhthhhhh.....    ...............   ... .         consensus/70%                                                       ........             ... ...       .hhlsh+hlhhh.....    .............     . .                                                                           321          .         .         :         .         .         .         .         4 400   1 cintestinalis(NP_001071820.1)/15-282                    100.0%     ---------------------------------------------------KDGKENDEKKVELVK-IEELE-D---H--       2 Drerio(Q6P0R5-Smyd1a)/18-279                             35.9%     ------------------------------------------------------------DSQLTT-LDMLE-D---H--       3 Derio(Q2MJQ9-Smyd1b)/13-274                              34.5%     ------------------------------------------------------------DNQLTT-LEDLE-D---H--       4 Xtropicalis(NP_001120357.1-SMYD1)/13-261                 38.9%     ------------------------------------------------------------EGCLVS-IDDLQ-N---H--       5 Hsapiens(Q8NB12-SMYD1)/18-279                            38.4%     ------------------------------------------------------------EGCLVS-VDDLQ-N---H--       6 Ggallus(NP_989486.1-SMYD1)/13-274                        39.1%     ------------------------------------------------------------ENCLVS-IDDLQ-N---H--       7 Athaliana(Q7XJS0-ASHR1)/22-274                           25.8%     ---------------------------------------------------------ITTTDNYSL-VEALV-S---H--       8 Amellifera(XP_625013.1-SMYD3-Predicted)/1-253            27.1%     ---------------------------------------------------------YYSKTKYRK-FKDLM-S---H--       9 Dmelanogaster(Buzidau-CG13761)/26-282                    25.6%     ---------------------------------------------------------YYTEHGSRK-FRDLM-S---H--      10 Agambiae(XP_319707.4-AGAP008954-PA)/1-254                25.0%     ---------------------------------------------------------YYTSKQYRK-FCDLM-P---H--      11 cintestinalis(XP_002128556.1)/14-266                     32.4%     -------------------------------------------------------------PEKNS-ILDLE-S---N--      12 Lgigantea(LOTGIDRAFT_177746)/1-216                       26.2%     --------------------------------------------------------------------------------      13 Drerio(E7EZZ6-SMYD3)/16-267                              34.7%     -------------------------------------------------------------EELYS-IAEHQ-S---H--      14 Xtropicalis(XP_004914684.1|-SMYD3-Predicted)/15-264      31.0%     -------------------------------------------------------------EELYT-ISDLQ-S---H--      15 Hsapiens(Q9H7B4-SMYD3)/15-266                            33.9%     -------------------------------------------------------------EKLYS-FYDLE-S---N--      16 Ggallus(XP_419536.1-SMYD3-Predicted)/15-266              33.2%     -------------------------------------------------------------ERLYS-FKDLQ-S---N--      17 Drerio(Q5RGL7-Smyd2b)/19-268                             30.9%     ------------------------------------------------------------SEILLL-LGEME-A---H--      18 Drerio(Q5BJI7-Smyd2a)/18-267                             32.0%     ------------------------------------------------------------SERVLT-LRELE-A---H--      19 Xtropicalis(XP_002934751.2-SMYD2-like-Predicted)/16-265  30.9%     ------------------------------------------------------------SERFLS-VKDFE-S---H--      20 Hsapiens(Q9NRG4-SMYD2)/18-267                            32.0%     ------------------------------------------------------------SEKLLA-VKEFE-S---H--      21 Ggallus(XP_419420.1-SMYD2-Predicted)/21-270              31.6%     ------------------------------------------------------------SEKLLA-VKEFE-S---H--      22 Tadhaerens(XP_002109888.1)/20-262                        28.4%     --------------------------------------------------------------RADQ-FKFLL-S---N--      23 Hmagnipapillata(XP_002163555.2)/16-259                   29.4%     ---------------------------------------------------------------NTL-INNLY-A---N--      24 Nvectensis(XP_001627600.1)/17-253                        32.1%     ---------------------------------------------------------------NGW-YDSLV-S---N--      25 Bfloridae(XP_002594889.1-BRAFLDRAFT_124463)/14-258       38.4%     ---------------------------------------------------------------GNS-IDELQ-S---N--      26 Skowalevskii(XP_006817727.1)/14-260                      33.2%     --------------------------------------------------------------TITE-FMSLQ-S---H--      27 Amellifera(XP_006565332.1)/43-285                        18.4%     -----------------------------------------------------------R---K-C-LATLQ-Y---N--      28 Dmelanogaster(msta-CG33548)/66-313                       17.7%     -----------------------------------------------------------R---H-L-VDAMQ-A---N--      29 Dmelanogaster(CG12119)/34-280                            19.0%     -----------------------------------------------------------R---D-L-IYCLQ-A---N--      30 Amellifera(XP_006565301.1)/26-284                        23.2%     -----------------------------------------------------------PELWK-R-VLSLE-S---H--      31 Dmelanogaster(CG9642)/21-271                             18.8%     -------------------------------------------T---------------LPLYE-E-MSQMD-S---Q--      32 Dmelanogaster(CG9640)/17-268                             16.9%     -------------------------------------------T---------------KGDCQ-M-LIDVPIN---L--      33 Amellifera(NP_001229486.1-LOC724300)/57-301              21.5%     -----------------------------------------------------------SKYWN-S-IQKLQ-S---H--      34 Dmelanogaster(CG14590-NP_610202.3)/55-322                18.9%     -----------------------------------------------------------PTKWS-A-LLEMQ-S---H--      35 Dmelanogaster(CG43129)/21-279                            17.1%     -----------------------------------------------------------PERWD-NEVAPME-H---H--      36 Dmelanogaster(G11160)/58-319                             20.8%     -----------------------------------------------------------PEQFA-L-IARME-S---H--      37 Amellifera(XP_624539.3-msta-like-Predicted)/54-297       21.5%     -----------------------------------------------------------PESYK-K-LISLE-S---H--      38 Dmelanogaster(CG8503-NP_610944.1)/52-301                 20.0%     -----------------------------------------------------------TEKAS-K-FQDLE-S---L--      39 Agambiae(XP_309979.4-AGAP011530-PA)/50-300               22.8%     -----------------------------------------------------------PARWE-T-LLKLE-S---H--      40 Dpulex(DAPPUDRAFT_120473)/58-292                         19.1%     -----------------------------------------------------------P-----E-YKMLE-N---H--      41 Dpulex(DAPPUDRAFT_194440-Predicted)/53-302               19.6%     -----------------------------------------------------------PERWD-E-LMQLE-A---H--      42 Dpulex(DAPPUDRAFT_2393)/50-297                           20.2%     -----------------------------------------------------------RERL-----DELV-S---H--      43 Dmelanogaster(CG18136-NP_649084.1)/58-318                22.6%     -----------------------------------------------------------PDAFL-K-LYNLE-D---H--      44 Agambiae(XP_309220.5-AGAP001025-PA)/55-318               20.7%     -----------------------------------------------------------PERYA-T-VQGFE-S---H--      45 Scerevisiae(P38890.1-SET5)/124-429                       16.1%     -------------------------------------------V---------------KEQWQ-K-LASISQ-------      46 Athaliana(Q9ZUM9-ASHR2)/22-296                           14.0%     -------------------------------------------S---------------PSDFQ-I-LLSLQGS---G--      47 Athaliana(Q9FG08.2-ATXR4)/53-321                         16.2%     -------------------------------------------R---------------PADCL-D-ILQPAVL---S--      48 Lgigantea(LOTGIDRAFT_232186)/323-670                     15.0%     -------------------------------------------TVTHWAKAK----APY-RRFIAY-GTT----------      49 Bfloridae(XP_002589246.1-BRAFLDRAFT_74594)/380-720       14.2%     -------------------------------------------TKDQWARAK----EPY-RRFLGF-GVS----------      50 Athaliana(Q5PP37-ATXR2)/52-466                           14.1%     -------------------------------------------KQSLLLEAWKPVSIGYKRRWWDC-IALP---------      51 Cowczarzaki(EPH53581.1)/160-496                          15.7%     ---------------------------------------------SNMQDTF--------QRFGNF-IASE---------      52 Mbrevicollis(MONBRDRAFT_29283)/14-364                    16.5%     -----------------------------------------------QPGIL--------SNFCHD-FAREI-N------      53 Cowczarzaki(EFW42079.2)/57-422                           15.4%     ---------------------------------------------KDAFTAF--------QQFYRC-YADA---------      54 Tadhaerens(XP_002114620.1)/25-373                        17.6%     ---------------------------------------------MDVLKLF--------SQFSRV-TANN---------      55 Bfloridae(XP_002609030.1-BRAFLDRAFT_84846)/1-276         11.7%     ---------------------------------------------HSLVLPF--------PQCCAV-KL-----------      56 cintestinalis(XP_002127168.1)/13-358                     17.5%     ---------------------------------------------EEFISDI--------EKFCHA-STND---------      57 Dpulex(EFX89935.1)/23-367                                16.0%     ---------------------------------------------EELKSQL--------MSLCHH-TVNE---------      58 Dmelanogaster(CG3353-NP_650955.1)/13-363                 15.5%     ---------------------------------------------EEFLEQL--------QSFQSL-IVNR---------      59 Hmagnipapillata(XP_002163562.2)/21-371                   16.0%     ---------------------------------------------EMVMSEF--------QQFFSK-TKNE---------      60 Agambiae(XP_313299.1-AGAP003552-PA)/13-365               16.8%     ---------------------------------------------QALQAEL--------QDFVHK-SVNE---------      61 Amellifera(XP_394075.2-SMYD5-like-Prediction)/16-364     16.1%     ---------------------------------------------EDILSTF--------SQFCHR-TVND---------      62 Nvectensis(XP_001627062.1)/18-370                        15.2%     ---------------------------------------------DVKKGSF--------SHFCSN-VVNK---------      63 Skowalevskii(XP_002735533.1)/24-372                      13.9%     ---------------------------------------------SEAVAAF--------SQFRRA-TVNE---------      64 Lgigantea(LOTGIDRAFT_231752)/19-367                      16.4%     ---------------------------------------------GHVITIF--------NKFVNN-TVNE---------      65 Drerio(F1RET2-Smyd5)/32-380                              16.4%     ---------------------------------------------ERWQRLF--------TNFCSR-TANE---------      66 Ggallus(NP_001012912.1-SMYD5)/39-387                     16.9%     ---------------------------------------------DWWIKAF--------SQFCSK-TANE---------      67 Hsapiens(Q6GMV2-SMYD5)/33-381                            15.8%     ---------------------------------------------DRWIRLF--------SQFCNK-TANE---------      68 Xtropicalis(A9ULL8-SMyd5)/32-382                         16.9%     ---------------------------------------------DWWMHLF--------SQFCNK-TANE---------      69 Mbrevicollis(MONBRDRAFT_36878)/153-462                   16.1%     ---------------------------------------------SDPDP-T-GILGLQH-------SSNQT-E---G--      70 Mbrevicollis(MONBRDRAFT_27776)/11-280                    20.3%     ----------------------------------------------DP-----GS-AVRQPRCA-D-FWAMA-Q---H--      71 Cowczarzaki(EFW45970.2)/35-344                           21.5%     ----------------------------------------------PPPP-S-QD-DLESPTFE-D-VALML-S---N--      72 Dpulex(EFX73755.1)/45-306                                19.5%     -------------------------------------------------------------------YREEV-I---Y--      73 Bfloridae(XP_002593048.1-BRAFLDRAFT_74375)/6-196         17.6%     -------------------------------------------------------------------LCDLC-P---H--      74 Bfloridae(XP_002594298.1-BRAFLDRAFT_117670)/15-265       21.7%     -------------------------------------------------------------------LSSLV-S---N--      75 Scerevisiae(Q12529.1)/23-365                             10.9%     SSWDE-I-------------------------------------ESKWIP-RINN---------------MK-S-AKR--      76 Dmelanogaster(CG1868-NP_724802.1)/226-549                15.1%     GMWEE-I---------------------------------------MNLS-RKPEESENAPEYL-R-SLRMV-S---Q--      77 Agambiae(XP_319721.4-AGAP008973-PA)/165-486              14.6%     VCYQR-V---------------------------------------LEAT-R--EEDNPFSHYG-R-VLRLV-T---N--      78 Athaliana(NP_174606.2)/229-550                           12.6%     IYY--------------------------------------------------------------F-ATDLI-QEHQH--      79 Cintestinalis(XP_002123001.1)/195-567                    14.8%     IY------------------QSR---------------NS-CADCSEPVG-TDPKDGVYKCDYW-S-IFCLK-TSSCV--      80 Drerio(Q08C84-Smyd4)/197-556                             15.4%     SPV--------------------Q-------LS-----LG-GDCGKSLD----HTDCFHGSSYM-G-IYSLL-P---H--      81 Xtropicalis(NP_001072288.1-SMYD4)/212-545                18.0%     VCN----------------------------------------------S-KSTYHEKYCSSYQ-S-VVNLL-P---H--      82 Hsapiens(Q8IYR2-SMYD4)/244-602                           16.3%     ICLPE-S-------N--NQVKTLN-------YGLGESEKN-GNIVETPIP-GCDINGKYENNYN-A-VFNLL-P---H--      83 Ggallus(NP_001025886.1-SMYD4)/241-573                    15.3%     LCNAE-A-------G--GEHPSEA--------------LD-TRAGRKVIP-GCNDNGQYQSSYQ-A-VFNLL-P---H--      84 Hmagnipapillata(XP_002160254.2/232-532                   15.5%     -------------------------------------------LPSSKIE-GCNDQGIYQGDYE-S-VYFLS-T---H--      85 Dpulex(DAPPUDRAFT_312722-Pedicted)/241-525               17.9%     ----------------------------------------------KKVP-GIDGP-YDTKSYQ-V-MFHLV-S---H--      86 Amellifera(XP_006565387.1-SMYD4-like-Predicted)/278-571  17.3%     ----------------------------------------------------------------------LI-T---N--      87 Bfloridae(XP_002589088.1-BRAFLDRAFT_75068)/251-714       13.4%     -------------------------------------------KPSDQAK-LCTDKVSPTSDGA-K-TVQID-SETGS--      88 Lgigantea(LOTGIDRAFT_169490)/248-638                     12.9%     -------------------------------------------TANSRLP-GLNERGKYERSYD-T-VYYLM-T---H--      89 Skowalevskii(XP_002733823.1)/75-447                      16.7%     -------------------------------------------VSSSHIP-GCDTRGNYKSDYN-S-IYSLI-T---H--      90 Nvectensis(XP_001627273.1)/170-547                       14.3%     -------------------------------------------TTSCSLP-GCTVSGSYPGDYG-S-VFSLV-T---N--      91 Amellifera(XP_003250668.1-SMYD4-like-Predicted)/183-473  15.8%     -------------------------------------------------K-VYNLKEMKENNLR-S-ILNLS-I---PMK      92 Cowczarzaki(XP_004349923.1)/103-371                      17.2%     ------------------------------------------------------GEPQLRNSYENI-AKNLA-Y---S--      93 Amellifera(XP_001121272.2-SMYD4-like-Predicted)/230-549  19.0%     QYY--------------------------------VTNIN-----KERSN-LGTNEIYSPYDYR-T-ILNLE-T---H--      94 Amellifera(XP_003249162.1-SMYD4-like-Predicted)/239-589  15.1%     IAESSTITTTAEKLSKSAKRRSRRKKLRDSRRAKGEETVEEERREIKGEE-GEDGEKMAENVDL-R-VYDLV-T---H--      95 Dmelanogaster(CG14122-NP_648574.1)/265-541               16.9%     ----------------------------------------------------------LATANL-L-FEHLC-S---H--      96 Agambiae(XP_311885.3-AGAP002999-PA)/268-544              17.3%     ----------------------------------------------------------LEEGKM-L-RDTFC-A---H--      97 Amellifera(XP_392262.3-SMYD4-like-Predicted)/252-555     20.2%     -------------------------------------------------E-VSASEPYRSNDFK-I-MFRLV-T---H--      98 Dmelanogaster(CG7759-NP_725048.1)/250-537                16.4%     -------------------------------------------------T-PEQLISLPKDDFR-R-VAQLE-R---H--      99 Agambiae(XP_319583.4-AGAP008839-PA)/240-523              16.0%     -------------------------------------------------T-NEQIDKLPVDDYR-K-VYKLV-T---H--     100 Dpulex(DAPPUDRAFT_68494-Predicted)/254-551               17.2%     -------------------------------------------------K-K-AALSEDVKKYI-K-TYHLV-T---H--     101 Dpulex(DAPPUDRAFT_309882)/300-599                        16.7%     -------------------------------------------------R-L-LASARNPDSYS-G-IYHLA-T---L--     102 Dmelanogaster(CG8378-NP_610730.1)/196-491                17.4%     ---------------------------------------------------LNYNELTPEEHYR-A-IHGLV-T---N--     103 Agambiae(XP_566179.1-AGAP000216-PA)/158-458              17.1%     ---------------------------------------------------LNWVDGQK-IAFS-A-VYILA-S---L--     104 Agambiae(XP_564258.1-AGAP011234-PA)/216-546              17.1%     ---------------------------------------------------VDWNKATDRDIYD-T-VHVLA-T---N--     105 Agambiae(XP_309407.4-AGAP011238-PA)/219-497              17.1%     ---------------------------------------------------MDWKNATVSDIYD-T-VHVLA-T---N--     106 Agambiae(XP_314169.4-AGAP005253-PB)/218-514              16.6%     ---------------------------------------------------VDWNKVTDSDIYD-T-VHVLA-T---N--     107 Agambiae(XP_309409.4-AGAP011237-PA)/206-481              16.3%     ---------------------------------------------------VDWNEITDSDMYD-T-VHVLA-T---N--     108 Agambiae(XP_307865.2-AGAP009448-PA)/166-466              16.5%     ---------------------------------------------------MDWRTATPKDVYS-T-VHVLS-T---N--     109 Agambiae(XP_309762.4-AGAP010931-PA)/113-383              17.2%     ---------------------------------------------------MDWKKATLQDVFN-T-VHVLC-T---N--     110 Agambiae(XP_309378.2-AGAP011267-PA)/149-447              17.9%     ---------------------------------------------------MDWNKATQQDIFN-T-VHVLT-T---N--     111 Agambiae(XP_309383.4-AGAP011257-PA)/149-447              18.5%     ---------------------------------------------------MDWNKATQQDIFN-T-VHVLT-T---N--     112 Agambiae(XP_307655.3-AGAP012638-PA)/149-447              18.5%     ---------------------------------------------------MDWNKATQQDIFN-T-VHVLT-T---N--     113 Agambiae(XP_320681.4-AGAP011835-PA)/183-484              16.8%     ---------------------------------------------------MDWTSASAKDIYD-T-VHVLE-T---N--     114 Agambiae(XP_309411.4-AGAP011232-PA)/162-434              16.4%     ---------------------------------------------------MDWRAASPRTVYE-T-VYSLA-T---N--     115 Amellifera(XP_001120776.2-SMYD4-like-Predicted)/251-554  19.0%     ------------------------------------------------AG-FTDEDILDSTSAR-S-ALSLA-T---N--     116 Dpulex(DAPPUDRAFT_305694-Predicted)/258-553              19.7%     ---------------------------------------------------PAGGELYDSTDYGRT-IHRLV-G---N--     117 Dpulex(EFX87901.1)/258-554                               19.0%     ---------------------------------------------------SFASQVFDSANYD-T-VHRLV-D---N--     118 Amellifera(XP_001122116.2-SMYD4-like-Predicted)/234-534  17.2%     ------------------------------------------------KG-FSKNGMFLSDKYR-S-LLGLI-T---N--     119 Hmagnipapillata(XP_002159692.1)/239-485                  18.1%     ---------------------------------------------------FDDLKPYSSTDYN-S-IFSLI-E---N--     120 Nvectensis(XP_001623892.1)/215-512                       18.8%     ------------------------------------------------LG-CNPDGVYDPSDYR-P-IYHLV-G---H--     121 Lgigantea(LOTGIDRAFT_143433)/100-395                     19.3%     ------------------------------------------------IG-FNKDGVYDSMDYD-T-VYSLV-K---H--     122 Skowalevskii(XP_002740933.1)/253-549                     20.1%     ------------------------------------------------HG-CTVDGEYES-NYY-S-VYNLV-G---H--         consensus/100%                                                     ................................................................................         consensus/90%                                                                                                 ....................... h.... .   .           consensus/80%                                                                                                   ..... ............... hhth. .   .           consensus/70%                                                                                                    .... .........tth..t hhph. s   p                                                                           401          .         .         .         .         :         .         .         . 480   1 cintestinalis(NP_001071820.1)/15-282                    100.0%     -LS-------KRNAE-EK---EAIDEKVYSF----GDYFT---YDE----------------------------------       2 Drerio(Q6P0R5-Smyd1a)/18-279                             35.9%     -LS-------RMTPE-DL---KELKADVKTF----YTYWP---KKS----------------------------------       3 Derio(Q2MJQ9-Smyd1b)/13-274                              34.5%     -IC-------DISED-DL---KDFKVDIHNF----LDYWP---RNS----------------------------------       4 Xtropicalis(NP_001120357.1-SMYD1)/13-261                 38.9%     -ID-------KFDEA-EK---GLLMEDVQKF----LEYWP---SQS----------------------------------       5 Hsapiens(Q8NB12-SMYD1)/18-279                            38.4%     -VE-------HFGEE-EQ---KDLRVDVDTF----LQYWP---PQS----------------------------------       6 Ggallus(NP_989486.1-SMYD1)/13-274                        39.1%     -VE-------SFDEE-EK---KDLRVDVESF----LEFWP---AQS----------------------------------       7 Athaliana(Q7XJS0-ASHR1)/22-274                           25.8%     -MS-------EIDEK-QM---LLYAQMANLV----NLI------------------------------------------       8 Amellifera(XP_625013.1-SMYD3-Predicted)/1-253            27.1%     -YS-------DIKKD-EKK-MEHFVCVCGVL----YEFLG---D------------------------------------       9 Dmelanogaster(Buzidau-CG13761)/26-282                    25.6%     -YA-------EIKND-PMR-LEHLDSLHAVL----TDMMA---E------------------------------------      10 Agambiae(XP_319707.4-AGAP008954-PA)/1-254                25.0%     -EE-------NIRAD-SKR-MEHFGTLYVVL----QRLLD---E------------------------------------      11 cintestinalis(XP_002128556.1)/14-266                     32.4%     -YN-------KLSQN-QK---EALMNFLVIL----HTFWS---P------------------------------------      12 Lgigantea(LOTGIDRAFT_177746)/1-216                       26.2%     -----------------------------MM----LSTVT---ELTK-------------------D-------------      13 Drerio(E7EZZ6-SMYD3)/16-267                              34.7%     -LA-------DMSEE-KK---EGLKHLCTTL----QVYLA---EENC-------------------D-------------      14 Xtropicalis(XP_004914684.1|-SMYD3-Predicted)/15-264      31.0%     -IK-------EASEE-VK---DGLRHLATAL----QHYLK---EEIQ-------------------E-------------      15 Hsapiens(Q9H7B4-SMYD3)/15-266                            33.9%     -IN-------KLTED-KK---EGLRQLVMTF----QHFMR---EEIQ-------------------D-------------      16 Ggallus(XP_419536.1-SMYD3-Predicted)/15-266              33.2%     -AE-------QLSEE-MK---EGLGHLAHTL----QLYLR---AEIQ-------------------D-------------      17 Drerio(Q5RGL7-Smyd2b)/19-268                             30.9%     -LE-------DMDNE-KR---EMTEAHIAGL----HQFYS---KHL----------------------------------      18 Drerio(Q5BJI7-Smyd2a)/18-267                             32.0%     -LD-------KLDNE-KN---EMNDTDIAAL----HHFYS---RHL----------------------------------      19 Xtropicalis(XP_002934751.2-SMYD2-like-Predicted)/16-265  30.9%     -LS-------KLDNE-KL---ELIQNDIAAL----HRFYS---KNL----------------------------------      20 Hsapiens(Q9NRG4-SMYD2)/18-267                            32.0%     -LD-------KLDNE-KK---DLIQSDIAAL----HHFYS---KHL----------------------------------      21 Ggallus(XP_419420.1-SMYD2-Predicted)/21-270              31.6%     -LD-------KLDNE-KR---ELIQNDIAAL----HHFYS---KHM----------------------------------      22 Tadhaerens(XP_002109888.1)/20-262                        28.4%     -RE-------LLEGS-RK---NTIVDGINLL----KEYLS---NKV----------------------------------      23 Hmagnipapillata(XP_002163555.2)/16-259                   29.4%     -KG-------NISNA-RK---EAFFTFAAVL----VEYLQ---DVN----------------------------------      24 Nvectensis(XP_001627600.1)/17-253                        32.1%     -VE-------KIDSD-AK---EDFVSVLMVL----NEYLG---SEI----------------------------------      25 Bfloridae(XP_002594889.1-BRAFLDRAFT_124463)/14-258       38.4%     -LR-------EMPEN-VK---EMFAQLAVVL----RMYVG---KDV----------------------------------      26 Skowalevskii(XP_006817727.1)/14-260                      33.2%     -DT-------ALTPE-KK---EQFSQLLFVL----NQYVD---EGT----------------------------------      27 Amellifera(XP_006565332.1)/43-285                        18.4%     -KN-------------LFS-KYEIELLKKNV----MNSPS---D------------------------------------      28 Dmelanogaster(msta-CG33548)/66-313                       17.7%     -AE-------------RAY-RREIIQAAQCF----RNFPT---T------------------------------------      29 Dmelanogaster(CG12119)/34-280                            19.0%     -LD-------------NNH-RTEVRNAAKCF----KNFPT---D------------------------------------      30 Amellifera(XP_006565301.1)/26-284                        23.2%     -LN-------KRRNT-IVW-EDREINIVNVL----KSLNF-------LEN------------------------------      31 Dmelanogaster(CG9642)/21-271                             18.8%     -LM-------TRRGT-EVW-KNYQEHAFTPL----DYGGV---LA-QLRG------------------------------      32 Dmelanogaster(CG9640)/17-268                             16.9%     -SD-------YRDGE-GMW-QEHEELVVRPL----MESGL---AD-VLPT------------------------------      33 Amellifera(NP_001229486.1-LOC724300)/57-301              21.5%     -ED-------SRGPGTSVY-EE-TMNIYYHI----QRLLP---DN-----------------------------------      34 Dmelanogaster(CG14590-NP_610202.3)/55-322                18.9%     -EE-------ERKGT-DLY-EEAEKRVVTYL----QKRFL---CR-LKQTNP----------------------------      35 Dmelanogaster(CG43129)/21-279                            17.1%     -KE-------ERQRDADVW-HADRVNIAQYL----RGPCQ---LA-----------------------------------      36 Dmelanogaster(G11160)/58-319                             20.8%     -TE-------ERRQNAVLW-RHYEEKVVQRL----RVTWQ---LE-----------------------------------      37 Amellifera(XP_624539.3-msta-like-Predicted)/54-297       21.5%     -CN-------EMNNS-K-----EPLNIAHFI----KRFFK---AD-----------------------------------      38 Dmelanogaster(CG8503-NP_610944.1)/52-301                 20.0%     -ES-------TRRGS-NQW-KADLVSIGQFI----PKFFK---TQ-----------------------------------      39 Agambiae(XP_309979.4-AGAP011530-PA)/50-300               22.8%     -EE-------ERRGS-EQW-RNDREGVAKLI----PRFFK---CE-----------------------------------      40 Dpulex(DAPPUDRAFT_120473)/58-292                         19.1%     -ND-------LRRQS-DMW-RIYQVNVVQFL----RKICG---LA-----------------------------------      41 Dpulex(DAPPUDRAFT_194440-Predicted)/53-302               19.6%     -VQ-------ERRQK-GME-DVDQATAVRFI----RETLG---LQ-----------------------------------      42 Dpulex(DAPPUDRAFT_2393)/50-297                           20.2%     -LE-------QRRGM-DIY-RLVEQNISSFL----RYRLL---LT-----------------------------------      43 Dmelanogaster(CG18136-NP_649084.1)/58-318                22.6%     -LK-------ERLET-PLY-QVLRANLITFI----KTVLG---MK-----------------------------------      44 Agambiae(XP_309220.5-AGAP001025-PA)/55-318               20.7%     -LD-------ERLAS-PLY-GVLRSNLVPFL----RQVLR---LQ-----------------------------------      45 Scerevisiae(P38890.1-SET5)/124-429                       16.1%     ------------RER---I-KLRDASGIGST----FSLLN---GTTVHTEEE-----------------SD---------      46 Athaliana(Q9ZUM9-ASHR2)/22-296                           14.0%     -SS-------NGDPS---C-SAGDSAAAGFL----HSLLS---SV-C-PSLP-----------------V----------      47 Athaliana(Q9FG08.2-ATXR4)/53-321                         16.2%     -S----------EMI---S---KIEDGYGLL----WNAFR---KANFKDDDV-----------------A----------      48 Lgigantea(LOTGIDRAFT_232186)/323-670                     15.0%     ----------------------SAISRMPHM----LPVFR----RVF--KNS----------------------------      49 Bfloridae(XP_002589246.1-BRAFLDRAFT_74594)/380-720       14.2%     ----------------------GFVKQIPKM----LKIMQ----AIF--QNT----------------------------      50 Athaliana(Q5PP37-ATXR2)/52-466                           14.1%     --D-------D--VD-PTD-EGAFRMQIKNLACTSLELLK---IAIF--DKE----------------------------      51 Cowczarzaki(EPH53581.1)/160-496                          15.7%     ----------EQTPH-D-------AEEFALI----LNAFS------P--PHR----------------------------      52 Mbrevicollis(MONBRDRAFT_29283)/14-364                    16.5%     --G-------EMVAH-RLL-APEFEASLSQL----NELVWAWAEECQ--LDN----------------------------      53 Cowczarzaki(EFW42079.2)/57-422                           15.4%     --E-------GHFIH-KFL-DKKYDKQLAFI----QELFK---TALY--DDR----------------------------      54 Tadhaerens(XP_002114620.1)/25-373                        17.6%     --E-------AHVAH-KLF-GKQFVEQIEIL----RLELI---NTLP--SSK----------------------------      55 Bfloridae(XP_002609030.1-BRAFLDRAFT_84846)/1-276         11.7%     -------------EQ-HVT-CPHCQDQLELL----RGLLT---EALY--EES----------------------------      56 cintestinalis(XP_002127168.1)/13-358                     17.5%     --V-------EQIAH-KLL-GEQFLVQLTTL----REQLA---SVFF--DES----------------------------      57 Dpulex(EFX89935.1)/23-367                                16.0%     --E-------ETIAH-KLL-GQEFESQLELL----RDLCT---KALG--MPE----------------------------      58 Dmelanogaster(CG3353-NP_650955.1)/13-363                 15.5%     --E-------QKIYH-KML-GENFEQQMEQL----YLAFC---NAFT--GEE----------------------------      59 Hmagnipapillata(XP_002163562.2)/21-371                   16.0%     --E-------HKITH-KLL-GNKFKEQIILM----HSLLQ---EIVP--TGE----------------------------      60 Agambiae(XP_313299.1-AGAP003552-PA)/13-365               16.8%     --D-------LLIFH-KML-GEKFTQQIEQL----YELFC---KAFQ--VES----------------------------      61 Amellifera(XP_394075.2-SMYD5-like-Prediction)/16-364     16.1%     --T-------HEIAH-KLL-GEKFVGQIDVL----RQMMQ---KTIN--IEF----------------------------      62 Nvectensis(XP_001627062.1)/18-370                        15.2%     --E-------QQIAH-KLL-GLHFQEQLDMI----RILLS---EAMY--DDR----------------------------      63 Skowalevskii(XP_002735533.1)/24-372                      13.9%     --E-------ADITH-KLL-GEEFQGDIDML----LPFLN---EALK--EET----------------------------      64 Lgigantea(LOTGIDRAFT_231752)/19-367                      16.4%     --E-------EQIAH-KLL-GDQFKCQLELL----RSTTA---EILF--DES----------------------------      65 Drerio(F1RET2-Smyd5)/32-380                              16.4%     --E-------EEIVH-KLL-GEKFQGQLGLL----RNLFT---TALY--EDR----------------------------      66 Ggallus(NP_001012912.1-SMYD5)/39-387                     16.9%     --E-------EEIAH-KLL-GDKFKGQLELL----RLLFT---EALY--DEQ----------------------------      67 Hsapiens(Q6GMV2-SMYD5)/33-381                            15.8%     --E-------EEIVH-KLL-GDKFKGQLELL----RRLFT---EALY--EEA----------------------------      68 Xtropicalis(A9ULL8-SMyd5)/32-382                         16.9%     --E-------EEIVH-KLL-GDKFKGQLDQL----RRLFT---DALY--EER----------------------------      69 Mbrevicollis(MONBRDRAFT_36878)/153-462                   16.1%     -DE-----DMKEDDD-DDDDGDKASAVAVH--------------Q-----------------------------------      70 Mbrevicollis(MONBRDRAFT_27776)/11-280                    20.3%     -TP-----TLN-----------SEELDDVLQ-------------------------------------------------      71 Cowczarzaki(EFW45970.2)/35-344                           21.5%     -AL-----PLARANA-KRY-ASNAELAALLA----TRY------S-----------------------------------      72 Dpulex(EFX73755.1)/45-306                                19.5%     -RK-----EIKEDT--KR----MEYFMTIC--------------------------------------------------      73 Bfloridae(XP_002593048.1-BRAFLDRAFT_74375)/6-196         17.6%     -TK-----ELKDSS----------ELRIQL--------------------------------------------------      74 Bfloridae(XP_002594298.1-BRAFLDRAFT_117670)/15-265       21.7%     -VE-----TLKNCE--EG----ITSLDSKM--------------------------------------------------      75 Scerevisiae(Q12529.1)/23-365                             10.9%     -IN-------QLPPT-CED---EYC-CIRFV----CESLF---NLKYMDPQCI---------------------------      76 Dmelanogaster(CG1868-NP_724802.1)/226-549                15.1%     -LD-------QAIDE-ELN---YHILCANLL----QLYLK---EHTDFYDQF-H---------------SL---------      77 Agambiae(XP_319721.4-AGAP008973-PA)/165-486              14.6%     -FD-------KMDPD-DRM---RYTLAGLML----TIYLQ---ECTPFAEAV-K---------------DY---------      78 Athaliana(NP_174606.2)/229-550                           12.6%     -ECRGANWPAVLPSD--------AVLAGRII----MKL-I---NQG---KAA-------------TDLSNLQEI------      79 Cintestinalis(XP_002123001.1)/195-567                    14.8%     -EKNS-----DFKND-CSW---FCMLVSTIR----SEVFG---EEE---NRA------------KDDVASLKEL------      80 Drerio(Q08C84-Smyd4)/197-556                             15.4%     -VA-------QHSPA-SRF---LMAITMAVI----YGKLQ---GGP-PPNK----------------------W------      81 Xtropicalis(NP_001072288.1-SMYD4)/212-545                18.0%     -TE-------NHPAE-RKF---LCGLTAAAL----YKKLC---LIM-AKDLV------------SST--SQTEK------      82 Hsapiens(Q8IYR2-SMYD4)/244-602                           16.3%     -TE-------NHSPE-HKF---LCALCVSAL----CRQLE---AAS-LQAIP------------TERIVN----------      83 Ggallus(NP_001025886.1-SMYD4)/241-573                    15.3%     -VE-------KHSPE-HKF---LCMLSIVAI----CKKLQ---ETG-LEAAV------------LNGESSTTGS------      84 Hmagnipapillata(XP_002160254.2/232-532                   15.5%     -SD-------RLPIE-DLF---QYSVAGFLL----YKLLI---NSS-FFKTHTVL-------------------------      85 Dpulex(DAPPUDRAFT_312722-Pedicted)/241-525               17.9%     -TE-------RMAPE-ELY---QYALTAAFL----TLLLE---QHSSFFQSA----------------------------      86 Amellifera(XP_006565387.1-SMYD4-like-Predicted)/278-571  17.3%     -FD-------KLSID-DLT---IYGITAIML----TIYLF---KYTNFFKTN--------------N-------------      87 Bfloridae(XP_002589088.1-BRAFLDRAFT_75068)/251-714       13.4%     -TS-------EQPGD--------LSVQTDVI----EENPP---SAG-MESPTTADKPSDQNKLCTDDLSSTSEEAKTDSE      88 Lgigantea(LOTGIDRAFT_169490)/248-638                     12.9%     -DN-------DILTE-DMY---QYSGTAALL----LIILV---HSG-WFNTNVTQI----ATHIDSTLQADLQSV-----      89 Skowalevskii(XP_002733823.1)/75-447                      16.7%     -SG-------KQPWK-DVF---FFTLTSILL----STLVT----KL-ISPSD----------DVD-DLLADTEAMKMTEA      90 Nvectensis(XP_001627273.1)/170-547                       14.3%     -SD-------LQPIK-ALM---SFAMNSAFL----VEFLE----NG-TSSAC---------IHCS-QIKSDKT--KVQTE      91 Amellifera(XP_003250668.1-SMYD4-like-Predicted)/183-473  15.8%     KDD-------NRTED-NLF---YSAKIAMLL----RNH------SNYMQGS-----------------------------      92 Cowczarzaki(XP_004349923.1)/103-371                      17.2%     -PT-------LADDN-DH----TLKFVVKFA----NELV-----------------------------------------      93 Amellifera(XP_001121272.2-SMYD4-like-Predicted)/230-549  19.0%     -CT-------KMEPK-TNL---IRAIEAIFL----AKCFT---FVLSKMDVVY---------------------------      94 Amellifera(XP_003249162.1-SMYD4-like-Predicted)/239-589  15.1%     -EK-------RRTAK-DFF---ERSLMAAFL----FKCLQ---KVGF-FDNPS------------------SN-------      95 Dmelanogaster(CG14122-NP_648574.1)/265-541               16.9%     -EE-------DRQPD-DYL---RRALMSGFL----LRILQ---KSLY-FGRRK------------------TE-------      96 Agambiae(XP_311885.3-AGAP002999-PA)/268-544              17.3%     -TE-------HRDPE-DHF---KRTLMTAFL----LRCLQ---KAEF-FGRRT------------------TE-------      97 Amellifera(XP_392262.3-SMYD4-like-Predicted)/252-555     20.2%     -ED-------TRTVE-DLF---HRTYIASWL----LRLLK---KGPY-FPKHVKT-----------P---DTI-------      98 Dmelanogaster(CG7759-NP_725048.1)/250-537                16.4%     -QG-------ERQPS-NFF---QHVLMARFL----TNCLR---AGGY-FGSEPK--------------------------      99 Agambiae(XP_319583.4-AGAP008839-PA)/240-523              16.0%     -ES-------TRSPE-DFF---QRTLMATLL----NACLT---LGGY-GA---C--------------------------     100 Dpulex(DAPPUDRAFT_68494-Predicted)/254-551               17.2%     -DT-------LRNKE-SFF---HVTLMANFL----LKCLK---VAGY-FGTRD------------------TT-------     101 Dpulex(DAPPUDRAFT_309882)/300-599                        16.7%     -SH-------LRSDK-DFF---DRTFMALFL----FQCLR---ASGY-LQTRFR------------Y---EED-------     102 Dmelanogaster(CG8378-NP_610730.1)/196-491                17.4%     -QH-------LRSVS-DLF---QRSVVCAVL----KHFII---EYTPV-KE-YL--------------------------     103 Agambiae(XP_566179.1-AGAP000216-PA)/158-458              17.1%     -AR-------APNDP-VEA---RVAQISREM----HCHLV---SENGQ-TANDD--------------------------     104 Agambiae(XP_564258.1-AGAP011234-PA)/216-546              17.1%     -QN-------RRDHK-QLA---SLIFFAYIV----QGLLL---DRTE-LRPLCW--------------------------     105 Agambiae(XP_309407.4-AGAP011238-PA)/219-497              17.1%     -QK-------RRSRK-DLA---ELIFFASIV----HRLLL---ERTD-FGPLCE--------------------------     106 Agambiae(XP_314169.4-AGAP005253-PB)/218-514              16.6%     -QK-------RRSRE-DLA---VLMFFTSIV----HRLLL---ERTD-LGPFCE--------------------------     107 Agambiae(XP_309409.4-AGAP011237-PA)/206-481              16.3%     -QK-------RRSCK-DLA---MLIFFASIV----HRLLL---ERTE-LGTLCE--------------------------     108 Agambiae(XP_307865.2-AGAP009448-PA)/166-466              16.5%     -QE-------RRPFM-QLV---FMVYLAIII----HKLML---ERTE-LGPRSR--------------------------     109 Agambiae(XP_309762.4-AGAP010931-PA)/113-383              17.2%     -QE-------RRNIK-ELA---GLTFFTVVM----HNHLL---EWTE-LGPACE--------------------------     110 Agambiae(XP_309378.2-AGAP011267-PA)/149-447              17.9%     -QE-------RRDSN-FLA---FHIFNATIL----HTLVL---ERTE-LGPVCE--------------------------     111 Agambiae(XP_309383.4-AGAP011257-PA)/149-447              18.5%     -QE-------RRHSM-FVA---MFIFNATIL----HTLIL---ERTE-LGPVCE--------------------------     112 Agambiae(XP_307655.3-AGAP012638-PA)/149-447              18.5%     -QE-------RRDSF-FVA---FYIFNATIL----HTLVL---ERTE-LGPVCE--------------------------     113 Agambiae(XP_320681.4-AGAP011835-PA)/183-484              16.8%     -ER-------TRDRK-DRM---VRVFYTTII----YRLLE---ERCPELGELCA--------------------------     114 Agambiae(XP_309411.4-AGAP011232-PA)/162-434              16.4%     -QR-------KRARK-DFA---LNVLVAMIT----HKLLL---KRTPA-AQVCG--------------------------     115 Amellifera(XP_001120776.2-SMYD4-like-Predicted)/251-554  19.0%     -MT-------MRPLI-GIS---AFACISALA----AILLA---TQTNFFCNKYEV-----------N---QLK-------     116 Dpulex(DAPPUDRAFT_305694-Predicted)/258-553              19.7%     -TA-------RRSVA-DLF---RRAVMAVYL----TSLIQ---QQRD---------------------------------     117 Dpulex(EFX87901.1)/258-554                               19.0%     -SS-------QRSTT-DIF---RRAVMAVYL----TSLIQ---IRD----------------------------------     118 Amellifera(XP_001122116.2-SMYD4-like-Predicted)/234-534  17.2%     -TE-------KRSVQ-DLF---RRSLDASFI----LYFLA---TCSNMFGNPLKK-----------D---LSV-------     119 Hmagnipapillata(XP_002159692.1)/239-485                  18.1%     --------------------------------------------------------------------------------     120 Nvectensis(XP_001623892.1)/215-512                       18.8%     -TH-------ERTLN-DLF---VRTLNAIYL----LRCLE---GT------EYYG-----------D---STK-------     121 Lgigantea(LOTGIDRAFT_143433)/100-395                     19.3%     -SE-------KRSLG-DLF---KRSVVAVFM----VKCLE---HT------LSSQ-----------P---LST-------     122 Skowalevskii(XP_002740933.1)/253-549                     20.1%     -SE-------DRKPG-DLF---KRVVKAVCL----LRCLQ---QT------NFFQ-----------S---VGA-------         consensus/100%                                                     ................................................................................         consensus/90%                                                       .t       ..... ... .......h..h    .....   ...........            .  ....                consensus/80%                                                       .p       t...t .h. ...h...h.hh    ..hh.   ...........                                   consensus/70%                                                       .p       ph..p ph. ...h...hthl    hphh.   tt.. ....                                                                                                     481          .         5         .         .         .         .         :         . 560   1 cintestinalis(NP_001071820.1)/15-282                    100.0%     --------------------------------------------------------------------------------       2 Drerio(Q6P0R5-Smyd1a)/18-279                             35.9%     --------------------------------------------------------------------------------       3 Derio(Q2MJQ9-Smyd1b)/13-274                              34.5%     --------------------------------------------------------------------------------       4 Xtropicalis(NP_001120357.1-SMYD1)/13-261                 38.9%     --------------------------------------------------------------------------------       5 Hsapiens(Q8NB12-SMYD1)/18-279                            38.4%     --------------------------------------------------------------------------------       6 Ggallus(NP_989486.1-SMYD1)/13-274                        39.1%     --------------------------------------------------------------------------------       7 Athaliana(Q7XJS0-ASHR1)/22-274                           25.8%     --------------------------------------------------------------------------------       8 Amellifera(XP_625013.1-SMYD3-Predicted)/1-253            27.1%     --------------------------------------------------------------------------------       9 Dmelanogaster(Buzidau-CG13761)/26-282                    25.6%     --------------------------------------------------------------------------------      10 Agambiae(XP_319707.4-AGAP008954-PA)/1-254                25.0%     --------------------------------------------------------------------------------      11 cintestinalis(XP_002128556.1)/14-266                     32.4%     --------------------------------------------------------------------------------      12 Lgigantea(LOTGIDRAFT_177746)/1-216                       26.2%     --------------------------------------------------------------------------------      13 Drerio(E7EZZ6-SMYD3)/16-267                              34.7%     --------------------------------------------------------------------------------      14 Xtropicalis(XP_004914684.1|-SMYD3-Predicted)/15-264      31.0%     --------------------------------------------------------------------------------      15 Hsapiens(Q9H7B4-SMYD3)/15-266                            33.9%     --------------------------------------------------------------------------------      16 Ggallus(XP_419536.1-SMYD3-Predicted)/15-266              33.2%     --------------------------------------------------------------------------------      17 Drerio(Q5RGL7-Smyd2b)/19-268                             30.9%     --------------------------------------------------------------------------------      18 Drerio(Q5BJI7-Smyd2a)/18-267                             32.0%     --------------------------------------------------------------------------------      19 Xtropicalis(XP_002934751.2-SMYD2-like-Predicted)/16-265  30.9%     --------------------------------------------------------------------------------      20 Hsapiens(Q9NRG4-SMYD2)/18-267                            32.0%     --------------------------------------------------------------------------------      21 Ggallus(XP_419420.1-SMYD2-Predicted)/21-270              31.6%     --------------------------------------------------------------------------------      22 Tadhaerens(XP_002109888.1)/20-262                        28.4%     --------------------------------------------------------------------------------      23 Hmagnipapillata(XP_002163555.2)/16-259                   29.4%     --------------------------------------------------------------------------------      24 Nvectensis(XP_001627600.1)/17-253                        32.1%     --------------------------------------------------------------------------------      25 Bfloridae(XP_002594889.1-BRAFLDRAFT_124463)/14-258       38.4%     --------------------------------------------------------------------------------      26 Skowalevskii(XP_006817727.1)/14-260                      33.2%     --------------------------------------------------------------------------------      27 Amellifera(XP_006565332.1)/43-285                        18.4%     --------------------------------------------------------------------------------      28 Dmelanogaster(msta-CG33548)/66-313                       17.7%     --------------------------------------------------------------------------------      29 Dmelanogaster(CG12119)/34-280                            19.0%     --------------------------------------------------------------------------------      30 Amellifera(XP_006565301.1)/26-284                        23.2%     --------------------------------------------------------------------------------      31 Dmelanogaster(CG9642)/21-271                             18.8%     --------------------------------------------------------------------------------      32 Dmelanogaster(CG9640)/17-268                             16.9%     --------------------------------------------------------------------------------      33 Amellifera(NP_001229486.1-LOC724300)/57-301              21.5%     --------------------------------------------------------------------------------      34 Dmelanogaster(CG14590-NP_610202.3)/55-322                18.9%     --------------------------------------------------------------------------------      35 Dmelanogaster(CG43129)/21-279                            17.1%     --------------------------------------------------------------------------------      36 Dmelanogaster(G11160)/58-319                             20.8%     --------------------------------------------------------------------------------      37 Amellifera(XP_624539.3-msta-like-Predicted)/54-297       21.5%     --------------------------------------------------------------------------------      38 Dmelanogaster(CG8503-NP_610944.1)/52-301                 20.0%     --------------------------------------------------------------------------------      39 Agambiae(XP_309979.4-AGAP011530-PA)/50-300               22.8%     --------------------------------------------------------------------------------      40 Dpulex(DAPPUDRAFT_120473)/58-292                         19.1%     --------------------------------------------------------------------------------      41 Dpulex(DAPPUDRAFT_194440-Predicted)/53-302               19.6%     --------------------------------------------------------------------------------      42 Dpulex(DAPPUDRAFT_2393)/50-297                           20.2%     --------------------------------------------------------------------------------      43 Dmelanogaster(CG18136-NP_649084.1)/58-318                22.6%     --------------------------------------------------------------------------------      44 Agambiae(XP_309220.5-AGAP001025-PA)/55-318               20.7%     --------------------------------------------------------------------------------      45 Scerevisiae(P38890.1-SET5)/124-429                       16.1%     --------------------------------------------------------------------------------      46 Athaliana(Q9ZUM9-ASHR2)/22-296                           14.0%     --------------------------------------------------------------------------------      47 Athaliana(Q9FG08.2-ATXR4)/53-321                         16.2%     --------------------------------------------------------------------------------      48 Lgigantea(LOTGIDRAFT_232186)/323-670                     15.0%     --------------------------------------------------------GH----------------------      49 Bfloridae(XP_002589246.1-BRAFLDRAFT_74594)/380-720       14.2%     --------------------------------------------------------E-----------------------      50 Athaliana(Q5PP37-ATXR2)/52-466                           14.1%     --------------------------------------------------------------------------------      51 Cowczarzaki(EPH53581.1)/160-496                          15.7%     --------------------------------------------------------K-----------------------      52 Mbrevicollis(MONBRDRAFT_29283)/14-364                    16.5%     --------------------------------------------------------------------------------      53 Cowczarzaki(EFW42079.2)/57-422                           15.4%     --------------------------------------------------------------------------------      54 Tadhaerens(XP_002114620.1)/25-373                        17.6%     --------------------------------------------------------------------------------      55 Bfloridae(XP_002609030.1-BRAFLDRAFT_84846)/1-276         11.7%     --------------------------------------------------------------------------------      56 cintestinalis(XP_002127168.1)/13-358                     17.5%     --------------------------------------------------------------------------------      57 Dpulex(EFX89935.1)/23-367                                16.0%     --------------------------------------------------------------------------------      58 Dmelanogaster(CG3353-NP_650955.1)/13-363                 15.5%     --------------------------------------------------------------------------------      59 Hmagnipapillata(XP_002163562.2)/21-371                   16.0%     --------------------------------------------------------------------------------      60 Agambiae(XP_313299.1-AGAP003552-PA)/13-365               16.8%     --------------------------------------------------------D-----------------------      61 Amellifera(XP_394075.2-SMYD5-like-Prediction)/16-364     16.1%     --------------------------------------------------------------------------------      62 Nvectensis(XP_001627062.1)/18-370                        15.2%     --------------------------------------------------------------------------------      63 Skowalevskii(XP_002735533.1)/24-372                      13.9%     --------------------------------------------------------------------------------      64 Lgigantea(LOTGIDRAFT_231752)/19-367                      16.4%     --------------------------------------------------------------------------------      65 Drerio(F1RET2-Smyd5)/32-380                              16.4%     --------------------------------------------------------------------------------      66 Ggallus(NP_001012912.1-SMYD5)/39-387                     16.9%     --------------------------------------------------------------------------------      67 Hsapiens(Q6GMV2-SMYD5)/33-381                            15.8%     --------------------------------------------------------------------------------      68 Xtropicalis(A9ULL8-SMyd5)/32-382                         16.9%     --------------------------------------------------------------------------------      69 Mbrevicollis(MONBRDRAFT_36878)/153-462                   16.1%     -------------------------------------------GSWAQSHAHLPSQ-----------------PWWQSA-      70 Mbrevicollis(MONBRDRAFT_27776)/11-280                    20.3%     ----------------------------------------------------------------------------LV--      71 Cowczarzaki(EFW45970.2)/35-344                           21.5%     -------------------------------------------GGFGPDFDKLTPE--------------------LLG-      72 Dpulex(EFX73755.1)/45-306                                19.5%     --------------------------------------------------G-------------------------VLN-      73 Bfloridae(XP_002593048.1-BRAFLDRAFT_74375)/6-196         17.6%     --------------------------------------------------E-------------------------KLS-      74 Bfloridae(XP_002594298.1-BRAFLDRAFT_117670)/15-265       21.7%     --------------------------------------------------E-------------------------CLS-      75 Scerevisiae(Q12529.1)/23-365                             10.9%     ---------------------------TYRAFNMLQSNELSKIS----KFPVLLHFQKL-VFQTL---------------      76 Dmelanogaster(CG1868-NP_724802.1)/226-549                15.1%     --------------------------------------------------------------------------------      77 Agambiae(XP_319721.4-AGAP008973-PA)/165-486              14.6%     --------------------------------------------------------------------------------      78 Athaliana(NP_174606.2)/229-550                           12.6%     LE-------------------------LSHTYSKMNPENK------------------------LELHLLSIVLIWCLSK      79 Cintestinalis(XP_002123001.1)/195-567                    14.8%     ---------------------------LQEIFNSLNLKYLCENPNFDMDF--FRNRIK-TISE----M---------KSL      80 Drerio(Q08C84-Smyd4)/197-556                             15.4%     ----------MSFK----D-------------------------------------------------------------      81 Xtropicalis(NP_001072288.1-SMYD4)/212-545                18.0%     ----------SLTK----E-------------------------------------------------------------      82 Hsapiens(Q8IYR2-SMYD4)/244-602                           16.3%     ----------SSQL----K-------------------------------------------------------------      83 Ggallus(NP_001025886.1-SMYD4)/241-573                    15.3%     ----------EQKT----C-------------------------------------------------------------      84 Hmagnipapillata(XP_002160254.2/232-532                   15.5%     --------------------------------------------------------------------------------      85 Dpulex(DAPPUDRAFT_312722-Pedicted)/241-525               17.9%     --------------------------------------------------------------------------------      86 Amellifera(XP_006565387.1-SMYD4-like-Predicted)/278-571  17.3%     LE------------------------------DSLM-KKF-LNNFFNLNFNILTN-------------------------      87 Bfloridae(XP_002589088.1-BRAFLDRAFT_75068)/251-714       13.4%     TESTLEQASNMSQAESTPEQASNLSVKTDSVQNSMQDVEL-HRGNYSSVYNLMTHTEHHSVEQLLTQMMVSCLMCKCLGV      88 Lgigantea(LOTGIDRAFT_169490)/248-638                     12.9%     -E--IDDKGGTIYANEGKDADIANDS----KDNSLTNQKK-QNNSISTN--------EK---------------------      89 Skowalevskii(XP_002733823.1)/75-447                      16.7%     AE--AENKSNRSPSE--DESQINGSAPSRHTLDATDADRD-R-NTSQDN--------T----------------------      90 Nvectensis(XP_001627273.1)/170-547                       14.3%     LD--SDDDS--------DCSEV---------YNACEEQRT-QNGNFEQD--------R----------------------      91 Amellifera(XP_003250668.1-SMYD4-like-Predicted)/183-473  15.8%     --------------------------------------------------------------------------------      92 Cowczarzaki(XP_004349923.1)/103-371                      17.2%     --------------------------------------------------------------------------------      93 Amellifera(XP_001121272.2-SMYD4-like-Predicted)/230-549  19.0%     --------------------------------------------------------------------------------      94 Amellifera(XP_003249162.1-SMYD4-like-Predicted)/239-589  15.1%     --------------------------------------------------------------------------------      95 Dmelanogaster(CG14122-NP_648574.1)/265-541               16.9%     --------------------------------------------------------------------------------      96 Agambiae(XP_311885.3-AGAP002999-PA)/268-544              17.3%     --------------------------------------------------------------------------------      97 Amellifera(XP_392262.3-SMYD4-like-Predicted)/252-555     20.2%     --------------------------------------------------------------------------------      98 Dmelanogaster(CG7759-NP_725048.1)/250-537                16.4%     --------------------------------------------------------------------------------      99 Agambiae(XP_319583.4-AGAP008839-PA)/240-523              16.0%     --------------------------------------------------------------------------------     100 Dpulex(DAPPUDRAFT_68494-Predicted)/254-551               17.2%     --------------------------------------------------------------------------------     101 Dpulex(DAPPUDRAFT_309882)/300-599                        16.7%     --------------------------------------------------------------------------------     102 Dmelanogaster(CG8378-NP_610730.1)/196-491                17.4%     --------------------------------------------------------------------------------     103 Agambiae(XP_566179.1-AGAP000216-PA)/158-458              17.1%     --------------------------------------------------------------------------------     104 Agambiae(XP_564258.1-AGAP011234-PA)/216-546              17.1%     --------------------------------------------------------------------------------     105 Agambiae(XP_309407.4-AGAP011238-PA)/219-497              17.1%     --------------------------------------------------------------------------------     106 Agambiae(XP_314169.4-AGAP005253-PB)/218-514              16.6%     --------------------------------------------------------------------------------     107 Agambiae(XP_309409.4-AGAP011237-PA)/206-481              16.3%     --------------------------------------------------------------------------------     108 Agambiae(XP_307865.2-AGAP009448-PA)/166-466              16.5%     --------------------------------------------------------------------------------     109 Agambiae(XP_309762.4-AGAP010931-PA)/113-383              17.2%     --------------------------------------------------------------------------------     110 Agambiae(XP_309378.2-AGAP011267-PA)/149-447              17.9%     --------------------------------------------------------------------------------     111 Agambiae(XP_309383.4-AGAP011257-PA)/149-447              18.5%     --------------------------------------------------------------------------------     112 Agambiae(XP_307655.3-AGAP012638-PA)/149-447              18.5%     --------------------------------------------------------------------------------     113 Agambiae(XP_320681.4-AGAP011835-PA)/183-484              16.8%     --------------------------------------------------------------------------------     114 Agambiae(XP_309411.4-AGAP011232-PA)/162-434              16.4%     --------------------------------------------------------------------------------     115 Amellifera(XP_001120776.2-SMYD4-like-Predicted)/251-554  19.0%     --------------------------------------------------------------------------------     116 Dpulex(DAPPUDRAFT_305694-Predicted)/258-553              19.7%     --------------------------------------------------------------------------------     117 Dpulex(EFX87901.1)/258-554                               19.0%     --------------------------------------------------------------------------------     118 Amellifera(XP_001122116.2-SMYD4-like-Predicted)/234-534  17.2%     --------------------------------------------------------------------------------     119 Hmagnipapillata(XP_002159692.1)/239-485                  18.1%     --------------------------------------------------------------------------------     120 Nvectensis(XP_001623892.1)/215-512                       18.8%     --------------------------------------------------------------------------------     121 Lgigantea(LOTGIDRAFT_143433)/100-395                     19.3%     --------------------------------------------------------------------------------     122 Skowalevskii(XP_002740933.1)/253-549                     20.1%     --------------------------------------------------------------------------------         consensus/100%                                                     ................................................................................         consensus/90%                                                                                                                                               consensus/80%                                                                                                                                               consensus/70%                                                                                                                                                                                                               561          .         .         .         6         .         .         .         . 640   1 cintestinalis(NP_001071820.1)/15-282                    100.0%     -----------------------------------------------MPDS--DEEMAHLFAIIDCNAIGLNDH------       2 Drerio(Q6P0R5-Smyd1a)/18-279                             35.9%     -----------------------------------------------KAVG--EDYVSHLFGVISCNGFTLSDQ------       3 Derio(Q2MJQ9-Smyd1b)/13-274                              34.5%     -----------------------------------------------KPHT--VDSVSHILGVINCNGFMVSDQ------       4 Xtropicalis(NP_001120357.1-SMYD1)/13-261                 38.9%     -----------------------------------------------QQFG--MQYISHIFSVISCNGFTLSDQ------       5 Hsapiens(Q8NB12-SMYD1)/18-279                            38.4%     -----------------------------------------------QQFS--MQYISHIFGVINCNGFTLSDQ------       6 Ggallus(NP_989486.1-SMYD1)/13-274                        39.1%     -----------------------------------------------QQFG--MQYISHIFGVINCNAFTLSDQ------       7 Athaliana(Q7XJS0-ASHR1)/22-274                           25.8%     -----------------------------------------L-QFP--SVD--LREIAENFSKFSCNAHSICDS------       8 Amellifera(XP_625013.1-SMYD3-Predicted)/1-253            27.1%     --------------------------------------------M--SIPN--SAELMGIYGRIYINSFNISDL------       9 Dmelanogaster(Buzidau-CG13761)/26-282                    25.6%     --------------------------------------------SPSTVPN--KTELMSIYGRLITNGFNILDA------      10 Agambiae(XP_319707.4-AGAP008954-PA)/1-254                25.0%     --------------------------------------------A--SRPT--KAELLRIYGKMCINTFNILDA------      11 cintestinalis(XP_002128556.1)/14-266                     32.4%     -----------------------------------------K-PLPPQVTD--NKMLLELCARIKNNSFAICNE------      12 Lgigantea(LOTGIDRAFT_177746)/1-216                       26.2%     ----------------------------------------HM-TLPPA------HTLFSFFGMMVINTFSICND------      13 Drerio(E7EZZ6-SMYD3)/16-267                              34.7%     ----------------------------------------LS-RLPSG------LDPVSLLARVTCNCFSISDG------      14 Xtropicalis(XP_004914684.1|-SMYD3-Predicted)/15-264      31.0%     ----------------------------------------IS-QLPPG------FQVLEYFGKVTCNSFTISDG------      15 Hsapiens(Q9H7B4-SMYD3)/15-266                            33.9%     ----------------------------------------AS-QLPPA------FDLFEAFAKVICNSFTICNA------      16 Ggallus(XP_419536.1-SMYD3-Predicted)/15-266              33.2%     ----------------------------------------AS-HLPPA------IDFFQIFTKVTCNCFTISNG------      17 Drerio(Q5RGL7-Smyd2b)/19-268                             30.9%     -----------------------------------------------DFPD--HQALLTLFSQVHCNGFTVEDE------      18 Drerio(Q5BJI7-Smyd2a)/18-267                             32.0%     -----------------------------------------------DFPD--NAALTELIAQVNCNGFTIEDE------      19 Xtropicalis(XP_002934751.2-SMYD2-like-Predicted)/16-265  30.9%     -----------------------------------------------HYSD--NAAQVFLFAQVNCNGFTIEDE------      20 Hsapiens(Q9NRG4-SMYD2)/18-267                            32.0%     -----------------------------------------------GFPD--NDSLVVLFAQVNCNGFTIEDE------      21 Ggallus(XP_419420.1-SMYD2-Predicted)/21-270              31.6%     -----------------------------------------------EYPD--NAALVVLFAQVNCNGFTIEDE------      22 Tadhaerens(XP_002109888.1)/20-262                        28.4%     ------------------------------------------------AIN--ENEIIEIISRVTCNTFTICNS------      23 Hmagnipapillata(XP_002163555.2)/16-259                   29.4%     -----------------------------------------------ININ--DIDIYGLMCKASCNSFAITNA------      24 Nvectensis(XP_001627600.1)/17-253                        32.1%     -----------------------------------------------SPP-----EGLELFSKISCNSFAICDG------      25 Bfloridae(XP_002594889.1-BRAFLDRAFT_124463)/14-258       38.4%     ------------------------------------------------MDD--AREIFELFGRMTCNTFSICDP------      26 Skowalevskii(XP_006817727.1)/14-260                      33.2%     -----------------------------------------------LPND--VSDLLCIFGRMTSNSFSVCDS------      27 Amellifera(XP_006565332.1)/43-285                        18.4%     -----------------------------------------------ED----IEMMERVCRAFNTNSFETICVH-----      28 Dmelanogaster(msta-CG33548)/66-313                       17.7%     -----------------------------------------------DRVF--MDQLFRIVGVLNTNAFEAPCRS-----      29 Dmelanogaster(CG12119)/34-280                            19.0%     -----------------------------------------------KK-L--IEIMNRTVAVLRTNGFDKTTDR-----      30 Amellifera(XP_006565301.1)/26-284                        23.2%     -----------------------------------------------DPSV--SEMIQQLCGILDVNSFELRSPG-----      31 Dmelanogaster(CG9642)/21-271                             18.8%     -----------------------------------------------AA-D--EDLVQGLLGILDINAYEIRAPE-----      32 Dmelanogaster(CG9640)/17-268                             16.9%     -----------------------------------------------QELT--SDALHAHCIRIDSNSFEVT-AK-----      33 Amellifera(NP_001229486.1-LOC724300)/57-301              21.5%     ------------------------------------------------SST--KDIVSKICGLIDINALETVPPE-----      34 Dmelanogaster(CG14590-NP_610202.3)/55-322                18.9%     --------------------------------------------NLLTDCG--PEMLHRLCGIIETNFMVIELPS-----      35 Dmelanogaster(CG43129)/21-279                            17.1%     -----------------------------------------------NRFS--EELIMQVVGVLEVNAFEARSPK-----      36 Dmelanogaster(G11160)/58-319                             20.8%     ------------------------------------------------DLE--AEQVHEVCGILDVNCFEIGQN------      37 Amellifera(XP_624539.3-msta-like-Predicted)/54-297       21.5%     ------------------------------------------------DIS--EEEIATIIGILQVNGHEVPLT------      38 Dmelanogaster(CG8503-NP_610944.1)/52-301                 20.0%     ------------------------------------------------KFT--EEEIMKAVGALQINGHEVPTT------      39 Agambiae(XP_309979.4-AGAP011530-PA)/50-300               22.8%     -----------------------------------------------NKWD--EDEILRVVGIIQVNGHEVPMT------      40 Dpulex(DAPPUDRAFT_120473)/58-292                         19.1%     -----------------------------------------------DEFS--EEEIHASCGVIDVNAFEIRLA------      41 Dpulex(DAPPUDRAFT_194440-Predicted)/53-302               19.6%     -------------------------------------------------IP--EELILQLCGILMVNSFEQPPMK-----      42 Dpulex(DAPPUDRAFT_2393)/50-297                           20.2%     ------------------------------------------------QYD--SESIQRVCGILETNCFEIRIQ------      43 Dmelanogaster(CG18136-NP_649084.1)/58-318                22.6%     ------------------------------------------------DWP--EMDILRIAAILDTNTFEVRQPR-----      44 Agambiae(XP_309220.5-AGAP001025-PA)/55-318               20.7%     ------------------------------------------------QYS--EQTVLKLSAILDTNCYEIRLPE-----      45 Scerevisiae(P38890.1-SET5)/124-429                       16.1%     ---------------------------------------NGTKKGVEKNIDDETVWEKCY--ELFCGAFPKASEE-----      46 Athaliana(Q9ZUM9-ASHR2)/22-296                           14.0%     --------------------------------------------SIS------PDLTAALLSKDKVNAFGL---------      47 Athaliana(Q9FG08.2-ATXR4)/53-321                         16.2%     --------------------------------------------FLT------KQWYTAILARIRINAFRIDLVG-----      48 Lgigantea(LOTGIDRAFT_232186)/323-670                     15.0%     -------------------------------------------EGVVFNVT--EEEFNGRYYQATCNLQEF-SAR-STPY      49 Bfloridae(XP_002589246.1-BRAFLDRAFT_74594)/380-720       14.2%     ---------------------------------------------IKYKID--ELEFERRYYQVACNVQSF-GPP-CVTW      50 Athaliana(Q5PP37-ATXR2)/52-466                           14.1%     ---------------------------------------------CEALFS--LEIYGNIIGMFELNNLDLVVASPVEDY      51 Cowczarzaki(EPH53581.1)/160-496                          15.7%     --------------------------------------------LMEIVLT--IQNFRMLDGAIMRNAQRL-NPVS--DL      52 Mbrevicollis(MONBRDRAFT_29283)/14-364                    16.5%     ---------------------------------------------AQQWLK--PEGFRRLWSLIGTNGAGV-ASNTLAAY      53 Cowczarzaki(EFW42079.2)/57-422                           15.4%     ---------------------------------------------IPELFT--THGFRSLLALVGMNGQGV-GTTALDMY      54 Tadhaerens(XP_002114620.1)/25-373                        17.6%     ---------------------------------------------ITEWYT--PGGFRSLLAMIGTNGQGI-ASSSFSQY      55 Bfloridae(XP_002609030.1-BRAFLDRAFT_84846)/1-276         11.7%     ---------------------------------------------LDQWFT--PDGFRSIFAMIGRNGQGI-GTSSLSVY      56 cintestinalis(XP_002127168.1)/13-358                     17.5%     ---------------------------------------------VQHWFT--DDGFKNLFALLGTNQQGV-GTSALSVW      57 Dpulex(EFX89935.1)/23-367                                16.0%     ---------------------------------------------THEFLT--PQGFRSLIALIGRNGQGI-GTSAFSVW      58 Dmelanogaster(CG3353-NP_650955.1)/13-363                 15.5%     ---------------------------------------------FSIFKT--PDAFKTLMAILGTNSQGI-ATSVLSQW      59 Hmagnipapillata(XP_002163562.2)/21-371                   16.0%     ---------------------------------------------LSKLLT--ISGVQSLFALIGMNGQGI-GTSSLSEY      60 Agambiae(XP_313299.1-AGAP003552-PA)/13-365               16.8%     --------------------------------------------ERLSWLT--PEGFKSLVALVGTNGQGI-GTSSFGDW      61 Amellifera(XP_394075.2-SMYD5-like-Prediction)/16-364     16.1%     ---------------------------------------------IEHWFT--PEGFKSLLALVGTNGQGI-GTSAFSRW      62 Nvectensis(XP_001627062.1)/18-370                        15.2%     ---------------------------------------------LEQWFT--PEGFSSLFALVGTNGQGI-GTSSLSLY      63 Skowalevskii(XP_002735533.1)/24-372                      13.9%     ---------------------------------------------VSHWFT--SEGIRSLFALIGTNGQGV-GTSSLSVY      64 Lgigantea(LOTGIDRAFT_231752)/19-367                      16.4%     ---------------------------------------------IPQWFT--PEGFQSLFALIGTNGQGI-GSCSISVW      65 Drerio(F1RET2-Smyd5)/32-380                              16.4%     ---------------------------------------------LSQWFT--PEGFRSLFSLVGTNGQGI-GTSSLSQW      66 Ggallus(NP_001012912.1-SMYD5)/39-387                     16.9%     ---------------------------------------------LSRWFT--PEGFRSLFALVGTNGQGI-GTSSLSQW      67 Hsapiens(Q6GMV2-SMYD5)/33-381                            15.8%     ---------------------------------------------VSQWFT--PDGFRSLFALVGTNGQGI-GTSSLSQW      68 Xtropicalis(A9ULL8-SMyd5)/32-382                         16.9%     ---------------------------------------------VSRWFT--PEGFRSLFALVGTNGQGI-GTSSLSQW      69 Mbrevicollis(MONBRDRAFT_36878)/153-462                   16.1%     ----------------------------------------K-CTQCDLTTV--TGLRQHLLRVLRHNQHGLAQV------      70 Mbrevicollis(MONBRDRAFT_27776)/11-280                    20.3%     ----------------------------------------A-VTQCPGSTD--KQRVMDVLQRADCNNFSIW--------      71 Cowczarzaki(EFW45970.2)/35-344                           21.5%     ----------------------------------------E-EGRLFSTRP--EQFMLHLLCVMQCNNFAIH--------      72 Dpulex(EFX73755.1)/45-306                                19.5%     ----------------------------------------E-YLSNEILPN--SVELLGIYGRMCINSFNIL--------      73 Bfloridae(XP_002593048.1-BRAFLDRAFT_74375)/6-196         17.6%     ----------------------------------------R-YVNEDILPD--RAQLESLYGKTTCNCFAIH--------      74 Bfloridae(XP_002594298.1-BRAFLDRAFT_117670)/15-265       21.7%     ----------------------------------------Q-HMEKDALPD--RAFMEEIYGKIASNSFAIL--------      75 Scerevisiae(Q12529.1)/23-365                             10.9%     ------Y--------------------------------ILLPSHLHRMLS---IPLLRHILGTEY--------------      76 Dmelanogaster(CG1868-NP_724802.1)/226-549                15.1%     ---------------------------------------PASIEDWQLIIS---ALILRFAGQLLANGHVGDAL------      77 Agambiae(XP_319721.4-AGAP008973-PA)/165-486              14.6%     ---------------------------------------TMSPTELLVCCG---AFITRHIGQLVCNGHAISEL------      78 Athaliana(NP_174606.2)/229-550                           12.6%     SSCPNLSVCEA-------------------------------------SVT----QTIILLSQIKVNSIAVARM------      79 Cintestinalis(XP_002123001.1)/195-567                    14.8%     SKMSNVMTKIE----------------------------PGQLTSMGEVIE---YLLHRHYLQVPINGQSISFV------      80 Drerio(Q08C84-Smyd4)/197-556                             15.4%     --------------------EGV----------------KASWQPEMSMLG---ATALRHMMQLRCNAQAITAV------      81 Xtropicalis(NP_001072288.1-SMYD4)/212-545                18.0%     --------------------SGT----------------IEDWSSVRQFLG---PTVLRHMLQLYCNAQAVTAL------      82 Hsapiens(Q8IYR2-SMYD4)/244-602                           16.3%     --------------------AAV----------------TPELCPDVTIWG---VAMLRHMLQLQCNAQAMTTI------      83 Ggallus(NP_001025886.1-SMYD4)/241-573                    15.3%     --------------------GKT----------------SDELSPELMIMA---EAMLRHVLQLQCNAQAITVM------      84 Hmagnipapillata(XP_002160254.2/232-532                   15.5%     -------------------------------------------QQHHFGVG---SLLIRHIQQLICNAHAVTCL------      85 Dpulex(DAPPUDRAFT_312722-Pedicted)/241-525               17.9%     ------------------------------------------SLESQYLVG---GLILVHVCQMVSNAHAITEL------      86 Amellifera(XP_006565387.1-SMYD4-like-Predicted)/278-571  17.3%     -------------------------------------------NDKQLYIS---SLLLRYILQLISNGHAITKS------      87 Bfloridae(XP_002589088.1-BRAFLDRAFT_75068)/251-714       13.4%     DMCVEVVKKLG--LEGGNCTGATEGGGCGENKEGGDCAKSEEGVVCVEKMA---ALLCHHMQQLRCNAQAITTL------      88 Lgigantea(LOTGIDRAFT_169490)/248-638                     12.9%     SMESNIDGLLTNGKTEACQSSSNKTSFCGVL------------TNEMLDIG---GLLLRHIEQLVCNAHAITEV------      89 Skowalevskii(XP_002733823.1)/75-447                      16.7%     TVCSNDSNKFL---AA---------EQKLSL------------GDAEKAVA---SVLLHHLLQLRCNVHAVTEV------      90 Nvectensis(XP_001627273.1)/170-547                       14.3%     TICSRNTPYSR---QAYTSLGITTEEFCGKD------------GLSSDVVG---ALLVHHLQQMPCNVHAITAI------      91 Amellifera(XP_003250668.1-SMYD4-like-Predicted)/183-473  15.8%     -------------------------------------------N-DILNLT---KLLCRLCYIYDIHARM--DF------      92 Cowczarzaki(XP_004349923.1)/103-371                      17.2%     ------------------------------------------PA-AVRIPG---PEFLNIFHRHQCNAFSMLGP------      93 Amellifera(XP_001121272.2-SMYD4-like-Predicted)/230-549  19.0%     ------------------------------------------LKESFISLA---VAILHHLQAINCNAYEIVEN------      94 Amellifera(XP_003249162.1-SMYD4-like-Predicted)/239-589  15.1%     ---------------------------------------EETPNDREIAVA---SLLLKHLQLLQFNAHEVFET------      95 Dmelanogaster(CG14122-NP_648574.1)/265-541               16.9%     ---------------------------------------GVNPTAVELQVA---TALLGLLQVLQYNAHQIYQT------      96 Agambiae(XP_311885.3-AGAP002999-PA)/268-544              17.3%     ---------------------------------------APEPTEQELEVG---AVLLSALQSLQFNAHEVYET------      97 Amellifera(XP_392262.3-SMYD4-like-Predicted)/252-555     20.2%     ---------------------------------------EAKLSDGELYIG---GLILHNLMTIQFNAHEISEL------      98 Dmelanogaster(CG7759-NP_725048.1)/250-537                16.4%     -------------------------------------------PDEVSIIC---SLVLRSLQFIQFNTHEVAEL------      99 Agambiae(XP_319583.4-AGAP008839-PA)/240-523              16.0%     -------------------------------------------PQEQNFIG---GLLVHNLQLLQFNAHEVSEM------     100 Dpulex(DAPPUDRAFT_68494-Predicted)/254-551               17.2%     ---------------------------------------DLKFSDQERWIG---SLLLRHLQLLQFNAHEVSEL------     101 Dpulex(DAPPUDRAFT_309882)/300-599                        16.7%     ---------------------------------------SLNITEDEIYFA---SLLLRHLQLLQFNAHEIHEF------     102 Dmelanogaster(CG8378-NP_610730.1)/196-491                17.4%     ----------------------------------------GGEE-GVNFFT---DLLFRHLQTSPSNMHGIDLV------     103 Agambiae(XP_566179.1-AGAP000216-PA)/158-458              17.1%     ----------------------------------------SGSV-PYPWVG---EMCYRFLKVMQCNARPAQLT------     104 Agambiae(XP_564258.1-AGAP011234-PA)/216-546              17.1%     ----------------------------------------SCPS-RRKLLF---ELLLRHTQTALTNKNDVYHM------     105 Agambiae(XP_309407.4-AGAP011238-PA)/219-497              17.1%     ----------------------------------------SNPI-RSKLLF---DLLLRHVQTSLINKKRLNDY------     106 Agambiae(XP_314169.4-AGAP005253-PB)/218-514              16.6%     ----------------------------------------SSPT-RSKLLF---DLLLRHWQTSLINKKQV---------     107 Agambiae(XP_309409.4-AGAP011237-PA)/206-481              16.3%     ----------------------------------------SNPA-RSKLLF---DLLLRHVQTSPINKKQFNNF------     108 Agambiae(XP_307865.2-AGAP009448-PA)/166-466              16.5%     ----------------------------------------AKPS-IGKLLF---DLILRHVQVMRINRQFLSFY------     109 Agambiae(XP_309762.4-AGAP010931-PA)/113-383              17.2%     ----------------------------------------ANPT-ASKLLL---DLILRYLQITECNYKLLTCI------     110 Agambiae(XP_309378.2-AGAP011267-PA)/149-447              17.9%     ----------------------------------------ANPA-TNKFLL---DLILRYMQIVEFNRKLLSSN------     111 Agambiae(XP_309383.4-AGAP011257-PA)/149-447              18.5%     ----------------------------------------ANPA-TNKFLL---DLILRYMQIVNCNRKLLSFN------     112 Agambiae(XP_307655.3-AGAP012638-PA)/149-447              18.5%     ----------------------------------------ANPA-TNKILL---DLILRYEQIVECNSKLLSFN------     113 Agambiae(XP_320681.4-AGAP011835-PA)/183-484              16.8%     ----------------------------------------MNDN-VRELLN---ELILRHLQTGPVNMHSLHYM------     114 Agambiae(XP_309411.4-AGAP011232-PA)/162-434              16.4%     ----------------------------------------ADPI-LRKTLL---NLLLHHLQSTIVNHQFLHYM------     115 Amellifera(XP_001120776.2-SMYD4-like-Predicted)/251-554  19.0%     ---------------------------------------DINNY-SDIIFC--SSIMFRACVIMSSN-------------     116 Dpulex(DAPPUDRAFT_305694-Predicted)/258-553              19.7%     ---------------------------------------GKEDD-PDEILA---TAVLQLIQSYPCNAHEISHL------     117 Dpulex(EFX87901.1)/258-554                               19.0%     ----------------------------------------GKDR-PDEVLA---TAVLRLLHSYPCNAHEISHM------     118 Amellifera(XP_001122116.2-SMYD4-like-Predicted)/234-534  17.2%     ---------------------------------------LIKND-NVIFVG---GLILRHQQLIPSNIHSFSEE------     119 Hmagnipapillata(XP_002159692.1)/239-485                  18.1%     ----------------------------------------------LKIVC---AHLLKHIQMLPCNAHEVSEL------     120 Nvectensis(XP_001623892.1)/215-512                       18.8%     ---------------------------------------LPSRE-DQAFIG---GLLLRHLQSLPCNAHEISEL------     121 Lgigantea(LOTGIDRAFT_143433)/100-395                     19.3%     ---------------------------------------KAHLP-EKCVIG---GHILRHIQMLPCNAHEVSEF------     122 Skowalevskii(XP_002740933.1)/253-549                     20.1%     ---------------------------------------DNEED-VAIFIG---GHMLTHLQTIPCNAHEISEY------         consensus/100%                                                     ................................................................................         consensus/90%                                                                                             ............  ...h.thh..h.hNt..h.........         consensus/80%                                                                                              ..........s  .t.hhphht.h.hNs.tl....              consensus/70%                                                                                              .........hs  .thhhphhthltsNuhtl.t.                                                                               641          :         .         .         .         .         7         .         . 720   1 cintestinalis(NP_001071820.1)/15-282                    100.0%     -------------------------------------------------RGVQTIGVGIYPGISMLNHDCSPNCVAMNN-       2 Drerio(Q6P0R5-Smyd1a)/18-279                             35.9%     -------------------------------------------------RGLQSVGIGLFPNLCLVNHDCWPNCTVILN-       3 Derio(Q2MJQ9-Smyd1b)/13-274                              34.5%     -------------------------------------------------RGLQAVGVGLFPNLCLVNHDCWPNCTVILN-       4 Xtropicalis(NP_001120357.1-SMYD1)/13-261                 38.9%     -------------------------------------------------RGLQAVGVGIFPNLCLANHDCWPNCTVIFN-       5 Hsapiens(Q8NB12-SMYD1)/18-279                            38.4%     -------------------------------------------------RGLQAVGVGIFPNLGLVNHDCWPNCTVIFN-       6 Ggallus(NP_989486.1-SMYD1)/13-274                        39.1%     -------------------------------------------------RGLQAVGVGIFPNLCQANHDCWPNCTVIFN-       7 Athaliana(Q7XJS0-ASHR1)/22-274                           25.8%     -------------------------------------------------ELR-PQGIGLFPLVSIINHSCSPNAVLVFE-       8 Amellifera(XP_625013.1-SMYD3-Predicted)/1-253            27.1%     -------------------------------------------------DMN-NIGAGIYLGPSILDHSCKPNAVATFE-       9 Dmelanogaster(Buzidau-CG13761)/26-282                    25.6%     -------------------------------------------------EMN-SIATAIYLGVSITDHSCQPNAVATFE-      10 Agambiae(XP_319707.4-AGAP008954-PA)/1-254                25.0%     -------------------------------------------------EMS-TIGTGMYIGASIIDHSCRPNVVVSFD-      11 cintestinalis(XP_002128556.1)/14-266                     32.4%     -------------------------------------------------ELQSDVGTGVYLNCSFINHSCEPNCVAEFN-      12 Lgigantea(LOTGIDRAFT_177746)/1-216                       26.2%     -------------------------------------------------DLQ-PIGSGIYTSPSMLDHSCDPNAVAIFS-      13 Drerio(E7EZZ6-SMYD3)/16-267                              34.7%     -------------------------------------------------ELQ-DVGVGLYPSMSLLNHDCQPNCIMMFE-      14 Xtropicalis(XP_004914684.1|-SMYD3-Predicted)/15-264      31.0%     -------------------------------------------------EMQ-DVGVGLYPSMSLLNHSCDPNCVIVFE-      15 Hsapiens(Q9H7B4-SMYD3)/15-266                            33.9%     -------------------------------------------------EMQ-EVGVGLYPSISLLNHSCDPNCSIVFN-      16 Ggallus(XP_419536.1-SMYD3-Predicted)/15-266              33.2%     -------------------------------------------------EMQ-DVGVGLYPSMSLLNHSCDPNCVIIFE-      17 Drerio(Q5RGL7-Smyd2b)/19-268                             30.9%     -------------------------------------------------ELS-NLGLAIFPDIALLNHSCSPNVIVTYR-      18 Drerio(Q5BJI7-Smyd2a)/18-267                             32.0%     -------------------------------------------------ELS-HLGSALFPDVALMNHSCSPNVIVTYK-      19 Xtropicalis(XP_002934751.2-SMYD2-like-Predicted)/16-265  30.9%     -------------------------------------------------ELS-HLGSAIFPDVALMNHSCCPNVIVTYK-      20 Hsapiens(Q9NRG4-SMYD2)/18-267                            32.0%     -------------------------------------------------ELS-HLGSAIFPDVALMNHSCCPNVIVTYK-      21 Ggallus(XP_419420.1-SMYD2-Predicted)/21-270              31.6%     -------------------------------------------------ELS-HLGSAIFPDVALMNHSCCPNVIVTYK-      22 Tadhaerens(XP_002109888.1)/20-262                        28.4%     -------------------------------------------------EMQ-TVGIGVYPGLSLVNHSCSPNCSATFR-      23 Hmagnipapillata(XP_002163555.2)/16-259                   29.4%     -------------------------------------------------ELN-SLGTGIFSSASLFNHSCDPNCVATFN-      24 Nvectensis(XP_001627600.1)/17-253                        32.1%     -------------------------------------------------EMQ-AIGTGIFPNAVCLNHSCAPNSVAVFN-      25 Bfloridae(XP_002594889.1-BRAFLDRAFT_124463)/14-258       38.4%     -------------------------------------------------EMQ-YIGIGIYPKMSLFNHSCEPNCVAVFN-      26 Skowalevskii(XP_006817727.1)/14-260                      33.2%     -------------------------------------------------EMK-PIGVGIYPSASLLNHSCDPNCVAVFN-      27 Amellifera(XP_006565332.1)/43-285                        18.4%     -----------------------------------------------D-KDHFTNLRGLYPLGSLQNHCCIPNTRHYFDE      28 Dmelanogaster(msta-CG33548)/66-313                       17.7%     -------------------------------------------------GGHETLLRGLFPLTAIMNHECTPNASHYFEN      29 Dmelanogaster(CG12119)/34-280                            19.0%     -----------------------------------------------TNDNQEFNYRALYPLFGVVNHDCIPNAYYTFEE      30 Amellifera(XP_006565301.1)/26-284                        23.2%     -------------------------------------------------GMDGLLLRGLYLEASMMAHDCRGNVHVTADD      31 Dmelanogaster(CG9642)/21-271                             18.8%     ---------------------------------------------------VGGAMRGLYRRAGLFAHSCTPNLVISIDD      32 Dmelanogaster(CG9640)/17-268                             16.9%     ---------------------------------------------------DGDTLKGIFVWGATLPHHCVPNTVVALDE      33 Amellifera(NP_001229486.1-LOC724300)/57-301              21.5%     ------------------------------------------------------GCVAIYETACLLEHSCLANTRHSFTI      34 Dmelanogaster(CG14590-NP_610202.3)/55-322                18.9%     -------------------------------------------------G---VELSGLFRQACMMEHACQPNCDFQFDN      35 Dmelanogaster(CG43129)/21-279                            17.1%     -------------------------------------------------G---YPLRCLFPYTGILAHNCVPNTSRSIYP      36 Dmelanogaster(G11160)/58-319                             20.8%     ----------------------------------------------------GAKARTLYPSAFLLAHDCTPNTAHTDDP      37 Amellifera(XP_624539.3-msta-like-Predicted)/54-297       21.5%     ----------------------------------------------------DSPYVAVYEMASLIEHNCRANCSKSFTD      38 Dmelanogaster(CG8503-NP_610944.1)/52-301                 20.0%     ----------------------------------------------------DPSHVAVFYTASFTENSCLPNLAKSFNK      39 Agambiae(XP_309979.4-AGAP011530-PA)/50-300               22.8%     ----------------------------------------------------EPSSVAIYNMASMLEHSCRPNLAKSFTN      40 Dpulex(DAPPUDRAFT_120473)/58-292                         19.1%     -------------------------------------------------GNQYQQVLGVFPLASMMSHNCVANTQHVIDA      41 Dpulex(DAPPUDRAFT_194440-Predicted)/53-302               19.6%     -------------------------------------------------GNSQHGLVAVYSTASLLEHDCVANAIKTFTN      42 Dpulex(DAPPUDRAFT_2393)/50-297                           20.2%     -------------------------------------------------G--RVSVRGLYPTASLMNHDCVANTRHVFDP      43 Dmelanogaster(CG18136-NP_649084.1)/58-318                22.6%     -------------------------------------------------E--RRKIRALYPGAAMISHDCVPNMRHRFDD      44 Agambiae(XP_309220.5-AGAP001025-PA)/55-318               20.7%     -------------------------------------------------Q--HVKVRGLYPLGAMLSHDCRPNTKHYFDD      45 Scerevisiae(P38890.1-SET5)/124-429                       16.1%     ---------I-D--FEKFL-----------------------TM--IGTFNINQYNGQVYHWISFINHDCEPNAYIEQVE      46 Athaliana(Q9ZUM9-ASHR2)/22-296                           14.0%     ------------------M-----------------------EPCSVSNEKRSVRAYGIYPKTSFFNHDCLPNACRFDYV      47 Athaliana(Q9FG08.2-ATXR4)/53-321                         16.2%     ---------GSC--GEDLL-----------------------SLAAASVEGEGAVGHAVYMLPSFYNHDCDPNAHIIWLH      48 Lgigantea(LOTGIDRAFT_232186)/323-670                     15.0%     HAFMKKLS---TD--------LRGF------QMIKYL----------EKSPPYAGFCGMFPLHACLNHSCCNNVEIRDG-      49 Bfloridae(XP_002589246.1-BRAFLDRAFT_74594)/380-720       14.2%     HEFVAEFH---RT--------ARPG--ENHRRVAQEM----------RGEPKDVTFGGLYALQSSLNHSCDKNVDVMDA-      50 Athaliana(Q5PP37-ATXR2)/52-466                           14.1%     FLYIDDLP---DAEKEETEEITRPF----LDALGDEY-------------SDCCQGTAFFPLQSCMNHSCCPNAKAFKR-      51 Cowczarzaki(EPH53581.1)/160-496                          15.7%     HAMIDRL---AQIDAHKLA----AV----LGKIGFKIPQLPGLRMSTPMRSLTVSGSGLFEIGNTMNHSCQPNVVSMTR-      52 Mbrevicollis(MONBRDRAFT_29283)/14-364                    16.5%     DRQLSALD-LDDATQSEVD----NT----MNTIYEIAGEV-------VGEFLDAEGSAIYATHSACNHSCRPNAKVFFE-      53 Cowczarzaki(EFW42079.2)/57-422                           15.4%     LVAVERLN-LSESDAKTRD----QF----VEKLLDDIDEH-------SGEFDACEGSALYCLQSCCNHNCQPNAVPTFT-      54 Tadhaerens(XP_002114620.1)/25-373                        17.6%     ARNVDAAK-FEKQEEDYIN----SF----LDQLYADMNEE-------SGDFLDCEGSGLYLLQSCCNHDCSPNVEINFL-      55 Bfloridae(XP_002609030.1-BRAFLDRAFT_84846)/1-276         11.7%     VHNCDALE-LPSQDREKLD----AF----IDQLYVDMEH--------------------------GNHSCEPTAEPSFD-      56 cintestinalis(XP_002127168.1)/13-358                     17.5%     VHNCDELD-LNPQDKEELD----NL----IDGLYEELENV-------AGSFLNCEGAGLYRIQSKCNHSCEPNAEVCFP-      57 Dpulex(EFX89935.1)/23-367                                16.0%     VRKVSEND-----IDPTTD----AL----IDTIYQEMENE-------SGDFLNNEGSALFAIQSACNHSCEPNCISTFP-      58 Dmelanogaster(CG3353-NP_650955.1)/13-363                 15.5%     VAKVSDLP-LTDSEKEQLD----TV----IDGLYAKVGEF-------AGEFLNNEGSGLYLLQSKINHSCVPNACSTFP-      59 Hmagnipapillata(XP_002163562.2)/21-371                   16.0%     VHNIDAKV-MSDNEREQID----AF----IDQLYLHMEKE-------SGSFLNCEGSGLFKMQSRCNHSCYPNAEATFP-      60 Agambiae(XP_313299.1-AGAP003552-PA)/13-365               16.8%     VKNATACE-MSDQERQAVD----QL----IDDLYAKMDDV-------VGSFLNNEGSALYARQSKINHSCAPNAETVFP-      61 Amellifera(XP_394075.2-SMYD5-like-Prediction)/16-364     16.1%     VKNVSALE-LPREERIQVD----KL----IDRIYDDMEEA-------VASFLNNEGSGLYILQSSVNHSCVPNAIVEFP-      62 Nvectensis(XP_001627062.1)/18-370                        15.2%     VHNIDSYPALSDDERQAID----IF----LNQLYEEMERV-------SGQFLNCEGAGLYALQSSCNHSCAPNAEVTFP-      63 Skowalevskii(XP_002735533.1)/24-372                      13.9%     VHNCDALD-LNTDDRQRLD----LF----IDQLYVDIEKE-------SGSFLNCEGSALYSLQSCCNHSCVPNAEVTFP-      64 Lgigantea(LOTGIDRAFT_231752)/19-367                      16.4%     VKNCEDLE-LPEDKKTELD----DF----IDQMYEELEKE-------SGSFLNCEGSGLYELTSSCNHSCDPNAGITFP-      65 Drerio(F1RET2-Smyd5)/32-380                              16.4%     VHACDALE-LPRQQREQLD----AF----IDQLYKDIDKE-------TGDFLNCEGSGLFLLQSSCNHSCVPNAEASFP-      66 Ggallus(NP_001012912.1-SMYD5)/39-387                     16.9%     VHACDALD-LPMLQREELD----AF----IDQLYKDIEKE-------SGEFLNCEGSGLYMLQSCCNHSCIPNAETSFP-      67 Hsapiens(Q6GMV2-SMYD5)/33-381                            15.8%     VHACDTLE-LKPQDREQLD----AF----IDQLYKDIEAA-------TGEFLNCEGSGLFVLQSCCNHSCVPNAETSFP-      68 Xtropicalis(A9ULL8-SMyd5)/32-382                         16.9%     VHACDALE-LPPREREQLD----SL----IDQLYKDIEKV-------TGEFLNCEGSGLYLLQSCCNHSCVPNAEASFP-      69 Mbrevicollis(MONBRDRAFT_36878)/153-462                   16.1%     -------ELQPSE------------------------------QNGEVGVVHQVYGAGLFVHGSLFNHSCVPNVHLHFH-      70 Mbrevicollis(MONBRDRAFT_27776)/11-280                    20.3%     ------------------------------------------------DELLLPRGAGVYPWGAILNHSCEPNCVMTYR-      71 Cowczarzaki(EFW45970.2)/35-344                           21.5%     ------------------------------------------------NDILFARGSGIYPVAALVNHACVANCVLTYD-      72 Dpulex(EFX73755.1)/45-306                                19.5%     ------------------------------------------------NGEMQAIGTGIYLAPSILDHSCSPNAVATFD-      73 Bfloridae(XP_002593048.1-BRAFLDRAFT_74375)/6-196         17.6%     ------------------------------------------------NLDLREIGVGLYPQAAMINHSCKSNCVSTFR-      74 Bfloridae(XP_002594298.1-BRAFLDRAFT_117670)/15-265       21.7%     ------------------------------------------------DENMCSIGIGVYPQASMINHSCKSNCIGMFY-      75 Scerevisiae(Q12529.1)/23-365                             10.9%     -------------------GN--AF---GLWQEG------------EASDSREYFGYWVFPEASYFNHSCNPNITKYRK-      76 Dmelanogaster(CG1868-NP_724802.1)/226-549                15.1%     --------LGVGMEPKEFVML--QP---ELWQKP----RHLKRGQLHNLSHSDPITAINLPYLSLCNHACEPSIRTKFD-      77 Agambiae(XP_319721.4-AGAP008973-PA)/165-486              14.6%     --------RLALPSKGQFYNL--N---------D----SLLLAGTLHLCLKSSRVFTAIFPRISMFNHSCDPNIRNHFE-      78 Athaliana(NP_174606.2)/229-550                           12.6%     --------KSSGDSFKCLPSG--N----------------ISTKEPIQSLEQIRVGQALYKTGSLFNHSCKPNIHLYFL-      79 Cintestinalis(XP_002123001.1)/195-567                    14.8%     --------TEELC-------------------------------DNVTVTRRDIVASAFFPTMSMMNHSCDCNTDALFN-      80 Drerio(Q08C84-Smyd4)/197-556                             15.4%     --------RVKEE---------------------------SG--MAVQSSSEIRIATAIFPVLSLLNHSCSPNTSISFT-      81 Xtropicalis(NP_001072288.1-SMYD4)/212-545                18.0%     --------QENED---------------------------ESSLSLVKSNKSIRLATAVFPVLSLLNHSCDPNTTVSFT-      82 Hsapiens(Q8IYR2-SMYD4)/244-602                           16.3%     --------QHTGP---------------------------KG--SIVTDSRQVRLATGIFPVISLLNHSCSPNTSVSFI-      83 Ggallus(NP_001025886.1-SMYD4)/241-573                    15.3%     --------QELES---------------------------GD--GAVVNKKPVRLATAFFPVLSLLNHSCSPNISVSFS-      84 Hmagnipapillata(XP_002160254.2/232-532                   15.5%     --------SAEKL-----------------------------DTTSVIDQEQVRIATAIYPTTSLLNHSCEPTILNCFH-      85 Dpulex(DAPPUDRAFT_312722-Pedicted)/241-525               17.9%     --------CLIDE----------------------------------NNERQERIATAIYPSASLMNHNCDPTVINSFQ-      86 Amellifera(XP_006565387.1-SMYD4-like-Predicted)/278-571  17.3%     --------NIFLS----------------------------ENDSS--MIQQDIVATGIYPSASIMNHSCDPNIINIFV-      87 Bfloridae(XP_002589088.1-BRAFLDRAFT_75068)/251-714       13.4%     --------QEQDS-------------------------------VSLLEDKQVRLATAVFPTEALLNHSCRPNVFVSFQ-      88 Lgigantea(LOTGIDRAFT_169490)/248-638                     12.9%     --------QCTDT----------------------------INDSMILDTSQVRIATAIYPTASLMNHSCDPTIISSFH-      89 Skowalevskii(XP_002733823.1)/75-447                      16.7%     --------ATKTD----------------------------SSTSFVATTQQIRIAVAVYGTASMLNHSCTPNVIAGYD-      90 Nvectensis(XP_001627273.1)/170-547                       14.3%     --------VSTSSSDEE-DEE--M----------------GSSHDQVVAREQRRIASAIYPTASLLNHACDPDVLVSFV-      91 Amellifera(XP_003250668.1-SMYD4-like-Predicted)/183-473  15.8%     ---------------------------------------------VPIFERYIALLQNLYFLLNLVRHSCSGNTIYTVHK      92 Cowczarzaki(XP_004349923.1)/103-371                      17.2%     -------------------------------------------------GRMDTMYGAYTACWHLMNHSCAPVLMREHHA      93 Amellifera(XP_001121272.2-SMYD4-like-Predicted)/230-549  19.0%     --------IYDK---------------------------------KTHIWEPRQIGGAIYPSVSLINHSCYPNVVRHTYP      94 Amellifera(XP_003249162.1-SMYD4-like-Predicted)/239-589  15.1%     --------RLGME-------------------------------HRFRGSKPIYIGVAIYPTVARFNHDCYPAVTRYFL-      95 Dmelanogaster(CG14122-NP_648574.1)/265-541               16.9%     --------QVTEE-------------------------------HRFDGSKTVYLAAGLYGTGSYFNHECWPSTACHFV-      96 Agambiae(XP_311885.3-AGAP002999-PA)/268-544              17.3%     --------RITGE-------------------------------HRFDTAKVQYIGVGIYRGASMFNHECYPGVTRTFL-      97 Amellifera(XP_392262.3-SMYD4-like-Predicted)/252-555     20.2%     --------VIPKA------------------------------DNNLANAKSKFIGGGLYPTISLFNHSCNPGIIRYFI-      98 Dmelanogaster(CG7759-NP_725048.1)/250-537                16.4%     --------HKFSS------------------------------S---GREKSIFIGGAIYPTLALFNHSCDPGVVRYFR-      99 Agambiae(XP_319583.4-AGAP008839-PA)/240-523              16.0%     --------IRETA------------------------------E---DIGKSTFIGGGLYPTLALFNHSCDPGVTRYYR-     100 Dpulex(DAPPUDRAFT_68494-Predicted)/254-551               17.2%     --------RMDRP-------------------------------GCMEGAKTFFLGAGVYSTVALLNHSCEPGVIRHFI-     101 Dpulex(DAPPUDRAFT_309882)/300-599                        16.7%     --------VQLNE-------------------------------KNMRSTKTVYIGVGIYPTVAFFNHSCRPDVARYFL-     102 Dmelanogaster(CG8378-NP_610730.1)/196-491                17.4%     -------EQV------------------------------------NETKDDQTHSSGAYAFLSLINHSCAPNTVRIYE-     103 Agambiae(XP_566179.1-AGAP000216-PA)/158-458              17.1%     ----------------------------------------RR-DEPEGQYRAVPFALRCHPLISLLNHSCAPNVKCFDLR     104 Agambiae(XP_564258.1-AGAP011234-PA)/216-546              17.1%     -------ERVKLEDDHVEDDE--SEEDSSVDNNN----SDDRKSPRNVHHEERTHAIAIYPLFSMVNHSCIPNVAPIHLL     105 Agambiae(XP_309407.4-AGAP011238-PA)/219-497              17.1%     -------FDY------------------------------DSDEGKPTHFEERPHAMAVYPLSSMLNHSCVPNVAPINLL     106 Agambiae(XP_314169.4-AGAP005253-PB)/218-514              16.6%     ------------------------------------------D--DEEEYSDEMHAIAVYPLFSMVNHSCIPNVAPIHLL     107 Agambiae(XP_309409.4-AGAP011237-PA)/206-481              16.3%     --------GY------------------------------DSD--DEDIFEERTHAIAVYPLFSMANHSCIPNVAPIHLL     108 Agambiae(XP_307865.2-AGAP009448-PA)/166-466              16.5%     --------EH---------------------------------RPDRQRFQAKEYGTACYPLVSMFNHSCASNVRRLILR     109 Agambiae(XP_309762.4-AGAP010931-PA)/113-383              17.2%     --------KI---------------------------------T--NRNPEDETFTTSCYPLISMLNHSCAPNVRRLILP     110 Agambiae(XP_309378.2-AGAP011267-PA)/149-447              17.9%     --------AY---------------------------------K--VKKYVAESLATSCYPLISMLNHSCAPNVQRITLR     111 Agambiae(XP_309383.4-AGAP011257-PA)/149-447              18.5%     --------AY---------------------------------K--VNEYVAESFAVGCYPLISMLNHSCAPNVKRITLP     112 Agambiae(XP_307655.3-AGAP012638-PA)/149-447              18.5%     --------AY---------------------------------K--VKEYVAESFAVGCYPLISMLNHSCAPNVQRITLP     113 Agambiae(XP_320681.4-AGAP011835-PA)/183-484              16.8%     --------EY---------------------------------QPEQRVYEMENHVSACFPILSMLNHSCAPNVTRITLR     114 Agambiae(XP_309411.4-AGAP011232-PA)/162-434              16.4%     --------DY---------------------------------LAEQDVYEPDEYAIACFPLLSMLNHSCAPNVKRITMR     115 Amellifera(XP_001120776.2-SMYD4-like-Predicted)/251-554  19.0%     -------------------------------------------CFSVQQEPGIKIGSGLYVTNSLYNHSCAPNTFRHFE-     116 Dpulex(DAPPUDRAFT_305694-Predicted)/258-553              19.7%     --------AFPLPGTPSG----------------------PDLPSTLQQIRLCEIGAAAMPVLSLINHSCDPNVVRDCY-     117 Dpulex(EFX87901.1)/258-554                               19.0%     --------AIPVPSGFCA----------------------QSKSLQLQQIQSCEIGSAAFPVVSLMNHSCNPNVVHLCY-     118 Amellifera(XP_001122116.2-SMYD4-like-Predicted)/234-534  17.2%     --------C---------------------------------------GLDAVERGIAAMPFFSLINHSCNPNILRHSR-     119 Hmagnipapillata(XP_002159692.1)/239-485                  18.1%     --------QLKAS--------------------------------NYKDSELKEIGSAVYATLSLLNHSCDPSVVRHCY-     120 Nvectensis(XP_001623892.1)/215-512                       18.8%     --------QLSLK--------------------------------SVATSEAAEIGAGIYGTLSLFNHSCEPNVTRFFY-     121 Lgigantea(LOTGIDRAFT_143433)/100-395                     19.3%     --------AYREY--------------------------------DLPNSQTMEIGSGIYATLSLINHSCDPNVVRHSY-     122 Skowalevskii(XP_002740933.1)/253-549                     20.1%     --------ELWRS--------------------------------DITKCHFVEVGSGLYPTMSLVNHSCDPVVTRNCY-         consensus/100%                                                     ...................................................................ptC..s.......         consensus/90%                                                      ...................    ..    ..........................h.uha...uhhsHsC.ssh...h..         consensus/80%                                                              ...........    .         .         ............s.ula..huhhNHsC.PNs.h.h..         consensus/70%                                                              .....                                ....t...thusulashhShhNHSCtPNshhha.                                                                          721          .         .         :         .         .         .         .         8 800   1 cintestinalis(NP_001071820.1)/15-282                    100.0%     G----------------------------------PRLE--VRALRVIQ--P--G--EEL--------CISYIDSLETTE       2 Drerio(Q6P0R5-Smyd1a)/18-279                             35.9%     HGDQSALDAS---------------------FHSSRRIE--LRALEPIS--A--G--QEL--------TVSYVDFLSVST       3 Derio(Q2MJQ9-Smyd1b)/13-274                              34.5%     NGNQSAIDTV---------------------FHSQKRIE--LRALGKIS--A--G--EEV--------TVAYVDYLNVSA       4 Xtropicalis(NP_001120357.1-SMYD1)/13-261                 38.9%     NGK----------------------------------IE--LRALGKIN--K--G--EEL--------TVSYVDFLNLTE       5 Hsapiens(Q8NB12-SMYD1)/18-279                            38.4%     NGNHEAVKSM---------------------FHTQMRIE--LRALGKIS--E--G--EEL--------TVSYIDFLNVSE       6 Ggallus(NP_989486.1-SMYD1)/13-274                        39.1%     NGNHEAVRSM---------------------FHTQMRIE--LRALSKIS--P--G--DEL--------TVSYVDFLNVSE       7 Athaliana(Q7XJS0-ASHR1)/22-274                           25.8%     E----------------------------------QMAV--VRAMDNIS--K--D--SEI--------TISYIETAGSTL       8 Amellifera(XP_625013.1-SMYD3-Predicted)/1-253            27.1%     G----------------------------------TTII--IRTTEDL--PC--LDLSQI--------RISYIDVIKTTK       9 Dmelanogaster(Buzidau-CG13761)/26-282                    25.6%     G----------------------------------NELH--VHAIEDM--EC--LDWSKI--------FISYIDLLNTPE      10 Agambiae(XP_319707.4-AGAP008954-PA)/1-254                25.0%     G----------------------------------ETLR--MRLLEDYPEQE--LDFGKL--------FISYIDLIDTAE      11 cintestinalis(XP_002128556.1)/14-266                     32.4%     M----------------------------------RTLK--IRAVKNIT--A--G--EEV--------LISYVDLFATSF      12 Lgigantea(LOTGIDRAFT_177746)/1-216                       26.2%     G----------------------------------KTVF--IRALKDIP--D--TTPNKM--------FISYIDQLKPSV      13 Drerio(E7EZZ6-SMYD3)/16-267                              34.7%     G----------------------------------KRLT--LRAVRVIR--S--A--EEL--------TISYTDILAPSK      14 Xtropicalis(XP_004914684.1|-SMYD3-Predicted)/15-264      31.0%     G----------------------------------TCLL--LRTVKEIP--K--G--EEL--------TISYIDVKMPTQ      15 Hsapiens(Q9H7B4-SMYD3)/15-266                            33.9%     G----------------------------------PHLL--LRAVRDIE--V--G--EEL--------TICYLDMLMTSE      16 Ggallus(XP_419536.1-SMYD3-Predicted)/15-266              33.2%     G----------------------------------YQLL--LRSIREIQ--I--G--EEL--------TISYIESLMPTS      17 Drerio(Q5RGL7-Smyd2b)/19-268                             30.9%     G----------------------------------INAE--VRAVKDIS--P--G--QEI--------YTSYIDLLYPTA      18 Drerio(Q5BJI7-Smyd2a)/18-267                             32.0%     G----------------------------------TVAE--VRAVQEIN--P--E--EEI--------FNSYIDLLYPTE      19 Xtropicalis(XP_002934751.2-SMYD2-like-Predicted)/16-265  30.9%     G----------------------------------TVAE--VRAVQEIH--A--G--DEV--------FTSYIDLLYPTE      20 Hsapiens(Q9NRG4-SMYD2)/18-267                            32.0%     G----------------------------------TLAE--VRAVQEIK--P--G--EEV--------FTSYIDLLYPTE      21 Ggallus(XP_419420.1-SMYD2-Predicted)/21-270              31.6%     G----------------------------------TLAE--VRAVKEIE--P--G--EEV--------FTSYIDLLYPTE      22 Tadhaerens(XP_002109888.1)/20-262                        28.4%     G----------------------------------KQMQ--LRIIENTK--I--G--DEL--------LISYIDPMQVLS      23 Hmagnipapillata(XP_002163555.2)/16-259                   29.4%     G----------------------------------RDIS--IRAIKPIA--E--G--EEL--------MLSYISILATSD      24 Nvectensis(XP_001627600.1)/17-253                        32.1%     G----------------------------------TNIY--IKALEEIP--V--G--EEL--------TISYIQQLHPRE      25 Bfloridae(XP_002594889.1-BRAFLDRAFT_124463)/14-258       38.4%     G----------------------------------LRME--VRAIQNIQ--P--G--EEL--------LISYVEMLAMSS      26 Skowalevskii(XP_006817727.1)/14-260                      33.2%     G----------------------------------TDLC--IRAVKPIS--V--G--DEC--------VISYIEMMSTTS      27 Amellifera(XP_006565332.1)/43-285                        18.4%     -------------------------------K--FR-LY--VRAALPIS--A--G--EEI--------TMSYTSLFWDTT      28 Dmelanogaster(msta-CG33548)/66-313                       17.7%     -------------------------------G---RLAV--VRAARDIP--K--G--GEI--------TTTYTKILWGNL      29 Dmelanogaster(CG12119)/34-280                            19.0%     -------------------------------K--TNNMI--VRAAVDIP--E--G--FEV--------TTTYTKLFTGNI      30 Amellifera(XP_006565301.1)/26-284                        23.2%     ----------------------------------NFHLT--VYASIPIK--E--G--DTI--------FFNYTSSLLGTT      31 Dmelanogaster(CG9642)/21-271                             18.8%     ----------------------------------EQRIK--VYANRFIA--A--G--EIL--------YNCYTNVLLGTE      32 Dmelanogaster(CG9640)/17-268                             16.9%     ----------------------------------QFNMK--LYAAVPLQ--P--G--DII--------YNSYTNPLMGTS      33 Amellifera(NP_001229486.1-LOC724300)/57-301              21.5%     -------------------------------DKGRPRIT--VKALCSIQ--K--G--DHL--------STMYTHALWATR      34 Dmelanogaster(CG14590-NP_610202.3)/55-322                18.9%     -------------------------------K--TQQVA--VRAGCDLR--K--G--DHL--------RITYTNILWGTQ      35 Dmelanogaster(CG43129)/21-279                            17.1%     -------------------------------S-EGYKIR--LRAMVDLE--E--G--QPL--------HHSYTYTLDGTA      36 Dmelanogaster(G11160)/58-319                             20.8%     -------------------------------S--SFEIL--LRTSRRVR--E--R--EAL--------TLSYAYTLQGTL      37 Amellifera(XP_624539.3-msta-like-Predicted)/54-297       21.5%     ----------------------------------MGGLI--IRAALPIT--K--G--DHI--------SICYTDPLWGTA      38 Dmelanogaster(CG8503-NP_610944.1)/52-301                 20.0%     ----------------------------------NGHCI--LWAPREIK--K--N--AHL--------SICYSDAMWGTA      39 Agambiae(XP_309979.4-AGAP011530-PA)/50-300               22.8%     ----------------------------------RGEVV--MWAPNPIR--R--G--DRL--------SICYTDVLWTTG      40 Dpulex(DAPPUDRAFT_120473)/58-292                         19.1%     ----------------------------------NYTMT--VRASVPIM--K--G--EQI--------FTSYTLPLEGTK      41 Dpulex(DAPPUDRAFT_194440-Predicted)/53-302               19.6%     ----------------------------------KGDIV--IRAAVPIP--K--G--EKI--------ALCYTEPLWGTM      42 Dpulex(DAPPUDRAFT_2393)/50-297                           20.2%     -------------------------------A--DFRIR--ILATKDIP--A--G--DKI--------SATYTRSLWNTL      43 Dmelanogaster(CG18136-NP_649084.1)/58-318                22.6%     ----------------------------------DMNIV--FLAKRKIA--K--G--EIL--------SISYTQPLRSTI      44 Agambiae(XP_309220.5-AGAP001025-PA)/55-318               20.7%     ----------------------------------RLHMV--LVATVDIP--A--G--GVI--------HASYTQPLLGTV      45 Scerevisiae(P38890.1-SET5)/124-429                       16.1%     ---------------------------------EHEELR--LHARKPIK--K--G--EQI--------RITYVNPLHGVR      46 Athaliana(Q9ZUM9-ASHR2)/22-296                           14.0%     ----------------------------DSASDGNTDII--IRMIHDVP--E--G--REV--------CLSYFPVNMNYS      47 Athaliana(Q9FG08.2-ATXR4)/53-321                         16.2%     ----------------------------------NADAR--LNTLRDVE--E--G--EEL--------RICYIDASMGYE      48 Lgigantea(LOTGIDRAFT_232186)/323-670                     15.0%     D--------------------------C----NGTPGVN--VVAKRFIK--T--G--EEL--------FTSYIDNKL---      49 Bfloridae(XP_002589246.1-BRAFLDRAFT_74594)/380-720       14.2%     V--------------------------V----DGKPGVV--IRAKQPIK--K--G--GEL--------YTTYIDTSM---      50 Athaliana(Q5PP37-ATXR2)/52-466                           14.1%     E--------------------------E----DRDGQAV--IIALRRIS--K--N--EEV--------TISYIDEELPY-      51 Cowczarzaki(EPH53581.1)/160-496                          15.7%     A--------------------------T------DFTLS--VVAVATIP--V--N--TEV--------CISYIDTDL---      52 Mbrevicollis(MONBRDRAFT_29283)/14-364                    16.5%     G--------------------------G------NFELT--IRAEQDIA--P--G--EASCLFDTSEVTISYLDDHILDH      53 Cowczarzaki(EFW42079.2)/57-422                           15.4%     E--------------------------N------NATLH--MRAERDIS--A--G--DEI--------CISYLTPEQRHM      54 Tadhaerens(XP_002114620.1)/25-373                        17.6%     D--------------------------N------NATLT--VKAIRNIS--E--G--QEL--------CISYIDSDI--K      55 Bfloridae(XP_002609030.1-BRAFLDRAFT_84846)/1-276         11.7%     E--------------------------S------NYVLS--MRALRDIT--E--G--EEL--------FICYLDECERTR      56 cintestinalis(XP_002127168.1)/13-358                     17.5%     N--------------------------N------NHRLA--VKACRDIA--A--G--EEI--------TISYLSQCQIAR      57 Dpulex(EFX89935.1)/23-367                                16.0%     F--------------------------S------NHTVA--LVASKDLE--E--G--EEI--------FISYLDECAQSR      58 Dmelanogaster(CG3353-NP_650955.1)/13-363                 15.5%     Y--------------------------S------NDIVV--LKALAPIQ--Q--G--EEI--------CISYLDECMLER      59 Hmagnipapillata(XP_002163562.2)/21-371                   16.0%     Y--------------------------N------NSTLV--LVATEDIT--K--D--EEI--------CVCYLDECQRSR      60 Agambiae(XP_313299.1-AGAP003552-PA)/13-365               16.8%     K--------------------------S------NHMLA--LRATRDIQ--P--G--EEI--------CISYLDECNLQR      61 Amellifera(XP_394075.2-SMYD5-like-Prediction)/16-364     16.1%     Y--------------------------S------NNVLV--LKAIRDIH--P--E--EEI--------CISYLDECCLER      62 Nvectensis(XP_001627062.1)/18-370                        15.2%     K--------------------------N------NSTLV--LKALHPIK--N--G--EEI--------CISYLEECQRER      63 Skowalevskii(XP_002735533.1)/24-372                      13.9%     D--------------------------N------DAAVS--VMALQDIQ--E--N--EEI--------CISYLGECDIGR      64 Lgigantea(LOTGIDRAFT_231752)/19-367                      16.4%     H--------------------------N------NHVLT--LVALKPIQ--P--E--EEI--------YISYISECEMSR      65 Drerio(F1RET2-Smyd5)/32-380                              16.4%     E--------------------------N------NFLLH--LTALGDIG--P--G--EEI--------CISYLDCCQRDR      66 Ggallus(NP_001012912.1-SMYD5)/39-387                     16.9%     D--------------------------N------NFLLY--LTALEDIE--A--G--EEI--------CISYLDCCQRER      67 Hsapiens(Q6GMV2-SMYD5)/33-381                            15.8%     E--------------------------N------NFLLH--VTALEDIK--P--G--EEI--------CISYLDCCQRER      68 Xtropicalis(A9ULL8-SMyd5)/32-382                         16.9%     D--------------------------N------NFILH--LTALEDIQ--P--G--EEI--------CISYLDCCQRDR      69 Mbrevicollis(MONBRDRAFT_36878)/153-462                   16.1%     G--------------------------DE--------LV--ATASKPIP--A--N--SEL--------TISYGPLAVRDA      70 Mbrevicollis(MONBRDRAFT_27776)/11-280                    20.3%     G--------------------------PL--------HAQAVKALRDIA--V--G--EEL--------CHSYIDLYAP--      71 Cowczarzaki(EFW45970.2)/35-344                           21.5%     L--------------------------KS--------KRQFIRAIRDIR--A--G--EEI--------THAFTDAASP--      72 Dpulex(EFX73755.1)/45-306                                19.5%     G--------------------------FK--------LR--IQLTQELP--KLEW--DSI--------RISYIDLMNS--      73 Bfloridae(XP_002593048.1-BRAFLDRAFT_74375)/6-196         17.6%     G--------------------------PT--------LQ--IRALVDIQ--P--G--EEV--------CYSYTEKGNV--      74 Bfloridae(XP_002594298.1-BRAFLDRAFT_117670)/15-265       21.7%     G--------------------------PQ--------IQ--IRANEFIR--P--G--EQI--------FHGYIPPLLP--      75 Scerevisiae(Q12529.1)/23-365                             10.9%     G--------------------------NS--------ML--FTMNRDIK--K--D--EQI--------CIDYSGV-LDLP      76 Dmelanogaster(CG1868-NP_724802.1)/226-549                15.1%     G--------------------------CS--------VV--NYAAKDIL--E--G--EEI--------FNCYTMDYRNSL      77 Agambiae(XP_319721.4-AGAP008973-PA)/165-486              14.6%     R--------------------------AT--------LT--VHATRPIG--A--G--GEV--------FNCYGPHYRLMA      78 Athaliana(NP_174606.2)/229-550                           12.6%     S--------------------------RG--------LI--MQTTEFVP--T--G--CPL--------ELSYGPEVGKWD      79 Cintestinalis(XP_002123001.1)/195-567                    14.8%     G--------------------------ST--------VT--FRSNQFIP--V--G--AEI--------THCYGPSVFHAS      80 Drerio(Q08C84-Smyd4)/197-556                             15.4%     TGFQPDPHNQLGCSEGHFDHPKGSRSGVT--------VT--VRASKDLT--A--G--QEI--------LHCYGPHRSRME      81 Xtropicalis(NP_001072288.1-SMYD4)/212-545                18.0%     G--------------------------RF--------VT--VRANRPIR--R--D--EEV--------THCYGPHKLRMD      82 Hsapiens(Q8IYR2-SMYD4)/244-602                           16.3%     S--------------------------TV--------AT--IRASQRIR--K--G--QEI--------LHCYGPHKSRMG      83 Ggallus(NP_001025886.1-SMYD4)/241-573                    15.3%     G--------------------------TA--------AT--VRASQPIP--S--G--QEI--------FHCYGEEM----      84 Hmagnipapillata(XP_002160254.2/232-532                   15.5%     K--------------------------NQ--------LI--VKVVKDVV--K--G--EQI--------FNCYGPHFKRMG      85 Dpulex(DAPPUDRAFT_312722-Pedicted)/241-525               17.9%     G--------------------------NT--------LI--VRAIRNVR--Q--G--DEV--------FNCYGPHYRRMR      86 Amellifera(XP_006565387.1-SMYD4-like-Predicted)/278-571  17.3%     N--------------------------QY--------LI--VRASRDIS--Q--G--EEI--------FNCYGPHYRHMT      87 Bfloridae(XP_002589088.1-BRAFLDRAFT_75068)/251-714       13.4%     G--------------------------KT--------LI--VRAVSHIK--P--G--EEL--------LHCYGPHAGRMV      88 Lgigantea(LOTGIDRAFT_169490)/248-638                     12.9%     G--------------------------DT--------LI--VKSVKKVL--E--G--EEI--------YNCYGPHHKRMV      89 Skowalevskii(XP_002733823.1)/75-447                      16.7%     G--------------------------NQ--------LT--IRATEMIK--K--G--GEV--------LHCYGPRVSDMF      90 Nvectensis(XP_001627273.1)/170-547                       14.3%     D--------------------------GV--------LV--ARATHNIA--P--G--SGI--------THCYGPHVNHMP      91 Amellifera(XP_003250668.1-SMYD4-like-Predicted)/183-473  15.8%     N--------------------------NV--------LV--LRAAKDIY--P--G--ELI--------TFNFMSKYVALE      92 Cowczarzaki(XP_004349923.1)/103-371                      17.2%     F--------------------------HRPLVDGLPHFE--ARAVIDVA--E--G--TEI--------TWCYS--NIRNP      93 Amellifera(XP_001121272.2-SMYD4-like-Predicted)/230-549  19.0%     S--------------------------GI--------VV--VRTLRFVG--K--G--TEI--------LDCYGPHWFSEN      94 Amellifera(XP_003249162.1-SMYD4-like-Predicted)/239-589  15.1%     G--------------------------RC--------IV--IRAIRSLR--P--G--DVV--------AENYGPIFTKRN      95 Dmelanogaster(CG14122-NP_648574.1)/265-541               16.9%     G--------------------------KK--------LV--LTATRPHR--A--N--ELV--------AVNYGPIFIKNN      96 Agambiae(XP_311885.3-AGAP002999-PA)/268-544              17.3%     G--------------------------TA--------MI--LHTSRPIP--A--G--AVV--------PENYGPHFMRQP      97 Amellifera(XP_392262.3-SMYD4-like-Predicted)/252-555     20.2%     G--------------------------TT--------MV--VRAIRSIS--S--G--EEI--------SENYGQIFTTTP      98 Dmelanogaster(CG7759-NP_725048.1)/250-537                16.4%     G--------------------------TT--------IH--INSVRPIE--A--G--LPI--------NENYGPMYTQDE      99 Agambiae(XP_319583.4-AGAP008839-PA)/240-523              16.0%     G--------------------------NQ--------VC--VRTVKNIP--A--D--SMV--------AENYGPLFTQVR     100 Dpulex(DAPPUDRAFT_68494-Predicted)/254-551               17.2%     G--------------------------DV--------MV--VRAIKSFQ--P--G--EMV--------NENYGPIFTQKR     101 Dpulex(DAPPUDRAFT_309882)/300-599                        16.7%     G--------------------------TT--------MV--ITSTRCVK--R--G--QMV--------AENYGPIFTHKH     102 Dmelanogaster(CG8378-NP_610730.1)/196-491                17.4%     G--------------------------TK--------AY--MFVLRPIK--A--G--NVL--------YDNYGAHFAICS     103 Agambiae(XP_566179.1-AGAP000216-PA)/158-458              17.1%     D--------------------------GR--------CS--AVVIQPIA--A--G--GQL--------FANYGYDYLQTG     104 Agambiae(XP_564258.1-AGAP011234-PA)/216-546              17.1%     D--------------------------GR--------LA--MVATRPIA--A--G--EQL--------YNINGFSTFDPD     105 Agambiae(XP_309407.4-AGAP011238-PA)/219-497              17.1%     D--------------------------GR--------CA--IVAIRPIA--A--G--EQL--------FDNYG-------     106 Agambiae(XP_314169.4-AGAP005253-PB)/218-514              16.6%     D--------------------------GR--------CA--FVATRPIA--A--G--EQL--------FDVYAFASMDFD     107 Agambiae(XP_309409.4-AGAP011237-PA)/206-481              16.3%     D--------------------------GR--------CA--FVVSRPIA--A--G--EQL--------FDVYG-------     108 Agambiae(XP_307865.2-AGAP009448-PA)/166-466              16.5%     D--------------------------GR--------CA--MIVIRPIG--P--G--EQL--------FDSYGLHHFSFE     109 Agambiae(XP_309762.4-AGAP010931-PA)/113-383              17.2%     D--------------------------GR--------CA--VIVIHTVA--K--G--GQL--------FDNYE-------     110 Agambiae(XP_309378.2-AGAP011267-PA)/149-447              17.9%     D--------------------------GR--------CA--VFVIRPVL--E--G--SQL--------FDSYETDHKSHE     111 Agambiae(XP_309383.4-AGAP011257-PA)/149-447              18.5%     D--------------------------GR--------CA--VFVIRPVL--E--G--SQL--------FDSYEAGHTLHE     112 Agambiae(XP_307655.3-AGAP012638-PA)/149-447              18.5%     D--------------------------GR--------CA--VFVIRPVL--E--G--SQL--------FDSYEADHILNK     113 Agambiae(XP_320681.4-AGAP011835-PA)/183-484              16.8%     D--------------------------GR--------CA--VLVTRPIA--K--G--GQL--------YDNYGMHHCLMS     114 Agambiae(XP_309411.4-AGAP011232-PA)/162-434              16.4%     D--------------------------GR--------CA--LVVTRQIA--D--G--GQL--------FDHYE-------     115 Amellifera(XP_001120776.2-SMYD4-like-Predicted)/251-554  19.0%     G--------------------------LT--------MI--TRALKPLY--P--G--DQI--------FTSYGAAYAYMT     116 Dpulex(DAPPUDRAFT_305694-Predicted)/258-553              19.7%     G--------------------------DV--------IA--VKAIRRIA--R--G--DEI--------LDNYGYHYATHD     117 Dpulex(EFX87901.1)/258-554                               19.0%     G--------------------------DV--------MV--VKVIHRIA--R--G--EEI--------LDNYGYHYATHE     118 Amellifera(XP_001122116.2-SMYD4-like-Predicted)/234-534  17.2%     S--------------------------NY--------MI--IYVIYPIK--K--G--EQL--------YDNYGQHYAITP     119 Hmagnipapillata(XP_002159692.1)/239-485                  18.1%     G--------------------------DT--------CV--LRAIKHIK--E--G--SEI--------VDNYGFLYAVES     120 Nvectensis(XP_001623892.1)/215-512                       18.8%     G--------------------------DK--------CV--VRAFSSIP--C--R--GEV--------VDNYGILSALTP     121 Lgigantea(LOTGIDRAFT_143433)/100-395                     19.3%     G--------------------------DF--------CA--VRAIRNIP--K--G--TEV--------YDSYGALYPLTA     122 Skowalevskii(XP_002740933.1)/253-549                     20.1%     G--------------------------ET--------CV--VRAIRNIY--K--G--EEI--------TDNYGYLYPVHD         consensus/100%                                                     .....................................h.........h................................         consensus/90%                                                      .                          ..  .  ...h.  hhs.t.l.  .  s  t.l        h.sYh.......         consensus/80%                                                      .                          ..     ...hh  lhshp.l.  .  G  ppl        h.sYh..h....         consensus/70%                                                      t                          ..     ...hh  lpAhcsIt  t  G  ppl        hhsYhs.h...t                                                                         801          .         .   ] 824   1 cintestinalis(NP_001071820.1)/15-282                    100.0%     -----KRREKLKLQYYFDCECDTC       2 Drerio(Q6P0R5-Smyd1a)/18-279                             35.9%     -----DRQRLLQQQYYFDCKCEHC       3 Derio(Q2MJQ9-Smyd1b)/13-274                              34.5%     -----DRQRLLKQQYFFDCTCKHC       4 Xtropicalis(NP_001120357.1-SMYD1)/13-261                 38.9%     -----DRKAQLKKQYYFDCTCEHC       5 Hsapiens(Q8NB12-SMYD1)/18-279                            38.4%     -----ERKRQLKKQYYFDCTCEHC       6 Ggallus(NP_989486.1-SMYD1)/13-274                        39.1%     -----ERRKQLKKQYYFDCTCEHC       7 Athaliana(Q7XJS0-ASHR1)/22-274                           25.8%     -----TRQKSLKEQYLFHCQCARC       8 Amellifera(XP_625013.1-SMYD3-Predicted)/1-253            27.1%     -----DRREELQSSYYFWCNCKKC       9 Dmelanogaster(Buzidau-CG13761)/26-282                    25.6%     -----QRRLDLKEHYYFLCVCSKC      10 Agambiae(XP_319707.4-AGAP008954-PA)/1-254                25.0%     -----VRQEQLAERYYFHCACERC      11 cintestinalis(XP_002128556.1)/14-266                     32.4%     -----ERQRELMSIYHFQCTCHSC      12 Lgigantea(LOTGIDRAFT_177746)/1-216                       26.2%     -----ERLAELEEQYYFSCECSRC      13 Drerio(E7EZZ6-SMYD3)/16-267                              34.7%     -----DRRSQLQEQYHFRCECKRC      14 Xtropicalis(XP_004914684.1|-SMYD3-Predicted)/15-264      31.0%     -----GRRDQLQRQYCFLCDCQRC      15 Hsapiens(Q9H7B4-SMYD3)/15-266                            33.9%     -----ERRKQLRDQYCFECDCFRC      16 Ggallus(XP_419536.1-SMYD3-Predicted)/15-266              33.2%     -----ERQKQLKRQYCFECDCCLC      17 Drerio(Q5RGL7-Smyd2b)/19-268                             30.9%     -----DRLERLRDMYYFSCDCKEC      18 Drerio(Q5BJI7-Smyd2a)/18-267                             32.0%     -----DRIERLKDSYFFNCDCKEC      19 Xtropicalis(XP_002934751.2-SMYD2-like-Predicted)/16-265  30.9%     -----DRNDRLIDSYFFNCDCREC      20 Hsapiens(Q9NRG4-SMYD2)/18-267                            32.0%     -----DRNDRLRDSYFFTCECQEC      21 Ggallus(XP_419420.1-SMYD2-Predicted)/21-270              31.6%     -----DRNDRLRDSYFFTCDCREC      22 Tadhaerens(XP_002109888.1)/20-262                        28.4%     -----SRQNQLQSQYCFKCICERC      23 Hmagnipapillata(XP_002163555.2)/16-259                   29.4%     -----VRQLELRESYMFTCKCTVC      24 Nvectensis(XP_001627600.1)/17-253                        32.1%     -----TRQEELQTQFCFYCQCHRC      25 Bfloridae(XP_002594889.1-BRAFLDRAFT_124463)/14-258       38.4%     -----VRKQQLLQQYYFTCKCPRC      26 Skowalevskii(XP_006817727.1)/14-260                      33.2%     -----ERREHLQDQYYFQCVCHAC      27 Amellifera(XP_006565332.1)/43-285                        18.4%     -----LRRQFLNVTKNFSCMCKRC      28 Dmelanogaster(msta-CG33548)/66-313                       17.7%     -----TRNIFLKMTKHFACDCVRC      29 Dmelanogaster(CG12119)/34-280                            19.0%     -----ARHLFLKMKKSFTCKCSRC      30 Amellifera(XP_006565301.1)/26-284                        23.2%     -----GRREYLRTGKYFECECDLC      31 Dmelanogaster(CG9642)/21-271                             18.8%     -----ERRKILKVGKCFDCSCPRC      32 Dmelanogaster(CG9640)/17-268                             16.9%     -----QRQHQLRLSRRLECICSRC      33 Amellifera(NP_001229486.1-LOC724300)/57-301              21.5%     -----VRRSHLLETKYFSCHCKRC      34 Dmelanogaster(CG14590-NP_610202.3)/55-322                18.9%     -----LRQHHLRLTKHFSCRCSRC      35 Dmelanogaster(CG43129)/21-279                            17.1%     -----QRQKHLKQGKFFTCQCERC      36 Dmelanogaster(G11160)/58-319                             20.8%     -----KRRAFMHEGKLFWCCCRRC      37 Amellifera(XP_624539.3-msta-like-Predicted)/54-297       21.5%     -----NRRHHLFKTKFFECICNRC      38 Dmelanogaster(CG8503-NP_610944.1)/52-301                 20.0%     -----DRQRHLMQTKLFKCACERC      39 Agambiae(XP_309979.4-AGAP011530-PA)/50-300               22.8%     -----NRLEHLQQTKMFRCECERC      40 Dpulex(DAPPUDRAFT_120473)/58-292                         19.1%     -----ERRDVLRHSKLFECDCSRC      41 Dpulex(DAPPUDRAFT_194440-Predicted)/53-302               19.6%     -----NRQRHLSQTKFFQCVCERC      42 Dpulex(DAPPUDRAFT_2393)/50-297                           20.2%     -----DRRLHLKSTKHFWCQCSRC      43 Dmelanogaster(CG18136-NP_649084.1)/58-318                22.6%     -----QRRVHLRQAKCFDCSCARC      44 Agambiae(XP_309220.5-AGAP001025-PA)/55-318               20.7%     -----QRRLALRQAKCFDCCCERC      45 Scerevisiae(P38890.1-SET5)/124-429                       16.1%     -----LRRRELRVNWGFLCQCDRC      46 Athaliana(Q9ZUM9-ASHR2)/22-296                           14.0%     -----SRQKRLLEDYGFKCDCDRC      47 Athaliana(Q9FG08.2-ATXR4)/53-321                         16.2%     -----ARQTILSQGFGFLCNCLRC      48 Lgigantea(LOTGIDRAFT_232186)/323-670                     15.0%     SRN--IRRAWLYKSFNFWCQCPQC      49 Bfloridae(XP_002589246.1-BRAFLDRAFT_74594)/380-720       14.2%     QRP--QRRAWLYRAYHFWCECQRC      50 Athaliana(Q5PP37-ATXR2)/52-466                           14.1%     --K--ER-QALLADYGFSCKCSKC      51 Cowczarzaki(EPH53581.1)/160-496                          15.7%     PKA--KRQAALEELYYFSCSCAKC      52 Mbrevicollis(MONBRDRAFT_29283)/14-364                    16.5%     GGD--VRREVLREQYLFECSCVRC      53 Cowczarzaki(EFW42079.2)/57-422                           15.4%     RRS--RRMATLRENYLFMCACAKC      54 Tadhaerens(XP_002114620.1)/25-373                        17.6%     NWK--KRQAILMENYLFECTCNRC      55 Bfloridae(XP_002609030.1-BRAFLDRAFT_84846)/1-276         11.7%     SRH--SRQKLLRENYLFSCTCEKC      56 cintestinalis(XP_002127168.1)/13-358                     17.5%     GCR--SRQQYLKENYLFHCCCSKC      57 Dpulex(EFX89935.1)/23-367                                16.0%     SRH--SRRKILKENYLFHCNCSRC      58 Dmelanogaster(CG3353-NP_650955.1)/13-363                 15.5%     SRH--SRHKVLRENYVFICQCPKC      59 Hmagnipapillata(XP_002163562.2)/21-371                   16.0%     SRH--SRRKLLRENYLFECTCSLC      60 Agambiae(XP_313299.1-AGAP003552-PA)/13-365               16.8%     SRH--SRQKTLKDYYLFICQCEKC      61 Amellifera(XP_394075.2-SMYD5-like-Prediction)/16-364     16.1%     SRH--SRQKALNSLYLFQCYCNKC      62 Nvectensis(XP_001627062.1)/18-370                        15.2%     SRH--SRLKYLRENYIFDCTCTKC      63 Skowalevskii(XP_002735533.1)/24-372                      13.9%     SRH--SRQKILRENYLFNCNCMKC      64 Lgigantea(LOTGIDRAFT_231752)/19-367                      16.4%     SRH--SRQKILRENYLFTCRCRKC      65 Drerio(F1RET2-Smyd5)/32-380                              16.4%     SRH--SRHKILRENYLFICSCQKC      66 Ggallus(NP_001012912.1-SMYD5)/39-387                     16.9%     SRH--SRNKILRENYLFTCSCPKC      67 Hsapiens(Q6GMV2-SMYD5)/33-381                            15.8%     SRH--SRHKILRENYLFVCSCPKC      68 Xtropicalis(A9ULL8-SMyd5)/32-382                         16.9%     SRH--SRQKILRENYLFVCSCPKC      69 Mbrevicollis(MONBRDRAFT_36878)/153-462                   16.1%     WHA--ARQTQLRNTFNFACQCIAC      70 Mbrevicollis(MONBRDRAFT_27776)/11-280                    20.3%     -TG--QRHSHLGDQYGFECDCALY      71 Cowczarzaki(EFW45970.2)/35-344                           21.5%     -TV--VRKAHLKSLYAFDCNCSRC      72 Dpulex(EFX73755.1)/45-306                                19.5%     -KS--HRKKELKDRYYFDCDCPRC      73 Bfloridae(XP_002593048.1-BRAFLDRAFT_74375)/6-196         17.6%     -TH--ERR-DELRKYFFECQCPHC      74 Bfloridae(XP_002594298.1-BRAFLDRAFT_117670)/15-265       21.7%     -TA--KRQEKLLKTYHFLCQCADC      75 Scerevisiae(Q12529.1)/23-365                             10.9%     -TV--KRRAFLADSWFFDCACERC      76 Dmelanogaster(CG1868-NP_724802.1)/226-549                15.1%     -KL--QRSHPLKAIYKFECTCAKC      77 Agambiae(XP_319721.4-AGAP008973-PA)/165-486              14.6%     -AA--ERKMLLRAQYCFECGCERC      78 Athaliana(NP_174606.2)/229-550                           12.6%     -CK--NRIRFLEEEYFFHCRCRGC      79 Cintestinalis(XP_002123001.1)/195-567                    14.8%     -FE--ERQKTLKENYSFDCDCTPC      80 Drerio(Q08C84-Smyd4)/197-556                             15.4%     -VK--ERQRLLLEQYFFQCVCQAC      81 Xtropicalis(NP_001072288.1-SMYD4)/212-545                18.0%     -VA--ERQQLLKDQYFFVCQCKAC      82 Hsapiens(Q8IYR2-SMYD4)/244-602                           16.3%     -VA--ERQQKLRSQYFFDCACPAC      83 Ggallus(NP_001025886.1-SMYD4)/241-573                    15.3%     ------------------------      84 Hmagnipapillata(XP_002160254.2/232-532                   15.5%     -YE--DRRAALMQQYFFLCSCEHC      85 Dpulex(DAPPUDRAFT_312722-Pedicted)/241-525               17.9%     -RS--ERVEALEAQYSFTCTCDSC      86 Amellifera(XP_006565387.1-SMYD4-like-Predicted)/278-571  17.3%     -TE--NRQKILKNQYCFICKCKAC      87 Bfloridae(XP_002589088.1-BRAFLDRAFT_75068)/251-714       13.4%     -YG--ERQAALKEQYFFSCSCDAC      88 Lgigantea(LOTGIDRAFT_169490)/248-638                     12.9%     -RK--RRQEVLENQYFFHCKCPPC      89 Skowalevskii(XP_002733823.1)/75-447                      16.7%     -RD--ERLKVLRDQYYFTCKCMFC      90 Nvectensis(XP_001627273.1)/170-547                       14.3%     -RE--ERQKLLYKQYFFTCQCSAC      91 Amellifera(XP_003250668.1-SMYD4-like-Predicted)/183-473  15.8%     -SNSMPRNVMLKNFFDISCDCEAC      92 Cowczarzaki(XP_004349923.1)/103-371                      17.2%     -KA--ERREHLRQFYGFLCECPRC      93 Amellifera(XP_001121272.2-SMYD4-like-Predicted)/230-549  19.0%     -KL--SRIEYLWKKYRFLCTCDAC      94 Amellifera(XP_003249162.1-SMYD4-like-Predicted)/239-589  15.1%     -LE--ERRRNLAGRYWFFCECNAC      95 Dmelanogaster(CG14122-NP_648574.1)/265-541               16.9%     -LK--ERQRSLRGRYSFSCSCMAC      96 Agambiae(XP_311885.3-AGAP002999-PA)/268-544              17.3%     -KA--IRQRNLRSRYWFKCDCRAC      97 Amellifera(XP_392262.3-SMYD4-like-Predicted)/252-555     20.2%     -ES--ERKRKLRLQYFFDCNCEAC      98 Dmelanogaster(CG7759-NP_725048.1)/250-537                16.4%     -RS--ERQARLKDLYWFECSCDAC      99 Agambiae(XP_319583.4-AGAP008839-PA)/240-523              16.0%     -RD--ERRDTLLHQYRFTCQCVPC     100 Dpulex(DAPPUDRAFT_68494-Predicted)/254-551               17.2%     -RV--DRQRSLKDRYWFDCRCNPC     101 Dpulex(DAPPUDRAFT_309882)/300-599                        16.7%     -LT--DRQQSLQGRYWFNCQCLAC     102 Dmelanogaster(CG8378-NP_610730.1)/196-491                17.4%     -KE--QRLKRLSLQYRFDCKCEGC     103 Agambiae(XP_566179.1-AGAP000216-PA)/158-458              17.1%     -RD--ERREGLQRVFGFTCNCDAC     104 Agambiae(XP_564258.1-AGAP011234-PA)/216-546              17.1%     -DS--ARRHALQLSHFFKCRCASC     105 Agambiae(XP_309407.4-AGAP011238-PA)/219-497              17.1%     ------------------------     106 Agambiae(XP_314169.4-AGAP005253-PB)/218-514              16.6%     -RS--FRIFCLRKSYYFKCRCAVC     107 Agambiae(XP_309409.4-AGAP011237-PA)/206-481              16.3%     ------------------------     108 Agambiae(XP_307865.2-AGAP009448-PA)/166-466              16.5%     -RS--HRQKGTFVMFNFECCCEAC     109 Agambiae(XP_309762.4-AGAP010931-PA)/113-383              17.2%     ------------------------     110 Agambiae(XP_309378.2-AGAP011267-PA)/149-447              17.9%     -RA--MRQLMLSFTYSFRCTCEAC     111 Agambiae(XP_309383.4-AGAP011257-PA)/149-447              18.5%     -RE--MRQSMLSFTYSFRCTCEAC     112 Agambiae(XP_307655.3-AGAP012638-PA)/149-447              18.5%     -RA--MRQSMLSFMYSFRCTCEAC     113 Agambiae(XP_320681.4-AGAP011835-PA)/183-484              16.8%     -RK--ERKTELLKQYRFICECEAC     114 Agambiae(XP_309411.4-AGAP011232-PA)/162-434              16.4%     ------------------------     115 Amellifera(XP_001120776.2-SMYD4-like-Predicted)/251-554  19.0%     -RS--ERREKIMQDYFFECDCIAC     116 Dpulex(DAPPUDRAFT_305694-Predicted)/258-553              19.7%     -KK--ERQLKLSQQYYFRCNCLAC     117 Dpulex(EFX87901.1)/258-554                               19.0%     -KR--ERQLKLCQQYYFRCRCQSC     118 Amellifera(XP_001122116.2-SMYD4-like-Predicted)/234-534  17.2%     -KE--ERQKELLKQYYFKCNCLAC     119 Hmagnipapillata(XP_002159692.1)/239-485                  18.1%     -KV--IRQSHLMEQYYFACQCEAC     120 Nvectensis(XP_001623892.1)/215-512                       18.8%     -RK--QRQESLQSQYYFKCNCHAC     121 Lgigantea(LOTGIDRAFT_143433)/100-395                     19.3%     -KK--DRQEKLLSQYFFKCSCKAC     122 Skowalevskii(XP_002740933.1)/253-549                     20.1%     -KS--ERQTRLKWQYFFECKCDAC         consensus/100%                                                     ........................         consensus/90%                                                      ...  .R...L...h.F.C.C..C         consensus/80%                                                       ..  tRpt.L.ttahF.CpC.tC         consensus/70%                                                       ..  pRpphLpppYhFpCpCttC ``` |
