# Supplementary material for: Evolutionary History of the Smyd Gene Family in Metazoans: A Framework to Identify the Orthologs of Human Smyd Genes in Drosophila and Other Animal Species
Source: PLoS One. 2015 Jul 31;10(7):e0134106. doi: 10.1371/journal.pone.0134106 (PMC4521844; doi:10.1371/journal.pone.0134106)
Supplement: S1 Table — (DOCX) [file pone.0134106.s011.docx]

| SPECIES | Nº | ACCESSION NUMBERS |
| --- | --- | --- |
| **Metazoan species** | | |
| *T. adhaerens* | 2 | GenBank:XP_002109888.1, GenBank:XP_002114620.1 |
| *H. magnipapillata* | 4 | GenBank:XP_002159692.1, GenBank:XP_002160254.2, GenBank:XP_002163555.2, GenBank:XP_002163562.2 |
| *N. vectensis* | 4 | GenBank:XP_001623892.1, GenBank:XP_001627273.1, GenBank:XP_001627600.1, GenBank:XP_001627062.1, |
| *L. gigantea* | 5 | GenBank:LOTGIDRAFT_143433, GenBank:LOTGIDRAFT_169490, GenBank:LOTGIDRAFT_177746, GenBank:LOTGIDRAFT_232186, GenBank:LOTGIDRAFT_231752 |
| *D. pulex* | 10 | GenBank:DAPPUDRAFT_312722, GenBank:EFX73755.1, GenBank:DAPPUDRAFT_305694, GenBank:EFX87901.1, GenBank:DAPPUDRAFT_68494, GenBank:EFX89935.1, GenBank:DAPPUDRAFT_194440, GenBank:DAPPUDRAFT_2393, GenBank:DAPPUDRAFT_120473, GenBank:DAPPUDRAFT_309882 |
| *D. melanogaster* | 15 | GenBank: NP_001014717.1, GenBank: NP_572539.2, GenBank: NP_611182.1, GenBank: NP_725048.1, GenBank: NP_610730.1, GenBank: NP_648574.1, GenBank: NP_724802.1, GenBank: NP_650955.1, GenBank: NP_649084.1-, GenBank: NP_610944.1, GenBank: NP_524768.2, GenBank: NP_609464.1, GenBank: NP_611181.1, GenBank: NP_572675.1, GenBank: NP_610202.3 |
| *A. mellifera* | 13 | GenBank:XP_006565332.1, GenBank:XP_006565301.1, GenBank:XP_001122116.2, GenBank:XP_392262.3, GenBank:XP_001120776.2, GenBank:XP_001121272.2, GenBank:XP_006565387.1, GenBank:XP_003250668.1, GenBank:XP_625013.1, GenBank:XP_624539.3, GenBank:XP_394075.2, GenBank:NP_001229486.1, GenBank:XP_003249162.1 |
| *A. gambiae* | 19 | GenBank:XP_313299.1, GenBank:XP_319707.4, GenBank:XP_309979.4, GenBank:XP_311885.3, GenBank:XP_309383.4, GenBank:XP_307655.3, GenBank:XP_319583.4, GenBank:XP_319721.4, GenBank:XP_320681.4, GenBank:XP_314169.4, GenBank:XP_307865.2, GenBank:XP_309407.4, GenBank:XP_564258.1, GenBank:XP_309220.5, GenBank:XP_309378.2, GenBank:XP_309762.4, GenBank:XP_309409.4, GenBank:XP_309411.4, GenBank:XP_566179.1 |
| *S. kowalevskii* | 4 | GenBank:XP_002733823.1, GenBank:XP_006817727.1, GenBank:XP_002740933.1, GenBank:XP_002735533.1 |
| *C. intestinalis* | 4 | GenBank:XP_002127168.1, GenBank:NP_001071820.1, GenBank:XP_002128556.1, GenBank:XP_002123001.1 |
| *B. floridae* | 6 | GenBank:XP_002589088.1, GenBank:XP_002593048.1, GenBank:XP_002594889.1, GenBank:XP_002594298.1, GenBank:XP_002589246.1, GenBank:XP_002609030.1, |
| *H. sapiens* | 5 | Swiss-Prot:Q8NB12, Swiss-Prot:Q9NRG4, Swiss-Prot:Q9H7B4, Swiss-Prot:Q8IYR2, Swiss-Prot:Q6GMV2 |
| *G. gallus* | 5 | GenBank:NP_001025886.1, GenBank:XP_419420.1, GenBank:XP_419536.1, GenBank:NP_989486.1, GenBank: NP_001012912.1 |
| *X. tropicalis* | 5 | GenBank:NP_001072288.1, GenBank:XP_002934751.2, GenBank:NP_001120357.1, GenBank:XP_004914684.1, Swiss-Prot:A9ULL8 |
| *D. rerio* | 7 | Swiss-Prot:Q6P0R5, Swiss-Prot:Q2MJQ9, Swiss-Prot:Q5BJI7, Swiss-Prot:Q5RGL7, Swiss-Prot:E7EZZ6, Swiss-Prot:Q08C84, Swiss-Prot:F1RET2 |
| **Non-metazoan species** | | |
| *S. cerevisiae* | 2 | Swiss-Prot:P38890.1, Swiss-Prot:Q12529.1 |
| *A. thaliana* | 5 | Swiss-Prot:Q7XJS0, Swiss-Prot:Q9ZUM9, Swiss-Prot:Q9FG08.2, GenBank:NP_174606.2, Swiss-Prot:Q5PP37 |
| *C. owczarzaki* | 4 | GenBank:EFW45970.2, GenBank:XP_004349923.1, GenBank:EFW42079.2, GenBank:EPH53581.1 |
| *M. brevicollis* | 3 | GenBank:MONBRDRAFT_36878, GenBank:MONBRDRAFT_27776, GenBank:MONBRDRAFT_29283 |
